# Diatoms (Bacillariophyta) of the Salish Sea, Northeast Pacific: annotated checklist and new species reports

**DOI:** 10.3897/BDJ.14.e189060

**Published:** 2026-05-15

**Authors:** Mark Webber, Elaine Humphrey, Arjan van Asselt, Alice Chang, Evan Morien, Andrew D. F. Simon

**Affiliations:** 1 Institute for Multidisciplinary Ecological Research in the Salish Sea, Galiano Island, Canada Institute for Multidisciplinary Ecological Research in the Salish Sea Galiano Island Canada; 2 Advanced Microscopy Facility, Bob Wright Centre, University of Victoria, Victoria, Canada Advanced Microscopy Facility, Bob Wright Centre, University of Victoria Victoria Canada; 3 Department of Earth, Ocean and Atmospheric Sciences, University of British Columbia, Vancouver, Canada Department of Earth, Ocean and Atmospheric Sciences, University of British Columbia Vancouver Canada; 4 Hakai Institute, Heriot Bay, Canada Hakai Institute Heriot Bay Canada; 5 Department of Botany and Biodiversity Research Centre, University of British Columbia, Vancouver, Canada Department of Botany and Biodiversity Research Centre, University of British Columbia Vancouver Canada; 6 University of Alberta, Edmonton, Canada University of Alberta Edmonton Canada

**Keywords:** biodiversity, eDNA, light microscopy, marine microbiome, metabarcoding, phytoplankton, scanning electron microscopy

## Abstract

**Background:**

Diatoms are major primary producers known to respond rapidly to environmental change, making them useful indicators for ecological assessment and monitoring. In the Salish Sea bioregion, diatom records date back to early inventories by Lord (1866) and Bailey and MacKay (1916), followed by scattered surveys throughout the 20^th^ and 21^st^ centuries. Due to this fragmented record, a consolidated regional baseline has been lacking.

**New information:**

We report 924 diatom taxa for the Salish Sea from historical records, voucher specimens and molecular data, forming a curated dataset of 11,469 records. Forty-two species, including six previously unreported genera, are newly recorded for the region, based on combined morphological and molecular evidence. For these taxa, including the recently described *Andrzeja fenestrata*, we provide taxonomic accounts with diagnostic light and electron micrographs. These data establish a baseline record of diatom diversity in the Salish Sea, setting an example for how collaboration amongst community and academic researchers can help establish data foundations for phytoplankton monitoring.

## Introduction

The Salish Sea is a biodiverse and culturally significant marine ecosystem spanning the coastal waters of south-western British Columbia (BC), Canada, and north-western Washington, USA. Encompassing the Georgia Basin and Puget Sound, this transboundary region forms the traditional territories of Coast Salish peoples, whose stewardship has shaped—and continues to shape—these lands and waters since time immemorial. Today, the Salish Sea, one of six distinct ecoregions within the Cold Temperate Northeast Pacific Province (Spalding et al. 2007), supports nearly nine million people and faces intensifying pressures from urbanisation, industrial activity, and habitat degradation (Sobocinski 2021). Thus, it has been recognized as a globally significant *urban sea*, where dense human populations intersect with highly productive coastal ecosystems (Sobocinski et al. 2022). At this intersection, the region represents an important setting for expanding plankton monitoring networks (United Nations Global Compact 2024), where diatoms—organisms known to respond rapidly to environmental variability (Dixit et al. 1992, Lowery et al. 2020, Sarker et al. 2023, Viljoen et al. 2024)—serve as key biological indicators.

Diatoms (Bacillariophyta) form a dominant lineage of photosynthetic eukaryotes within the Ochrophyta (stramenopiles), a group that ranks amongst the most abundant and species-rich components of aquatic ecosystems. While they are commonly regarded as planktonic primary producers, the majority of their diversity is associated with benthic habitats (Nakov et al. 2018). Currently divided into ten classes (Kociolek et al. 2026), more than 18,000 diatom species have been formally described (Guiry 2024), with total diversity estimated to be much higher — potentially reaching 100,000–200,000 species (Adl et al. 2005, Mann and Vanormelingen 2013, Kociolek and Williams 2015, Mann et al. 2016, Simpson and Eglit 2016, Benoiston et al. 2017, Kociolek et al. 2018, WoRMS Editorial Board 2021).

Diatoms are predominantly unicellular algae, although some taxa form chains of connected cells. Individual cells typically range from 5–200 µm in length, with larger species occasionally reaching 1–2 mm. Diatoms occupy an extensive range of sunlit environments wherever nutrients are available, including marine and freshwater systems and, in some cases, solid substrates, such as soils and even atmospheric particulates. They are amongst the most ecologically successful and productive organisms on Earth, generating much of the organic matter that forms the base of aquatic food webs. Estimated to account for approximately 40% of marine primary production, diatoms contribute about 20% of the global photosynthetic output annually and are responsible for approximately 20–40% of the Planet’s oxygen generation (Field et al. 1998, Benoiston et al. 2017, Tréguer et al. 2018). They also play a pivotal role in global biogeochemical cycling — particularly in the cycling of silica, iron and nitrogen — and mediate the transfer of organic carbon and biogenic silica from surface waters to oceanic sediments and other aquatic depositional environments (Nelson et al. 1995, Falkowski et al. 1998, Kooistra et al. 2007, Armbrust 2009, Hoppenrath et al. 2009, Gugi et al. 2015, Medlin 2015, Cermeño 2016).

Fossil evidence and molecular phylogenies indicate that diatoms originated over 200 million years ago (Ma), likely during the early Mesozoic era and became dominant components of marine microalgae communities by the early Cretaceous period (ca. 110–115 Ma) (Vardi et al. 2009, Nakov et al. 2018, Behrenfeld et al. 2021, Pietluch et al. 2024). Yet their deeper evolutionary roots trace back further to the origins of the Ochrophyta, which emerged during the Mesoproterozoic (approximately 1–1.6 billion years ago) in a secondary endosymbiotic event that gave rise to the plastids shared amongst diatoms, brown algae (Phaeophyceae) and other stramenopiles (Armbrust 2009, Medlin 2015, Cermeño 2016, Benoiston et al. 2017, Pietluch et al. 2024).

Diatoms have intricately patterned cell walls (frustules) composed of amorphous silica, embedded proteins (frustulins, pleuralins, silaffins), mono- and polysaccharides (fucose, rhamnose, galactose, mannose, glucuronic acid, xylose) and long-chain polyamines. The external surface is further coated with exopolysaccharides and some genera also incorporate β-chitin in spines and excreted fibres. Each cell has two halves (valves) that fit together like a Petri dish and are connected by girdle bands. Valves contain intricate chambers, struts, tubes and partitions, with numerous pores for gas exchange, nutrient absorption and chemical signalling. These biological processes involve calcium ions, nitric oxide, oxylipins and aldehydes that mediate environmental sensing, defence, population dynamics, sexual reproduction and apoptosis. Frustule shape and patterning are diverse, yet relatively conserved within species, making morphological characters a key basis for taxonomy, typically corroborated by molecular analyses (Kooistra et al. 2007, Vardi 2008, Hoppenrath et al. 2009, Mann and Vanormelingen 2013, Nanjappa et al. 2014, Gugi et al. 2015, Kociolek and Williams 2015, Medlin 2015, Mann et al. 2016, Vasselon et al. 2017, Li et al. 2018).

As major primary producers in marine food webs, diatoms support fisheries that have sustained Coast Salish peoples for millennia (Carlson 2001). Yet, the earliest documented records of diatoms in the Salish Sea date back to the late 19^th^ and early 20^th^ centuries, beginning with the work of Lord (1866) and Bailey and MacKay (1916). Subsequent research along the west coast of North America expanded throughout the 20^th^ century, with extensive taxonomic and ecological studies by Gran and Angst (1931), Phifer (1932), Cupp (1943), Buchanan (1966), Rao and Lewin (1976), Shim (1976), and Tynni (1986), amongst others. Given the ecological importance of diatoms, integrating these fragmented records into a comprehensive regional baseline is critical amid intensifying pollution and habitat change. To support ongoing research and monitoring, we present an annotated checklist of Salish Sea diatoms, including new records that are verified from morphological and molecular data. This growing dataset, including taxonomic diagnostics, imagery, and references, provides a foundation for the continued study and monitoring of diatom diversity in the Northeast Pacific.

## Materials and methods

### Study area

**Figure 1.**
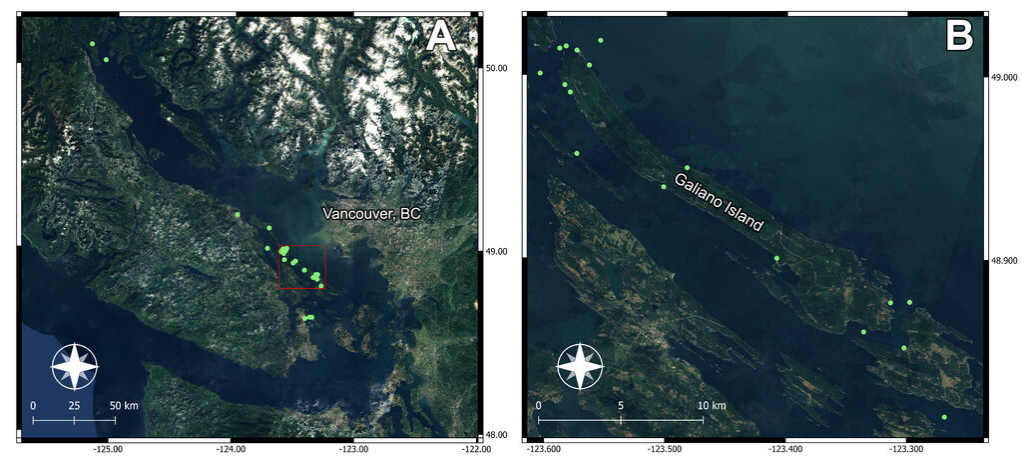
Map of sampling locations (sampling details in Table 1). **A** Distribution of sampling sites throughout the Salish Sea (green markers). Red frame: area of enlargement around Galiano Island (1B); **B** Sampling sites around Galiano Island (green markers). Imagery: Sentinel-2 cloudless map of the world by EOX IT Services GmbH (Austria; https://s2maps.eu); contains modified Copernicus Sentinel imagery from 2016–2017 (Sentinel Hub 2025).

The Salish Sea, one of the world’s largest and most biologically diverse inland seas, encompasses roughly 7,000 km of coastline, hundreds of islands, and a network of oceanic and estuarine channels ranging from shallow to deep (Sobocinski 2021). Its waters extend from northern Vancouver Island through the southern Gulf Islands in the Strait of Georgia, British Columbia, to the San Juan Islands in Puget Sound, Washington State (Fig. 1). Circulation is driven by Pacific Ocean inflows via the Strait of Juan de Fuca and Johnstone Strait and by freshwater outflows from the Fraser and other rivers, forming a stratified, estuarine system.

Sampling was concentrated around Galiano Island (48.9236, -123.4415), within the traditional territories of Hul’qumi’num- and SENĆOŦEN-speaking peoples, with additional sites distributed more broadly across the Salish Sea (Table 1), including Quadra Island, in the territories of the We Wai Kai, Wei Wai Kum, K’ómoks, Klahoose and Xwemalkhwu First Nations. Bounded to the north and south by the major tidal channels Porlier Pass and Active Pass, Galiano Island lies at the interface between waters more directly influenced by Pacific inflow via the Trincomali Channel to the southwest and more stratified, freshwater-influenced waters of the Strait of Georgia to the northeast. Intertidal habitats around the island are dominated by rocky shores formed from sandstones, conglomerates, and shales of the Upper Cretaceous Nanaimo Group (Johnstone et al. 2006). Interspersed shell, sand and mud beaches, together with brackish intertidal zones at the mouths of seasonal and perennial creeks (Simon et al. 2022), provide a diversity of soft-sediment and low-salinity habitats.

### Sample collection and preservation

We collected planktonic, epiphytic, benthic and epipsammic diatoms, sampling the water column with 20-µm and occasionally 60-µm mesh nets and swabbing biofilms from eelgrass (*Zostera marina*) (Jackman et al. 2025), macroalgae, sand and mud. All samples, including host algal substrates, were preserved in formalin (4% final concentration) and stored at 4°C. For microscopy, we brushed eelgrass, macroalgae and other substrates into a small pool of 0.2-µm-filtered marine water using a medium-firm toothbrush. After initial collection, all samples were soaked in deionised and distilled water (dH₂O) to remove salts.

Diatom frustules were typically treated with 10% hydrochloric acid, rinsed with dH₂O and cleaned with 30% hydrogen peroxide to remove organics. When samples contained substantial organic debris, such as fragments of macroalgae or eelgrass, sediment or concentrated mixed phytoplankton, we used a stronger digestion consisting of a 1:1 mixture of concentrated nitric and sulphuric acids and heated in a water bath at 95–100°C for 1.5–5 hours. In some cases, samples were subsequently treated again with 30% hydrogen peroxide, followed by multiple rinses in dH₂O to reach a pH of 6.5–7.

Cleaning times varied with the robustness of the frustules and the amount of organic matter present. Planktonic samples, which typically contained fewer adherent materials, often required noticeably shorter digestion times than macroalgal, benthic or sediment-associated samples. For taxa with lightly silicified frustules, we sometimes digested with only 30% hydrogen peroxide at room temperature for 7–10 days to avoid damaging the frustules (Webber et al. 2011).

For light microscopy (LM), we pipetted samples on to 22 × 22- or 18 × 18-mm glass coverslips and air dried and attached them to glass slides prepared with Naphrax (Brunel Microscopes, UK). We captured LM micrographs using either a Nikon Eclipse TE300 microscope that was equipped with a Tuscen DigiRetna 16-megapixel camera or a Nikon Eclipse E800 microscope that was equipped with bright-field or differential interference contrast (DIC) PlanApo 60× and 100×/1.40 objectives and an NA 1.40 oil condenser, unless otherwise noted and paired with a Zeiss AxioCam MRc5 digital camera (Jackman et al. 2025). For scanning electron microscopy (SEM), we pipetted cleaned samples directly on to 13-mm aluminium SEM stubs or on to 12-mm round glass coverslips that were then mounted on to the 13-mm aluminium stubs.

We isolated living diatoms with an inverted microscope, using drawn sterile Pasteur pipettes or pipettors. We repeatedly rinsed the diatoms in sterile drops of marine water and grew them in f/2 or f/10 media (Algae Research and Supply, Vista, California, USA; Guillard and Ryther (1962)), with the occasional addition of 1–2% antibiotic-antimycotic solution (A5955, Sigma-Aldrich, St. Louis, Missouri, USA) and, infrequently, HESNW growth media (Canadian Center Sfor the Culture of Microorganisms, University of British Columbia, Vancouver, Canada). We grew isolates in styrene well plates, Petri dishes or culture flasks and, depending on the original environment, in temperatures between 11 and 17°C, under 50–80 μmol photons m^-2^ s^-1^ (12:12h light/dark cycle) in an incubator. Successful clones were: 1) imaged live; 2) prepared in 3 × 1.5-ml polytubes for DNA bar coding in 95% ethanol or RNALater® reagent (Thermo Fisher Scientific, USA) and 3) stored in a final concentration of 4% formalin. When time permitted, clones were: 4a) set on to slides with Naphrax and 4b) prepared on SEM stubs.

We cleaned diatom clones that were grown in f/2 or other culture media and had delicate frustules with 30% hydrogen peroxide at room temperature for 5–10 days. We then rinsed the samples to remove salts and either prepared them for LM as described above or filtered them by syringe on to 13-mm-diameter polycarbonate filters (0.8–3-µm pore size), dried them and mounted them on to 13-mm SEM stubs. For both LM and SEM, we occasionally cleaned specimens directly on square or 12-mm round coverslips following Trobajo and Mann (2019). All stubs were sputter-coated with gold and imaged using either a Hitachi S-4800 field-emission SEM or a Hitachi TM4000Plus tabletop SEM at the Advanced Microscope Facility (AMF), University of Victoria (UVic), Victoria, Canada.

For in situ, environmentally prepared samples, we collected 8–10 mm sections of eelgrass leaves or macroalgae, soaked them in dH₂O to remove salts and dehydrated them through an ethanol series (50–100%), followed by three treatments of 100% hexamethyldisilazane (HMDS) (Hazrin-Chong and Manefield 2012, Jackman et al. 2025). Samples were mounted on to conductive carbon stickers (Ted Pella Inc., USA) that were affixed to 13-mm SEM stubs and imaged with the aforementioned SEM models at AMF, UVic. All preserved samples, slides and SEM stubs were deposited in the Webber Lab (IMERSS) collection on Galiano Island, BC or SEM stubs were stored temporarily for use at the AMF, UVic.

For molecular analyses, we concentrated samples by centrifugation into at least three 1.5-ml Eppendorf tubes, two of which were retained at the IMERSS lab as vouchers. We preserved the sub-samples in 95% ethanol (Linke et al. 2010, Lang and Kaczmarska 2011) or 50–60% RNAlater® (Hamilton et al. 2015) and stored them at -18 to -22°C.

### Taxonomy and Nomenclature

We followed the valve terminology of Anonymous (1975), Ross et al. (1979), Round et al. (1990) and recent literature where terminology has been revised for specific genera. For *Cocconeis* species, sternum and raphe-sternum valves are abbreviated SV and RSV, respectively, and striae were counted at the valve centre along the apical *hyaline* area and on the opposite margin (Romero 1996).

We verified species and varieties with reference to valid binomials and authorities listed in AlgaeBase (Guiry and Guiry 2025) as the primary reference, adopting the same system of classification used in this resource. Where names could not be resolved through AlgaeBase, we consulted the World Register of Marine Species (WoRMS Editorial Board 2021) and DiatomBase (Kociolek et al. 2018). All taxonomic databases were accessed in 2024 and 2025. For class-level organization, however, taxa were arranged within the three-class diatom framework of Round et al. (1990), with all taxa assigned to Coscinodiscophyceae, Fragilariophyceae, or Bacillariophyceae. This framework was adopted to facilitate a more intuitive morphological navigation of the checklist.

### Literature review

We summarised species reported for the Salish Sea from all available references that mentioned diatoms, beginning with Lord (1866). Using online searches, library catalogues and private literature collections of researchers, we included published sources from books, journals, government technical reports, such as Mather et al. (2010) and graduate student theses. All sources that had relevant information are cited in the annotated checklist (Suppl. material 1). We excluded private publications, personal communications and general references.

### DNA extraction, PCR and molecular sequencing

We used Illumina metabarcoding and Sanger sequencing to sequence diatom samples from: 1) a published eelgrass epiphyte study (amplicon sequencing, Jackman et al. (2025)) (European Nucleotide Archive accession: PRJEB72893); 2) macroalgal epiphytes collected during the 2023 Galiano Island BioBlitz (amplicon) (ENA accession: PRJEB102646); 3) plankton tows (amplicon) (ENA accession: PRJEB102646) and 4) isolated diatom clones (Sanger sequencing) (ENA accession: ERZ29258993). DNA was extracted using the DNeasy PowerSoil Pro Kit (QIAGEN, Hilden, Germany; catalogue number 47014) following the manufacturer’s instructions. Extraction blanks were included to detect potential contamination. DNA from each sample was amplified by PCR, using two primer sets to target general eukaryotes (18S) and diatoms (rbcL), which all contained Illumina adaptors and Golay barcodes, as follows:

- 18S: E572F CYGCGGTAATTCCAGCTC, E1009R and AYGGTATCTRATCRTCTTYG (Caporaso et al. 2012);
- rbcL: Diat_rbcL_708F AGGTGAAGTTAAAGGTTCATACTTDAA, R3 CCTTCTAATTTACCAACAACTG (Vasselon et al. 2017).

Library preparation and sequencing followed the procedures described in Jackman et al. (2025), the only exception being that the PCR chloroplast blocker was not used.

### Sequence processing and taxonomic assignment

We processed raw amplicon reads for each amplicon, following the length-variable ITS workflow for DADA2 (v.1.24.0; Callahan et al. (2016)) in R (v.4.2.2; R Core Team (2023)), except where noted. We used Cutadapt (v.4.0; Martin (2011)) to remove primers and DADA2 using default parameters to process reads into amplicon sequence variants (ASV), except for the following modifications: trimRight(5,40), minLen(150,150), (18S V4), trimRight(5,10), minLen(150,150) (rbcL) and setting maxEE to c(4,6) for both datasets. After computing error rates, dereplication and sample inference with default DADA2 settings, we removed samples that had one or fewer identifiable unique sequences from the analysis, merged forward and reverse read pairs, denoised the data by removing ASVs that only appeared in a single sample at less than 0.001 relative abundance and then removed chimeric ASVs using the “removeBimeraDenovo” function with the “pooled” setting from DADA2.

For the 18S data, we performed taxonomic assignment in R using DECIPHER’s IDTAXA function (Wright 2019), with PR2 5.0 (Guillou et al. 2013) and SILVA v.138 (Quast et al. 2012) reference databases, using IDTAXA's default threshold for identification (50). For rbcL data, we used BLASTn to query a local copy of the NT database (dated 01-11-2023) from the National Center for Biotechnology Information (NCBI; Bethesda, Maryland, USA), retaining the top 10 hits for each ASV at 96% identity and 50% query coverage. We used Galaxy Tool LCA (Beentjes et al. 2019) to determine a consensus lowest common ancestor (LCA) taxonomy from the BLAST hits for each ASV. We then integrated sequence tables, taxonomy tables and metadata into phyloseq objects (phyloseq v.1.40.0; McLaren (2020)) and filtered as follows:

- removed off-target ASVs (18S, rbcL: bacteria, macroalgae, vascular plants, *Zostera* spp., unassigned);
- removed samples with < 1,000 reads and ASVs that represented < 0.001% of total reads;
- converted ASV counts ≤ 9 to zero.

Following taxonomic classification, we manually curated the resulting taxon tables by re-evaluating ambiguous or low-confidence assignments using BLAST against the National Center for Biotechnology Information nucleotide database, ensuring consistency with reference taxonomy. Curated taxon tables are provided as supplementary materials (Suppl. materials 3, 4, 5, 6, 7).

### Data synthesis

We used R (v.4.5.0; R Core Team (2025)) to process and harmonise diatom occurrence records from: a) the literature review; b) the Global Biodiversity Information Facility (GBIF) and c) the morphological and molecular datasets generated through our sampling. We then filtered records to retain only diatom records that were identified to genus, species or infraspecific level, excluding higher-rank identifications, taxa sampled from freshwater bodies and any questionable reports. As diatom identification often requires detailed morphological or molecular analysis, we excluded most observations from iNaturalist (iNaturalist 2025) and kept only a subset that we verified directly from our sampling.

We aligned species names with taxonomic standards maintained by AlgaeBase (Guiry and Guiry 2025) and standardised all metadata to Darwin Core (DwC) standards. As the historical record is often insufficiently resolved to determine precisely which infrataxa are encompassed within reported Latin binomials, we retained species-level names alongside infrataxa as warranted. Morphological sources included museum collections, data from past regional monitoring (Del Bel Belluz et al. 2024, Esenkulova and Salinas-Ruiz 2024), as well as vouchers and micrographs that were generated through our sampling. Molecular data included sequences from the European Bioinformatics Institute (EBI; Cambridgeshire, UK) that were aggregated on GBIF (MGnify 2021), along with amplicon and Sanger datasets that were generated through our sampling (Table 2).

We concatenated annotations at the species level to build a comprehensive matrix that integrated all source data and identified new records from our sampling, based on historical precedence. Finally, we added fields for synonymy and critical notes, based on our review of the data. The matrix generated from this anlysis was then used to create the annotated checklist available as supplementary materials (Suppl. materials 1, 2). All novel data generated through this study are available as a DwC dataset on the Global Biodiversity Information Facility (Simon et al. 2026); the complete DwC dataset that we used to generate the checklist (synthesising all source data) is also available as supplementary materials (Suppl. materials 8, 9).

In determining new records, we compared the results of our sampling against both the historical record and detections arising from recent molecular surveys. As uncertainties can arise throughout the metabarcoding workflow (Mathieu et al. 2020), we regard detections, based solely on amplicon data, as provisional until they are confirmed morphologically. Thus, we treated as “new” any taxa that were previously unreported in literature or were unrepresented in collection databases, as well as records we verified morphologically that were previously known only from environmental amplicon sequencing. New records are presented as taxon treatments below.

## Taxon treatments

### Achnanthes groenlandica meridiana

Giffen

D111173B-3F83-59AE-8262-62765F0C15C6

**Figure 3.**
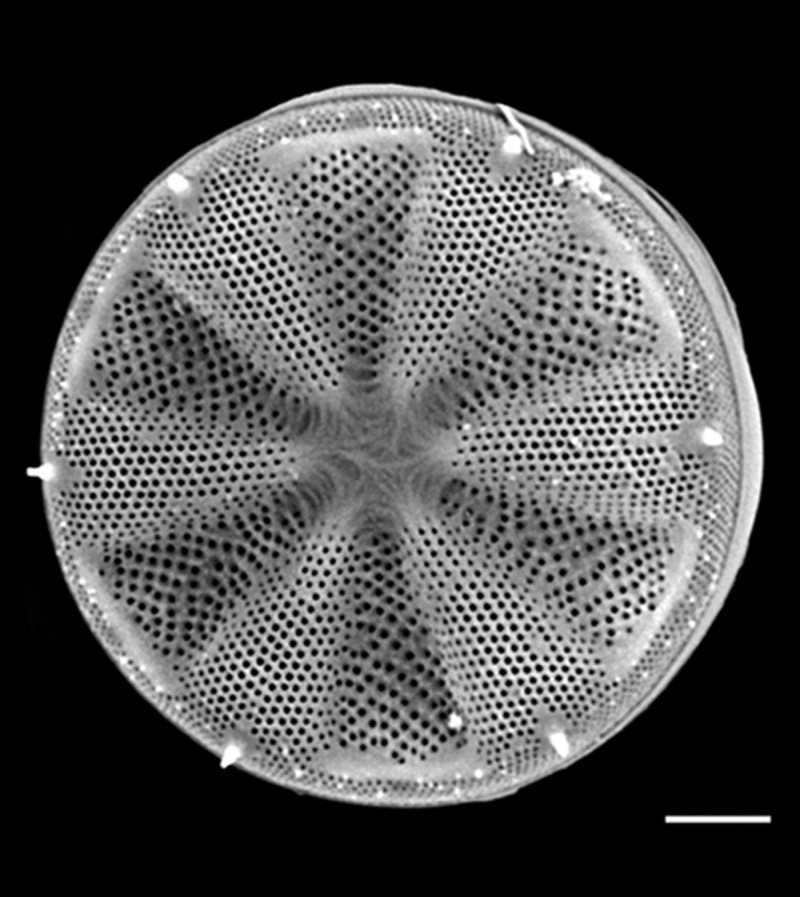
Actinoptychus adriaticus var. pumila, exterior valve view. SEM. Scale bar: 5 µm.

Achnanthes groenlandica var. meridiana Giffen (1973): 33, figs. 1–4, 619, 620, 621, 625—McIntire and Reimer (1974): pl. II, figs. 3a–c, pl. III; Tynni (1986): 14–15, table 1, pl. 15, fig. 91; Majewska et al. (2017): table 1; Kaleli et al. (2023): 43, pls. 5, 15–16.
Achnanthes groenlandica var. phinneyi McIntire and Reimer (1974)—Guiry and Guiry (2025).

#### Description

In girdle view, valve slightly geniculate, with convex RLV and concave RV. Valves linear-lanceolate with broadly rounded apices, slightly convex in the middle, length 22.9–32 (13.9–46) µm, width 4–5.0 (4–7) μm. RLV: narrow linear sternum, approximately central on valve face. Striae broken into one or two large and transversely elongate areolae. Central areolae occasionally larger than other areolae. Transapical rows of areolae 7–8 (6–8) in 10 µm. RV: moderately broad linear axial sternum. Central area has transverse fascia extending and slightly widening to the margins. Raphe filiform, narrow, broadening moderately near the slightly enlarged, slightly deflected proximal raphe ends. Distal raphe ends curved in the same direction, forming a shallow hook that terminates around the middle of the apices. Striae on both sides of sternum alternate, composed of two large, elliptical, usually transapically elongated areolae: one on valve face, one on mantle. The four or five striae nearest apices are moderately radiate, have smaller areolae and are more closely spaced than other striae on valve. Transapical rows of areolae 9–10 (8–13) in 10 µm on RV. Puncta on girdle bands next to RV 13–14 (10–12) in 10 µm.

#### Diagnosis

The lower striae density, more closely spaced areolae near apices, smaller width range, lack of terminal orbiculi and other characteristics of the Galiano Island specimens place them outside of measurements for *Achnanthes pseudogroenlandica* Hendey (Hendey (1964): 177, pl. 28, figs. 9–12; Witkowski et al. (2000): 94, pl. 44, figs. 16–23; Suzuki et al. (2009); Majewska et al. (2017): table 1) and *Achnanthes groenlandica* (Cleve) Grunow, which have a lower striae density range (Witkowski et al. (2000): 89, 90, pl. 44, figs. 1–7). McIntire and Reimer (1974) distinguished Achnanthes groenlandica var. meridiana (p. 172, pl. 2, figs. 5a and b) from Achnanthes groenlandica var. phinneyi f. jadyei, where the latter has a RLV sternum that is centred within the valve and has more increased striae than the var. phinneyi. Our samples contain both forms, however. Since AlgaeBase (Guiry and Guiry 2025) does not recognise Achnanthes groenlandica var. phinneyi f. jadyei, we have placed both forms within Achnanthes groenlandica var. meridiana.

#### Notes

Although this is the first report of an extant Achnanthes groenlandica var. meridiana within the Salish Sea, Tynni (1986) recorded this taxon as a rare occurrence in the Shine peat deposit (> 5.3 million years old) at a depth of 5.2 m at Squamish Harbor, Washington. The taxon was also identified by McIntire and Reimer (1974) in the Yaquina Estuary, Oregon. iNaturalist ID: 323108312, 323262404 (Fig. 2).

### Actinoptychus adriaticus pumila

Grunow

D0A460E3-D0EC-5F6C-8BE9-4B0C41119C48

Actinoptychus adriaticus var. pumila Grunow 1883: pl. 121, fig. 3, in Van Heurck (1882)—Lee and Chang (1996).

#### Description

Valves disc-shaped, diameter 13.5 (13.5–30) µm. Sectors 12, alternately raised and depressed. Externally, valve centre *hyaline* with curved wrinkles forming star-like pattern between sectors. Rimopotulae have medium tube length, are thin and hollow and face away from frustule centre, with slightly bulbous proximal connections to margin on the raised sectors. External rimopotulae located between valve face and valve mantle is open and round to elliptical in shape. Small *hyaline* area around base of rimopotulae. Hyaline rim on margin of depressed sector. Depressed sectors have a small and faint *hyaline* area in the margin. Areolae in raised sectors 22 (22) in 10 µm, in depressed sectors 14 (14) in 10 µm. Small siliceous nodules scattered on margin surface. Internally, rimopotulae located in the centre of raised sectors.

#### Notes

iNaturalist ID: 185626241 (Fig. 3).

### Andrzeja fenestrata

Mayama & Kryk

4A2CB6EB-B580-5219-B28B-4F3A378DBB19

Andrzeja fenestrata Mayama et al. (2025): 15, 17, table 2, figs. 44–59.

#### Description

Frustules rectangular, box-like in girdle view. Valve narrowly hemi-lanceolate with acute ends slightly inflated on dorsal side and flat on ventral side, length 8-15.4 (7–16) μm and width 1.5-2.2 (1.3–2.3) μm. Externally, valve face flat, curving roundly into the dorsal mantle. Ridge at juncture between valve face and mantle. External raphe branches short, arcuate and almost fully on the ventral mantle, with a small distal section along the valve face. Raphe proximal ends form drop-shaped holes within slightly curved elevations and distal ends are simple and curved away from marginal ridge. Internally, raphe fissures slightly curved (straight) and located along the face/mantle juncture, proximal ending simple, co-axially symmetrical, with low profile helictoglossa at the distal ends. Areolae elongate, parallel, uniseriate, occluded by hymens and 14-20 in 10 µm on valve face and dorsal mantle. Striae on ventral mantle, 30-35 (28–30) in 10 μm, are denser than on valve face. Striae elongated below raphe branches, 2-4 and generally 4 (4-5), have oval-shaped areolae between proximal raphe endings, with narrow slits near apices. Epicingulum has 4-5 (4-5) bands, each with a single row of pores.

#### Notes

In both the Gulf Island specimens of *Andrzeja fenestrata* and in figs. 44-59 in Mayama et al. (2025), the distal raphe often travels a short distance across the ridge on the valve face-mantle juncture into the valve face, ending in a helictoglossa. Specimens of epiphytic and epipsammic *A. fenestrata* from Madagascar and Mozambique, as shown in Mayama et al. (2025), have a distal raphe that often travels a short distance across the ridge on the valve face-mantle juncture into the valve face, ending in a helictoglossa. These morphometric data closely match the epiphytic specimens on the eelgrass *Zostera marina* from the Southern Gulf Islands, BC, Canada. An abundance of *A. fenestrata* colonies were found on the eelgrass *Zostera marina* at Montague Harbour Marine Provincial Park (MHMPP), Galiano Island, BC. Canada, in 2020 to 2022 collections. *Andrzeja fenestrata* has also been observed 30 km from MHMPP on *Z. marina* from Sidney Beach, Sidney, BC. 12 September 2025. iNat: 331457188, 331455851 (Fig. 4).

### Attheya longicornis

R.M.Crawford & C.Gardner

56B09C57-D0D6-561E-8247-F41128B73EE1

Chaetoceros septentrionale Østrup (1895): 457, pl. 7, fig. 88.
Attheya septentrionalis Østrup (1895)—Crawford et al. (1994): 41, figs. 42–49.
Attheya longicornis Crawford et al. (1994): 38, figs. 36–41—Hendey (1964); Hasle and Syvertsen (1997): 225; Bérard-Therriault et al. (1999): 38, pl. 22c–h; Orlova et al. (2002); Stonik et al. (2006); Rampen et al. (2009); Sugie and Kuma (2017); Stonik and Efimova (2020).

#### Description

Solitary marine species, not known to form colonies, except in culture. Valves flat to noticeably concave and lacking spines, spinules and rimoportulae. Length 5.2–6.4 (4–12) µm, 4–7.5 (3–16) µm in the apical axis (wide); pervalvar axis in culture often reported as 2–5 (2–5) times the width. Horns built of many silicious hoops and vary from long, thin, wavy, curly and straight, with horn length averaging 4.3–11 (4–10) times the cell length. Horns initially parallel to pervalvar axis, then may go in different directions. Longitudinal non-spiralling support bands 3 (3–4) in horns. Horns end in spines around an open apical aperture. Plastids per cell 1–2 (1–2). No resting cells or spores have been reported.

#### Diagnosis

Horns on *A. septentrionalis* are three times the valve length (Crawford et al. 1994; Hasle and Syvertsen 1997; Bérard-Therriault et al. 1999; Orlova et al. 2002; Stonik and Aizdaicher 2016) and are distinctly shorter than our specimens of *A. longicornis*, whose horns are 8–10 times the cell length. *Attheya longicornis* horns are strongly undulate (at least for one valve), as they arise from the valve corners and project parallel to the valvar plane, then often go in different directions. The two species of *Attheya* often differ in the longitudinal support bands in the horns: *A. septentrionalis* has four spiralling bands (Crawford et al. 1994; Stonik et al. 2006). Stonik and Aizdaicher (2016) report that from observations of six species, including *A. longicornis* and *A. septentrionalis*, found in the Sea of Japan, the only reliably different features that are useful for diagnosis between the two species are horn length and the number of longitudinal support rods in the horns. The horns of *A. longicornis* end in spines around an open apical aperture.

#### Notes

iNaturalist ID: 263094504 (Fig. 5).

### Bacteriastrum hyalinum

Lauder

595E350D-F58E-5842-9FC9-47F1EA0AEA41

Bacteriastrum hyalinum Lauder (1864): 8, pl. III, fig. 7—Cupp (1943): 96, figs. 56a–d; Hendey (1964); Hoppenrath et al. (2009): 73; Bosak et al. (2015); Barcode of Life Data System (2024); Guiry and Guiry (2025).
Bacteriastrum varians f. hyaline (Lauder) Frenguelli (1928): 543.

#### Description

Cells cylindrical, diameter 21.4–22 (14–54) μm. Chains are long, straight or slightly curved. Apertures narrow, but distinct. Inner setae 14–22 (12–46) on each valve, with short basal part, orientated almost perpendicular to the chain axis. Hairy appearance due to bifurcations in the pervalvar axis (parallel to chain axis). Forked parts gently curved and usually weakly twisted. Terminal setae not bifurcated and are umbrella-shaped, straight or undulate, stronger than inner setae, 2–3 (2) times longer than valve diameter and with spirally arranged tiny spines. Central process appears as a short, flattened tube on valve exterior and as a simple slit on interior side.

#### Notes

**Figure 6.**
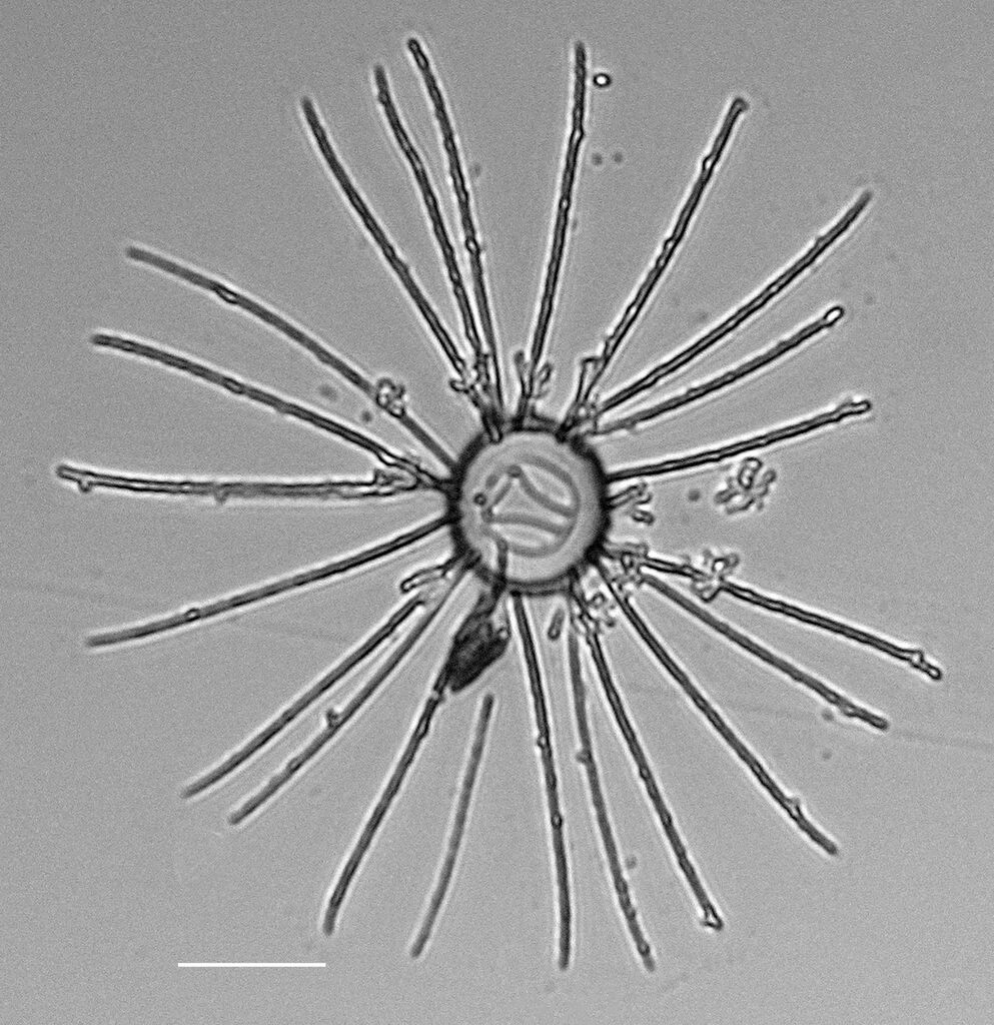
*Bacteriastrum hyalinium*, valve view. LM. Scale bar 20 µm.

iNaturalist ID: 315424735 (Fig. 6).

### Bacterosira constricta

(Gaarder) J.S.Park & J.H.Lee

E916773A-C6C2-51C0-914C-6A32C4A05897

Thalassiosira constricta Gaarder (1938): 6, fig. 6; Harris et al. (1995): 125, fig. 15; Hasle and Syvertsen (1997): 69–70; Throndsen et al. (2007): 137.
Bacterosira constricta —Park et al. (2016a).

#### Description

Frustules rectangular in girdle view, with slightly round valve edge. Cells in chains, often connected by chitin threads extruded from central fultoportulae; rarely solitary. Pervalvar axis length 17.8 (13.5–23.0) μm, sometimes exceeding length of valve diameter. Cells circular in valve view, diameter 21.9–22.7 (9.8–25.7) μm, with slight depression in centre. Clusters of 2 (0–14) fultoportulae present in valve centrs. Fultoportulae, with raised tubes, located on valve margin, 3–4 (5–7) in 10 µm. Single rimoportulae located mid-way between marginal fultoportulae. Areolar density, 36–44 per 10 μm on valve face, 37–46 per 10 μm on valve margin and 30 (41–51) per 10 μm on valve mantle.

#### Diagnosis

This specimen from Trincomali Channel has two central, buttressed, strutted processes instead of a cluster of tubes (fultoportulae) in the centre of the valve, which is unusual. Normally, for the two species of this genus, the buttresses are not apparent, though they are mentioned in Harris et al. (1995). All other morphological features of this specimen conform reasonably well with published accounts of *Bacterosira constricta*. This specimen is unlike the only other accepted species, *Bacterosira bathyomphala* (Cleve) Syvertsen & Hasle (Guiry and Guiry 2025). More specimens are required to examine the internal valve and girdle band morphology.

#### Notes

iNaturalist ID: 259444260. Mol. data: ERS27217975, ERS27218291, ERS27218292, ERS27218293, ERS27218294, ERS27218295, ERS27218296, ERS27218297, ERS27218298 (Fig. 7).

### Campylodiscus bicostatus

W.Smith ex Roper

7008E86F-E827-51DA-82EB-11B4D3BC1A38

Campylodiscus bicostatus W.Smith ex Roper (1856): 75, pl. 6, fig. 4 (as ‘*bi-costatus*’)—Sims (1996); Al-Handal et al. (2018): 143, fig. 103; Plinski and Witkowski (2020): 133, fig. 706.
Campylodiscus clypeus var. bicostata (W.Smith ex Roper) Hustedt (1930a): 448, fig. 874.

#### Description

Valve subcircular, convex along apical plane, diameter 57.5 (18–85) µm. Frustule saddle-shaped with round corners. Hyaline band surrounds the oblong central area (depressed elliptical central area). Distinctive radiate heavy costae, 2.5–3 (2.5–3) in 10 µm, do not reach centre of valve. Axial area linear-lanceolate, narrow or slightly expanded. Inner transapical striae 8–9 (9–10) in 10 µm.

#### Notes

**Figure 8.**
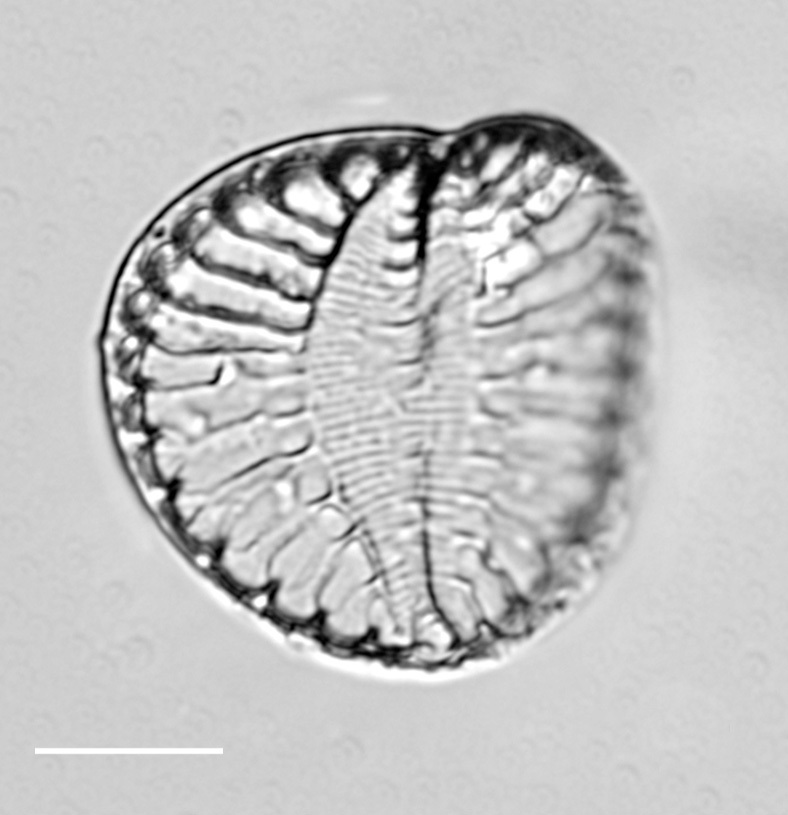
*Campylodiscus bicostatus*, exterior valve view. LM. Scale bar: 20 µm.

iNaturalist ID: 189680990 (Fig. 8).

### Cocconeis costata hexagona

Grunow

7ECAF15E-7228-5D64-A47B-AE840502A22D

Cocconeis costata var. hexagona Grunow 1880: pl. XXX, figs. 15–17, in Van Heurck (1880): pls. I–XXX—Romero and Rivera (1996): 327, figs. 17–34; Riaux-Gobin and Romero (2003): 24, 76, pl. 3; Al-Handal et al. (2022): 420, 425, fig. 50.

#### Description

Valves hexagonal in outline, with acute to rounded apices, length 14.5–23.1 (9–30) µm, width 8.9–11.9 (5.5–15) µm. SV: sternum relatively broad (narrow), linear, rarely linear-lanceolate. Transapical striae bi- to triseriate (bi- to tetraseriate), parallel, radiate at apices, 6–7 (6–13) in 10 µm. RV: similar to the nominate variety, transapical striae biseriate, parallel in middle becoming radiate towards apices, 18 (6–11) in 10 µm, 40 areolae (30–35) in 10 µm.

#### Notes

iNaturalist ID: 259362490; 259359045 (Fig. 9).

### Cocconeis kerguelensis

P.Petit

9C055198-AE70-535D-8E24-A5ED019348A2

Cocconeis kerguelensis Petit (1889): 116, pl. X, fig. 5—Cleve (1895): 182; Bourrelly and Manguin (1950); Desikachary (1988): 7, pl. 506, figs. 1–12.

#### Description

Valves elliptical, disc-like, length 53.1 (32–55) µm, width 47.9 (38–85) µm. SV: axial area relatively narrow, linear-lanceolate. Striae biseriate, 7 (4–6) in (10) µm, 4 striae at margins, slightly parallel at centre and highly radiate at apices. Areolae 16 (10) in 10 µm. RV (not imaged): raphe ends near margin. Axial area very narrow. Central area has transverse, outwardly narrowed fascia. Striae biseriate, 4 in 10 µm. Areolae 10–12 in 10 µm and crossed near margin by narrow blank band.

#### Diagnosis

The description above is based on Cleve (1895) and matches well with the images in Desikachary (1988). Only one image of the sternum valve.

#### Notes

*Cocconeis kerguelensis* (P.Petit) Cleve 1895 (as Cocconeis costata var. kerguelensis (P.Petit) Cleve 1895 was reported from the Yaquina Estuary, Oregon, USA by Riznyk (1973: 119, Pl. 5, Figs. 3 and 4). iNaturalist ID: 259379467 (Fig. 10).

### Cocconeis notata

P.Petit

DC1735B2-37F0-50EE-8E31-3F0B7705CE0F

Cocconeis notata Petit (1877): 168, pl. 4, fig. 1—Poulin et al. (1984): 54, figs. 22–25; Siqueiros-Beltrones and Ibarra Obando (1985); De Stefano and Marino (2001): 297, figs. 1–14, 44–46; Sar et al. (2003): 91, fig. 32; Lee et al. (2012): 250, fig. 1.

#### Description

SV: valves broadly elliptical, length 21.2 (12–29) µm, width 12.9 (6.4–19) µm. Sternum narrow and sigmoid with thin, stauros-like area expanding into a hemispheric *hyaline* area close to valve margin. Striae uniseriate, 10 (9–23) in 10 μm, parallel at the centre and curved to radiate at apices. Areolae round, 10 (10–22) in 10 μm. RV: valves broadly elliptical, length 23.7–26.9 (12–30) μm, width 15.9–16.2 (5.6–19) μm. A complete fascia reaches valve margin on one side and expands into stirrup-shaped *hyaline* area. Raphe narrow, sigmoid and slightly deflected in opposite directions. Central terminal endings slightly expanded. Transapical striae uniseriate, 16–18 (14–25) in 10 μm, parallel at centre and curved to radiate at apices. Areolae 15–16 (17–24) in 10 µm.

#### Notes

For the Eastern Pacific, *C. notata* was previously reported by Siqueiros-Beltrones and Ibarra Obando (1985) for the Baja California Peninsula on the Mexican Pacific coast. iNaturalist ID: 259450952 (Fig. 11).

### Cocconeis pseudomarginata intermedia

Grunow

35C2BD64-1B0D-56CF-A727-4D11556E3B4B

Cocconeis pseudomarginata var. intermedia Grunow (1867): 13, pl. 1, fig. 6—Joh (2021): 166, figs. 73–76, 88, 89.

#### Description

Valves elliptical, length 73.2 (26–68) μm, width 60.1 (14–51) μm. SV (note: no SV specimen to date): sternum broadly lanceolate with ends enlarged and pear-shaped, connected to submarginal *hyaline* area, with longitudinal line bisecting striae between sternum and valve margins. Outer surface of sternum longitudinally depressed. Transapical striae radiate towards apices, 25–30 in 10 μm; striae composed of elongated areolae. RV: raphe weakly sigmoid, sternum very narrow with terminal ends expanded and with crescent-shaped *hyaline* area, resulting in raphe distant from apices. Longitudinal depression between raphe and valve margins. Central area small, rhombic. Transapical striae radiate towards apices, 23 (23–28) in 10 μm in inner parts of valve (Joh 2021).

#### Diagnosis

This taxon differs from the nominate variety, *Cocconeis pseudomarginata*, in that the raphe is slightly sigmoid and transapical striae of SV are dense (Joh 2021).

#### Notes

**Figure 12.**
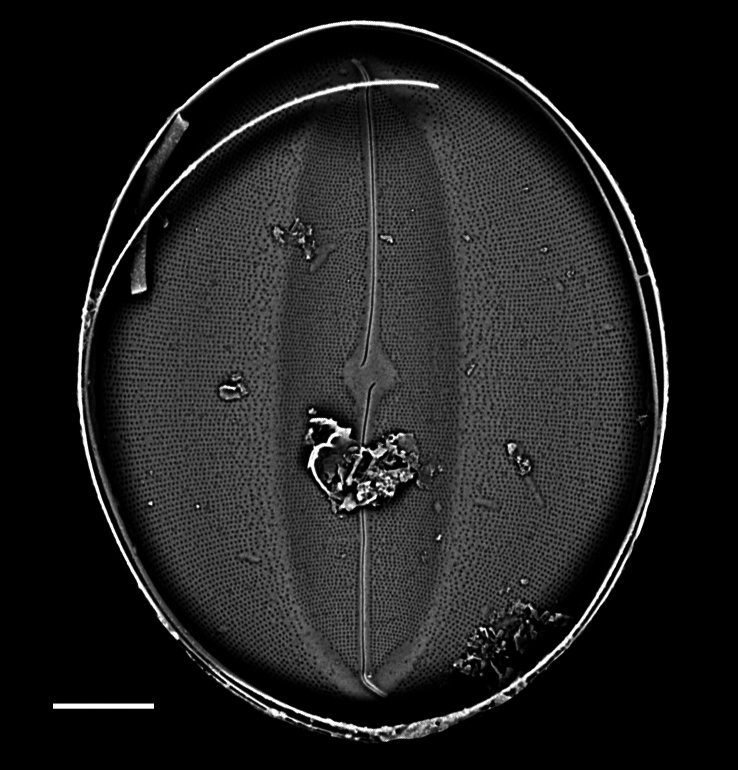
Cocconeis pseudomarginata var. intermedia, RV. SEM. Scale bar: 10 µm.

iNaturalist ID: 259455426 (Fig. 12).

### Cocconeis scutellum posidoniae

M.De Stefano, D.Marino & L.Mazzella

14E0B15F-D635-51FA-9DAE-5D7447F62A4C

Cocconeis scutellum var. posidoniae De Stefano et al. (2000): 235–237, figs. 72–86—De Stefano et al. (2008); Riaux-Gobin and Witkowski (2017); Riaux-Gobin et al. (2018).

#### Description

Valves elliptic-oval shaped, length 22.5–48.8 (13–40) µm, width of transapical axis 15.0–34.1 (8–23) µm. SV: Thickly silicified sternum lacks differentiated central area. Striae have radiate arrangement (11–14 in 10 µm), uniseriate in centre of valve to biseriate at margin and triseriate in mantle. Valve-face areolae, 9–13 in 10 µm arranged in apically aligned pattern. RV: Raphe straight and thin with coaxial proximal endings converging in very small central area and distal endings terminating in reduced, submarginal areas. Striae radiate, uniseriate from valve centre to submarginal *hyaline* area, which appears as a thickened rim to internal face of valve. Striae on mantle biseriate. Striae and areolae range from 12–17 (18–23) and from 18–22 in 10 µm, respectively. Papillae with parallel furrows on valvocopula (De Stefano et al. 2000).

#### Diagnosis

The specimens found on *Z. marina* conform well to the original description (De Stefano et al. 2000; De Stefano et al. 2008), especially the distinctive papillae on the valvocopula.

#### Notes

iNaturalist ID: 259478500 (Fig. 13).

### Cyclotella baltica

(Grunow) Håkansson

3672732D-C7BA-5E62-9128-DF39221C12C8

Cyclotella striata var. baltica Grunow 1882: pl. 92, figs. 13–15, in Van Heurck (1882)—Guiry and Guiry (2025).
Cyclotella baltica (Grunow)—Håkansson (2002): 104, 105, figs. 373–380; Hasle and Syvertsen (1997): 33, 34, table 1; Prasad and Nienow (2006); Tanaka (2007): 18, 19, pls. 11–13.

#### Description

Valve diameter 27.3 (11–67) µm with strong, tangentially undulate central area extending two-thirds (one-half to two-thirds) of the valve diameter. Central area colliculate with 9 (3–11) valve-face fultoportulae arranged in semicircular array on elevated (convex) area. Marginal striae 11–12 (8–16) in 10 µm. Internally, valve-face fultoportulae are surrounded by 3 (2–3) satellite pores. Externally, marginal area striae and interstriae are equal length and continue on to mantle. Mantle fultoportulae 7–8 (8–16) in 10 µm on non-recessed costae. At every second, occasionally third (second to fourth), interstriae are mantle fultoportulae, internally with two satellite pores orientated lengthwise to costae. Costae between mantle fultoportulae often slightly recessed. Internally, rimoportula located on mantle at same level as mantle fultoportulae, with opening running radially in same direction as costa.

#### Diagnosis

*Cyclotella baltica* and *C. litoralis* have similar morphologies. The latter species has marginal fultoportulae on recessed costae, often paired with a single costa of separation. In addition, the SHW specimen has a mantle fultoportula (MF) density of 7–8 in 10 µm, which falls within the published data for *C. baltica* and below densities and MF distribution for C. litoralis (MF 11–14 in 10 µm) and *C. striata* (MP 8–11 in 10 µm), but recessed on the second to fourth interstria, with absence of valve-face fultoportulae; *C. stylorum* (MF 9–12 in 10 µm, grouped in pairs or triplets); and *C. caspia* (MP 20–28 in 10 µm, every third or fourth interstria, diameter 3.5–22 µm).

#### Notes

Brackish to marine waters. iNaturalist ID: 265283095 (Fig. 14).

### Didymosphenia geminata

(Lyngbye) Mart.Schmidt

8D2F4614-09A5-5365-BEA9-3FD7ACE7AE66

Didymosphenia geminata —Schmidt (1899): pl. 214, figs. 7–10; Round et al. (1990): 496, 497; Snoeijs and Balashova (1998): pl. 430; Khan-Bureau et al. (2016): 7, figs. 17–24; Lange-Bertalot et al. (2017): 188, 189, pl. 102, fig. 1.

#### Description

Head pole distinct and subcapitate; foot pole rounded to slightly capitate, with distinct constriction of apices and inflated central part. Valve face flat. Length 124.5 (48–161) µm, width 43.6 (25–45) µm, length-to-width ratio 2.9 (1.9–3.9). Stigma number 4–5 (1–7) in a row to one side of central area. Striae uniseriate 10 (7–10) in 10 µm, radiate and becoming parallel to convergent towards poles and of different lengths in middle of valve, circling terminal fissure in head pole. Clear area in foot pole has very small pores where mucilage stalks are secreted. Areolae 12–13 (9–12) in 10 µm. Raphe laterally displaced, mostly straight, with expanded central ends and long, hooked terminal fissures in same direction.

#### Diagnosis

A distinctive species, especially the row of stigma to one side of the central area and inflation of the valves between the subcapitate poles.

#### Notes

**Figure 15.**
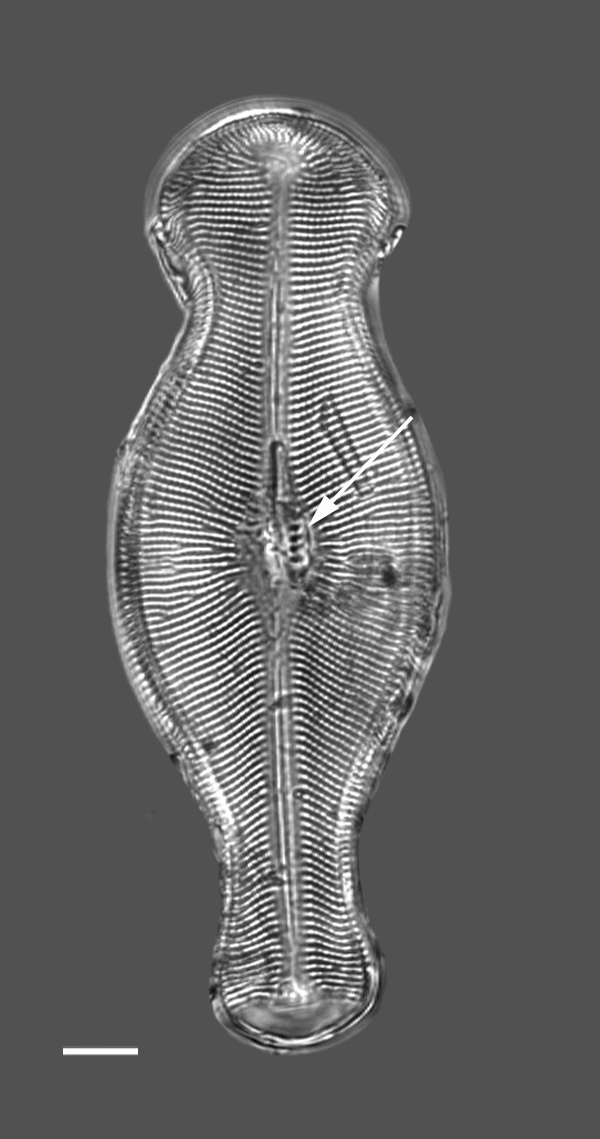
*Didymosphenia geminata*, valve view. Arrow indicates a row of five stigma. LM. Scale bar: 10 µm.

This is a freshwater epiphytic and epilithic species. Specimens were possibly washed into Galiano Island waters from the Fraser River outflow. iNaturalist ID: 264211715; 318331778 (Fig. 15).

### Diploneis exemta

(A.W.F.Schmidt) Cleve

1F3FD30D-C158-550C-B681-7A823DAA2E78

Navicula exemta Schmidt (1875): pl. 11, figs. 28, 29.
Diploneis exemta Cleve (1894): 86—Kuntze (1898): 548, 549, 552; Peragallo and Peragallo (1908): 112, pl. 15, figs. 16, 17; López-Fuerte and Siqueiros-Beltrones (2016): 242; Guiry and Guiry (2025).
Schizonema exemtum (A.W.F.Schmidt) Kuntze (1898): 549, 552—Guiry and Guiry (2025).

#### Description

Valve panduriform with tongue-shaped segments, length 85.0 (60–140) µm, width 21.8 (32–42) µm, 14.1 (16–30) µm at constriction. Central nodule quadrate, moderately large, slightly expanded in apical direction; horns parallel and furrows linear. Striae density 8 (5–6) in 10 µm (8 in 10 µm for D. exemta var. digrediens), crossed by 1 (1–2) longitudinal lines. Distal to furrow is a line of faint or large puncta, 8 in 10 µm.

#### Diagnosis

This specimen conforms fairly well to the description in Cleve (1894) and the drawings (figs. 28 and 29) in Schmidt (1875), including shape proportions and is a fairly exact match with the drawing in Peragallo and Peragallo (1908). Exceptions include a slightly higher striae density in Cleve’s and Peragallo and Peragallo’s descriptions (5–6 in 10 µm) and the row of large puncta that is interrupted in the middle of the valve, distal to the longitudinal furrows as in Peragallo and Peragallo’s drawing. Either this specimen is a new variety or it can be placed into the unverified (Guiry and Guiry 2025) variety of D. exemta var. digrediens Cleve (1894) with a reported striae density of 8 in 10 µm.

#### Notes

This species is listed as a freshwater species by AlgaeBase (Guiry and Guiry 2025) and has been reported by López-Fuerte and Siqueiros-Beltrones (2016) as epiphytic in the marine waters of Baja California and the Gulf of Mexico.

**Figure 16.**
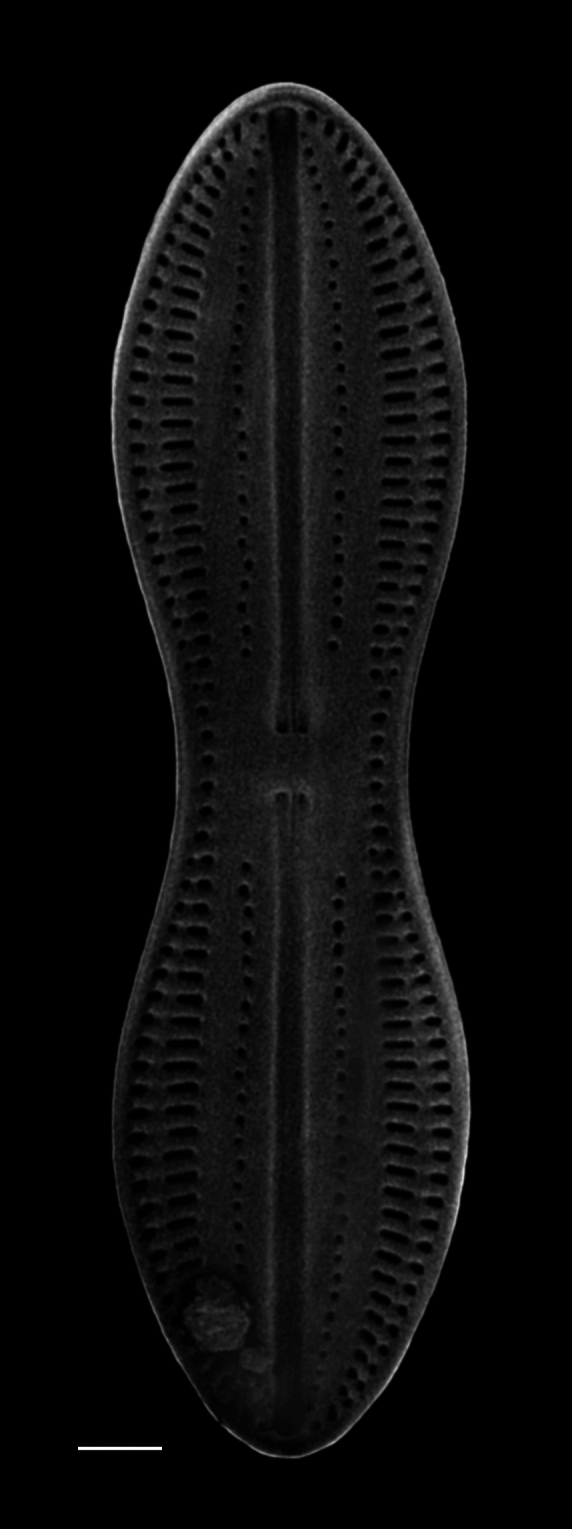
*Diploneis exemta*, interior valve view. SEM. Scale bar: 5 µm.

*Diploneis exemta* is newly reported for the Pacific coast of North America, with the exception of a fossil report from California. This species is listed by Cleve (1894) from marine waters of the east coast of Madagascar, Tahiti, Kerguelen Island and Campeche Bay in the Gulf of Mexico, with fossils from Oamaru, New Zealand and Santa Monica, California. Cleve reported Diploneis exemta cf. digrediens from China and a fossil specimen from Hungary. Peragallo and Peragallo (1908) identified *Diploneis exemta* from the English Channel and Banyuls-sur-Mer, Balearic Sea, France. iNaturalist ID: 266785298 (Fig. 16).

### Extubocellulus spinifer

(Hargreaves & Guillard) Hasle, Stosch & Syvertsen

62323E22-CF28-587A-940E-98A5461E25CB

Bellerochea spinifera Hargraves and Guillard (1974): 168, figs. 9–12.
Extubocellulus spinifer —Hasle et al. (1983): 70–73, figs. 362–390, 392; Saunders et al. (2010): 135, 136, fig. 3.14d; Dabek et al. (2019).

#### Description

Chain-forming. Valves broadly elliptical to circular, length 2.6–2.8 (1.6–3.6) µm, width 2.2–2.3 (1.5–3.0) µm, with shallow mantle. Rectangular in girdle view. Small, raised, short and funnel-like ocelluli located at each apex. Valve face perforated by approximately equidistant larger and unoccluded poroids 50 (50) in 10 μm of diameter 60 (78) nm and smaller and unoccluded poroids 60–70 in 10 µm of diameter 20 nm. Globules or short spines are scattered, or not, on valve face. Occasional tubular processes.

#### Diagnosis

No vela could be found in any of our specimens (n = 2), which is in accordance with accounts in North American and European literature (Hasle et al. 1983). However, Saunders et al. (2010) reported vela in the poroids (areolae) for their Australian specimens. Tubular processes were not observed in any of those samples.

#### Notes

**Figure 17.**
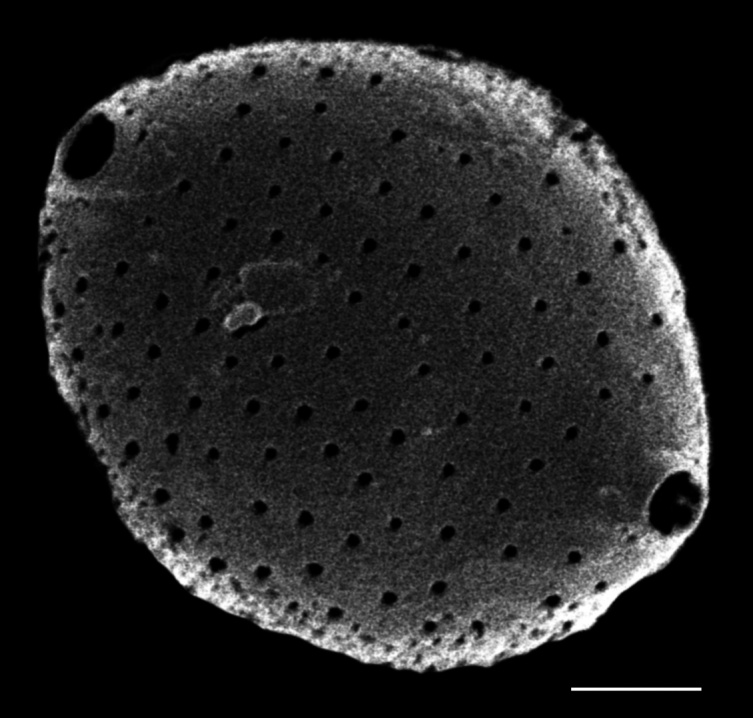
*Extubocellulus spinifer*, exterior valve view. SEM. Scale bar 0.5 µm.

iNaturalist ID: 311561542. Mol. data: ERS21395345, ERS27630062 (Fig. 17).

### Fogedia krammeri

Witkowski, Lange-Bertalot, Kociolek & M.Kulikovskiy

BD114DFA-999C-555B-8485-D2E410D02560

Fogedia krammeri Witkowski et al. (2010): 50, figs. 1, 2—Kim et al. (2022).

#### Description

Valves moderately variable in outline, from broad-elliptic or broadly elliptic-lanceolate to linear-elliptic, with ends cuneate and terminus shortly protracted and subrostrate. Length 23.5–28.2 (20–30) μm, width 11.7–12.0 (9.5–12.0) μm. Raphe slightly lateral, with two minor undulations at one-quarter distance from central endings and one-quarter distance from terminal endings. External central raphe endings expanded; terminal raphe endings short and slightly bent in same direction. Axial area narrow, linear throughout. Central area forming a shortened, almost rectangular fascia connected to lateral area, with transapical striae interrupted in marginal part on either side. Striae subparallel to slightly radiate proximally to the ends and becoming more strongly radiate distally, where they are very slightly denser at 11–12 (10–12) in 10 μm. Lineolae of the striae comparatively coarse, discernible by LM, 27–30 (26–29) in 10 μm.

#### Diagnosis

This specimen has similarities to both *Fogedia krammeri* and *F. finmarchica* (Cleve 1895: 28) (Van der Werff and Huls (1975): P.D G XVI. 109; Hendey (1964): 198, pl. 30, fig. 5; Riznyk (1973): 126, pl. 11, fig. 7; Sims (1996): pl. 141, fig. 12; Witkowski et al. (1997)). As noted in Witkowski et al. (2010), *F. finmarchica* has a more lanceolate outline and a higher lineolae density of 35–40 in 10 μm, which is very difficult to resolve by LM. For the MHMPP specimens observed with LM, the valve outline is more broadly elliptic than drawings in the literature for *F. finmarchica*; the lineolae can be resolved and are 27 in 10 µm, matching data for *F. krammeri* and the SEM image from the SHW specimen shows an areolae density of 30 in 10 µm. Additionally, the undulations in the raphe of the SHW specimen (SEM) match the undulations in the images of specimens from San Francisco Bay (Witkowski et al. 2010). Further, the expansion of the raphe endings are more pronounced in *F. krammeri*, matching our specimens. Lastly, most images of *F. finmarchica* show a pronounced lanceolate outline. From these criteria, we place our specimens in *F. krammeri*. This species was previously reported and first described in San Francisco Bay by Witkowski et al. (2010) and was, as far as can be ascertained before this report, the only known locality for *F. krammeri*.

#### Notes

iNaturalist ID: 312508075, 323689358 (Fig. 18).

### Gomphonemopsis pseudexigua

Medlin

9C00DB37-A094-506A-AAEF-4FA4E884EA5F

Gomphonemopsis pseudexigua Medlin and Round (1986): 212, figs. 16–18—Krzywda et al. (2019); Witkowski et al. (2000): 222, pl. 61, figs. 17, 18; Pienitz et al. (2003): 58, pl. 17, fig. 21.

#### Description

Valves linear to narrowly linear lanceolate, length 20.9–26.9 (6–40) µm, width 1.6–1.9 (1.5–5.0) µm. Striae 18–20 (11–22) in 10 µm, elongate to generally round at the poles, slightly parallel to slightly radiate at the apices. Narrow axial area, small rectangular or round central area that reaches the valve margin with a small stria lying in the mantle opposite either side of the central area.

#### Notes

The specimens from MHMPP fit well with the morphological summary of Krzywda et al. (2019). iNaturalist ID: 189577227. Mol. data: ERS27214058, ERS27214059, ERS27214060, ERS27214061, ERS27214062, ERS27214063, ERS27214064, ERS27214065, ERS27214066, ERS27214067, ERS27214068, ERS27214069, ERS27214070 (Fig. 19).

### Gomphoseptatum aestuarii

(Cleve) Medlin

77EAF7F6-6C63-5525-B827-43E8DB80752E

Gomphomena aestuarii Cleve (1893): 55, pl. 3, fig. 4.
Gomphoseptatum aestuarii —Medlin and Round (1986): 212, figs. 16–18; Witkowski et al. (2000): 222, pl. 61, figs. 17, 18; Pienitz et al. (2003): 58, pl. 17, fig. 21; Li et al. (2020).

#### Description

Valves linear with obtusely rounded apices, length 10.6–17.7 (9–35) µm, width 2.4–3.5 (2–4.5) µm. Raphe straight, external central endings expanded and relatively distant, axial area narrow. Distal raphe endings bent and slightly recurved in same direction at both apices. Central area transversely expanded into fascia that reaches valve margins. Distinctive pore field in foot pole. Well-developed pseudoseptum at foot pole. Transapical striae 16–20 (16–24) in 10 µm, slightly radiate to parallel at apices and punctate. Striae are crossed by longitudinal rib that runs midway between raphe and valve margin.

#### Notes

The morphology of *G. aestuarii* found on the red alga *S. americana* at Miners Bay Wharf conforms well to the original description and images in Medlin and Round (1986) for specimens from Oregon, USA and the morphometric data provided by Pienitz et al. (2003) for a specimen from Haida Gwaii, north of the Salish Sea. iNaturalist ID: 258910079 (Fig. 20).

### Gomphoseptatum pseudoseptatum

(Giffen) Witkowski, Lange-Bertalot & Metzeltin

6C3C5823-79AC-5C0D-AE8D-49037312CDAB

Gomphonema pseudoseptatum Giffen (1970): 13, figs. 29–32—Medlin and Round (1986): 476, 477.
Gomphoseptatum pseudoseptatum —Witkowski et al. (2000): 222, pl. 60, figs. 22–26; Guiry and Guiry (2025).

#### Description

Frustules in girdle view weakly clavate with distinctive pseudosepta on both apices. Valves linear to linear-lanceolate, gradually tapering from obtusely rounded head pole to slightly produced foot pole. Length 40.7–63.1 (12–33.3) μm, width 4.6–4.9 (2.5–5) μm. Raphe straight, axial area narrow. Central area has moderately wide transverse fascia that extend to margins. Externally, raphe is central, straight and opens slightly laterally. Central raphe endings slightly expanded; pore-like, polar (distal) fissures are bent, hook-like and curved to one side like a question mark, then curve back to centre of pole and are heavily silicified. Transapical striae uniseriate and mostly parallel, 8–9 (10–16) in 10 μm in mid-valve, 12 in 10 µm near poles. Striae crossed by rim of silica that runs around circumference of valve. Half of each stria subdivided into pores with cribra-like complex (30–80 nm pores), round to transapically slightly elongate. Striae towards each apex are reduced to smaller, single, round pores with the same cribra-like feature. Internally, pseudosepta are well-developed and equal-sized. Raphe internal endings straight. Pores closest to central area reduced in size and without marginal pore.

#### Diagnosis

Morphologically, the illustrations in Giffen (1970), an unpublished image by Giffen (image A1008Sa02: A. Witkowski pers. comm., 07-06-2023) and the description and images of *G. pseudoseptatum* in Witkowski et al. (2000) support placing the two specimens from Galiano Island into *Gomphoseptatum pseudoseptatum*.

#### Notes

iNaturalist ID: 258910629 (Fig. 21).

### Gyrosigma arcuatum

(Donkin) Sterrenburg

4D35C073-FFFE-5770-9634-4B0E1B39A7FF

Pleurosigma arcuatum Donkin (1858): 25, pl. 3, fig. 10.
Gyrosigma fasciola var. arcuatum (Donkin) Cleve (1894): 116 (as ‘*arcuata*’)—Proshkina-Lavrenko (1950): 249, pl. 82, fig. 7; Jin et al. (1985): 80, pl. 24, fig. 188; Cardinal et al. (1989): 18, fig. 7; Stidolph (1994); Sims (1996): pl. 113, fig. 4; Jahn et al. (2005): 309.
Gyrosigma arcuatum —Jahn et al. (2005): 308.

#### Description

Distinguished by long, rostrate apices curving in opposite directions and sharply turned out from valve body. Valve distinctly sigmoid, lanceolate to elliptical-lanceolate in central portion, narrowing to long and thin extensions that are bluntly rounded or flattened. Valve face fairly flat, length 100.9–105.0 (60–150) µm, width 10.6–14.3 (10–24) µm. Axial area and raphe central and sigmoid, appearing slightly eccentric in extensions of some valves. Central area quite small, orbicular or longitudinally elliptical. Central raphe endings slightly deflected to opposite sides. Longitudinal striae, 25–30 (27) in 10 µm and transverse striae, 24–30 in 10 µm, about equal and distinct. Two marginal, thin plastids, almost exactly opposite.

#### Notes

These three specimens of *Gyrosigma*, with long and narrow apical extensions of the valve and curved in opposite directions, are placed into *Gyrosigma arcuatum* (Donkin) Sterrenburg (Guiry and Guiry 2025), as recommended by Jahn et al. (2005). iNaturalist ID: 254241262, 255788398, 254242421 (Fig. 22).

### Homoeocladia spathulatoides

(Lobban, Ashworth, Calaor & E.C.Theriot) Lobban & Ashworth

E438FB89-5E9A-5B98-BD1F-3BAFFA9A37C3

Nitzschia spathulatoides Lobban et al. (2019): 227, figs. 121–129—Lobban and Ashworth (2022): 5.
Homoeocladia spathulatoides (Lobban, Ashworth, Calaor & E.C.Theriot)—Lobban and Ashworth (2022).

#### Description

Valves lanceolate, rostrate, length 45.7 (110–125) μm, width ca. 7–8 (ca. 11) μm, generally lying in girdle view. Striae 46–48 (45) in 10 μm, comprising tiny puncta, transapically 68 (66) in 10 μm. Keel straight, central, with distinct spathulate extensions at apices. Fibulae 2–4 in 10 μm. Several rows of pores on the keel apex. Copulae reported with numerous pores arranged in short transapical striae. Plastids apparently two plates against girdle face.

#### Diagnosis

This specimen is assigned to *Homoeocladia spathulatoides* as it is morphologically distinct from *H. spathulata* (Syn: *N. spathulata*): a) the MHMPP specimen has striae with very small discrete pores vs. the long slits found in *N. spathulata*; b) there are pores over the apex and c) the striae are slightly less dense than *N. spathulata* (Lobban et al. 2019: 226).

#### Notes

iNaturalist ID: 193640716 (Fig. 23).

### Isthmia enervis

Ehrenberg

EBF9706D-7FD9-5924-B2EC-866B4BC712F4

Isthmia enervis Ehrenberg (1838): 209, pl. 16, fig. 6—Kützing (1844); Smith (1856): pl. 47, fig. F; Van Heurck (1881): XCVI, figs. 1–3; Hendey (1964): 110, 111, pl. XXV, fig. 3, pl. XXV, fig. 2; Round (1984); Round et al. (1990); Sims (1996); Guiry and Guiry (2025).

#### Description

Cells form branching colonies with large mucilage pads. Cells rhomboid-shaped, heteropolar. Valves elliptical and ovoid, apical axis 70 (48–200) µm, length of whole frustule in girdle view 195–268 (150–300) µm. No transition between valve face and mantle. Single pseudocellus per cell, with cluster of smaller areolae. Externally, areolae are large, cribrate, suspended by struts, ovoid to quadrate and enclosed by robust silica. In the apical direction, every 2–3 areolae have a pore in the silica mesh framework; in the transapical direction, 1–2 areolae between poroids. Areolae pattern curved, slightly radiate transapically, 2–3 in 10 µm. Areolae size is mostly regular over valve face, becoming smaller at pseudocellus. Girdle bands complete, with smaller areolae than valve and closed by cribra.

#### Notes

iNaturalist ID: 204882487 (Fig. 24).

### Licmophora communis

(Heiberg) Grunow

A927DF5E-615F-580A-A010-2B15A10095AD

Podosphenia communis Heiberg (1863): 76, pl. 6, fig. 23—Guiry and Guiry (2025).
Licmophora communis —Grunow 1881: pl. 48, figs. 8, 9, in Van Heurck (1881); Proshkina-Lavrenko (1950); Van der Werff and Huls (1975): P.A.D. X b. 66; Honeywill (1998): 238, fig. 5a–i; Snoeijs (1993): 65, pl. 51; *Sims (1996)*: 260, pl. 122, figs. 3, 4; Witkowski et al. (2000): 63, pl. 20, fig. 1; Plinski and Witkowski (2020): 57, 246, fig. 221; Romagnoli et al. (2014).

#### Description

Cells in girdle view wedge-shaped with strongly rounded corners, septa mostly long, rarely fairly short. Valves clavate, head pole broadly rounded, 17.7 (20–60) µm long, 5 (6–15) µm broad. Sternum (very) distinct, transapical striae (robust), 25–30 (11–17) in 10 µm near foot pole to 30–35 (23–30) in 10 µm at head pole. Areolae 50 in 10 µm. Rimoportula at head pole with short stalk.

#### Diagnosis

For now, this single valve from Galiano Island will be assigned to Licmophora cf. communis until more specimens are imaged, even though this specimen fits well with the published data.

#### Notes

**Figure 25.**
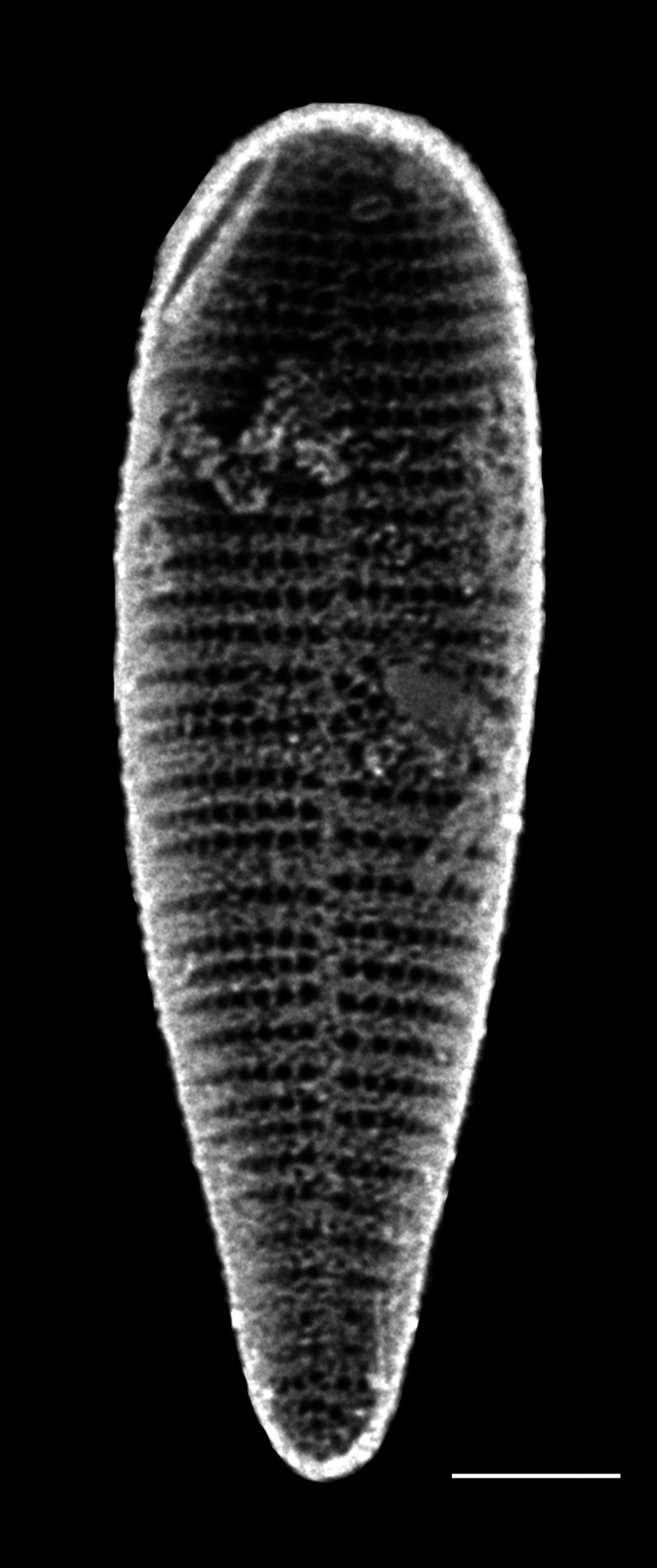
Licmophora cf. communis, interior valve view. SEM. Scale bar: 2 µm.

iNaturalist ID: 263351671 (Fig. 25).

### Licmophora tincta

(C.Agardh) Grunow

8D46A1B2-4A48-5442-90DE-3A07FCACBDB9

*Licmophora tincta Agardh (1828)*: 32 (no measurements given)—Grunow (1868): 35; Proshkina-Lavrenko (1950): 20, pl. 6, fig. 11a, b; Hendey (1964): 168; *Sims (1996)*: pl. 123, fig. 3; Honeywill (1998): 239.
Licmophora paradoxa var. tincta (Agardh) Hustedt (1931): 77, fig. 607—Guiry and Guiry (2025).

#### Description

Wedge-shaped cells in girdle view with wide round head pole. Valves clavate with broadly rounded poles; valve margin slightly concave near foot pole. Septa distinctly deep. Length 66.0–90.2 (30–140) µm, width 8.5–12.5 (7–18) µm, sternum distinct. Transapical striae fine, 29–34 (27–30) in 10 µm at head pole, 26–30 (23–32) in 10 µm at foot pole, 26–29 striae in 10 µm in mid-valve. Transapical areolae 50 in 10 µm, elongate near sternum (elongate to round). Two rimoportulae per cell (Honeywill 1998). Basal rimoportula has very short stalk and wide internal lips. Multiscissura has 14–15 (10) slits.

#### Diagnosis

Due to the high striae count at both poles, we assigned this specimen to *Licmophora tincta* (C.Agardh) Grunow, syn. Licmophora paradoxa var. tincta (Agardh) Hustedt. *Licmophora tincta* is very similar to *L. paradoxa*; however, this specimen has more striae at 33 in 10 µm (Proshkina-Lavrenko (1950): 20, pl. 6, fig. 11a, b).

#### Notes

*Licmorphora paradoxa* (Lyngbye) C. Agardh was previously reported by Shim (1976) in the Strait of Georgia, Salish Sea. iNaturalist ID: 195808793 (Fig. 26).

### Minidiscus proschkinae

(Makarova) J.S.Park & J.H.Lee

BBA59E05-CB2B-57CB-B4EC-F97D36C7A0E6

Thalassiosira proschkinae Makarova et al. (1979): 922, pl. 1, figs. 1–7—Snoeijs (1993): 113, pl. 99; Hasle and Syvertsen (1997): 84, table 14, pl. 13; Hoppenrath et al. (2007): 59, fig. 24e.
Minidiscus proschkinae —Park et al. (2017); Li et al. (2020); Guiry and Guiry (2025).

#### Description

Forms chains of 2–3 cells, linked by mucilage thread extruded from central fultoportula. Cells circular with flattened valve face or slightly convex, with sharp-angled mantle. Diameter 11.7 (3–11.5) µm. Single rimoportula located on valve surface adjacent to central fultoportula, both without external tubes and with a single areola in between. Areolae in tangential rows, 30–35 (25–30) in 10 μm, at valve centre. Marginal fultoportula processes (MFPs), without external tubes, 6 (6–8) closely spaced, 5 (1.24–1.91 µm) separation, processes 2.75 in 10 µm.

#### Diagnosis

The external rimoportula and central fultoportula, both without external tubes and close together, the MFPs without external tubes and the unique ornamentation of the valve surface place this specimen in *Minidiscus proschkinae*. Our molecular data from Trincomali Channel and plankton from Miners Bay Wharf, Mayne Island, support the presence of *Minidiscus proschkinae*.

#### Notes

**Figure 27.**
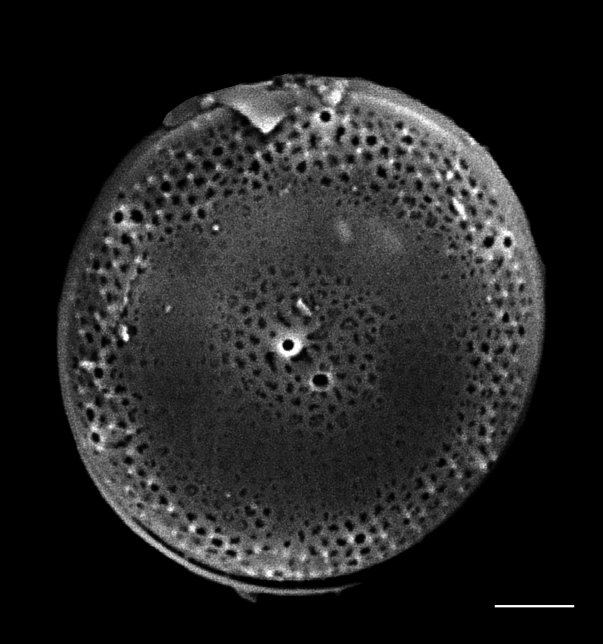
*Minidiscus proschkinae*, exterior valve view. SEM. Scale bar: 2 µm.

Previously identified in Haida Gwaii, BC, by Pienitz et al. (2003). iNaturalist ID: 260112129 (Fig. 27).

### Neocalyptrella robusta

(G.Norman ex Ralfs) Hernández-Becerril & Meave

942E60E9-FFCF-5770-8035-0942305C6A7F

Rhizosolenia robusta Ralfs 1861: 866, pl. VIII, fig. 42, in Pritchard (1861)—Cupp (1943).
Neocalyptrella robusta —Hernández-Becerril and Meave del Castillo (1996); Hernández-Becerril and Maeve del Castillo (1997): 329; Hasle and Syvertsen (1997); Sunesen and Sar (2007); Hoppenrath et al. (2009); Yun and Lee (2011); Boonprakob et al. (2021); Guiry and Guiry (2025).

#### Description

Cells in valvar cross section elliptical; cells in girdle view sigmoid, curved and crescent-shaped. Length 227–240 (in culture) to 477 (500–1000) µm, diameter 59 (109, in culture) (40–400) µm. Valves noticeably conical, with longitudinal undulations converging near truncated apex. Valve apex has calyptra, which bears a needle-like external tube or process that is a complex rimportula. Valve areolae 13–20 in 10 µm arranged in regular, straight striations and with secondary quincunx pattern of 22–25 in 10 µm.

#### Notes

The morphology of these LM images agrees with literature, especially the images and description in Sunesen and Sar (2007). Only observed in December. Rare in Galiano Island waters. iNaturalist ID: 48460936 (Fig. 28).

### Parlibellus delognei ellipticus

(Lobban) E.J.Cox

EB395CF7-7288-5F04-85D8-755F8C98DFF3

Parlibellus delognei f. ellipticus (Lobban)—Cox (1988): 21.

#### Description

Parlibellus delognei f. ellipticus (Lobban) E.J.Cox is more elliptical-rhomboid-shaped and lacks occasional puncta (carinoportula) in central area compared to the typical form. Length 45.4–62.8 (42–50) µm, width 12.7–19.4 (13–17) µm, striae 20–23 (19–20) in 10 µm, areolae 24 in 10 µm.

#### Notes

Naturalist ID: 256411671. Mol. data: ERS27214058, ERS27214059, ERS27214060, ERS27214061, ERS27214062, ERS27214063, ERS27214064, ERS27214065, ERS27214066, ERS27214067, ERS27214068, ERS27214069, ERS27214070, ERS21395346, ERS21395352, ERS21395359, ERS21395365, ERS21395369, ERS27630064, ERS21395356, ERS27217975, ERS27218291, ERS27218292, ERS27218293, ERS27218294, ERS27218295, ERS27218296, ERS27218297, ERS27218298 (Fig. 29).

### Proschkinia complanatula

(Hustedt ex Simonsen) D.G.Mann

82326123-659F-549E-BF44-AEC288820616

Amphora complanata Grunow 1867: 25—Van der Werff and Huls (1975): P.D G XVI. 109; Guiry and Guiry (2025).
Navicula complanatula Hustedt ex Simonsen (1987): 479, pl. 735, figs. 1–10.
Proschkinia complanatula —Mann 1990: 675, in Round et al. (1990): 596, 597; Majewska et al. (2019); Kim et al. (2020): 10–12, fig. 4; Plinski and Witkowski (2020): 115, 324, fig. 576.

#### Description

Cells solitary, usually positioned in girdle view, rectangular with rounded corners and with numerous, moderately broad bands, 9 (7–8) in 10 µm. Valves lanceolate, slightly arched, with acutely rounded apices, length 64.7 (30–58) µm, width 7.4 (5.5–8.0) µm. Raphe straight and linear with proximal ends close and distal ends strongly hooked. Transapical uniseriate striae parallel at centre, 18 (12–17) in 10 µm, becoming slightly convergent and denser near apices, 22 (17–20) in 10 µm. Central area asymmetric with striae more distant than towards the apices, producing a stauros-like structure. Fistula located in central area on primary side of valve.

#### Notes

iNaturalist ID: 188679253 (Fig. 30).

### Seminavis robusta

D.B.Danielidis & D.G.Mann

B0DE6EEF-8E6B-56A0-B43E-F26A3785EC95

Seminavis robusta Danielidis and Mann (2002): 440, figs. 39–53—Wachnicka and Gaiser (2007); Kaleli et al. (2023).

#### Description

Valves strongly dorsiventral, frustules semi-lanceolate to rhombic-lanceolate, ends more or less obtusely rounded. Length 61.5 (34–104) µm, width 11.5 (6.5–13.5) µm. Margins convex dorsal with straight to slightly convex ventral margins. On dorsal side, axial area more expanded and rounded than on ventral side. Longitudinal raphe straight and displaced to ventral side. Externally, central raphe branches are slightly deflected ventrally and proximal raphe ends are deflected ventrally at apices. Dorsal striae slightly radiate in middle, 14 (12–20.7) in 10 µm and slightly denser 18 (15–18) in 10 µm at apices. Ventral striae at centre 14 (11–15) in 10 µm and at ends 17 (12–18). Middle striae are shorter than the others. Solitary cells.

#### Notes

**Figure 31.**
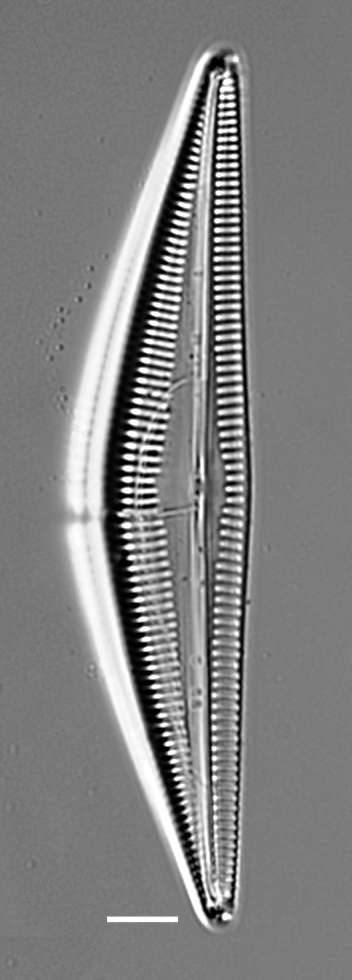
*Seminavis robusta*, LM, valve view. LM. Scale bar: 5 µm.

This specimen from Retreat Cove conforms well with descriptions and data in literature. However, this specimen differs slightly from the description of Danielidis and Mann (2002) by having slightly radiate striae at the apices. iNaturalist ID: 263684273. Mol. data: ERS27214058, ERS27214059, ERS27214060, ERS27214061, ERS27214062, ERS27214063, ERS27214064, ERS27214065, ERS27214066, ERS27214067, ERS27214068, ERS27214069, ERS27214070, ERS21395352, ERS21395356, ERS21395359, ERS21395369, ERS21395365 (Fig. 31).

### Shionodiscus endoseriatus

(Hasle & Fryxell) Alverson, Kang & Theriot

656DC94B-82EE-5DC4-882A-CFDA9C7CF387

Thalassiosira endoseriata Fryxell and Hasle (1977): 78, pl. 8, figs. 45–49—Fryxell and Hasle (1980); Rivera Ramírez (1981): 68, figs. 145–157; Mahood et al. (1986): 146, fig. 91; Hasle and Syvertsen (1997): 81, 82, pl. 13; Guiry and Guiry (2025).
Shionodiscus endoseriatus —Alverson et al. (2006); Wilks and Armand (2017).

#### Description

Diameter 30.0–54.1 (20–60) µm. Valve face flat or slightly convex. Irregular ring of 8–10 (4–14) central fultoportulae. Rimoportula is 7 (6) areolae from the margin towards centre of valve. Areolae usually fasciculate, 8–12 (11–18) in 10 μm at valve centre. MFP 6–8 (5–6) in 10 µm and extending into interior of frustule.

#### Notes

Specimens from the Strait of Georgia conform well with the published data. iNaturalist ID: 259613928 (Fig. 32).

### Shionodiscus frenguelliopsis

(Fryxell & Johansen) Alverson, Kang & Theriot

1C136809-A713-52A4-92CB-D45CEA0D6D49

Thalassiosira frenguelliopsis Johansen and Fryxell (1985): 168, figs. 6, 67, 68, 71, 81—Sar et al. (2002): 378, 379, figs. 2–6, table 3; Guiry and Guiry (2025).
Shionodiscus frenguelliopsis —Alverson et al. (2006): 259; Wilks and Armand (2017): tables 2, 11.

#### Description

Valves circular and flat, diameter 18.2 (12–34) μm, no external projections of processes. Areolae pattern on valve face irregularly eccentric to irregularly linear. Striae 28–30 (24–30) in 10 µm, areolae 20–22 (12–26) in 10 µm from centre towards mantle and 24 (18–20) near mantle. One (1) sub-central fultoportula off-centre and operculate. Rimoportula is between margin and centre, transverse (radial), 6–7 (5.8–8) areolae from the sub-central process. Ten MFPs are long and extend out internally, 3 in 10 µm, 10–11 areolae between MFPs, 4.1–5.5 (5–10) µm apart.

#### Notes

**Figure 33.**
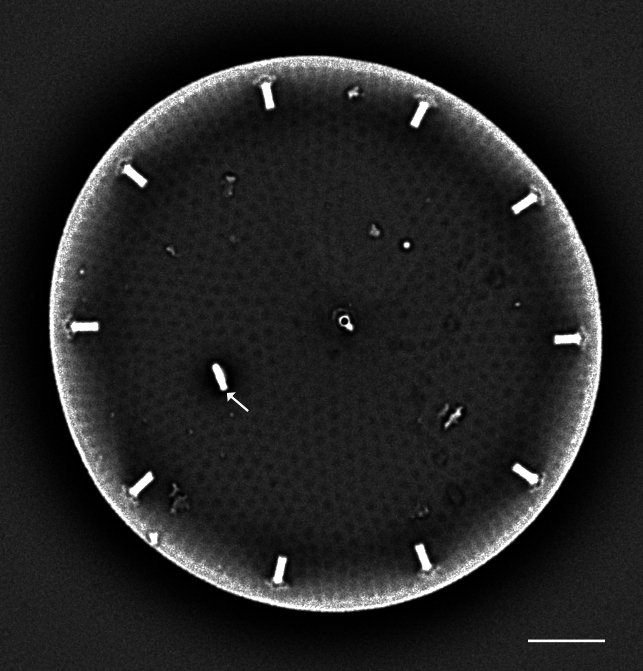
*Shionodiscus frenguelliopsis*, interior valve view. Arrow indicates location of the rimoportula. SEM. Scale Bar: 2.5 µm.

The morphometric data of this specimen are sufficient to place it as *S. frenguelliopsis*. iNaturalist ID: 301822368 (Fig. 33).

### Shionodiscus karianus

Georgiev & Gololobova

ABDFA921-98E0-51A1-BD2A-C3724C88BBB4

*Shionodiscus karianus Georgiev and Gololobova (2022)*.

#### Description

Valves circular and heavily silicified, diameter 30.8 (26–54) μm, no external projections of processes. Areolae pattern on valve face curved in tangential rows or in sectors of radial or sub-radial rows. Striae 10 in 10 µm, areolae 7 (7–11) in 10 µm from centre towards mantle and 8 (8–13) near mantle zone. Areolae loculate with internal domed cribra, slightly elevated relative to surrounding *hyaline* area, 6–7 (10–16) cribrum pores in 1 μm. One (1) central fultoportula process (CFP), operculate with 4 (4) satellite struts, 4 (4) pores with cowlings. Rimoportula radially orientated and located 1 (0–2) areolae, 1.2 µm (1.3–3.4) µm distance, from CFP. Four (4) marginal fultoportula processes (MFP), strutted, have 4 satellite pores, 15 (13–35) per valve. MFP internally long, more or less evenly placed around valve margin, 1.6 (1.5–2.4) in 10 µm, 4–6 (2–7) areolae between processes and 1.7–2.8 (2.7–6.5) µm apart.

#### Diagnosis

External valves, girdle band images and external valve ultrastructural details are lacking at this time. The *Shionodiscus* species closest to this specimen are *S. bioculatus*, S. bioculatus var. exiguus, *S. biporus*, *S. centrus*, *S. gaarderae* and *S. variantius*. All have an operculate CFP with a rimoportula that is located nearby or midway between the valve margin and CFP. All of the above species have areolae densities that are over double that of the SHW specimen. Only *S. bioculatus* and *S. gaarderae* have a rimoportula that is immediately adjacent to a central areola; however, the separation is more than 1 areolae distant, valve areolae density is too high, at 16–23 in 10 µm and MFP are 5–7 µm apart (Hasle and Syvertsen (1997): table 14, Ferrario et al. (2018): table 1, Georgiev and Gololobova (2022): table 2). Although *S. bioculatus* has been previously reported in the Salish Sea, the morphometric data of this SHW specimen are more than adequate to place it as *Shionodiscus karianus*.

#### Notes

iNaturalist ID: 322791476 (Fig. 34).

### Shionodiscus oestrupii

(Ostenfeld) A.J.Alverson, S.-H.Kang & E.C.Theriot

9BB2A47C-0E37-5E69-8DA7-D36EF84F0717

Coscinosira oestrupii Ostenfeld (1900): 52—Fryxell and Hasle (1980); Hasle and Syvertsen (1997): 81, 82, pl. 13.
Shionodiscus oestrupii —Alverson et al. (2006): 258; Wilks and Armand (2017); Guiry and Guiry (2025).

#### Description

Diameter 21.7–23.9 (7–60) μm, pervalvar axis half to twice the diameter. Valve face flat or slightly convex. Mantle low and rounded. Areolae usually larger in central part of valve than closer to the margin, sometimes in sublinear array. One nearly central strutted fultoportula. Rimoportulae is 3–4 areolae from central process. Areolae 9 (6–12) in 10 μm at valve centre. MFPs 3–4 in 10 µm, 0.96–1.1 (0.8–1.9) µm between MFPs.

#### Notes

iNaturalist ID:142951511, 142950297 (Fig. 35).

### Sundstroemia pungens

(A.Cleve) Medlin, Lundholm, Boonprakob & Moestrup

8DF4447D-A071-5363-A241-6459261C4708

*Rhizosolenia pungens Cleve-Euler (1937)*: 43, fig. 10—Hasle and Syvertsen (1997): 157; Bérard-Therriault et al. (1987); Bérard-Therriault et al. (1999): 37, pl. 21g, m; Hoppenrath et al. (2009): 63, fig. 27a–c; Yun and Lee (2011): 142–145: fig. 1A–F.
Sundstroemia pungens —Medlin et al. (2021): 242; Boonprakob et al. (2021): 162–164, figs. 106–118; Guiry and Guiry (2025).

#### Description

Cells solitary and narrow, diameter 16.0 (4–20) µm, length up to 213.2 (150–589) µm without processes, process length (85–276) μm. Valves conical. External processes long, almost straight and needle-like. Basal part of process narrow, abruptly swollen for about half its length, narrow again and gently tapering towards tip. Otarium absent. Girdle composed of segments in two dorsiventral columns. Numerous chloroplasts.

#### Diagnosis

Very similar in morphology to *Sundstroemia setigera*. However, the narrow basal section, then a swollen zone of the process in *S. pungens* (seen in LM and SEM), differentiates it from *S. setigera*, which has a long and continuously shaped narrow process.

#### Notes

*Sundstroemia pungens* is found in the plankton throughout most of the year, but more frequently observed in the Trincomali Channel and Porlier Pass in September and October. A small bloom of *S. setigera* occurred at SHW on 21 July 2025. iNaturalist ID: 259931970, 315872333. Mol. data: SRS969492, SRS966492 (Fig. 36).

### Tabellaria quadriseptata

B.M.Knudson

AFFCEE67-40C6-577C-B7A7-D5CFDEA0B730

*Tabellaria quadriseptata Knudson (1952)*: 436, figs. I–N—Patrick and Reimer (1966): 105, pl. 1, fig. 3; Flower and Battarbee (1985); Sims (1996): pl. 284, figs. 6, 7; Plinski and Witkowski (2020): 56, fig. 212.

#### Description

Colonies of zigzag filaments, linked together at corners by mucilage pads. Valve median and apical inflations approximately same width. Apical inflations not distinctly capitate as in *Tabellaria fenestrata*. Axial area narrow. Valves linear with parallel margins between inflated area and poles. Length 70.7 (23–130) µm, width 5.7 (6–9) µm. Septa straight and few, usually four. Single rimoportula located adjacent to axial area at edge of central inflation, at base of valve shaft or periphery of median inflation. Striae density 14 (13–20) in 10 µm. Marginal spines short, length up to 1 µm, with equal distribution.

#### Diagnosis

The morphometric data of the MBW specimen fit the descriptions in literature for *T. quadriseptata*: approximately the same width for median and apical inflations (5.7 and 4.7 µm, respectively), parallel margins, location of the rimoportula and the presence of relatively equally spaced spines along the margins. However, currently there are no images of the septa, so the specimen is assigned T. cf. quadriseptata.

#### Notes

iNaturalist ID: 259219380 (Fig. 37).

### Thalassiosira allenii

H.Takano

2432209A-EB46-55F6-9C79-ACF3DAE6549E

Thalassiosira allenii Takano (1965): 4, pl. 1: figs. 9–11, pl. 2: fig. 1—Hasle (1978a); Hasle and Syvertsen (1997): 51, table 7, pl. 4; Jameson and Hallegraeff (2010): 57, fig. 2.18B, C; Li et al. (2013): 85, 86, figs. 2–5; Hernández-Becerril et al. (2015); Park et al. (2016); Efimova et al. (2023).

#### Description

Valve diameter 17.2 (5–20) µm. Areolae on valve face 22–24 (18–28) in 10 µm in fasciculate pattern, with smaller areolae 30–40 in 10 µm on narrow valve mantle. Central, non-prominent, central process (fultoportula) located next to one, two or three larger areolae. Valve may be covered by minute granules (not observed). Marginal processes long and coarse, 6 (5–9) in 10 µm. External rimoportula, single and prominent, takes place of and is occasionally very slightly inside of the ring of marginal strutted process. Mantle narrow, oblique and angled, 5 (2–3) areolae wide.

#### Diagnosis

*Thalassiosira allenii* differs from *T. nordenskioeldii* in that the former has a lower mantle in girdle view, a higher areolae density on the valve face and mantle, marginal processes that are closer together and have no collars, processes that are stouter and mantle areolae that are smaller than those in *T. nordenskioeldii*.

#### Notes

iNaturalist ID: 259835781 (Fig. 38).

### Thalassiosira curviseriata

Takano

FF4993C8-C989-5AAC-8025-DC51A56C7DA7

Thalassiosira curviseriata Takano (1983): 32—Hallegraeff (1984); Hasle and Syvertsen (1997); Hoppenrath et al. (2007); Li et al. (2014); Park et al. (2016).

#### Description

Cells discoid, diameter 13.1 (5–14) μm. Valve face flat with central concavity. Areolae in radial rows, siliceous granules all over valve surface, occasional silicious spinules on surface. One sub-central strutted process. Central process adjacent to a more or less off-centred annulus. One marginal ring of five conspicuous winged strutted processes, two wings per process opposed into two or three branches. Marginal processes 2–3 (2–3) in 10 µm, 5.1–5.9 µm apart.

#### Notes

This specimen conforms with morphometric data from Hallegraeff (1984), Hasle and Syvertsen (1997), Hoppenrath et al. (2007), Li et al. (2014) and Park et al. (2016). Mol. Data: ENA:ERS27630061. iNaturalist ID: 142613095 (Fig. 39).

### Thalassiosira minima

Gaarder

85BEAD0A-0153-5BC2-81C4-D819A6535824

Thalassiosira minima Gaarder (1951): 31, fig. 18—Hasle (1980): 167, figs. 1–17; Rivera Ramírez (1981): 90–94, pl. 36–38; figs. 226–245; Harris et al. (1995): 119, table 1, fig. 14; Hasle and Syvertsen (1997): 65, table 9; Bérard-Therriault et al. (1999): 25, 26, pl. 11g–f; Li et al. (2014): 388, figs. 50–54.

#### Description

Valve diameter 9.9 (5–15) μm. Valve face flat and slightly depressed in the centre; mantle low and bevelled. Two central processes. Areolae arranged in radial or fasciculate rows and decrease slightly in size (increase in density) from 28 (22–40) in 10 μm near centre to 32 (30–50) in 10 μm towards valve margin. Normally two (2) fultoportulae, seldom one or three, present near valve centre and always surrounded by several larger areolae. MFPs, 4 (4–5) in 10 μm, each bearing external tube and keeping one areola inside from a small siliceous granule. One marginal rimoportula, with long external tube, included in MFPs.

#### Notes

**Figure 40.**
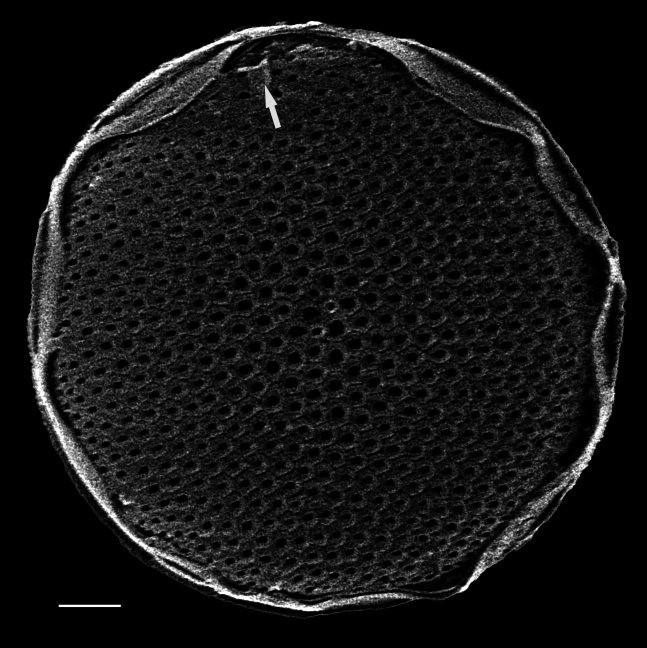
*Thalassiosira minima*, external valve view. Arrow indicates location of the rimoportulae. SEM. Scale bar: 1 µm.

iNaturalist ID: 259931970 (Fig. 40).

### Thalassiosira oceanica

Hasle

5E8C2416-F10D-5E20-8B81-13B607DFAC40

Thalassiosira oceanica Hasle (1983): 220, table 1, figs. 1–18—Harris et al. (1995): 121, figs. 8, 26; Hasle and Syvertsen (1997): 56, 57, table 7; Bérard-Therriault et al. (1999): 26, pl. 11d; Hoppenrath et al. (2007): 59, fig. 24a; Li et al. (2014): 390, fig. 62.

#### Description

Cells rectangular in girdle view, pervalvar axis slightly shorter than cell diameter 6.8–7.3 (3.0–12.0) µm. Valve has fine ornamentation of silicious radial ribs and poroidal areolation, 50–60 (40–60) areolae in 10 µm. Valve has undulating marginal ridge. One central strutted process, located slightly sub-central. Single marginal ring of 4–6 (3–8) relatively widely spaced strutted processes in 10 µm without external tubes, 2 µm apart. One labiate process close to one marginal strutted process. Valve rim has ribs.

#### Notes

**Figure 41.**
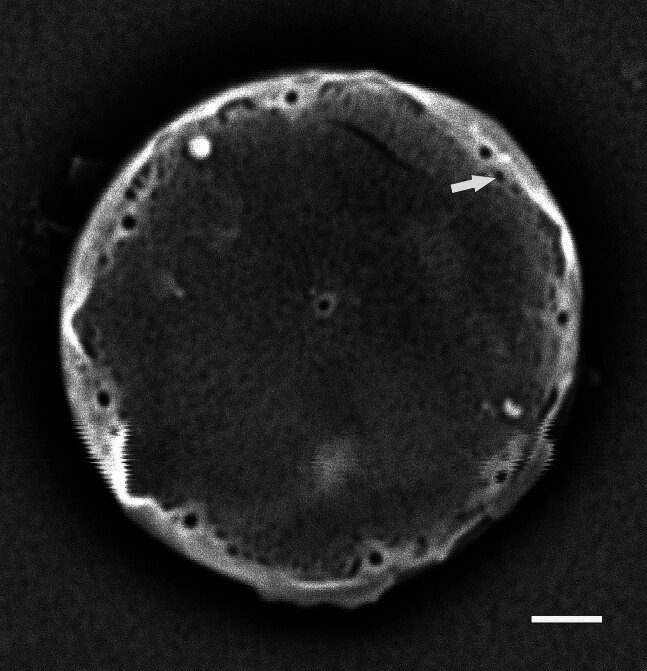
*Thalassiosira oceanica*, external valve view. Arrow shows location of rimoportula adjacent to an external marginal process. SEM. Scale bar: 1 µm.

The morphometric data of this specimen fit the description in literature and is supported by molecular data. iNaturalist ID: 259589003. Mol. data: SRS968559, SRS964086, SRS966570, SRS1690317, SRS966764, SRS969492, SRS968562, SRS966566, SRS968542, SRS966614, SRS967659, SRS968132, SRS964211, SRS966901 (Fig. 41).

### Thalassiosira visurgis

Hustedt

833F9301-AAA5-50D3-B745-5FECC8B23873

*Thalassiosira visurgis Hustedt (1957)*: 207, pl. 1: figs 1-4—Simonsen (1987): 439, pl. 657, figs. 1–9; Hasle (1978b): 263, 264, figs. 1–4; Mahood et al. (1986): 138, figs. 56–61, 95, 96; Aké-Castillo et al. (1999): 497, figs. 45, 46; Li et al. (2013): 104, 105, figs. 126–128.

#### Description

Valve face flat, concave or convex; diameter 14.0 (12.5–18.0) μm. Areolae arranged in linear or eccentric lines, 10 (12–15) in 10 μm on valve face, 28–32 (17–20) in 10 μm on mantle. One fultoportula present in centre close to large areola. Valve margin characterised by ring of disorganised fultoportulae, 4–5 (4–10) in 10 µm and distinctly short and dentate external tubes. Two rimoportulae with external tubes having variable length and smooth openings, separated 120°–180° apart, located on opposite sides and slightly inside ring of marginal fultoportulae, with each rimoportula taking the place of a fultoportula. Valve mantle ornamented with ribbed rim. Fine silicious granules on valve extend to margin.

#### Diagnosis

**Figure 42.**
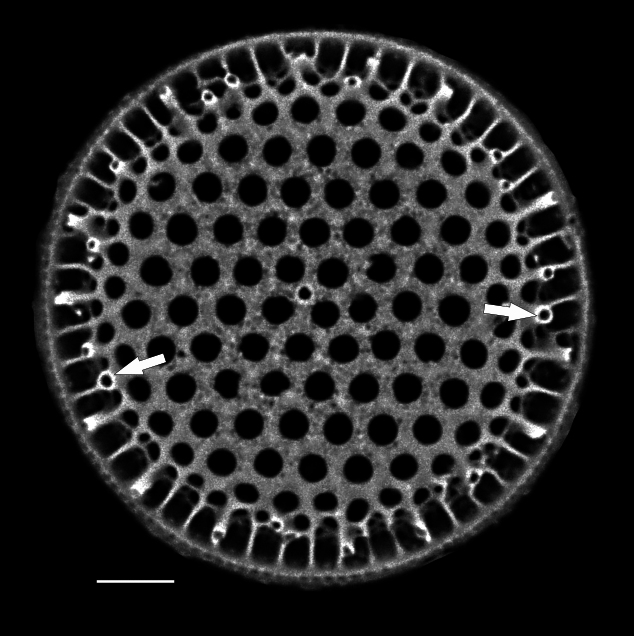
Thalassiosira cf. visurgis, external valve view, arrows indicate the locations of the two rimoportula. SEM. Scale bar: 2 µm.

Some variability in morphological characters exists within specimens identified as *T. visurgis* in literature. The number and position of MFSs in the Galiano Island specimen are similar to those described by Hasle (1978b) and Li et al. (2013), but dissimilar to those observed in Mahood et al. (1986). The MHMPP specimen has both a pronounced ribbed margin and two slightly larger external tubes (indicated by arrows in Fig. 42) that are presumably rimoportulae with smooth openings, as they are in the correct position, but that are shorter than described in literature, with the exception of fig. 3a in Hasle (1978b). The major difference between the MHMPP specimen and literature describing *T. visurgis* is the organisation of the marginal processes. If we compare *T. visurgis*, as described in Hasle (1978b), Mahood et al. (1986) and Li et al. (2013), with *T. elsayedii* (Fryxell 1975), the latter species has the following characteristics: 1) areolae appear smaller, but are similar in density, at 9–11 in 10 µm; 2) external processes have spines; 3) the number of striations (ribbed margin) are more numerous and 4) Fryxell (1975) makes no mention of granulation on the valve surface, nor do granules appear in her images. Thus, *T. elsayedii* is similar to *T. visurgis* in having two labiate processes on opposite sides of the valve, but is dissimilar in four other characteristics. The MHMPP specimen is unlikely to be *T. baltica*, which has 2–9 central strutted processes. However, the marginal process patterning in *T. baltica* is clearly in two rows, which is somewhat similar to the MHMPP specimen. Until we study more material, we will consider the MHMPP specimen to be T. cf. visurgis, as it fits most closely with descriptions in literature.

#### Notes

iNaturalist ID: 260003450 (Fig. 42).

### Trigonium quinquelobatum

(Greville) A.Mann

95DDA7D0-838A-5218-A4CA-5CD97C32DDE6

Triceratium quinquelobatum Greville (1866): 83, pl. IX, fig. 21—Tempère and Peragallo (1908): pl. 104, fig. 2; Mann (1925): 171; Hustedt (1930b): 820, fig. 482.
Trigonium quinquelobatum —Mann (1925): 171.
Trigonium arcticum var. quinquelobatum (Greville) Desikachary and Ranjitha (1986): 7, pl. 68, fig. 4; Guiry and Guiry (2025).

#### Description

Valve pentagonal in outline and thickly silicified. Length between adjacent and slightly non-equidistant corners 96.7–118.6 (61) µm. Convex sides. Areolae coarsely polygonal, many hexagonal, arranged in radiating and decussating rows, 2–6 (2–6) in 10 µm in mid-valve and finer approaching corners. Areolae 4–6 in 10 µm approaching pseudocelli immediately transition to tri- to bi-lobed pores 12–13 in 10 µm, then 16–19 in 10 µm into the slightly elevated pseudocelli. Some lines of areolae discontinuous from margin. Areolae flattened and occluded by a silica layer (vela), usually 2.3–5 µm wide, with distinct papilla in middle of many areola (only observed with LM) and ring of 5–13 (average 9) small, round pores formed by attachment spokes (rotae). Central sub-circular areola surrounded by rosette of 7–9 slightly smaller areolae, often elliptically-elongated. Externally, exit holes of rimoportulae round and inconspicuous, with 12–17 small protuberances scattered in centre of valve face. Internally, rimoportulae equally small and stalkless with tiny slits, occasionally with multiple openings. Unraised pores scattered across external and internal surfaces, round to oval, connected to loculate chambers. Narrow septum of valve wall occurs at each corner. Mantle deep, steep and vertical. Girdle deep with closed bands containing transverse rows of seven areolae in 10 µm. Silica nodules and short spines occur along edge of valvocopula.

#### Diagnosis

The genera *Trigonium* and *Triceratium* are in desperate need of revision. AlgaeBase (Guiry and Guiry 2025) lists occurrences of *Trigonium quinquelobatum* from the Philippines (Mann 1925), Colombia (Lozano-Duque et al. 2010) and Korea (Lee et al. 1995). The original description is a fossil from a deposit in Spain (Greville (1866): 83, pl. IX: fig. 21) that conforms well with our pentagonal specimens found in Porlier Pass. AlgaeBase notes a report from Cuba (Foged (1984); no mention of *T. quinquelobatum*, yet a different genus, *Triceratium pentacrinus*, is illustrated in pl. 23, figs. 3, 4). The genus *Trigonium* is readily separated from *Triceratium*, as the former has pseudocelli and the latter has true ocelli that have a distinctive silica ring around the pore field (Ashworth et al. 2013). *Triceratium formosum* Brightwell (Brightwell (1856): 274, pl. 17, fig. 8) is not a *Triceratium*, since it lacks true ocelli and is quadrangular in outline. Triceratium formosum f. quinquelobata (Hustedt (1930b): 819, 820, fig. 482), as Hustedt notes, is likely synonymous with the above-described *T. quinquelobatum* (Greville 1866). Unlike in the triangular- and quadrangular-shaped Trigonium arcticum and var. quadratum found in Salish Sea waters, *T. quinquelobatum* has a distinctive papilla (seen with LM), possibly due to the size and shape of the foramina in the centre of most areolae, a characteristic noted by Mann (1925), who designated the pentagonal form as a separate species. Additionally, *T. quinquelobatum* has a central areola surrounded by 7–9 slightly smaller areolae, giving the appearance of a spiral rosette. No silica granules were observed around the areolae, as is commonly found on Trigonium arcticum and var. quadratum.

#### Notes

*Trigonium quinquelobatum* is rare in Salish Sea waters, so far found only once on a leafy red alga at depth in Porlier Pass, near Dionisio Point, Galiano Island. iNaturalist ID: 259931970, 300280287 (Fig. 43 and Fig. 44).

## Analysis

**Figure 45.**
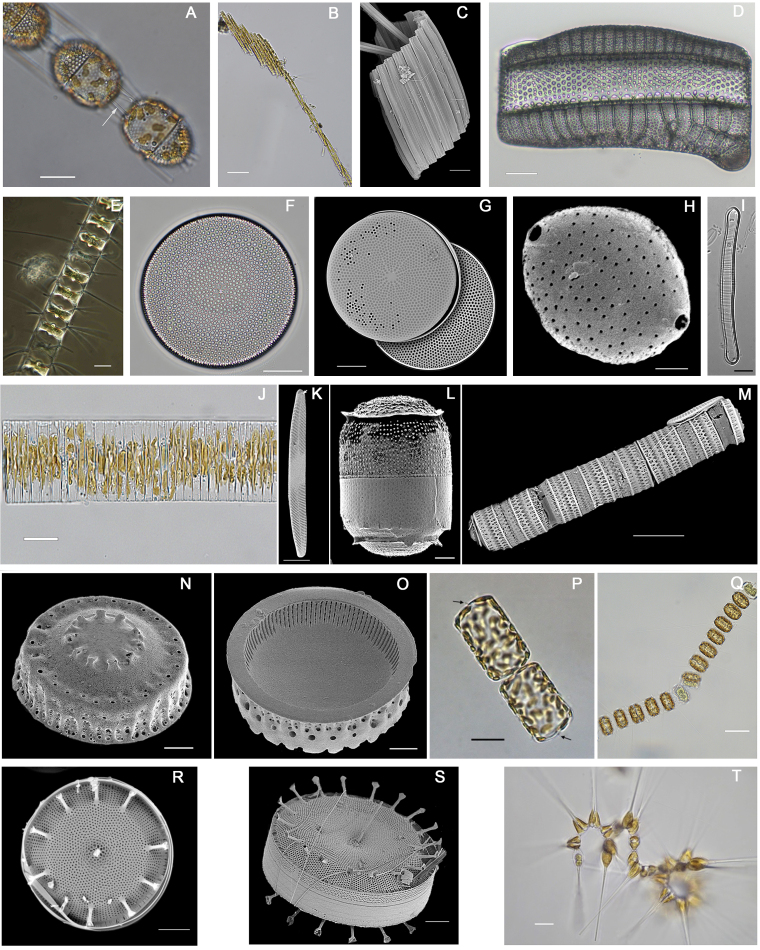
Examples of diatoms (by subclass) found within the Salish Sea. **A** Archaegladiopsophycidae: *Stephanopyxis niponnica*. Girdle view. Arrow shows a continuous process between cells. LM. iNat: 45809431; **B** Bacillariophycidae: *Bacillaria paxillifera*. Live. Girdle view. LM. iNat: 192871056; **C** *Bacillaria paxillifera*. Girdle view and partial valve view. SEM. iNat: 192871056; **D** Biddulphiophycidae: *Isthmia nervosa*. Girdle and valve views. LM. iNat: 194751473, 5197632; **E** Chaetocerotophycidae: *Chaetoceros didymus*. Live. Girdle view. LM. iNat. 4849241; **F** Coscinodiscophycidae: *Coscinodiscus radiatus*. Exterior valve view. LM, cleaned. iNat: 40703800; **G** *Coscinodiscus radiatus*. Exterior and interior valve views. SEM. iNat: 40702227; **H** Cymatosirophycidae: *Extubocellulus spinifer*. Exterior valve view. SEM. iNat: 311561542; **I** Eunotiophycidae: *Eunotia formica*. Valve view. LM, cleaned. iNat: 316197977; **J** Fragilariophycidae: *Fragilaria striatula*. Exterior girdle view. LM. iNat: 243251313; **K** *Fragilaria striatula*. Exterior valve view, single frustule, tilted. Arrow indicates the exit hole of the single rimoportula. SEM. iNat: 243251313; **L** Melosirophycidae: *Melosira nummuloides*. Girdle view. SEM. iNat: 205351626; **M** Paraliophycidae: Paralia cf. sulcata. Girdle view. SEM. iNat: 4873115, 189407891; **N** *Paralia sulcata*. Exterior valve view. iNat: 189407891; **O** *Paralia sulcata*. SEM. Interior valve view. iNat: 189407891; **P** Rhizosoleniophycidae: *Dactyliosolen fragilissimus*. Girdle view. Arrows indicate the location of the external process on each valve. LM. iNat: 50047033; **Q** Thalassiosirophycidae: *Thalassiosira nordenskioeldii*. Chain of cells. Girdle view. LM. iNat: 111064233; **R** *Thalassiosira nordenskioeldii*. Valve view. SEM. iNat: 143410067; **S** *Thalassiosira nordenskioeldii*. Girdle and valve views. SEM. iNat: 201221176; **T** Urneidophycidae: *Asterionellopsis glacialis*. Live. Girdle and valve views. LM. iNat: 111057760, 98753620. Scale bars: D = 50 µm; Q = 40 µm; B, J, T = 25 µm; A, E–G, I, M, P = 20 µm; C, K = 10 µm; R, S = 5 µm; L, N, O = 2 µm; H = 0.5 µm.

Through systematic review of literature and synthesis of existing data, including morphological and molecular data from our sampling, we report 924 diatom species/infrataxa for the Salish Sea. These reports represent three classes, 13 subclasses, 40 orders, 80 families and 207 genera, which together reflect a broad range of morphological diversity (Fig. 45). Of these records, 42 constitute novel reports of taxa that were previously unreported for the region (Table 3). In total, we synthesised 11,469 records, of which 9,049 were resolved to genus, species or infrataxon (Suppl. material 8), in alignment with the names reported in the annotated checklist (Suppl. material 1).

## Discussion

This work establishes a baseline record of diatom diversity reported for the Salish Sea. Drawing on historical records dating from the 1860s to present, together with existing collections and our recent taxonomic and molecular analysis of plankton, eelgrass, macroalgae, benthic and epipsammic samples, we have added 42 species (including six new genera) to the regional record, bringing the total to 924 taxa. Supporting this work are 31 amplicon sequence libraries now publicly accessible via the European Nucleotide Archive (ENA accession: PRJEB102646) and a small set of Sanger sequence data (ENA accession: ERZ29258993). Together, these resources form the foundation for an ongoing effort involving both community and academic research scientists to document and validate the regional diatom diversity.

This comprehensive baseline creates new opportunities for future work in the Salish Sea, such as investigations of regional and global diatom biogeography (e.g. Mazur-Marzec et al. 2024, Williams 2025), assessments of human-mediated dispersal events (Villac et al. 2017), broader ecological studies including the marine microbiome (e.g. Mazur-Marzec et al. 2024, Jackman et al. 2025) and long-term monitoring of environmental change (e.g. Bopp et al. 2005, Edwards et al. 2022). These potential applications underline the value of a well-resolved regional inventory for addressing persistent shortfalls in biodiversity knowledge (Hortal et al. 2015).

Notable reports include taxa previously unreported for the Pacific coast of North America (e.g. *Trigonium quinquelobatum*) and those previously known only from warmer waters, such as *Cocconeis kerguelensis* (Indian Ocean), *Isthmiа enervis* and *Neocalyptrella robusta* (California). As we resolve a clearer picture of the regional diversity, taxonomic gaps are also becoming apparent: *Trigonium*, *Triceratium*, *Hyalodiscus*, small-celled araphids and the substantial undescribed diversity within *Cocconeis*, all emerge as clear priorities for future taxonomic work. Ecological patterns have also emerged: for example, *Tabularia*, a species-poor, yet abundant genus, stands out as a dominant component of epiphytic assemblages on eelgrass and macroalgae (Jackman et al. 2025). In contrast, genera such as *Auliscus*, *Biddulphia* and *Mastogloia*, are species-poor and rare in the Salish Sea despite being more diverse and widespread elsewhere, raising questions about the factors limiting their regional occurrence.

While our work showcases the contributions that community science can make to phycology (Bahls 2015), we acknowledge the many challenges that impede reliable diatom identification — challenges that have historically hindered professional and amateur diatomist alike. Looking back, we acknowledge the consequences of the Linnaean shortfall for the reliability of the historical record: as taxonomic knowledge has expanded over the last century, species concepts and diagnostic criteria have changed substantially, as well as the tools we use to study diatoms. Historical identifications should thus be interpreted with the appropriate discretion. Looking forward, we also recognise the implications of the Darwinian shortfall: given the inherent limitations of short-read metabarcoding and the incomplete availability of reference sequences, molecular identifications likewise warrant cautious interpretation.

Addressing these challenges today requires an integrated approach that combines molecular, morphological and culture-based techniques. Our results highlight the value of microscopy and cloning as complementary to environmental DNA, especially while marine diatom reference sequences remain under-represented in molecular databases (Hamsher et al. 2013, Chen et al. 2024, Mazur-Marzec et al. 2024, Jackman et al. 2025). Light microscopy remains essential for clarifying certain diagnostic features (e.g. partecta in *Mastogloia*), while SEM is indispensable for resolving ultrastructural traits, including areolae architecture and small rimoportulae that cannot be seen under LM (Li et al. 2018). Culture isolation and cloning yield the most reliable validation by enabling LM, SEM and molecular analyses to be performed on near-axenic and axenic material (Rad‑Menéndez et al. 2015, Witkowski et al. 2016; Mönnich et al. 2020). However, establishing and maintaining cultures remains a major bottleneck; the work is time-consuming, technically demanding and constrained by limited personnel and resources. Access to authoritative diatom monographs, reference books and historical literature — much of it unavailable in digital form — has also proven crucial for accurate diatom identification.

As we sample a broader range of habitats, our integrative morphological and molecular approach continues to yield new records of small, taxonomically problematic and cryptic taxa, including the recently described *Andrzeja fenestrata*, as well as *Extubocellulus* sp., *Gedaniella* sp., *Opephora*-like and other small-celled raphids and araphids (Vaulot et al. 2008, Li et al. 2018, Pérez-Burillo et al. 2022, Jackman et al. 2025, Mayama et al. 2025). These findings demonstrate that community science can fill major data gaps through the generation of high-resolution datasets (Theobald et al. 2015, Chandler et al. 2017, Peter et al. 2021), highlighting its potential to play a more central role in the global research community (Simon et al. 2022). Although community science contributions to biodiversity monitoring have been valued at roughly US$2.5 billion annually (Theobald et al. 2015, Chandler et al. 2017), substantial geographic and taxonomic gaps remain and much of the data never reaches the peer-reviewed literature (Theobald et al. 2015, Chandler et al. 2017). Our study demonstrates how sustained collaboration between community scientists and research institutes can bridge these gaps by partnering community expertise and observation with access to microscopy and molecular technologies (Bahls 2015). Based on these early results, we anticipate that this approach will continue to reveal overlooked diversity while strengthening the baseline record for phytoplankton monitoring in the Salish Sea.

## Supplementary Material

XML Treatment for Achnanthes groenlandica meridiana

XML Treatment for Actinoptychus adriaticus pumila

XML Treatment for Andrzeja fenestrata

XML Treatment for Attheya longicornis

XML Treatment for Bacteriastrum hyalinum

XML Treatment for Bacterosira constricta

XML Treatment for Campylodiscus bicostatus

XML Treatment for Cocconeis costata hexagona

XML Treatment for Cocconeis kerguelensis

XML Treatment for Cocconeis notata

XML Treatment for Cocconeis pseudomarginata intermedia

XML Treatment for Cocconeis scutellum posidoniae

XML Treatment for Cyclotella baltica

XML Treatment for Didymosphenia geminata

XML Treatment for Diploneis exemta

XML Treatment for Extubocellulus spinifer

XML Treatment for Fogedia krammeri

XML Treatment for Gomphonemopsis pseudexigua

XML Treatment for Gomphoseptatum aestuarii

XML Treatment for Gomphoseptatum pseudoseptatum

XML Treatment for Gyrosigma arcuatum

XML Treatment for Homoeocladia spathulatoides

XML Treatment for Isthmia enervis

XML Treatment for Licmophora communis

XML Treatment for Licmophora tincta

XML Treatment for Minidiscus proschkinae

XML Treatment for Neocalyptrella robusta

XML Treatment for Parlibellus delognei ellipticus

XML Treatment for Proschkinia complanatula

XML Treatment for Seminavis robusta

XML Treatment for Shionodiscus endoseriatus

XML Treatment for Shionodiscus frenguelliopsis

XML Treatment for Shionodiscus karianus

XML Treatment for Shionodiscus oestrupii

XML Treatment for Sundstroemia pungens

XML Treatment for Tabellaria quadriseptata

XML Treatment for Thalassiosira allenii

XML Treatment for Thalassiosira curviseriata

XML Treatment for Thalassiosira minima

XML Treatment for Thalassiosira oceanica

XML Treatment for Thalassiosira visurgis

XML Treatment for Trigonium quinquelobatum

05CDF445-CA8F-5D79-8AC9-299EF59DA172 10.3897/BDJ.14.e189060.suppl1 Supplementary material 1 Diatoms (Bacillariophyta) of the Salish Sea, Northeast Pacific (1866–2026): Annotated Checklist Data type annotated checklist Brief description Annotated checklist of diatoms reported for the Salish Sea (1866–2026). File: oo_1598027.pdf https://binary.pensoft.net/file/1598027 Mark Webber, Arjan van Asselt, Alice Chang, Andrew Simon

60941A5C-8B4C-5D69-ACF6-83333EC6A6F2 10.3897/BDJ.14.e189060.suppl2 Supplementary material 2 Diatoms (Bacillariophyta) of the Salish Sea, Northeast Pacific (1866–2026): Annotated Checklist Data type annotated checklist (CSV format) Brief description Annotated checklist of diatoms reported for the Salish Sea (1866–2026) - summary in CSV format (used as the basis for the formatted checklist provided as PDF). File: oo_1598028.csv https://binary.pensoft.net/file/1598028 Mark Webber, Arjan van Asselt, Alice Chang, Andrew Simon

CDA5F61F-CA7E-5B2D-9972-01702E792643 10.3897/BDJ.14.e189060.suppl3 Supplementary material 3 Scagelia sample - Miners Bay, Mayne Island, BC - manually curated 18S ASV taxon table Data type manually curated ASV taxon table (18S) File: oo_1572887.csv https://binary.pensoft.net/file/1572887 Mark Webber, Evan Morien, Andrew Simon

42D3185F-4C73-5DFD-9C66-DBDF5E477806 10.3897/BDJ.14.e189060.suppl4 Supplementary material 4 Zostera marina samples - Montague Harbour - manually curated 18S ASV taxon table Data type manually curated ASV taxon table (18S) File: oo_1572894.csv https://binary.pensoft.net/file/1572894 Mark Webber, Evan Morien, Andrew Simon

5CB4413C-2715-538F-8BE9-F4011F4AD7FB 10.3897/BDJ.14.e189060.suppl5 Supplementary material 5 Zostera marina samples - Montague Harbour - manually curated rbcL ASV taxon table Data type manually curated ASV taxon table (rbcL) File: oo_1572898.csv https://binary.pensoft.net/file/1572898 Mark Webber, Evan Morien, Andrew Simon

9532727B-E1EF-507B-8181-97E8A38E7E1E 10.3897/BDJ.14.e189060.suppl6 Supplementary material 6 plankton samples - Salish Sea - manually curated rbcL ASV taxon table Data type manually curated ASV taxon tables (rbcL) File: oo_1572899.csv https://binary.pensoft.net/file/1572899 Mark Webber, Evan Morien, Andrew Simon

7ABA2043-6969-5505-830F-9741AA8CF1F1 10.3897/BDJ.14.e189060.suppl7 Supplementary material 7 Galiano BioBlitz 2023 - Galiano Island, BC - manually curated rbcL ASV taxon table Data type manually curated ASV taxon table (rbcL) File: oo_1572901.csv https://binary.pensoft.net/file/1572901 Mark Webber, Evan Morien, Andrew Simon

92DB1732-A587-58D4-A9C5-BC0143CED14C 10.3897/BDJ.14.e189060.suppl8 Supplementary material 8 Diatoms (Bacillariophyta) of the Salish Sea, Northeast Pacific (1866–2026): Catalogue of occurrence data (aligned) Data type occurrences Brief description Catalogue of occurrence data used for baseline analysis - all records aligned with the curated summary of unique taxa reported in the annotated checklist. File: oo_1598029.csv https://binary.pensoft.net/file/1598029 Mark Webber, Evan Morien, Arjan van Asselt, Andrew Simon

D2AE6225-65E1-5260-8C73-35C7D7AA23BC 10.3897/BDJ.14.e189060.suppl9 Supplementary material 9 Diatoms (Bacillariophyta) of the Salish Sea, Northeast Pacific (1866–2026): Catalogue of occurrence data (complete) Data type occurences Brief description Catalogue of occurrence data - all diatom records (including taxa unresolved beyond family or genus). File: oo_1598030.csv https://binary.pensoft.net/file/1598030 Mark Webber, Evan Morien, Arjan van Asselt, Andrew Simon

## Figures and Tables

**Figure 2a.**
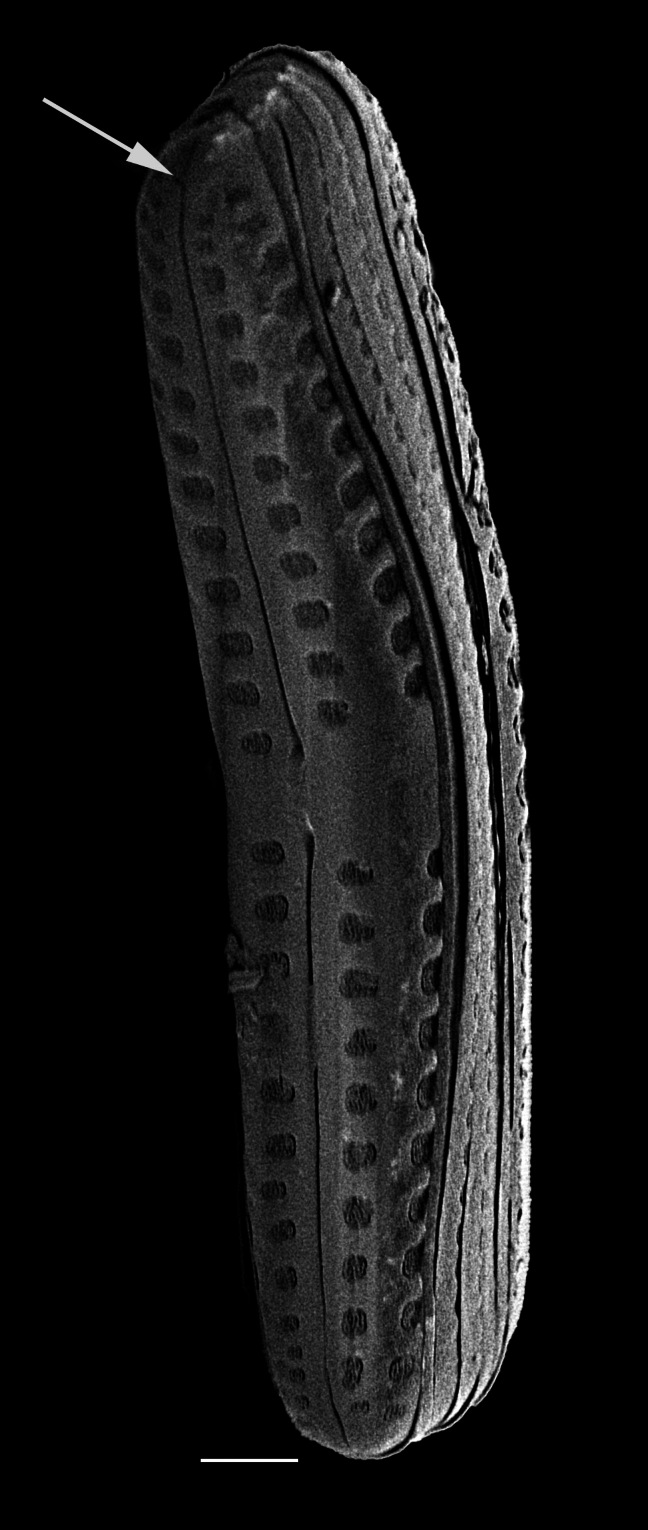
Achnanthes groenlandica var. meridiana, exterior RV. SEM. Scale bar: 2 µm;

**Figure 2b.**
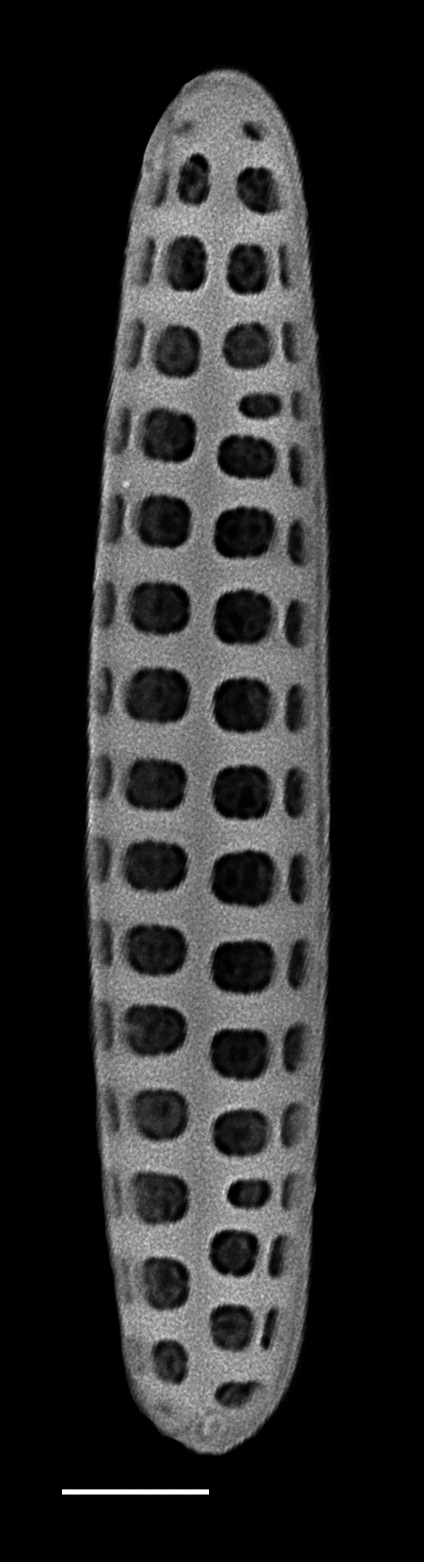
Achnanthes groenlandica var. meridiana, exterior RLV and girdle view. SEM. Scale bar: 2.5 µm.

**Figure 4a.**
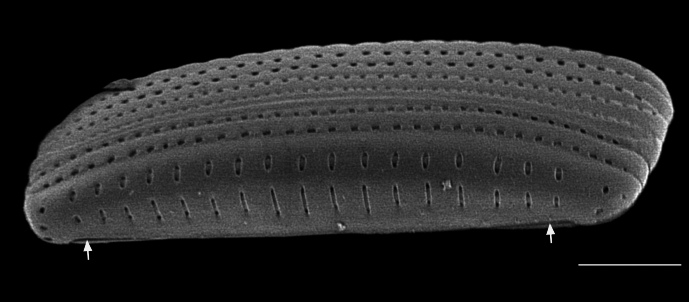
*Andrzeja fenestrata*, exterior, girdle and valve views. Arrow indicates location of raphe along part of the valve and mantle edge. SEM. Scale bar: 2 µm;

**Figure 4b.**
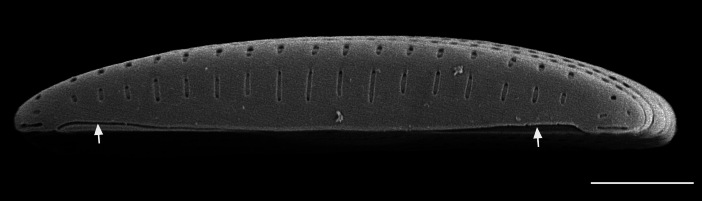
*Andrzeja fenestrata*, valve view. Tilted, with a section of vental valve showing. Arrows indicate location of raphes. SEM. Scale bar: 2 µm;

**Figure 4c.**
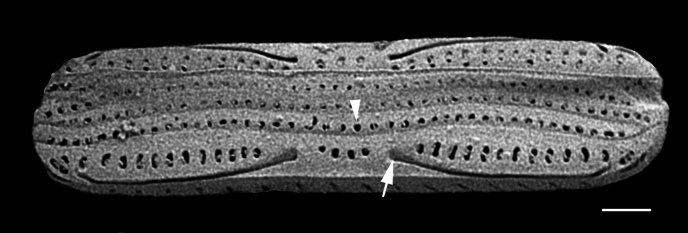
*Andrzeja fenestrata*, ventral valve and girdle views. Short arrow indicates location of four girdle bands with simple singular row of pores. Longer arrow shows raphe on ventral valve side and proximal termination ending in a helictoglossa with a slight rim. SEM. Scale bar: 1 µm;

**Figure 4d.**
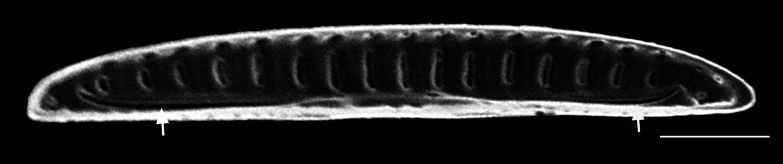
*Andrzeja fenestrata*, interior valve view. Arrows showing distal raphe termination ending in a helictoglossa. Scale bar: 2 µm.

**Figure 5a.**
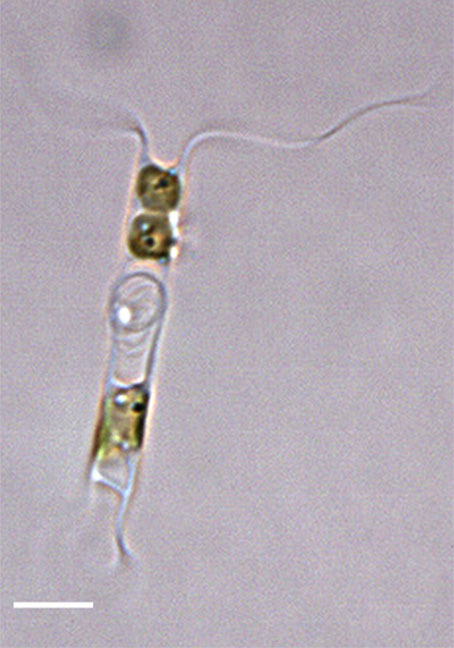
*Attheya longicornis*, girdle view. LM. Scale bar: 10 µm;

**Figure 5b.**
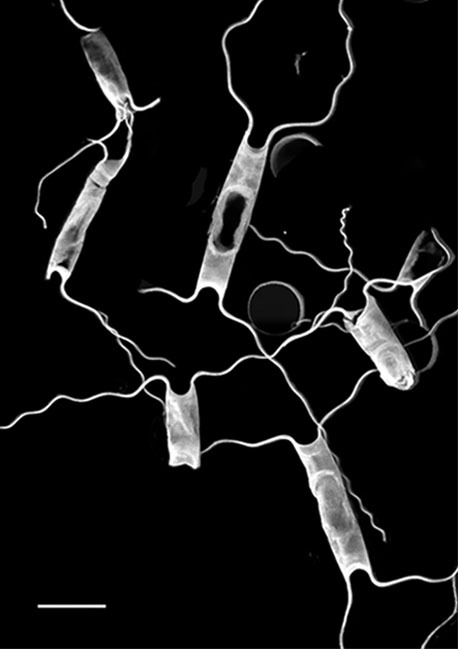
*Attheya longicornis*, girdle view. SEM. Scale bar: 10 µm;

**Figure 5c.**
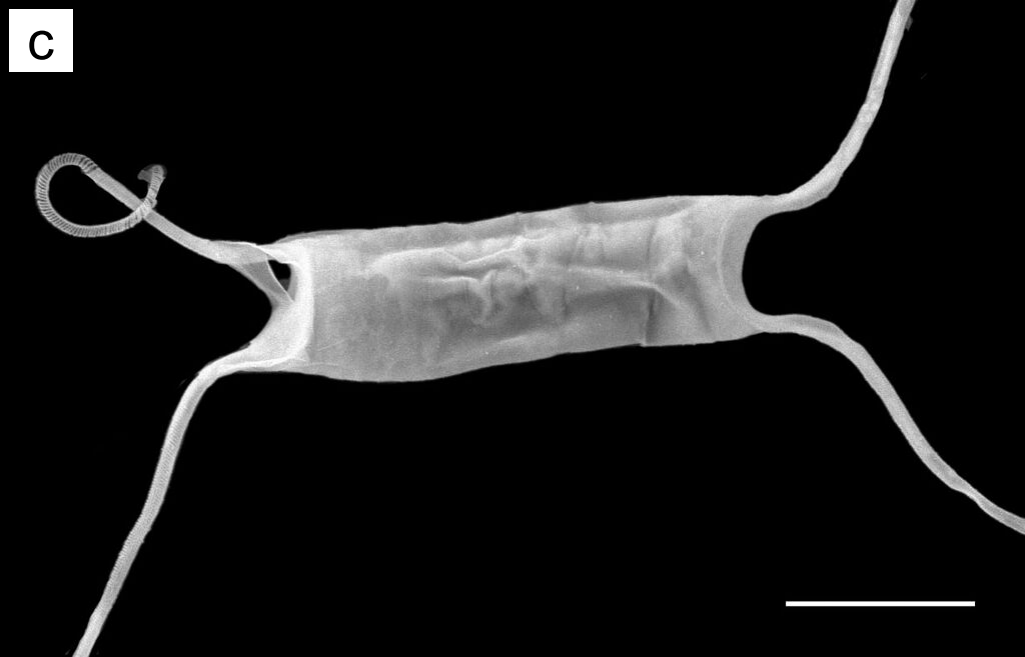
*Attheya longicornis*, girdle view. SEM. Scale bar: 5 µm;

**Figure 5d.**
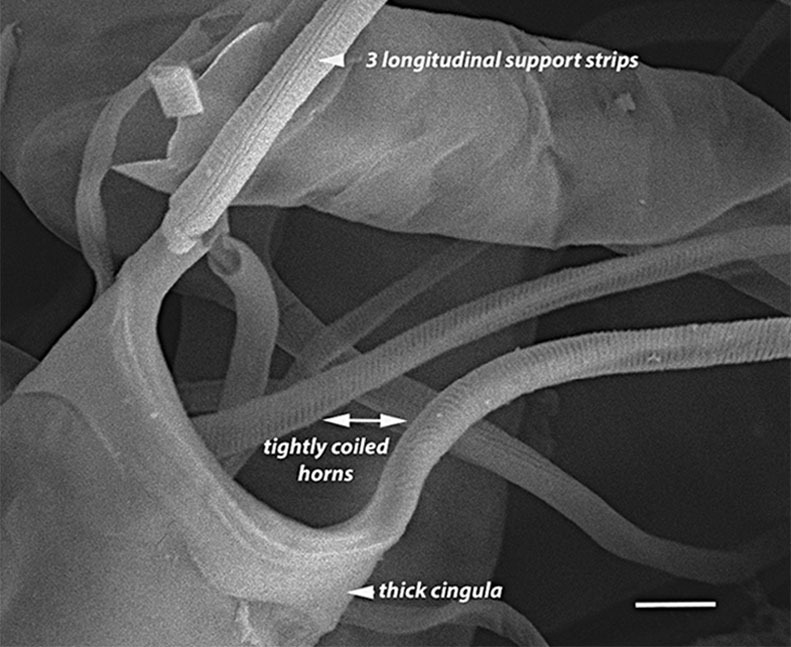
*Attheya longicornis*, girdle view. SEM. Scale bar: 1 µm.

**Figure 7a.**
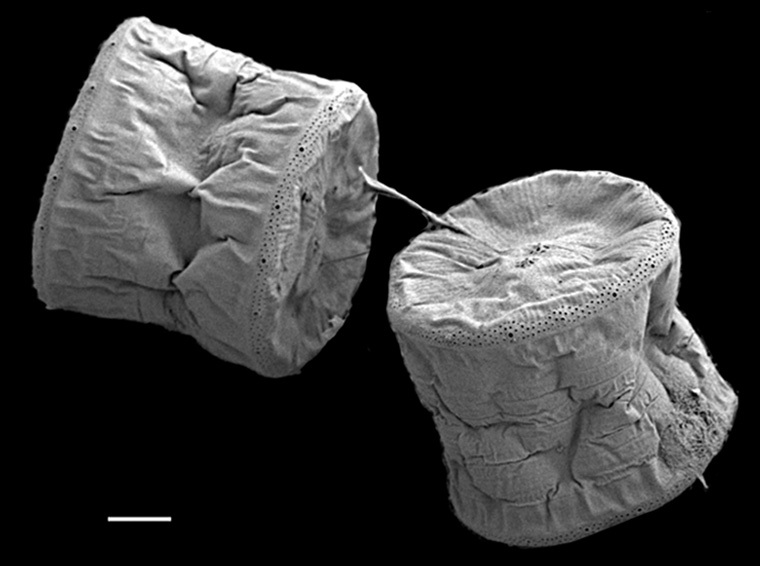
Bacterosira cf. constricta, external girdle and valve views. SEM. Scale bar: 5 µm

**Figure 7b.**
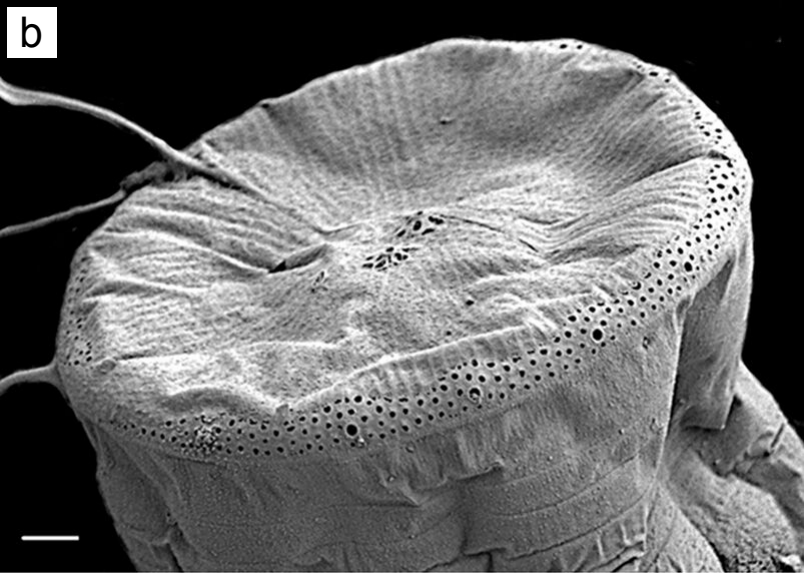
Bacterosira cf. constricta, external valve and girdle views. SEM. Scale bar: 2 µm.

**Figure 9a.**
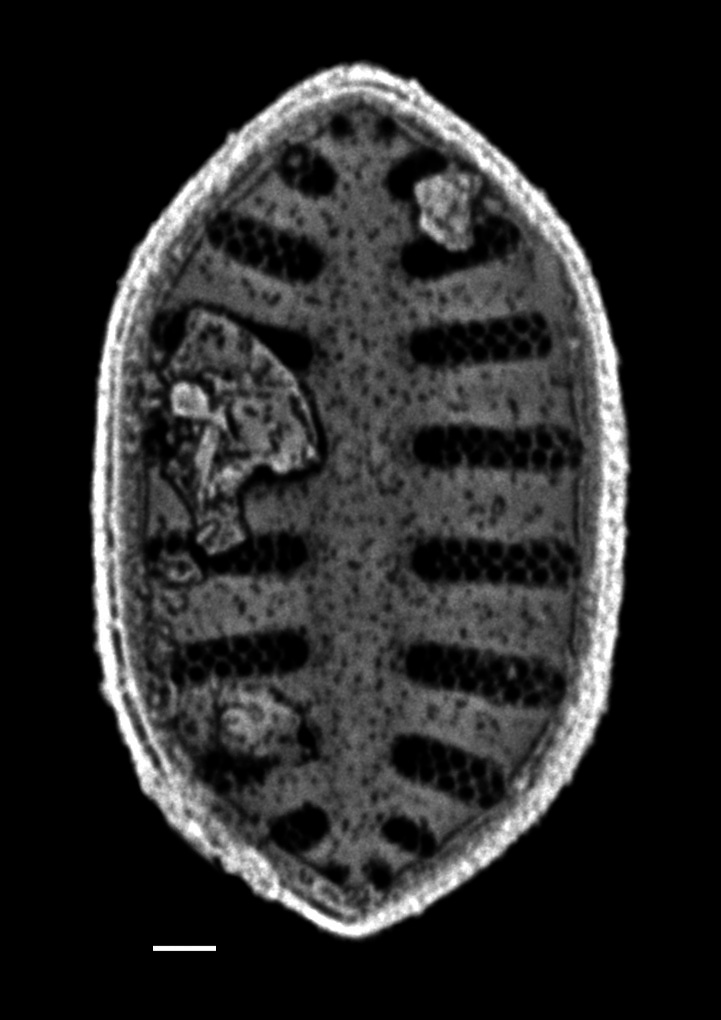
Cocconeis costata var. hexagona, SV. SEM. Scale bar: 1 µm;

**Figure 9b.**
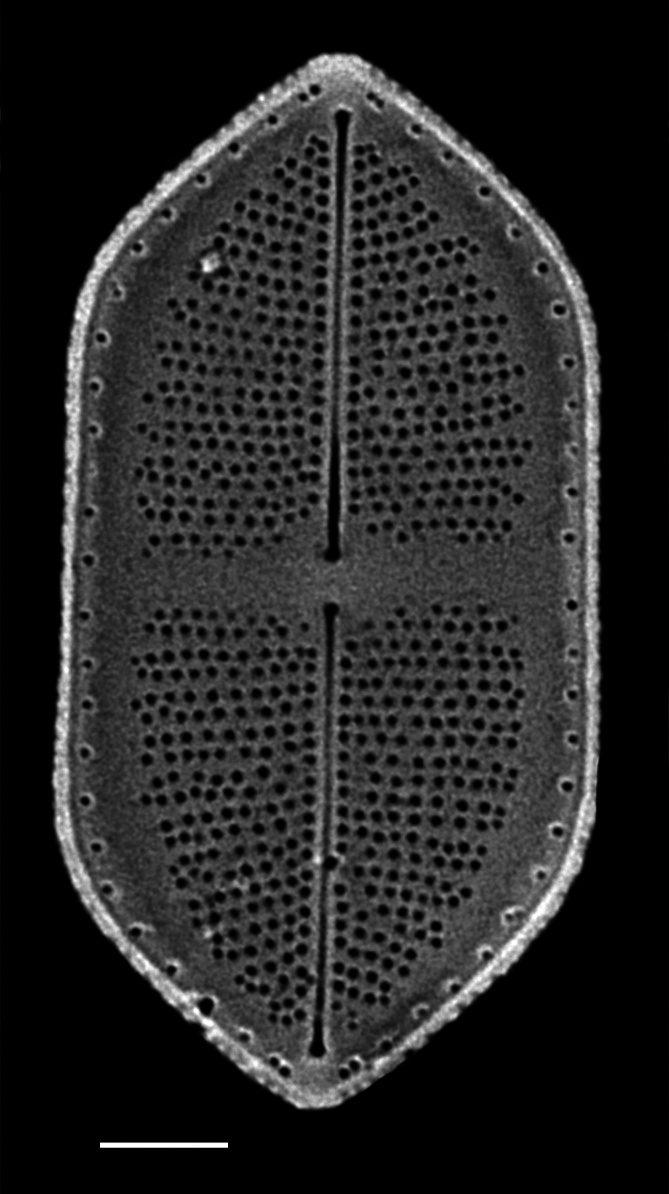
Cocconeis costata var. hexagona, interior SV. SEM. Scale bar: 2 µm.

**Figure 10a.**
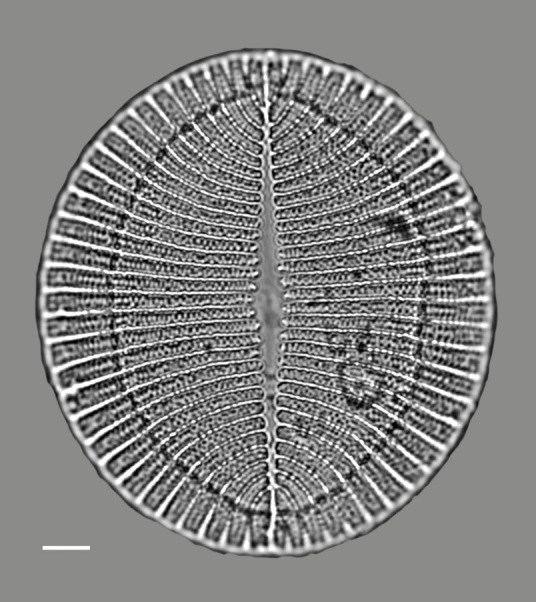
*Cocconeis kerguelensis*, SV. LM. Scale bar: 5 µm;

**Figure 10b.**
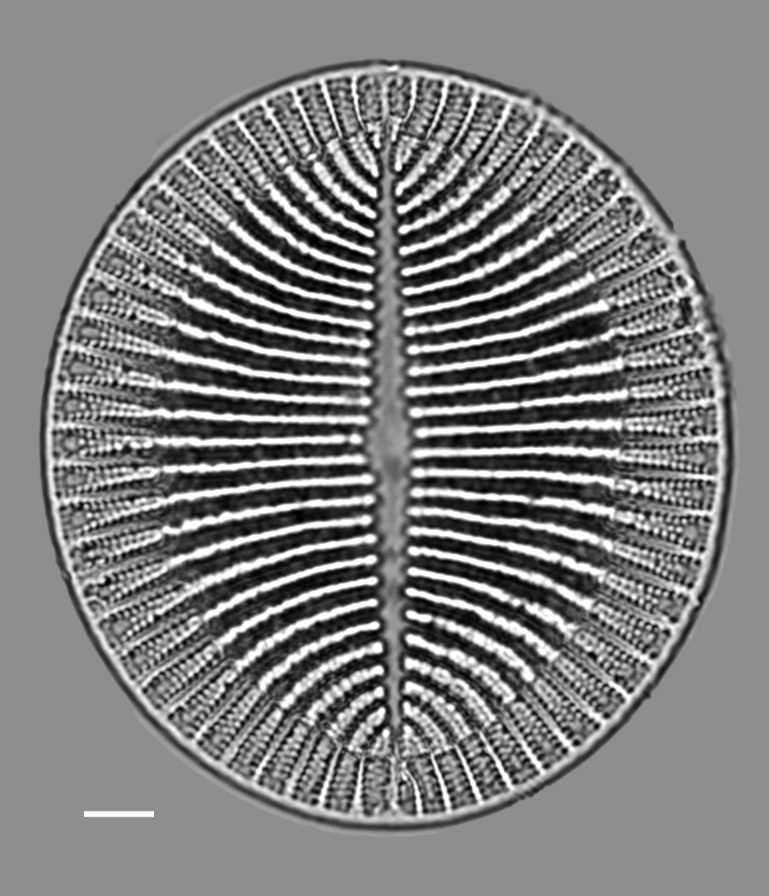
*Cocconeis kerguelensis*, SV. LM. Scale bar: 5 µm.

**Figure 11a.**
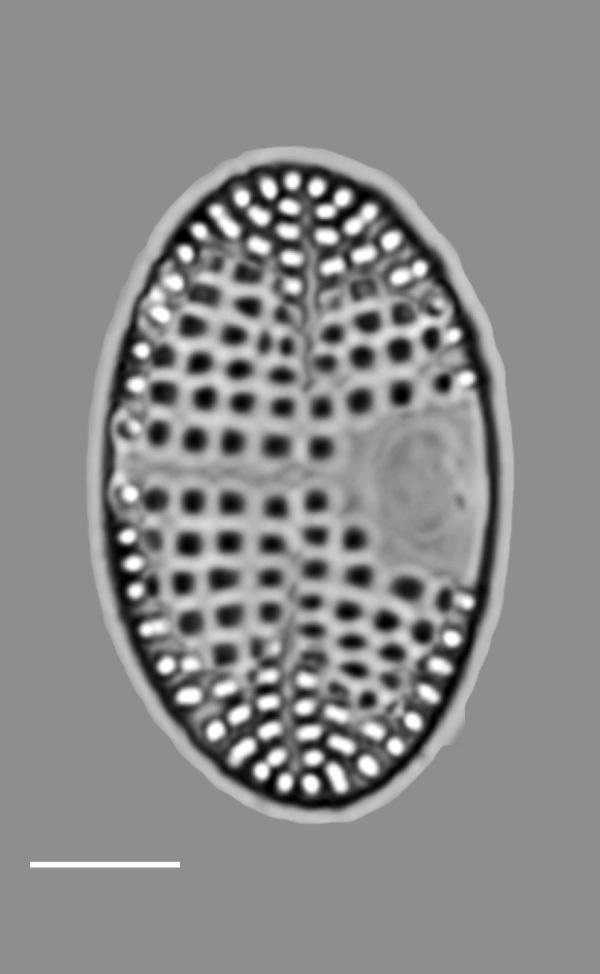
*Cocconeis notata*, SV. LM. Scale bar: 5 µm;

**Figure 11b.**
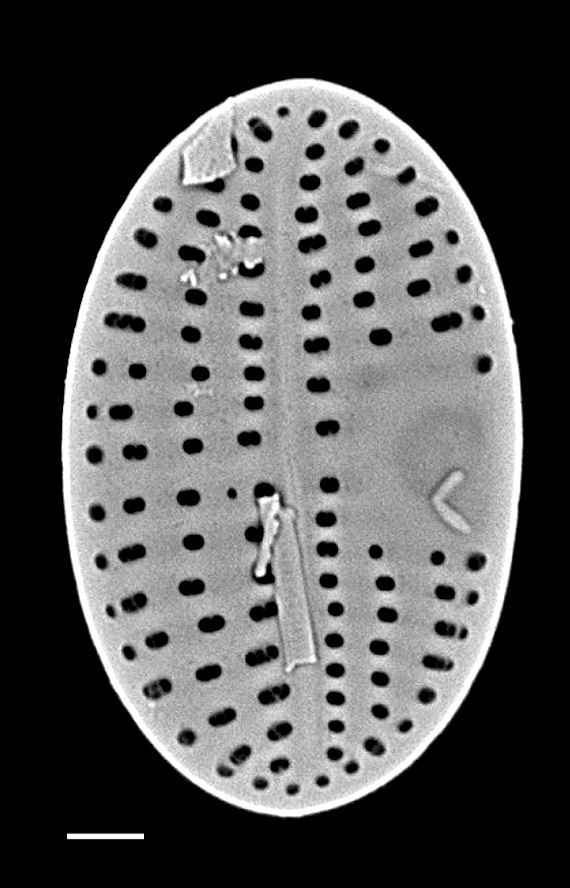
*Cocconeis notata*, SV. SEM. Scale bar: 2 µm;

**Figure 11c.**
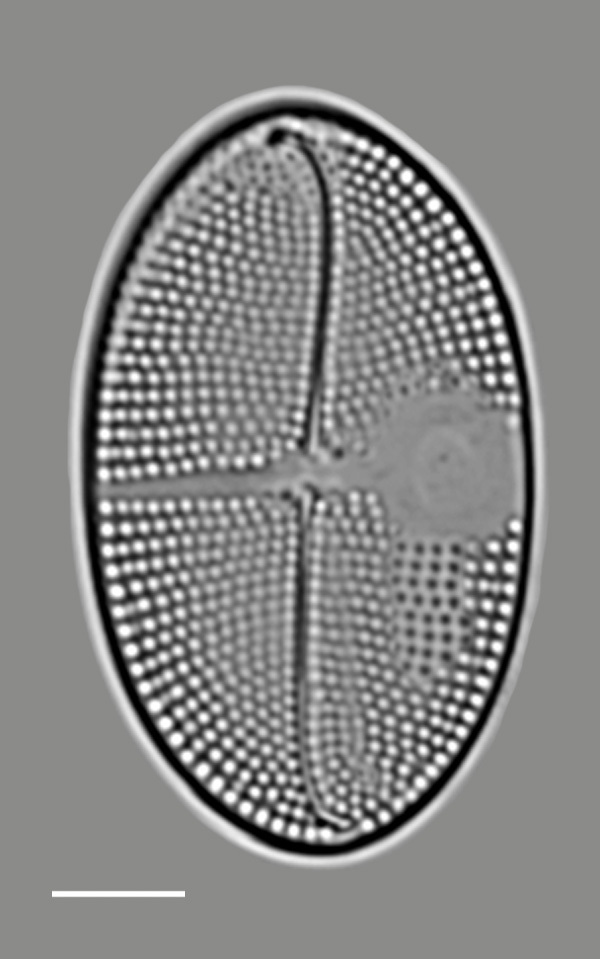
*Cocconeis notata*, RV. LM. Scale bar: 5 µm;

**Figure 11d.**
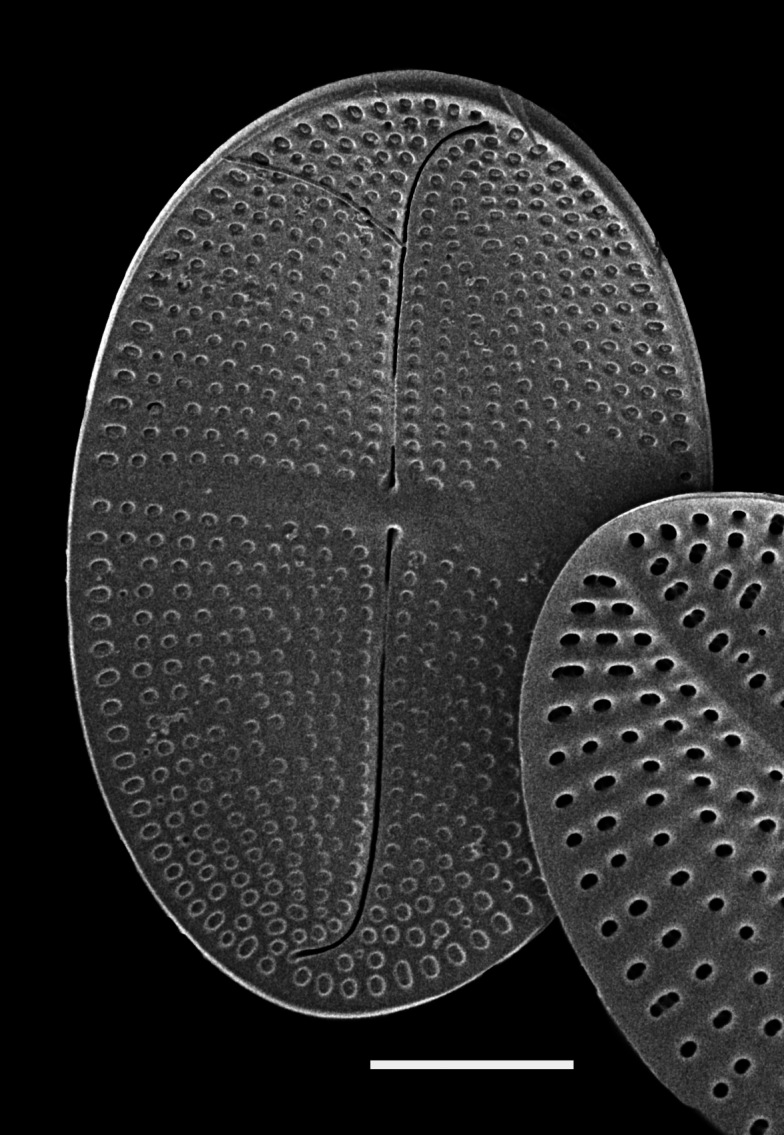
*Cocconeis notata*, RV. SEM. Scale bar: 5 µm.

**Figure 13a.**
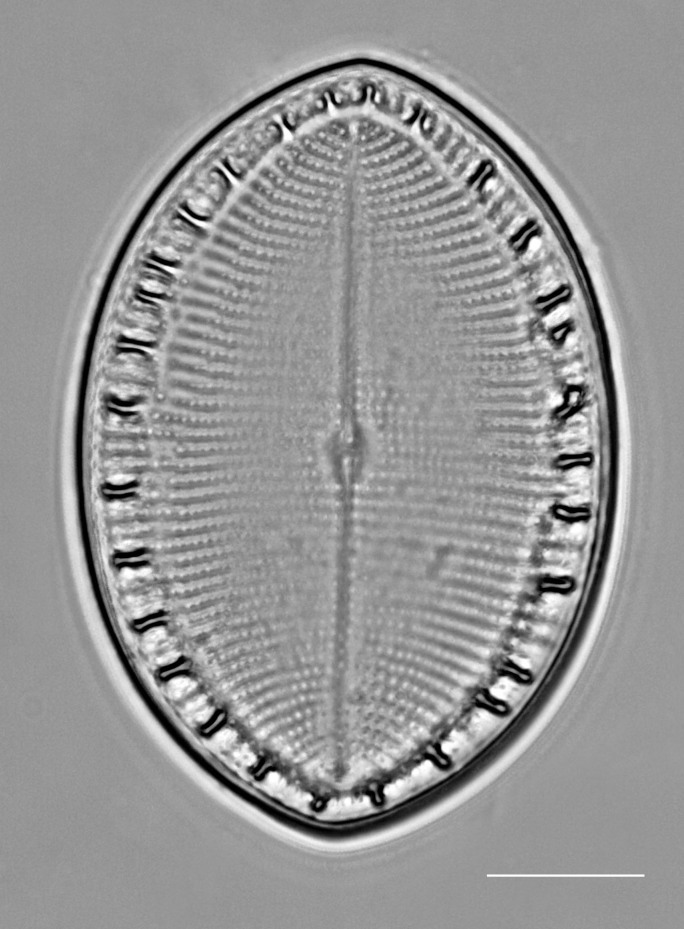
Cocconeis scutellum var. posidoniae, RV. SEM. Scale bar: 2 µm;

**Figure 13b.**
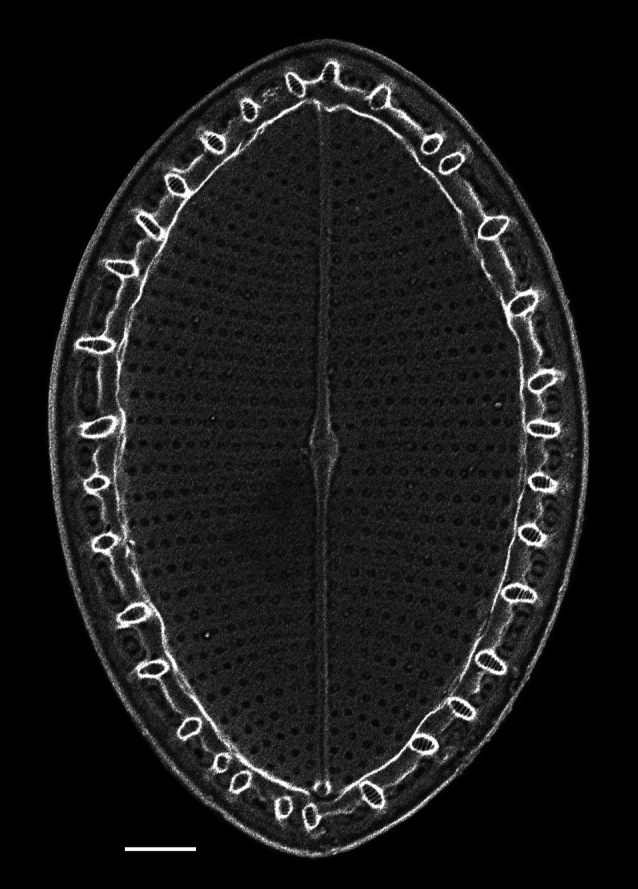
Cocconeis scutellum var. posidoniae, RV. Arrows indicate interfimbial space and papilla on each fimbria. SEM. Scale bar: 1 µm;

**Figure 13c.**
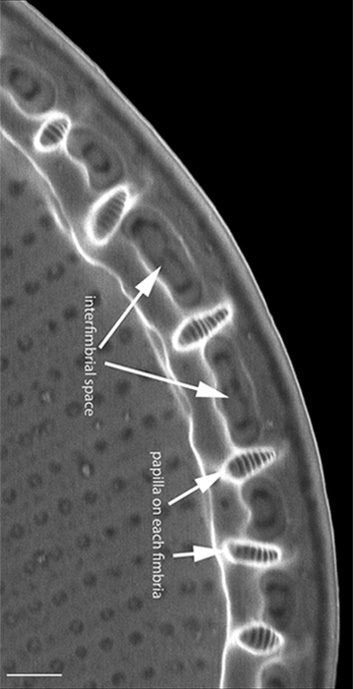
Cocconeis scutellum var. posidoniae, RV. SEM. Scale bar:10 µm.

**Figure 14a.**
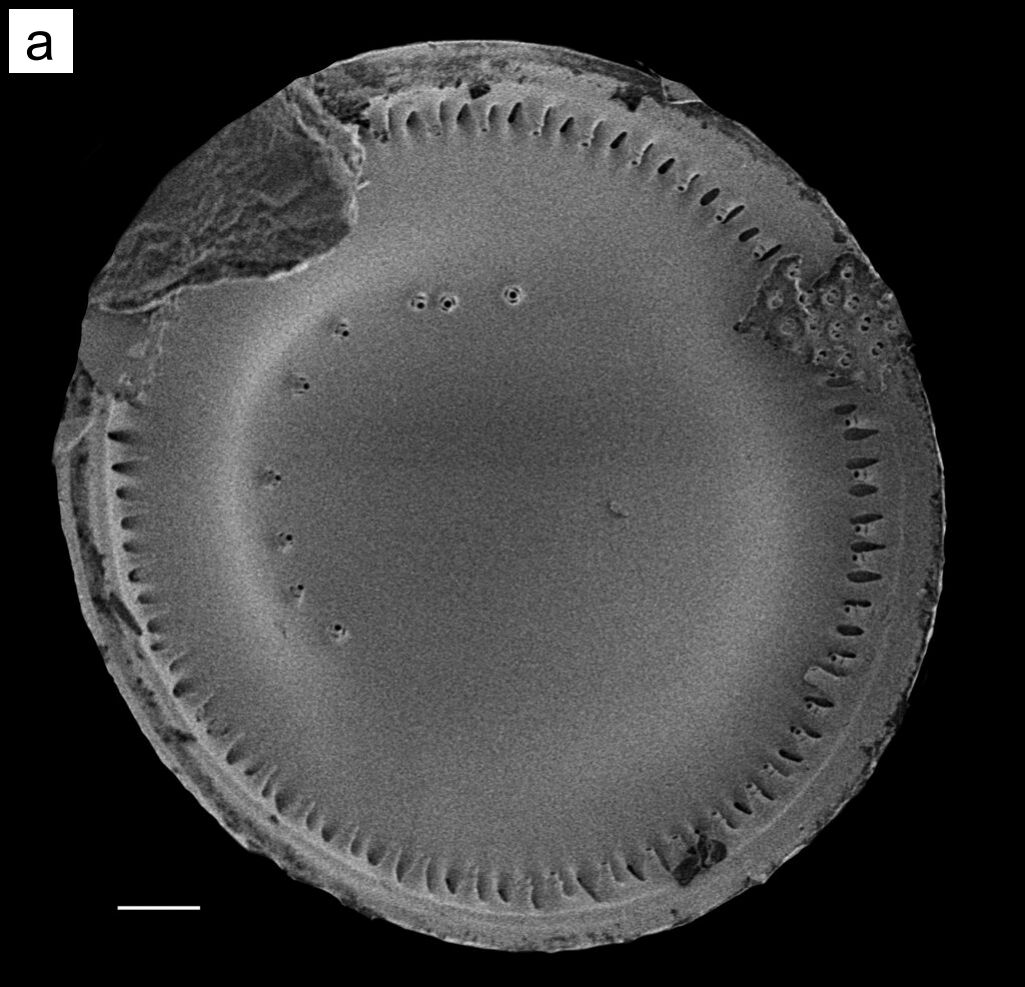
*Cyclotella baltica*, interior valve view. SEM. Scale bar: 2.5 µm;

**Figure 14b.**
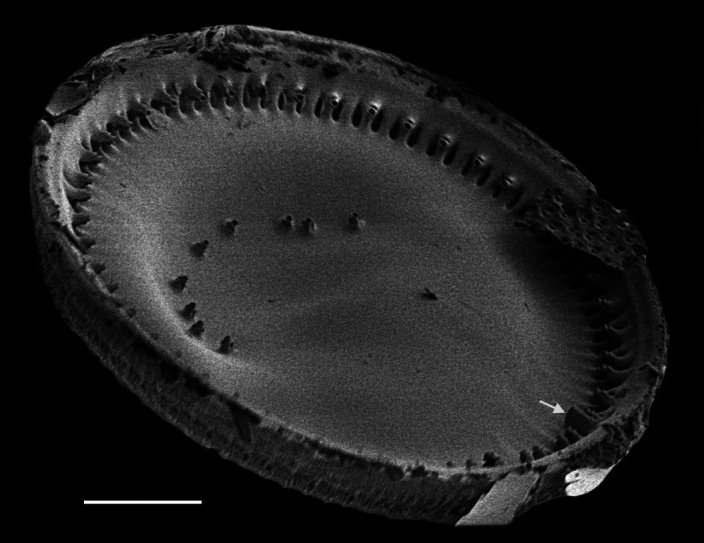
*Cyclotella baltica*, interior valve view. Arrow indicates location of the rimoportula. SEM. Scale bar: 5 µm.

**Figure 18a.**
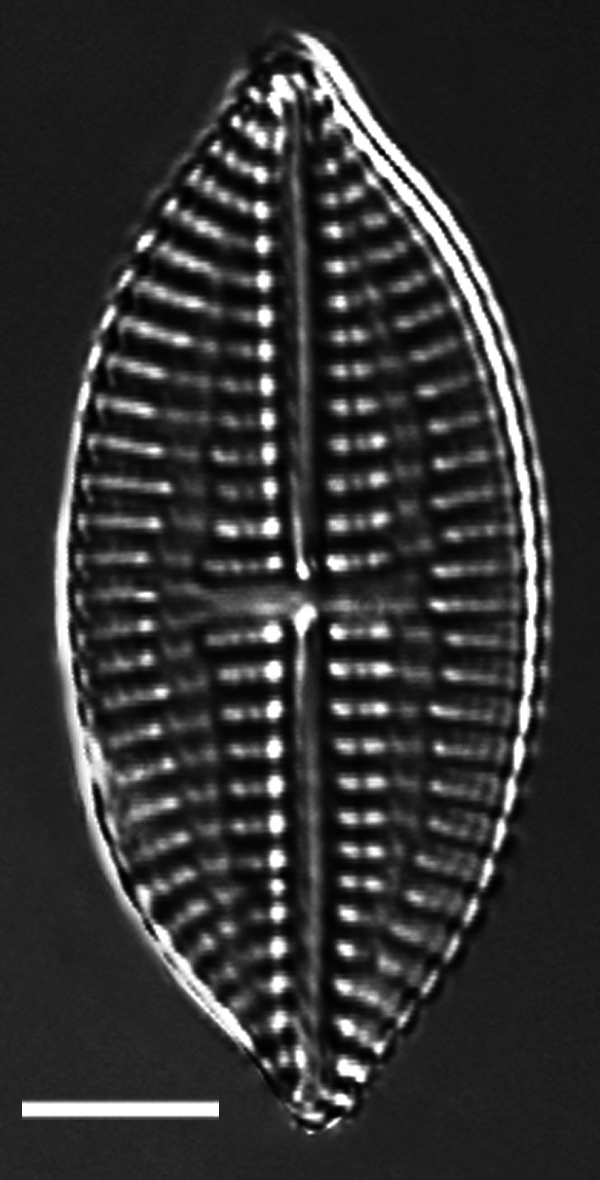
*Fogedia krammeri*, exterior valve view. LM. Scale bar: 5 µm;

**Figure 18b.**
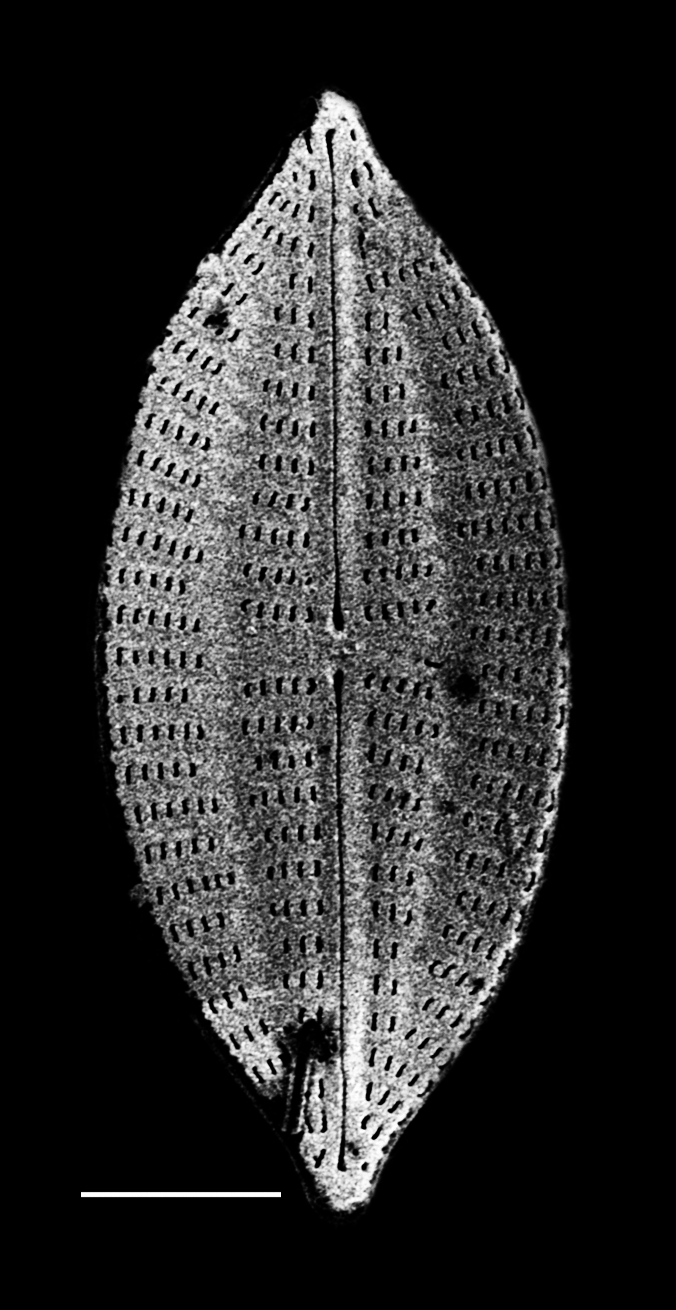
*Fogedia krammeri*, exterior valve view. SEM. Scale bar: 5 µm.

**Figure 19a.**
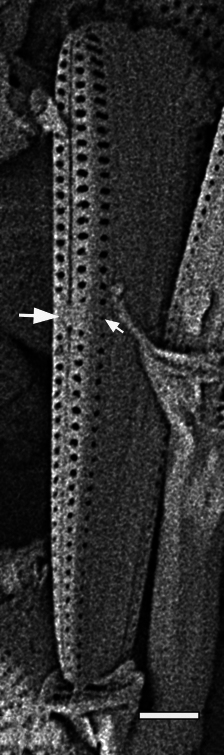
*Gomphonemopsis pseudoexigua*, exterior valve view. Large arrow indicates small rectangular central area that reaches the valve margin and small arrow shows the small stria. SEM. Scale bar: 2 µm;

**Figure 19b.**
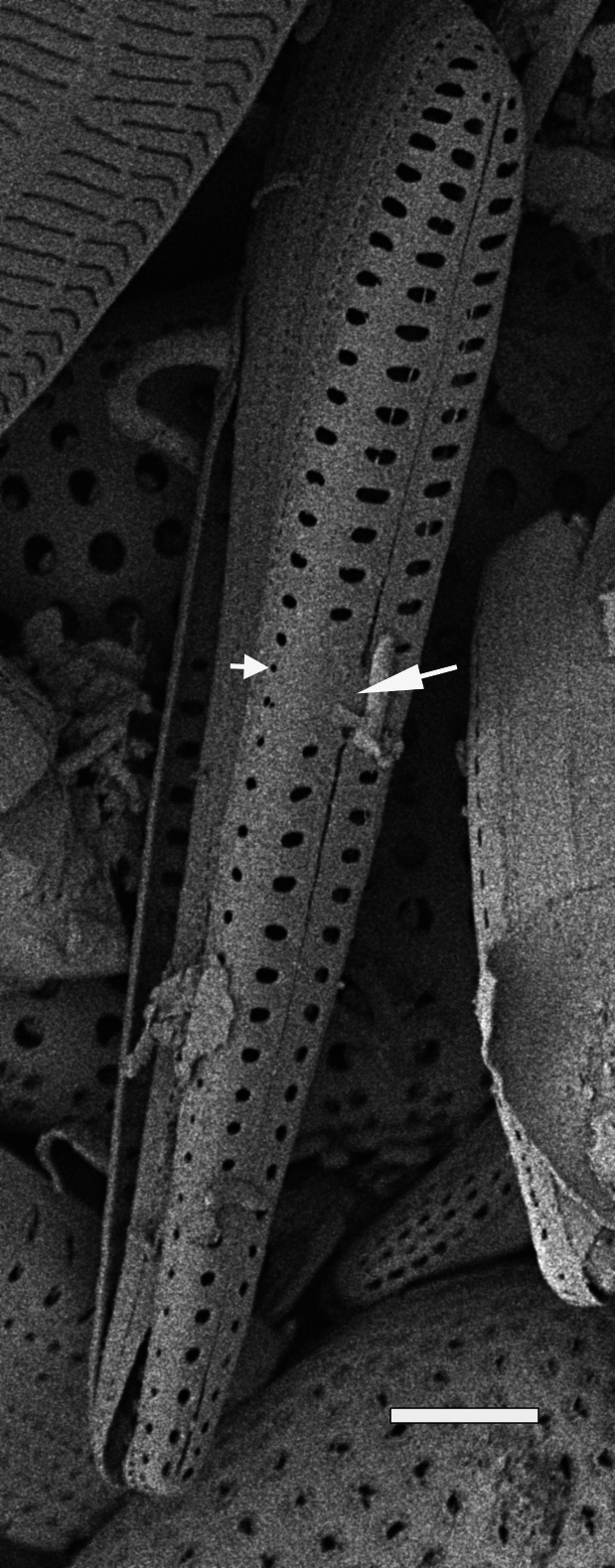
*Gomphonemopsis pseudoexigua*, exterior valve view. Large arrow indicates small rectangular central area that reaches the valve margin and small arrow shows the small stria. SEM. Scale bar: 2.5 µm.

**Figure 20a.**
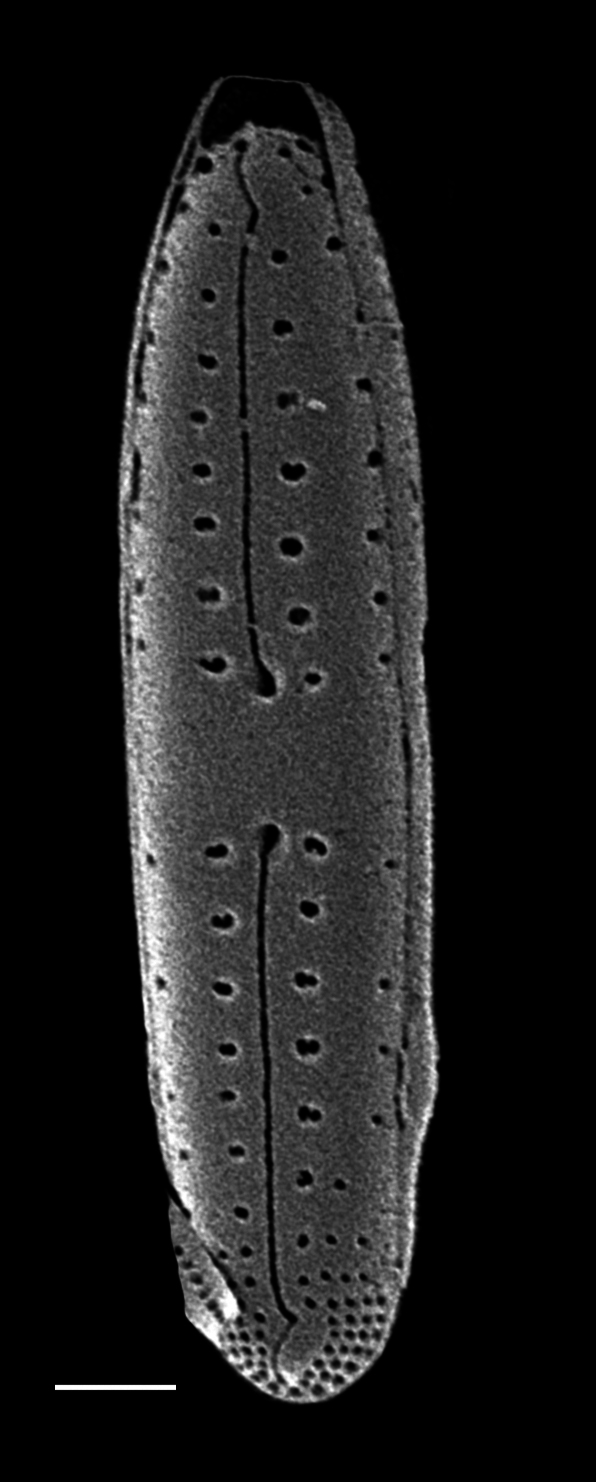
*Gomphoseptatum aestuaril*, exterior valve view. SEM. Scale bar: 1 µm;

**Figure 20b.**
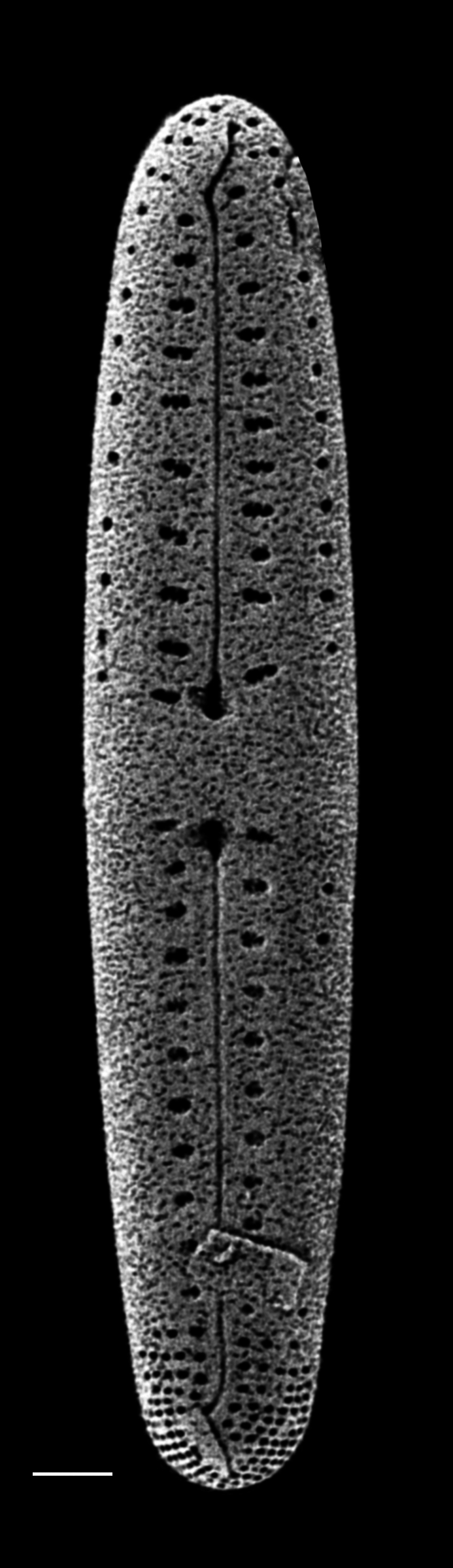
*Gomphoseptatum aestuaril*, exterior valve view. SEM. Scale bar: 1 µm;

**Figure 20c.**
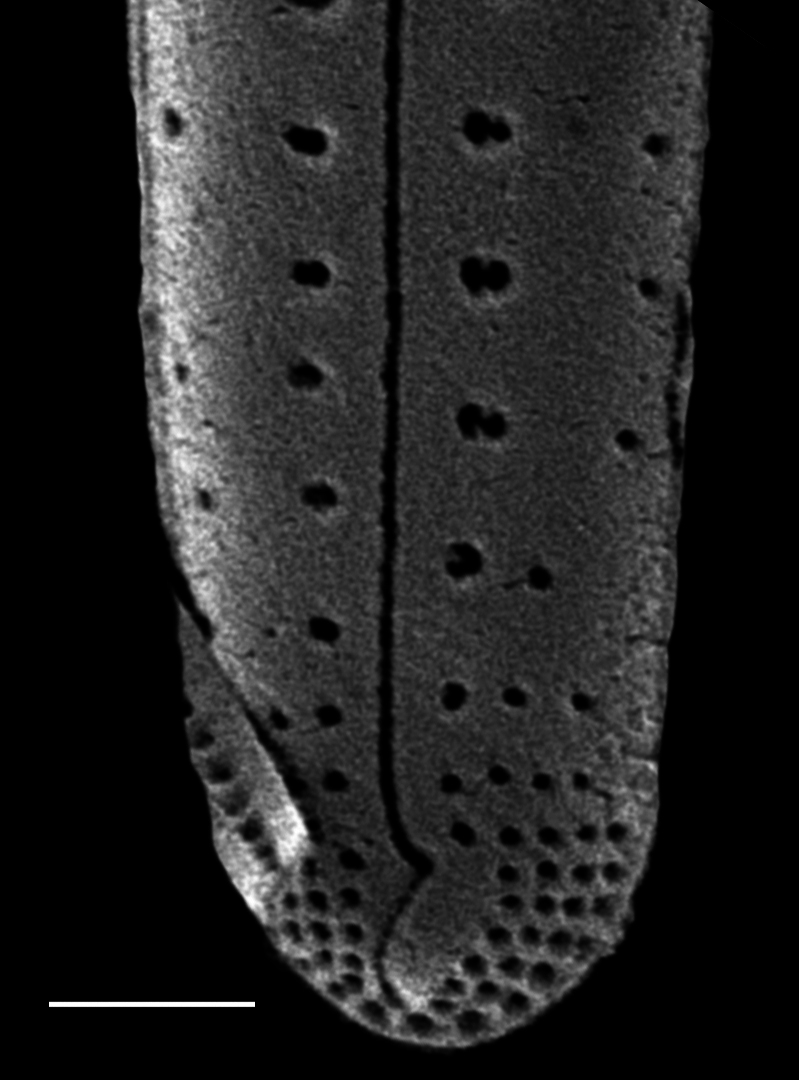
*Gomphoseptatum aestuaril*, exterior valve view, foot pole. SEM. Scale bar: 1 µm.

**Figure 21a.**
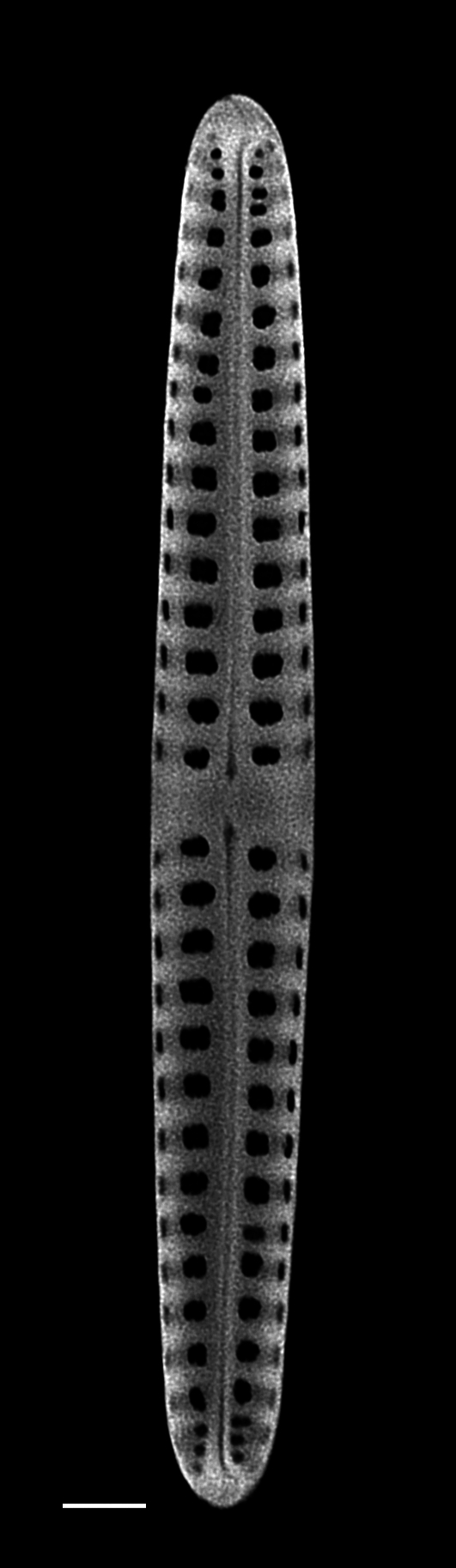
*Gomphoseptatum pseudoseptatum*, exterior valve view. SEM. Scale bar: 2 µm;

**Figure 21b.**
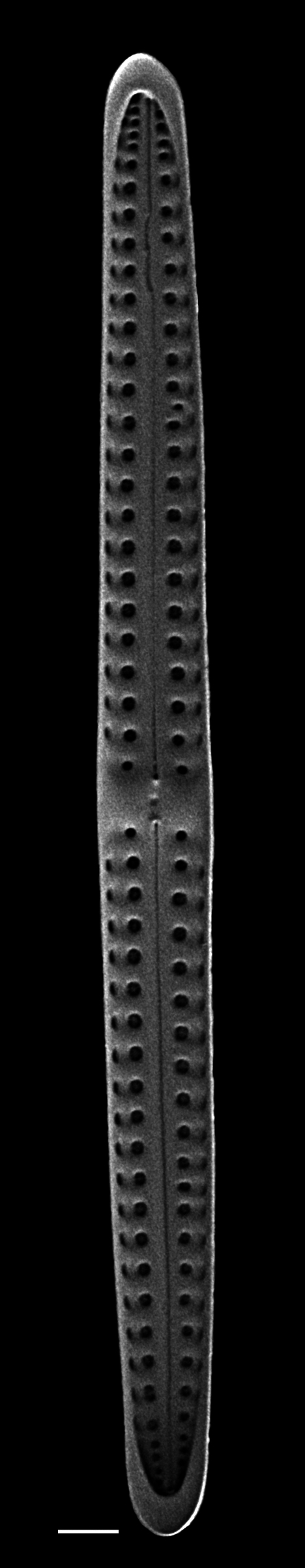
*Gomphoseptatum pseudoseptatum*, interior valve view. SEM. Scale bar: 1 µm;

**Figure 21c.**
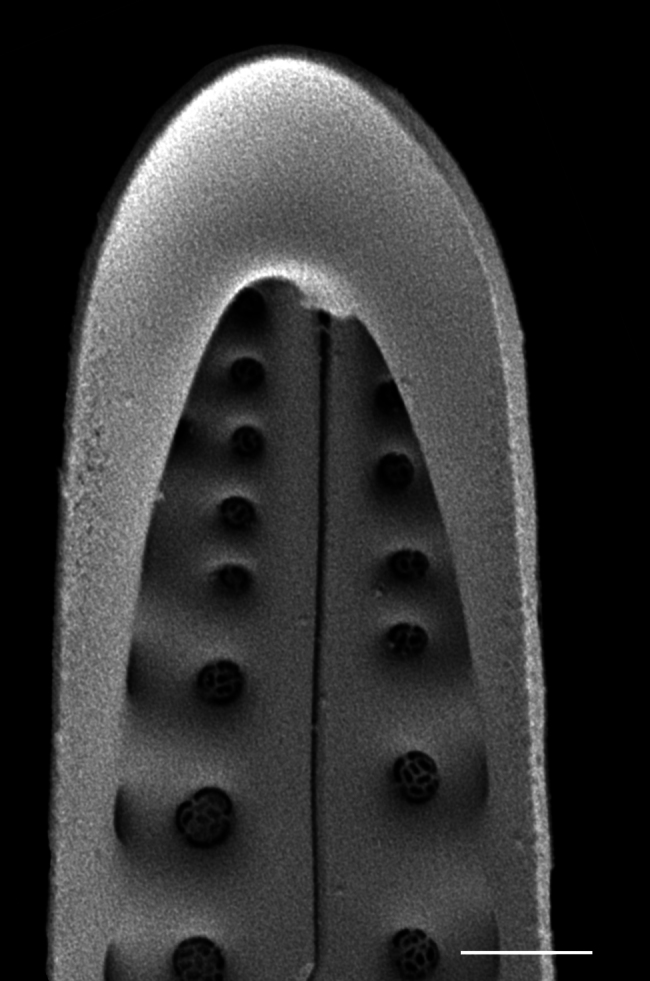
*Gomphoseptatum pseudoseptatum*, interior valve view. SEM. Scale bar: 1 µm.

**Figure 22a.**
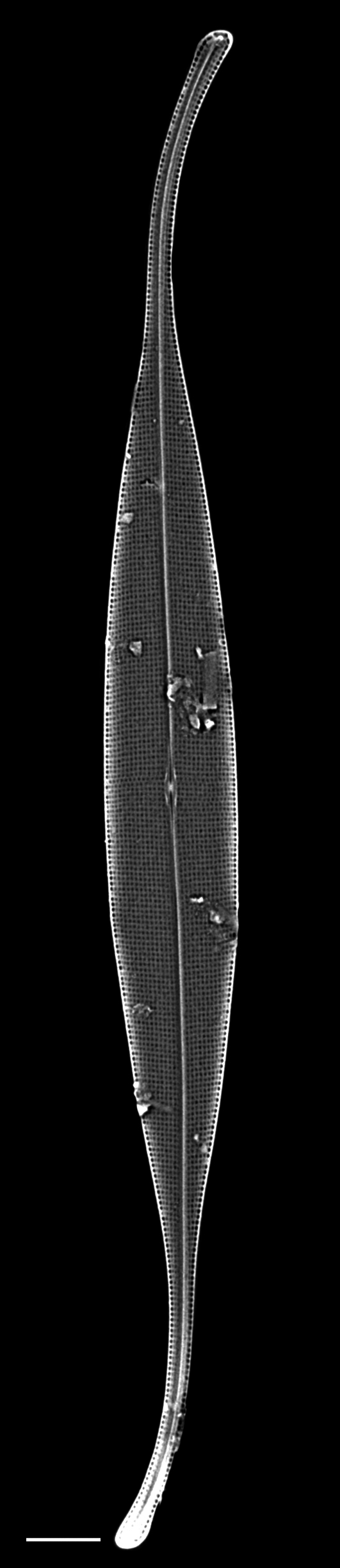
*Gyrosigma arcuatum*, exterior valve view. SEM. Scale bar: 5 µm;

**Figure 22b.**
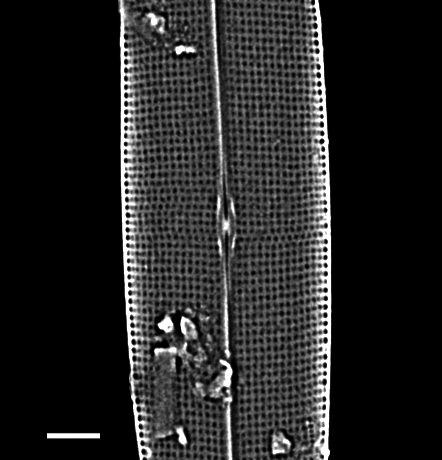
*Gyrosigma arcuatum*, central area. Exterior valve view. SEM. Scale bar: 2.5 µm.

**Figure 23a.**
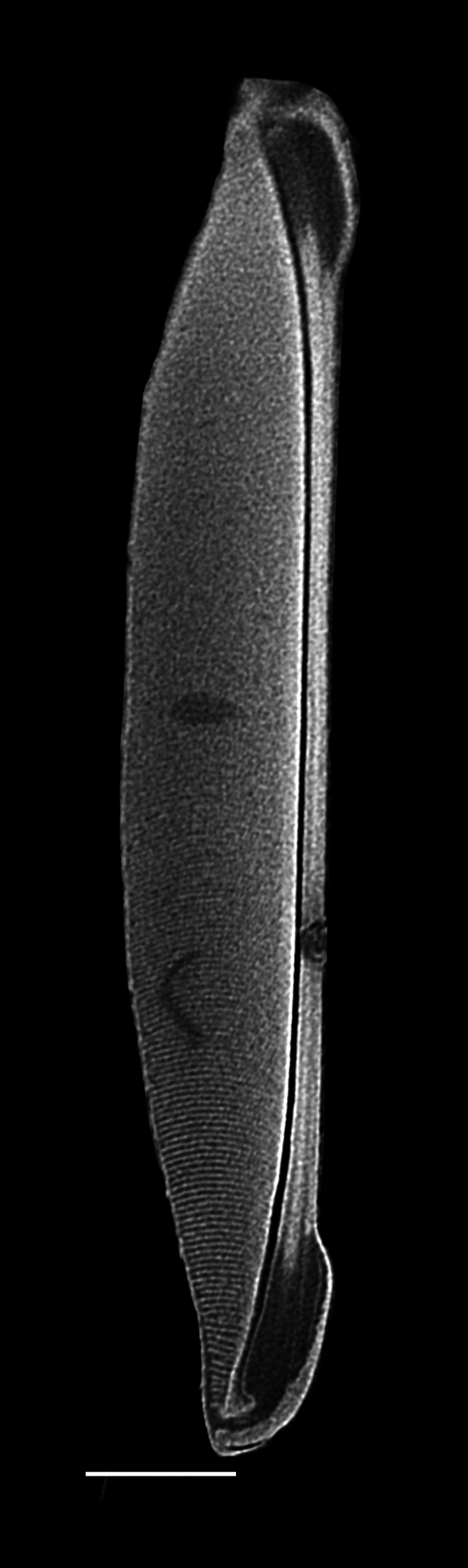
*Homoeocladia spathulatoides*, exterior valve view. SEM. Scale bar: 5 µm;

**Figure 23b.**
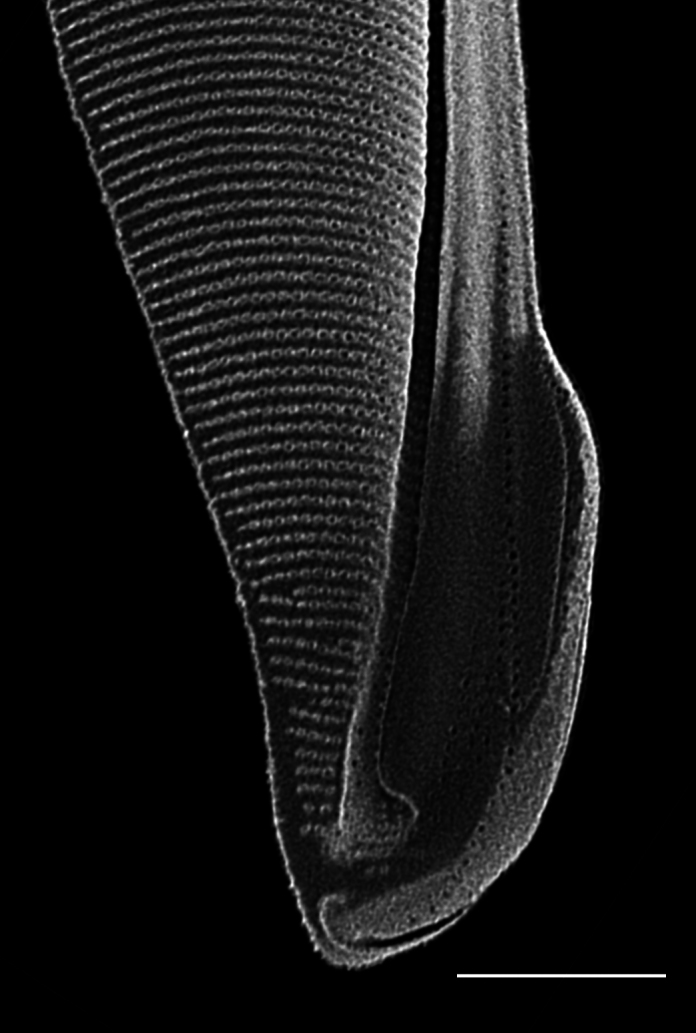
*Homoeocladia spathulatoides*, apex. Exterior valve view. SEM. Scale bar: 2.5 µm.

**Figure 24a.**
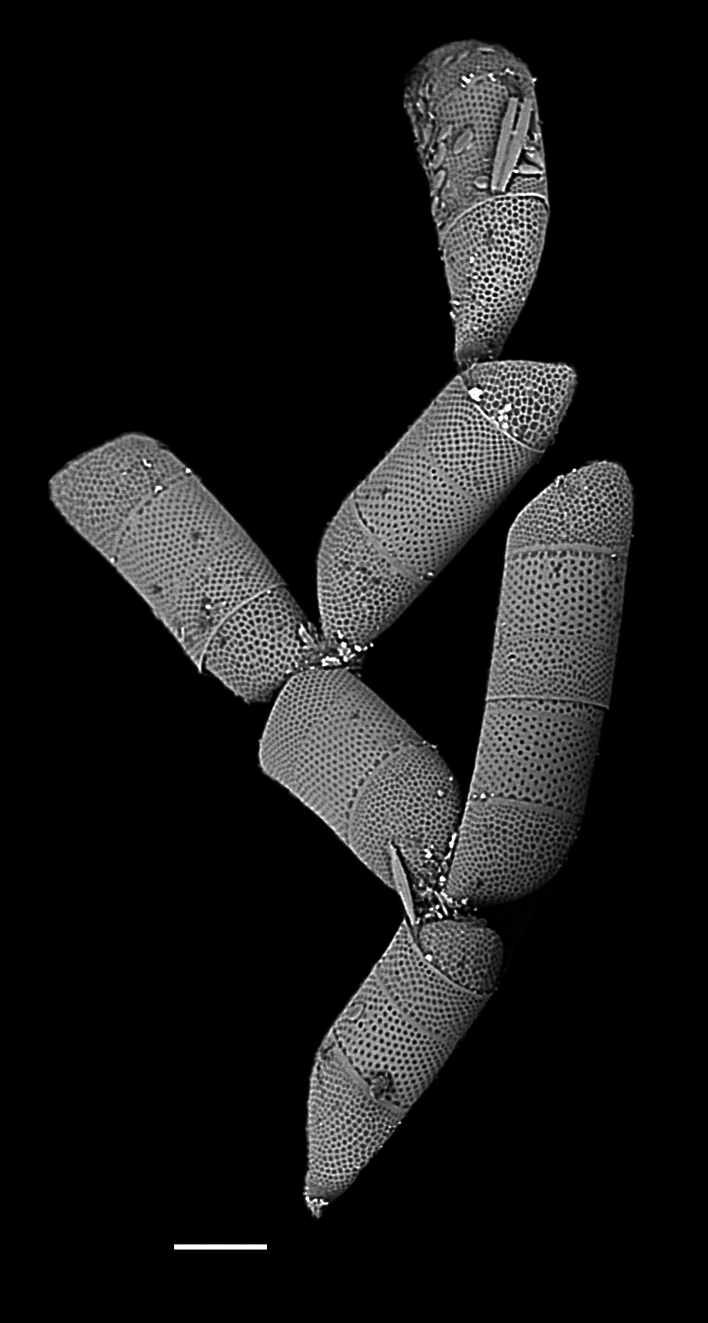
*Isthmia enervis*, girdle view. SEM. Scale bar: 50 µm;

**Figure 24b.**
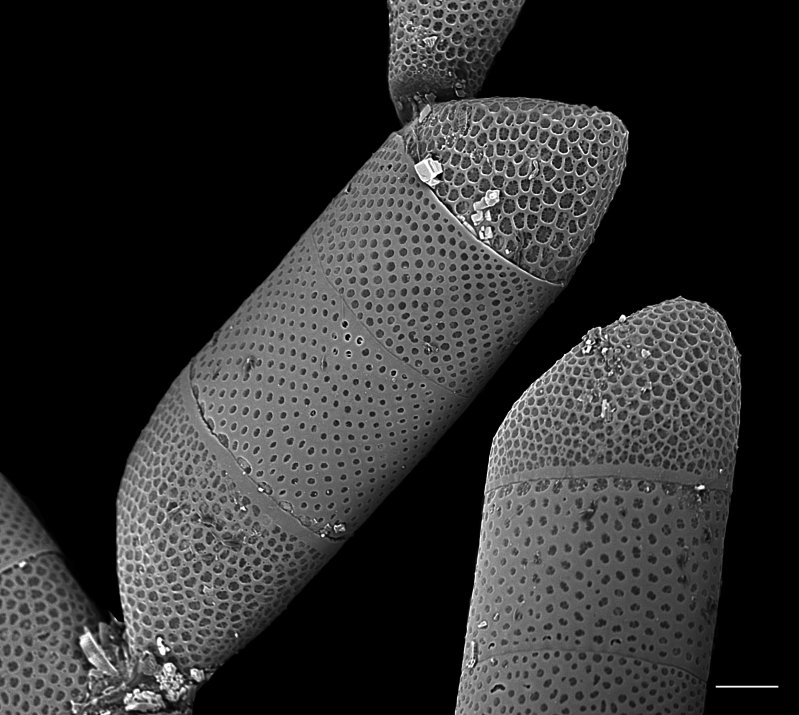
*Isthmia enervis*, girdle and valve views. SEM. Scale bar: 20 µm;

**Figure 24c.**
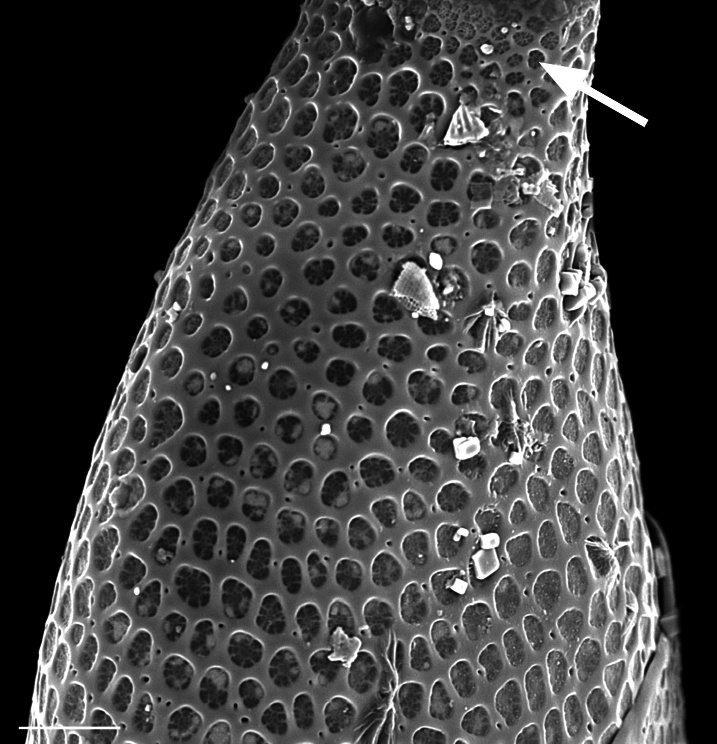
*Isthmia enervis*, girdle and valve views. Arrow indicates a gradual reduction in areolae size in the pseudocellus. SEM. Scale bar: 10 µm.

**Figure 26a.**
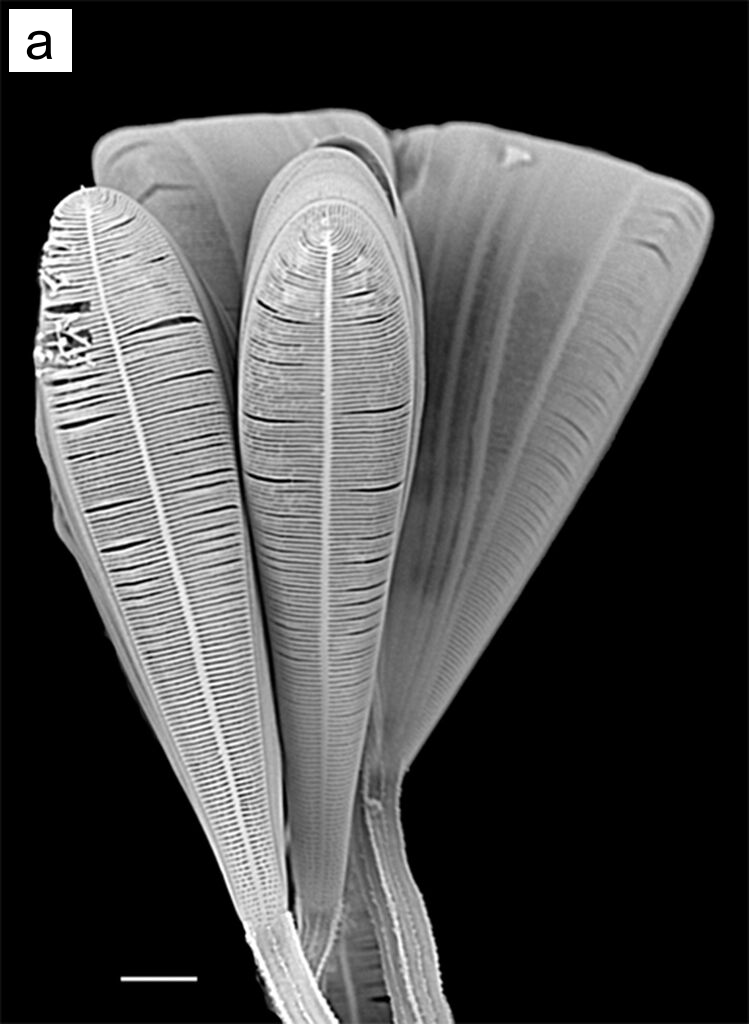
*Licmophora tincta*, exterior valve view. SEM. Scale bar: 5 µm;

**Figure 26b.**
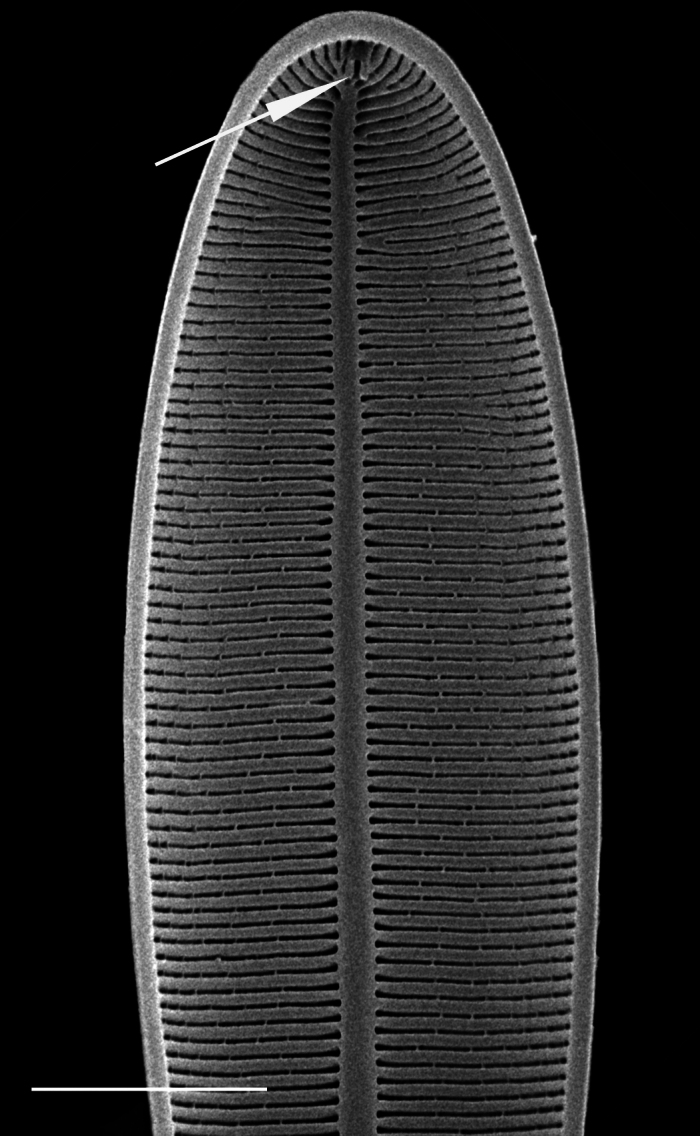
*Licmophora tincta*, interior valve view. Arrow indicates rimoportula. SEM. Scale bar: 5 µm.

**Figure 28a.**
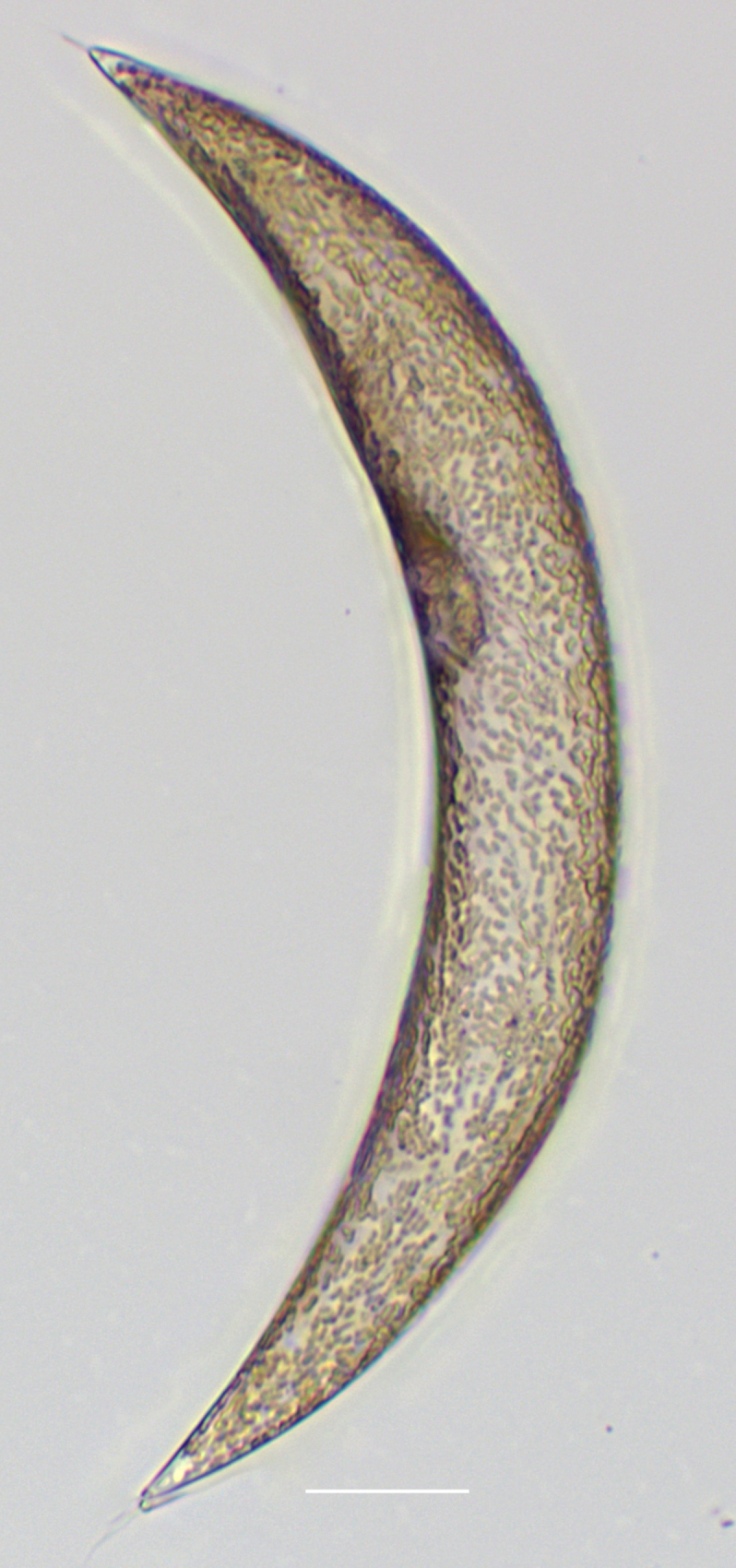
*Neocalyptrella robusta*, live, girdle view. LM. Scale bar: 50 µm;

**Figure 28b.**
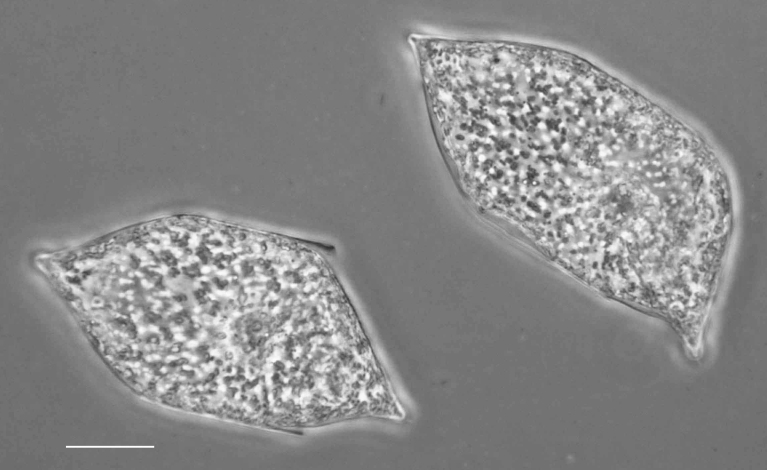
*Neocalyptrella robusta*, live, in culture. LM. Girdle view. Scale bar: 50 µm;

**Figure 28c.**
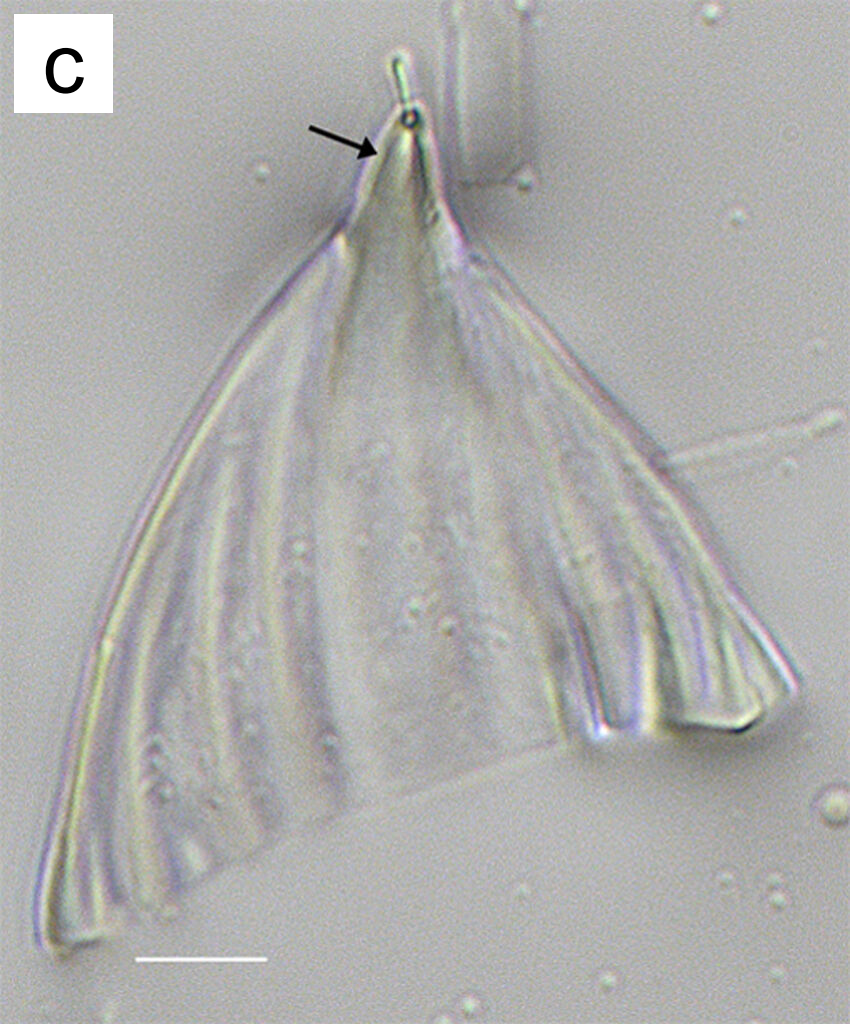
*Neocalyptrella robusta*, LM, girdle view. Arrow indicates calyptra. LM. Scale bar: 20 µm;

**Figure 28d.**
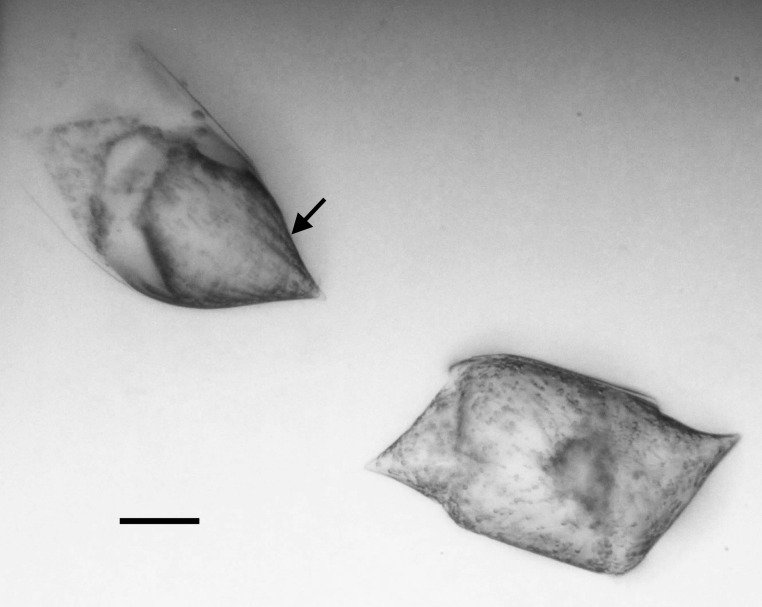
*Neocalyptrella robusta*, clones. Girdle view. Large arrow indicates longitudinal undulations converging on apex. LM. Scale bar: 50 µm;

**Figure 28e.**
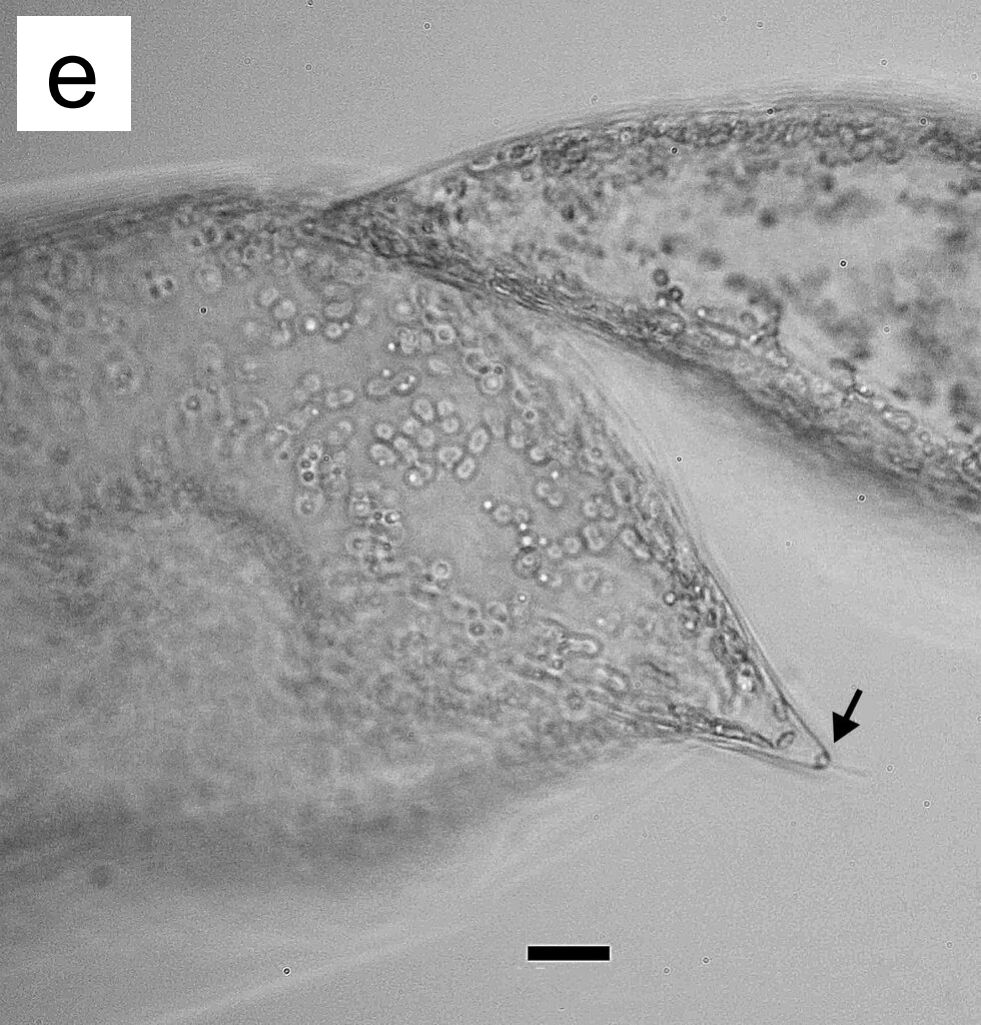
*Neocalyptrella robusta*, clones. Girdle view. Arrow indicates calyptra. LM. Scale bar: 10 µm.

**Figure 29a.**
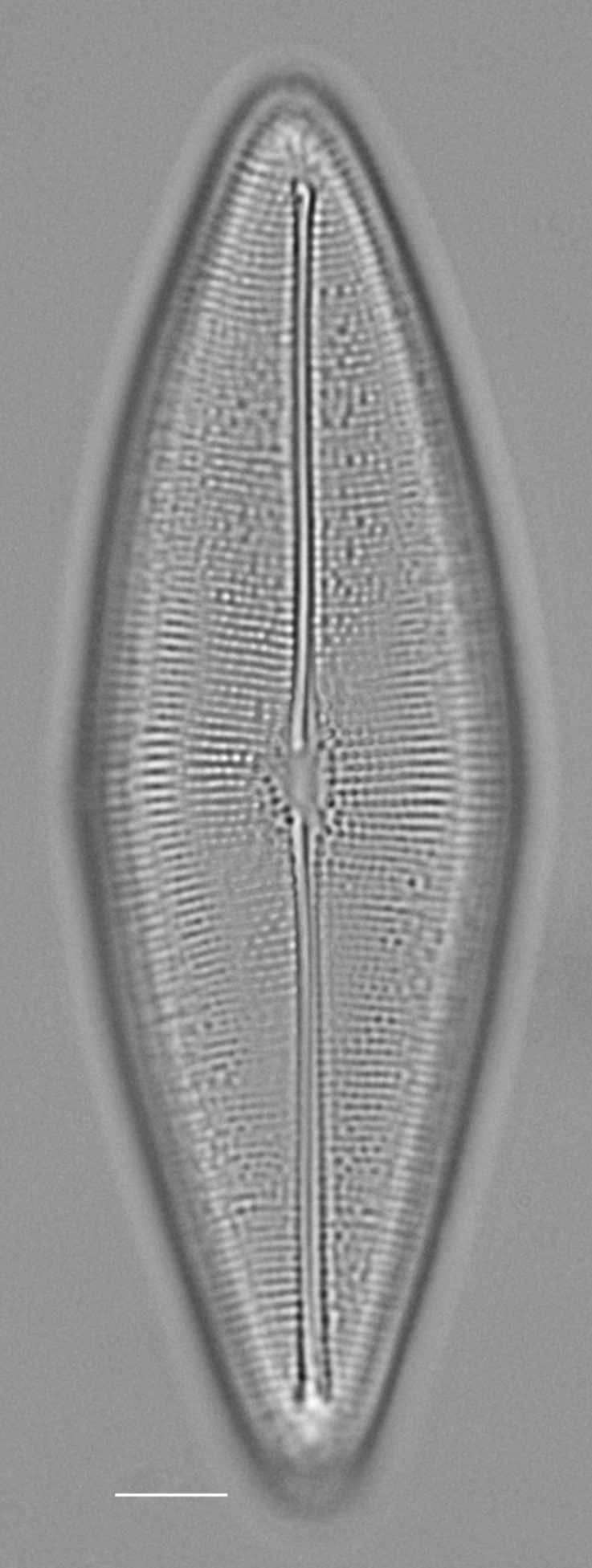
Parlibellus delognei f. ellipticus, LM exterior valve view. LM. Scale bar: 5 µm;

**Figure 29b.**
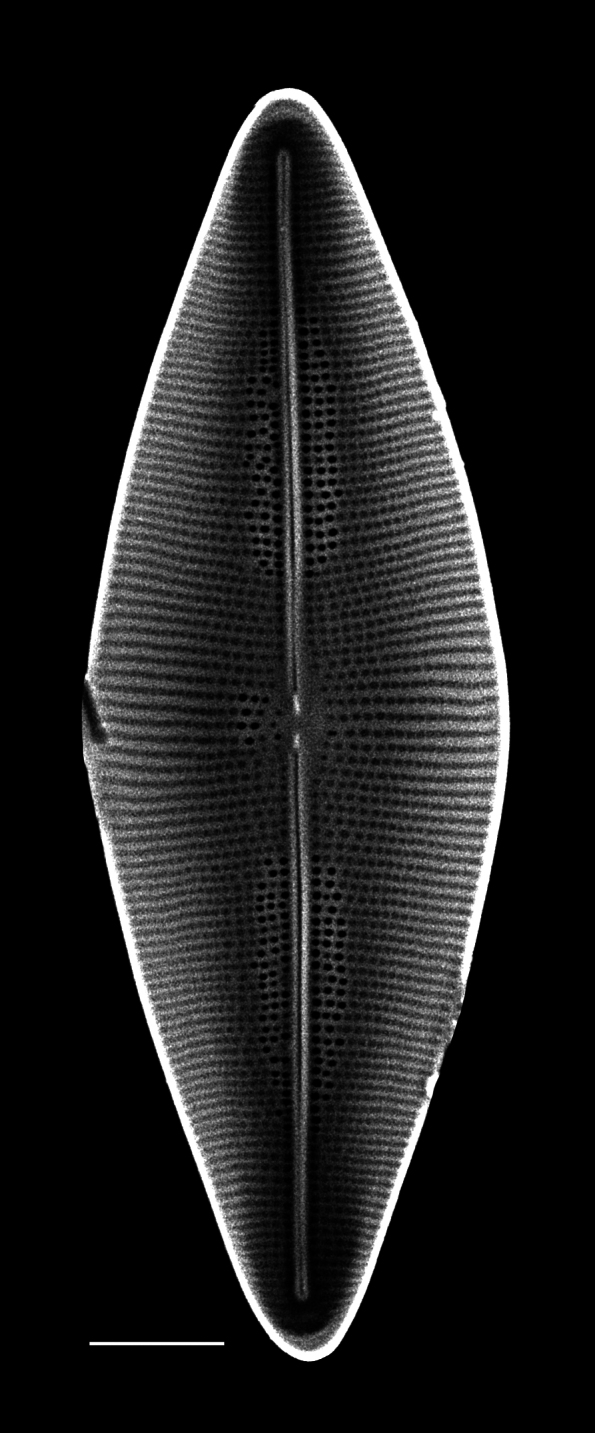
Parlibellus delognei f. ellipticus, interior valve view. SEM. Scale bar: 5 µm;

**Figure 29c.**
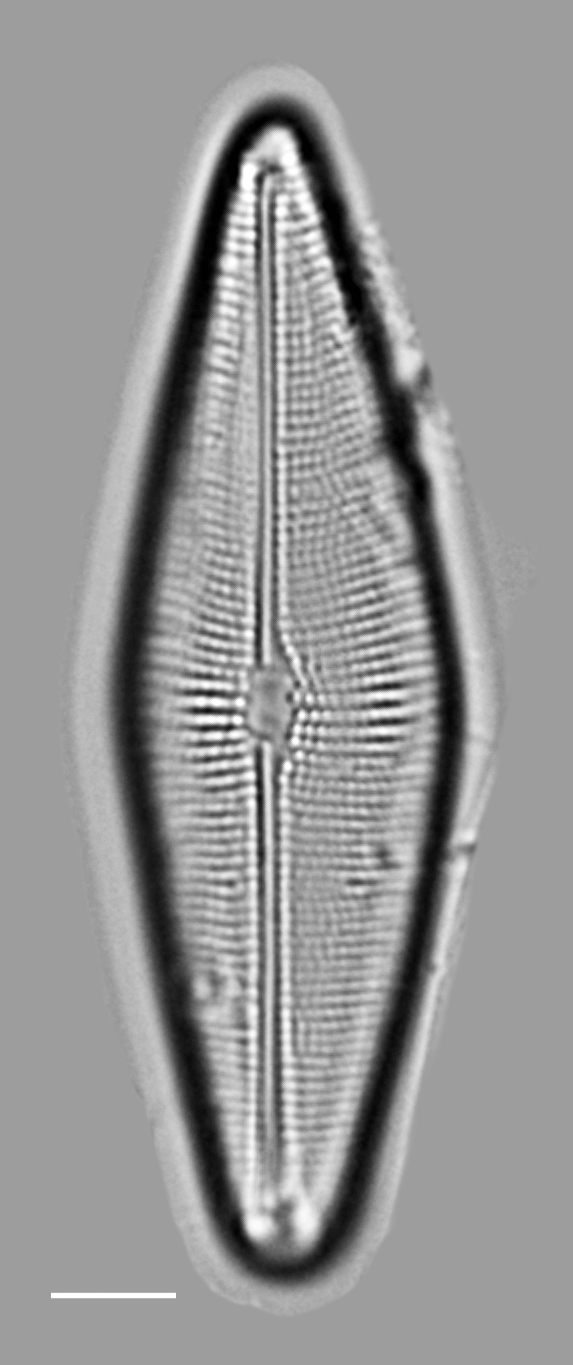
Parlibellus delognei f. ellipticus, LM, exterior valve view. LM. Scale bar: 5 µm.

**Figure 30a.**
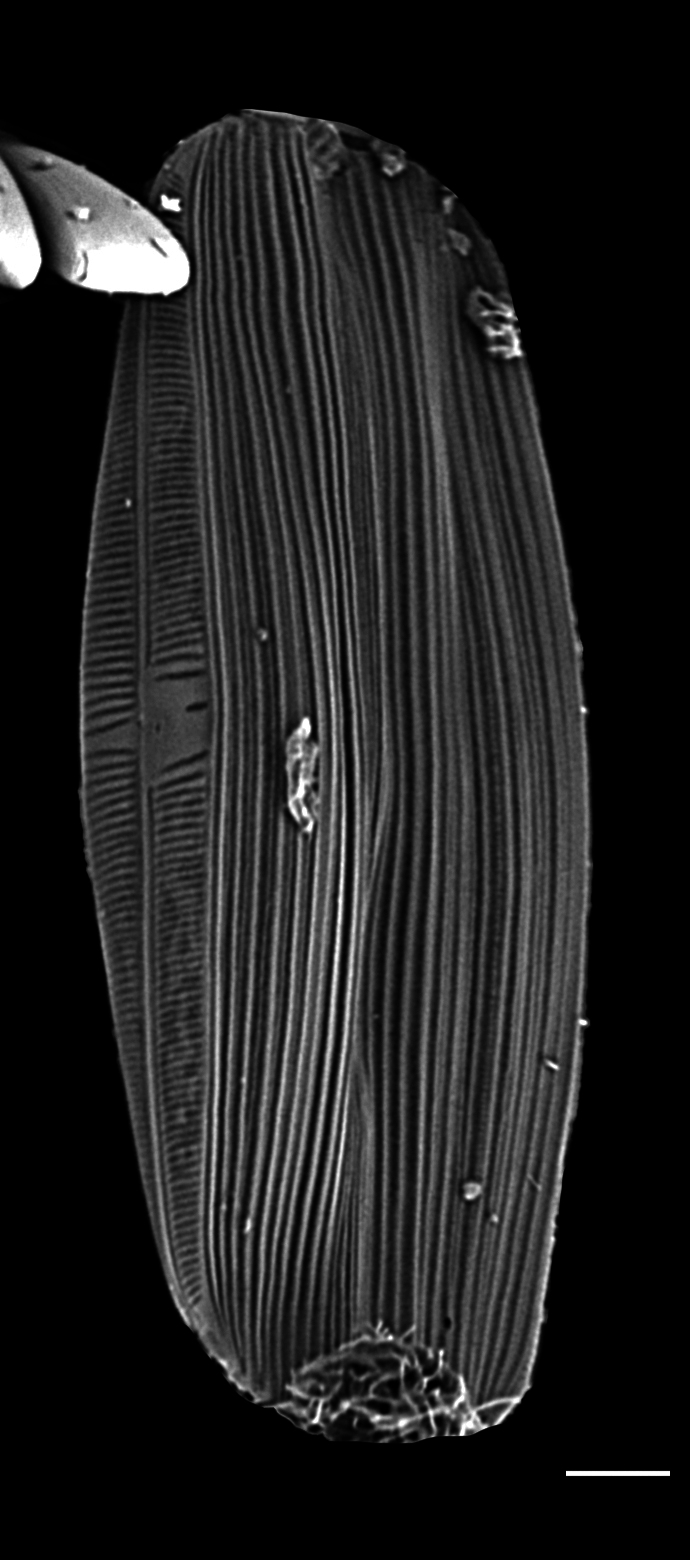
*Proschkinia complanatula*, girdle and valve views. SEM. Scale bar: 5 µm;

**Figure 30b.**
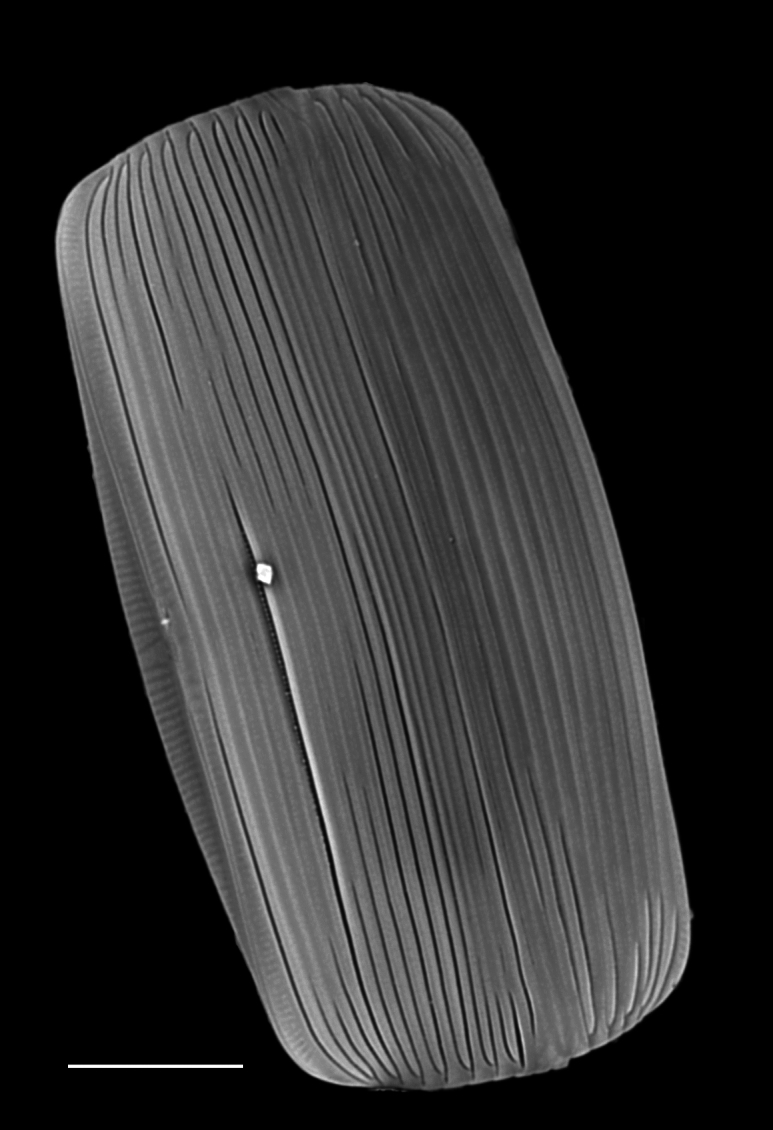
*Proschkinia complanatula*, girdle view. SEM. Scale bar: 10 µm.

**Figure 32a.**
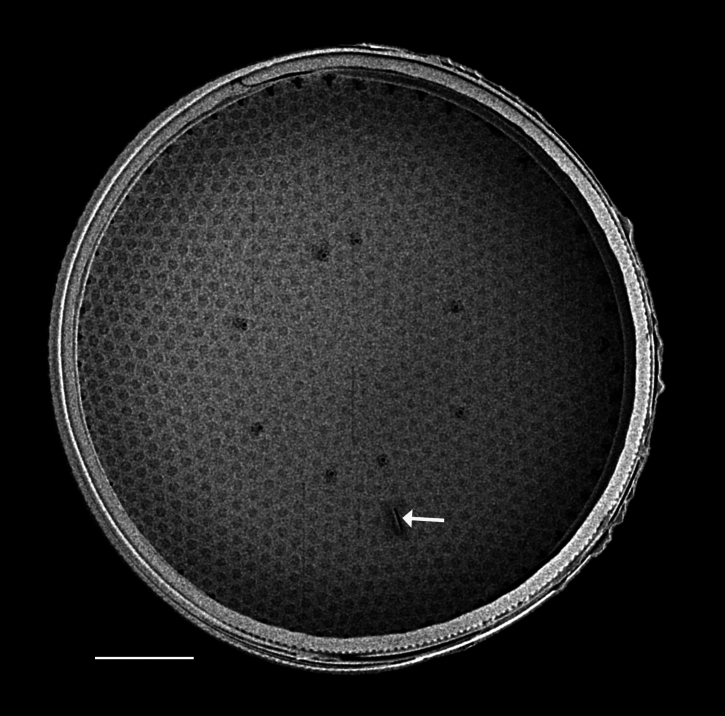
*Shionodiscus endoseriatus*, interior valve view. Arrow indicates rimoportula. SEM. Scale bar: 5 µm;

**Figure 32b.**
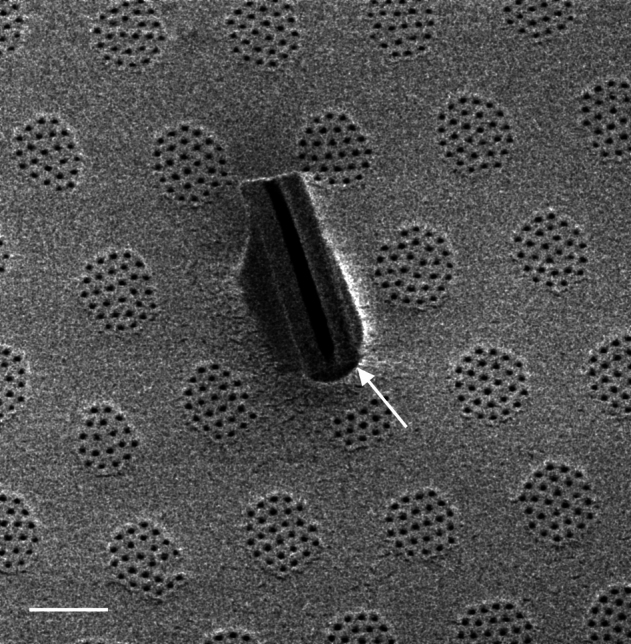
*Shionodiscus endoseriatus*, interior valve view. Arrow indicates rimoportula orientated radially, taking the place of an areola arranged in a radial row. SEM. Scale bar: 0.5 µm;

**Figure 32c.**
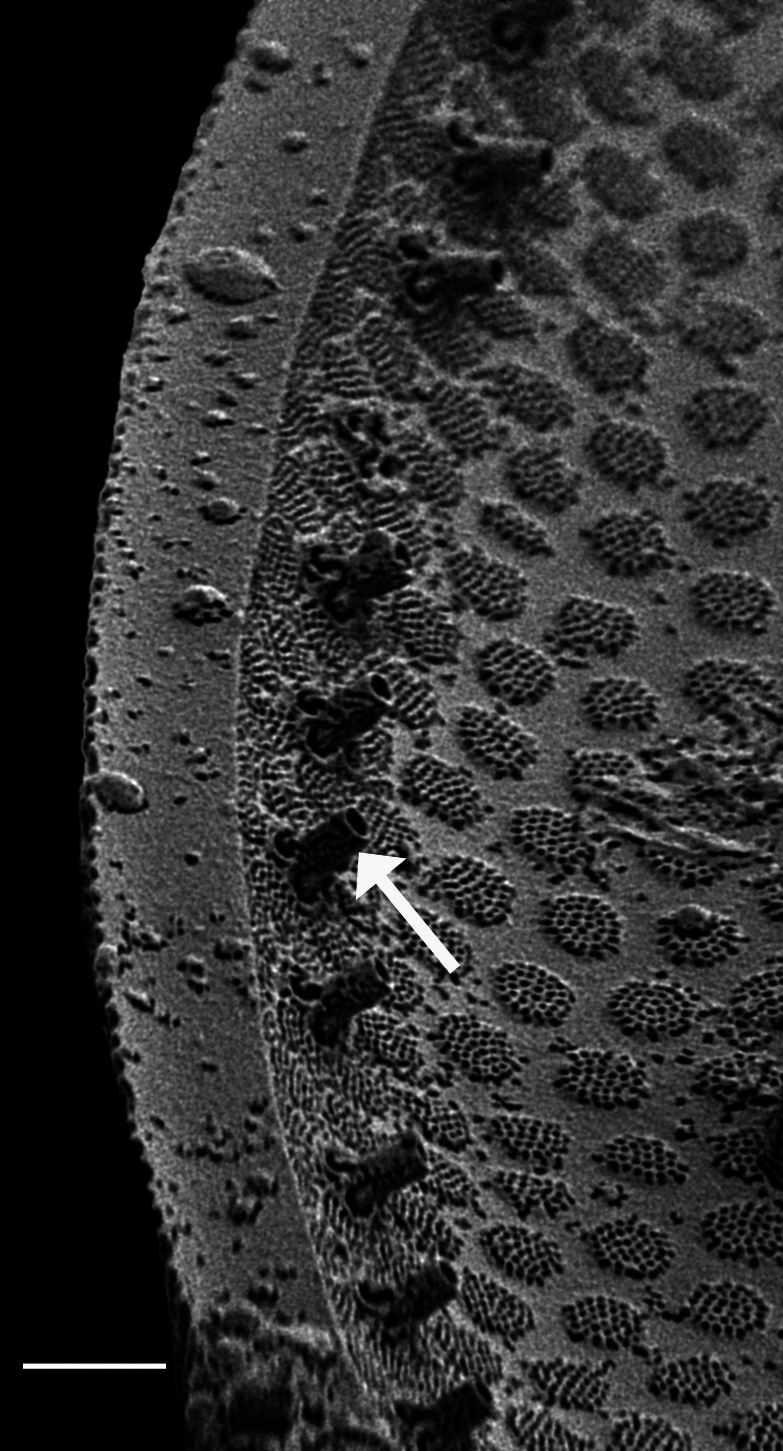
*Shionodiscus endoseriatus*, interior valve view. Arrow shows the marginal strutted processes extending into the interior. SEM. Scale bar: 1 µm.

**Figure 34a.**
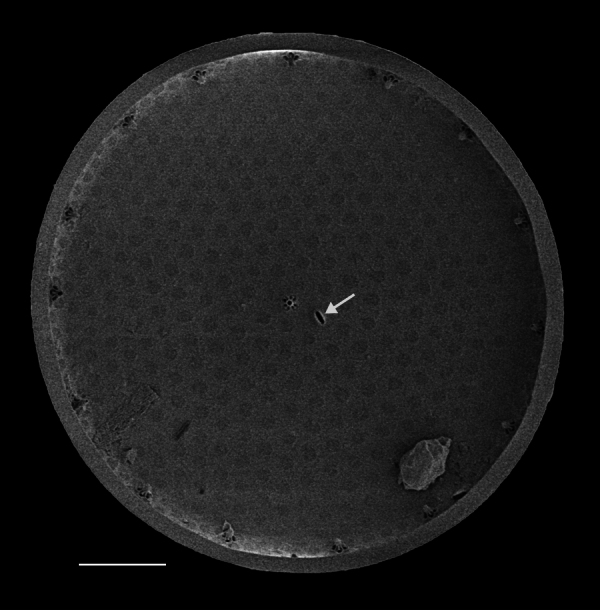
*Shionodiscus karianus*, interior valve view. Arrow indicates location of the rimoportula. SEM. Scale Bar: 5 µm;

**Figure 34b.**
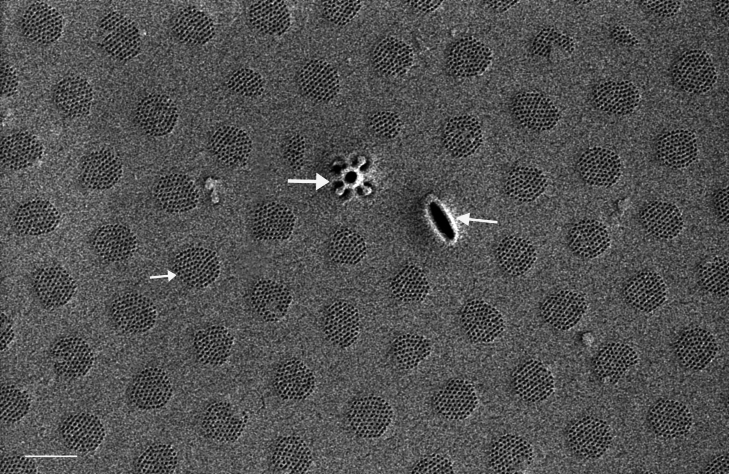
*Shionodiscus karianus*, central interior valve view. Arrows indicate, in decreasing size, the location of: the operculate central fultoportula (CFP), rimoportula and cribrate areolae. SEM. Scale Bar: 1 µm;

**Figure 34c.**
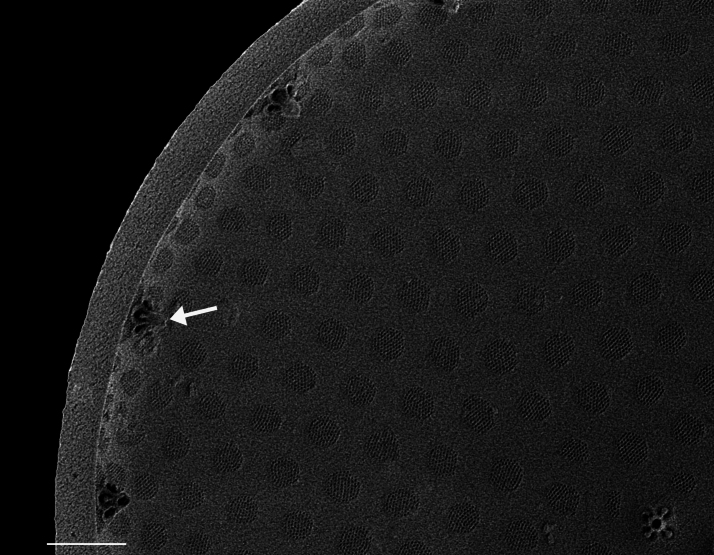
*Shionodiscus karianus*, interior valve view. Arrow indicates a marginal process that extends internally. SEM. Scale Bar: 2 µm.

**Figure 35a.**
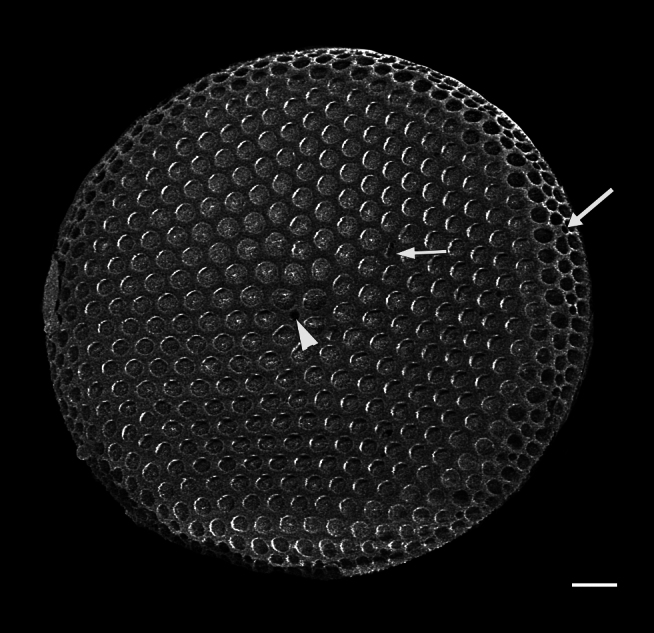
*Shionodiscus oestrupii*, exterior valve view. Shortest arrow is location of the central process; medium arrow is the location of the exit hole of labiate process; longest arrow is exit hole of a marginal process. SEM. Scale Bar: 2.0 µm;

**Figure 35b.**
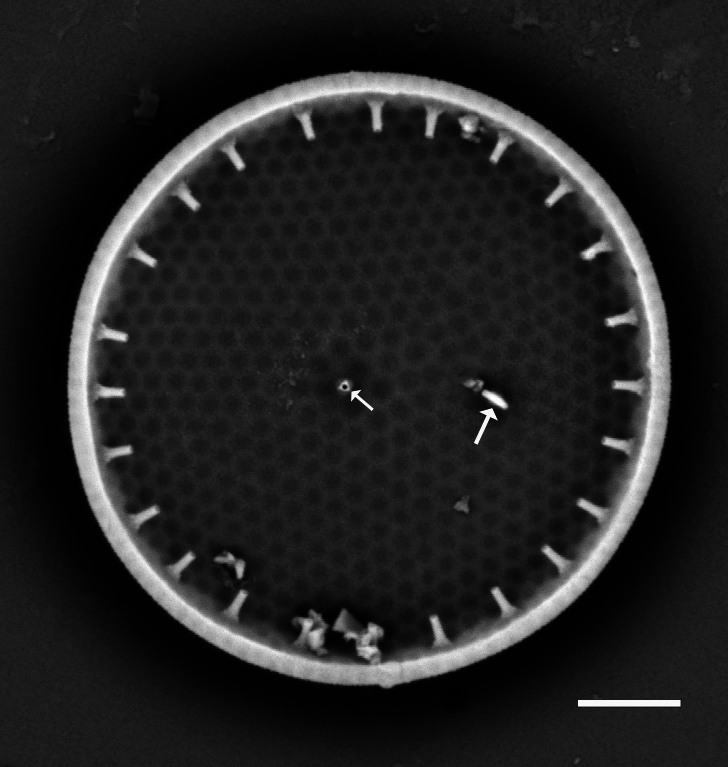
*Shionodiscus oestrupii*. internal valve view. Small arrow shows the central fultoportula and larger arrow is the rimoportula. Note: MFPs extend internally and not externally. SEM. Scale bar: 2 µm.

**Figure 36a.**
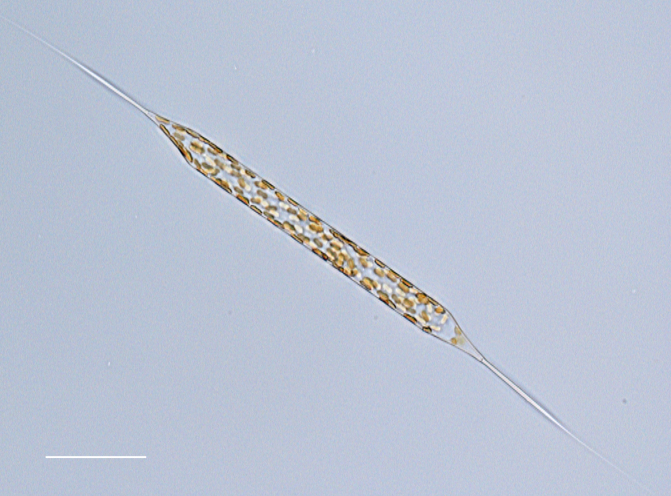
*Sundstroemia pungens*, live cell, girdle view. LM. Scale bar: 50 µm;

**Figure 36b.**
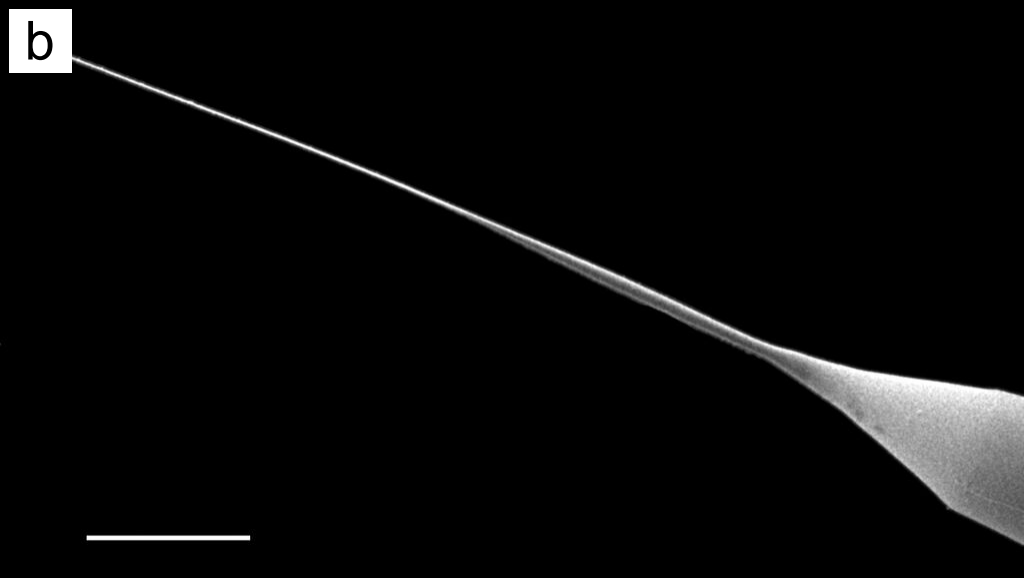
*Sundstroemia pungens*, valve and a distinctive process abruptly swollen for about half its length. SEM. Scale bar: 20 µm.

**Figure 37a.**
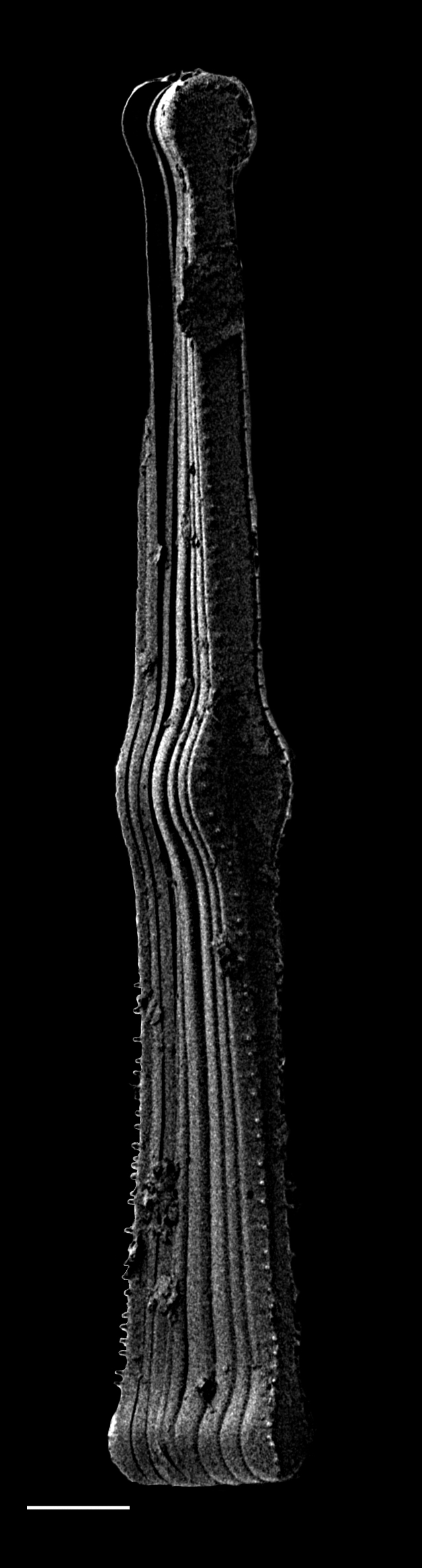
Tabellaria cf. quadriseptata, external girdle and valve views. SEM. Scale bar: 5 µm;

**Figure 37b.**
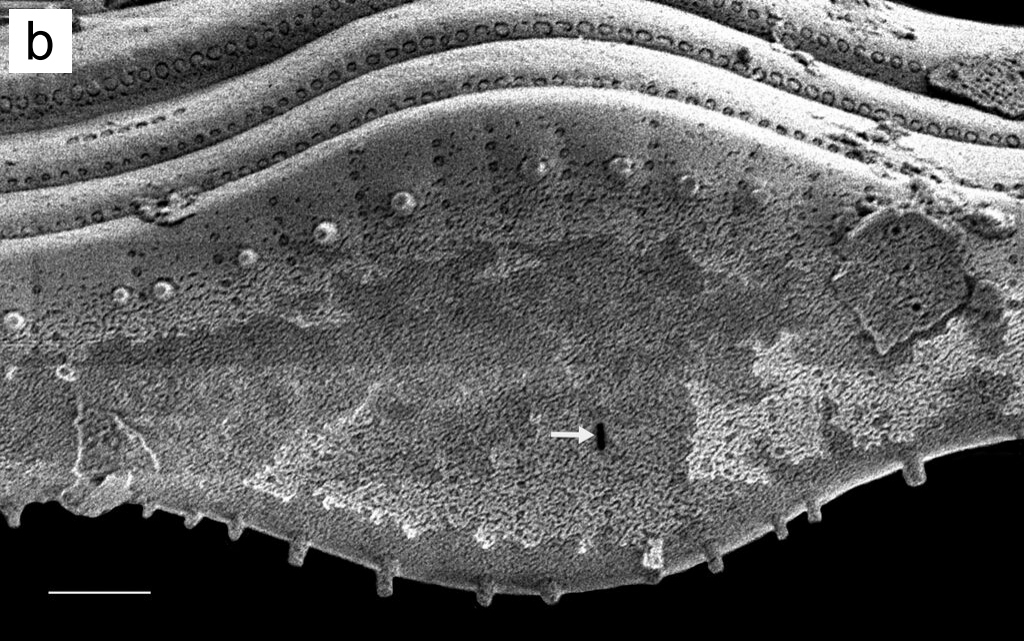
Tabellaria cf. quadriseptata, external valve view. Inflated central area, arrow shows external opening of the rimoportula. SEM. Scale bar: 1 µm;

**Figure 37c.**
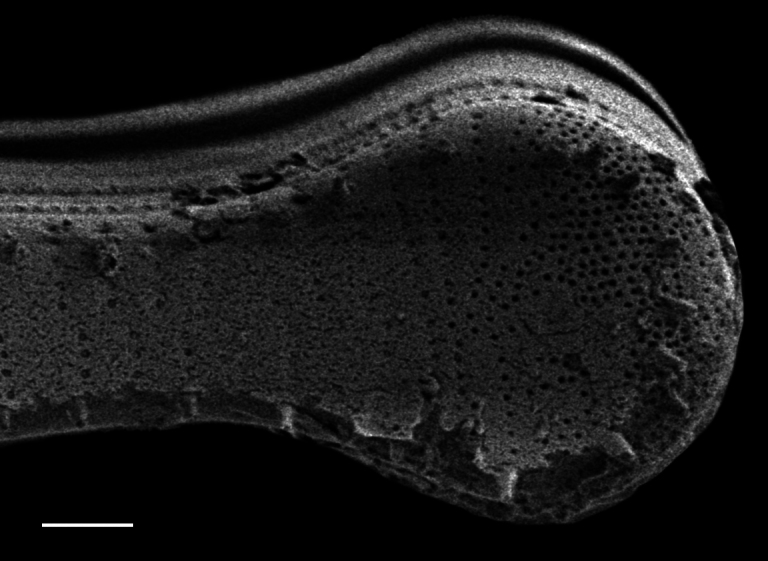
Tabellaria cf. quadriseptata, valve view, external apical pore field. SEM. Scale bar: 1 µm.

**Figure 38a.**
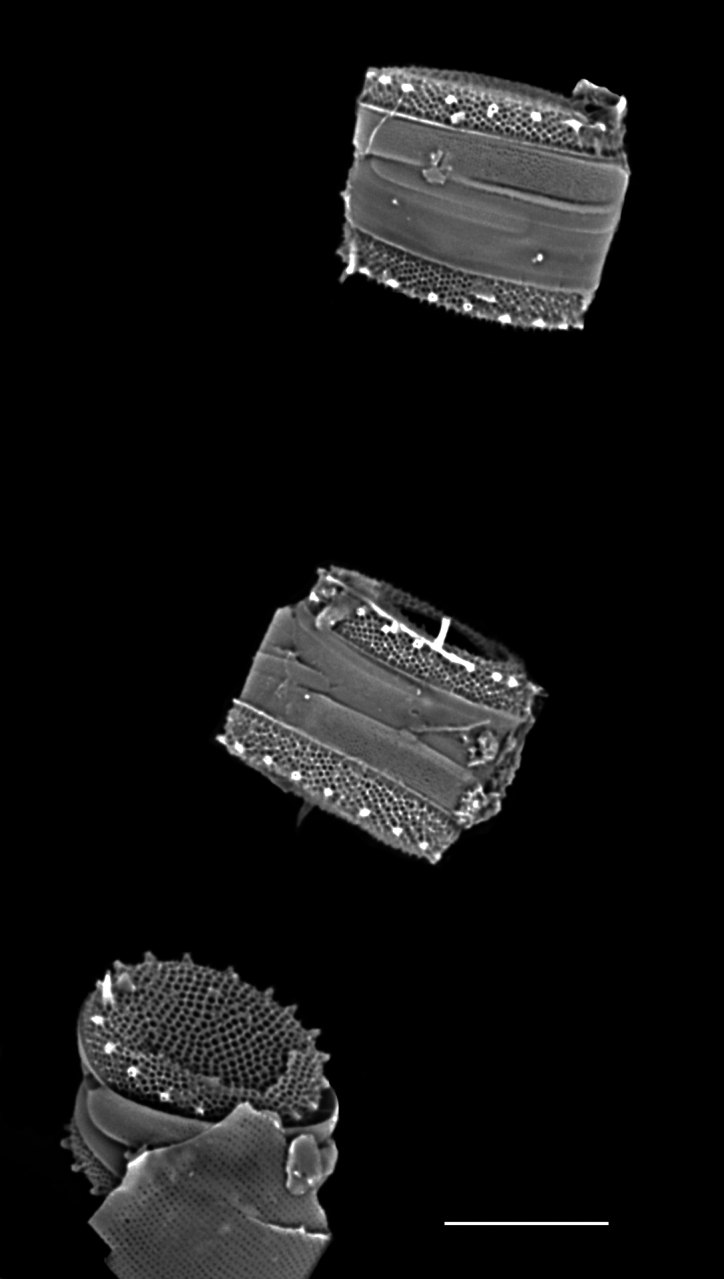
*Thalassiosira allenii*, girdle and valve views. SEM. Scale bar: 10 µm;

**Figure 38b.**
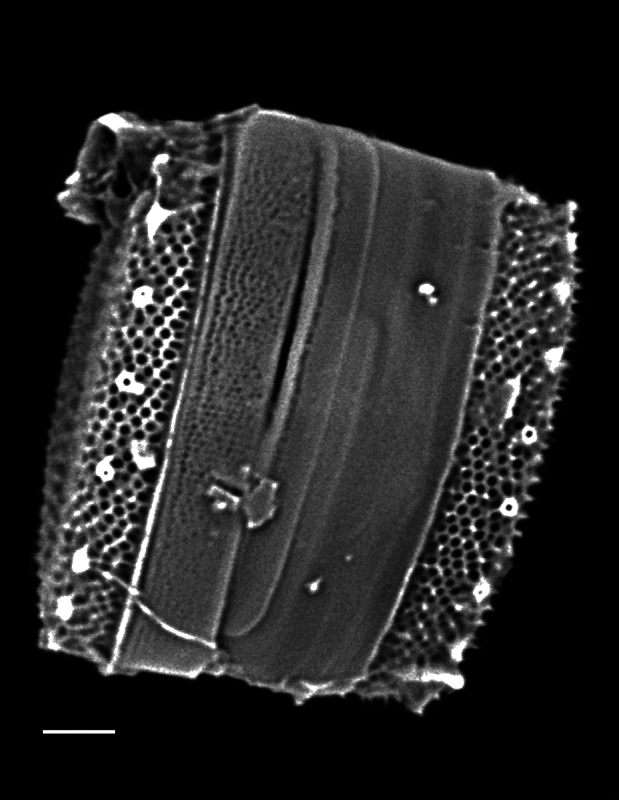
*Thalassiosira allenii*, girdle view. SEM. Scale bar: 2 µm.

**Figure 39a.**
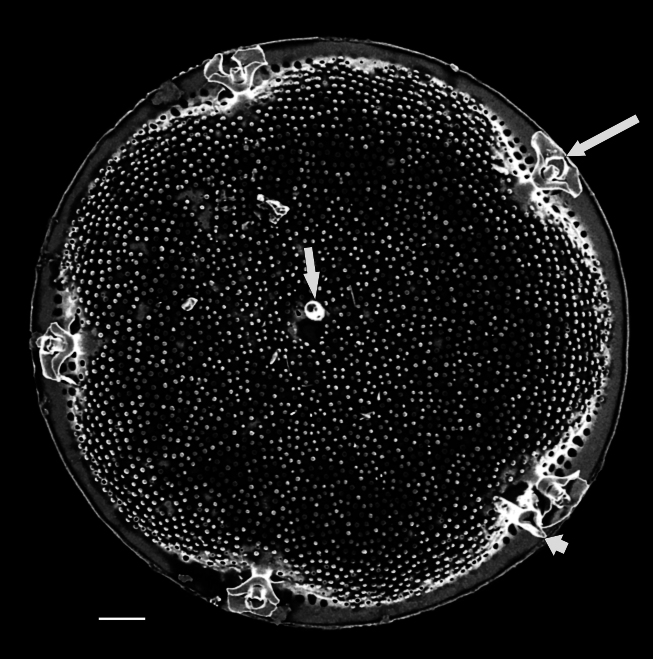
*Thalassiosira curviseriata*, external valve view. Smallest arrow indicates the external rimoportula, mid-size arrow shows the central process and the largest arrow indicates a winged marginal process (MSP). SEM. Scale bar: 1 µm;

**Figure 39b.**
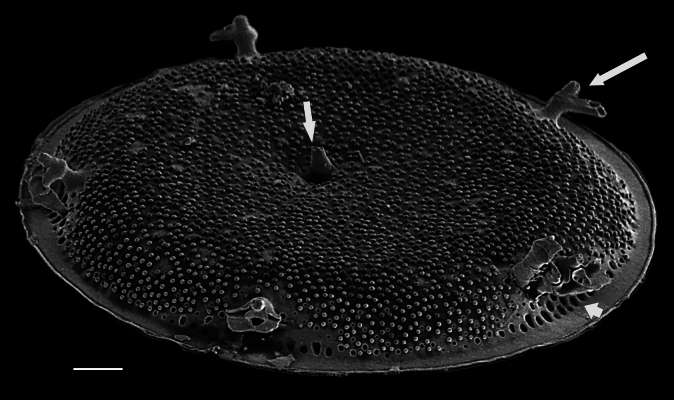
*Thalassiosira curviseriata*, external valve view of the distinctive winged MSP. SEM. Scale bar: 400 nm;

**Figure 39c.**
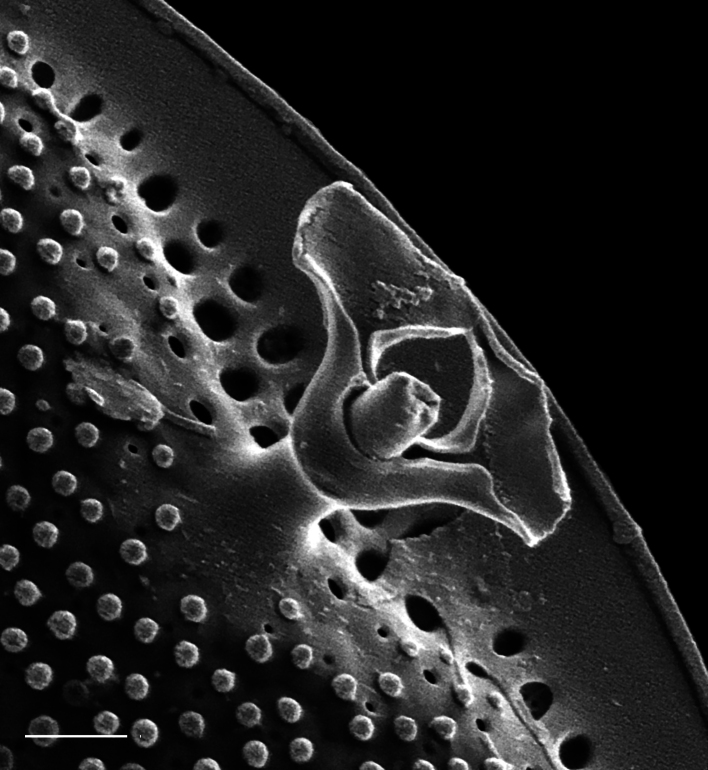
*Thalassiosira curviseriata*, external valve view, image of a winged MSP next to the external rimoportula, oblique angle. SEM. Scale bar: 1 µm;

**Figure 39d.**
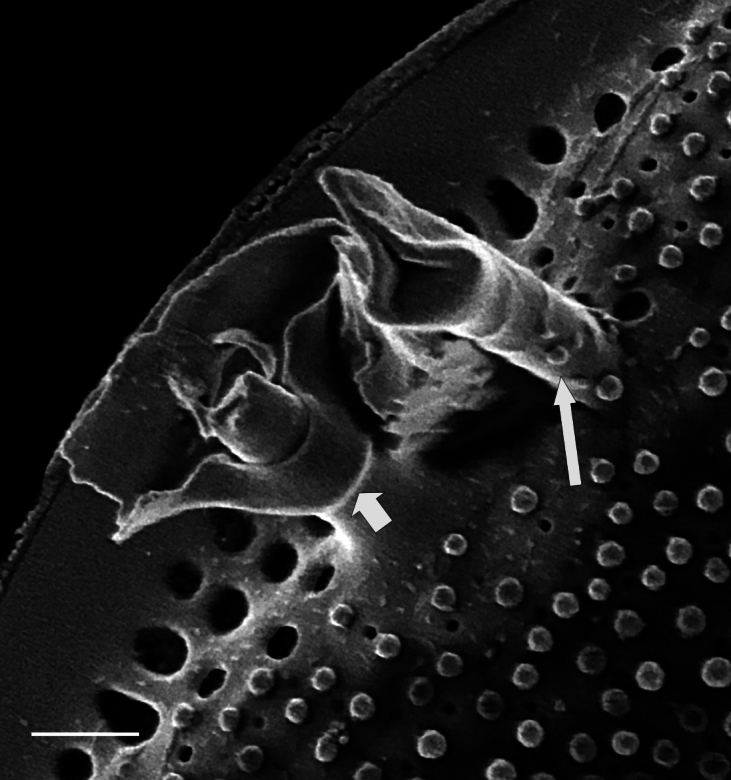
*Thalassiosira curviseriata*, external valve view, image of central process (1), annulus (2) and (3) long spinules. SEM. Scale bar: 400 nm.

**Figure 43a.**
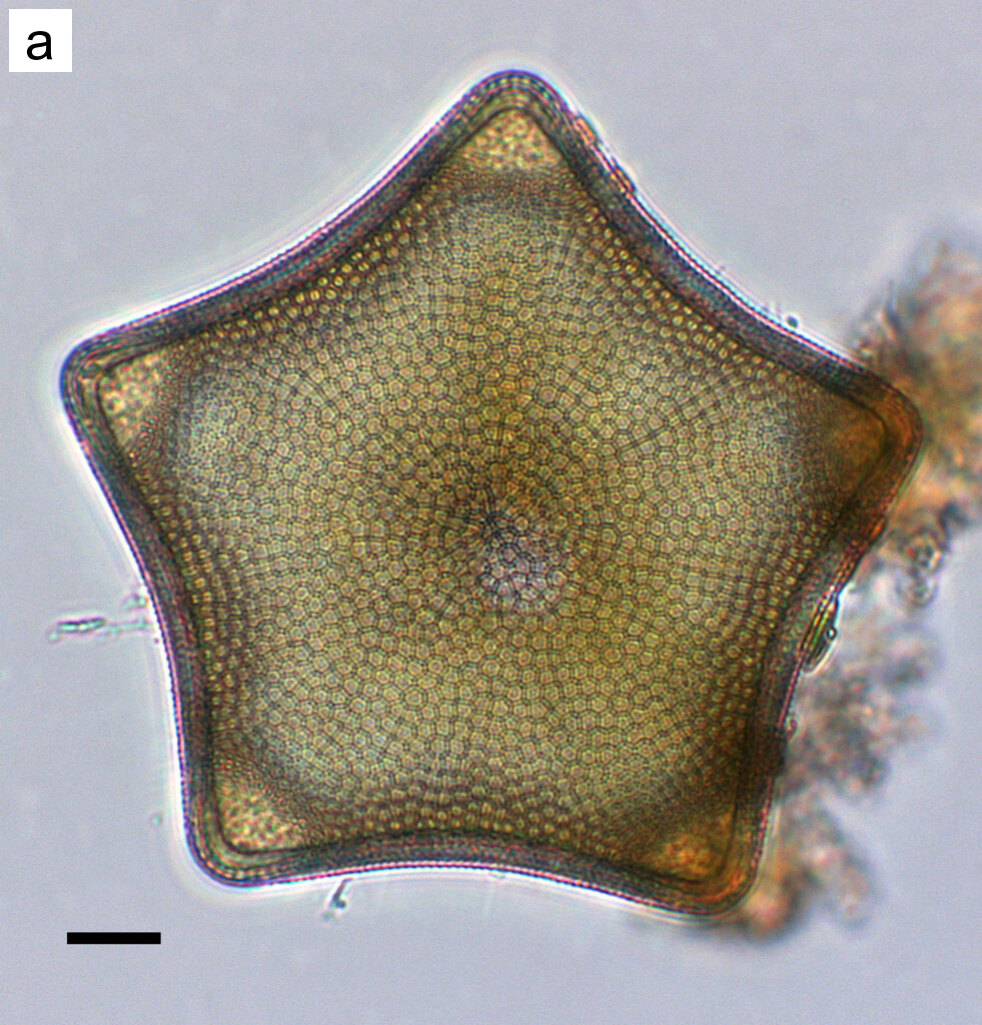
*Trigonium quinquelobatum*, live cell, valve view. LM. Scale bar: 20 µm;

**Figure 43b.**
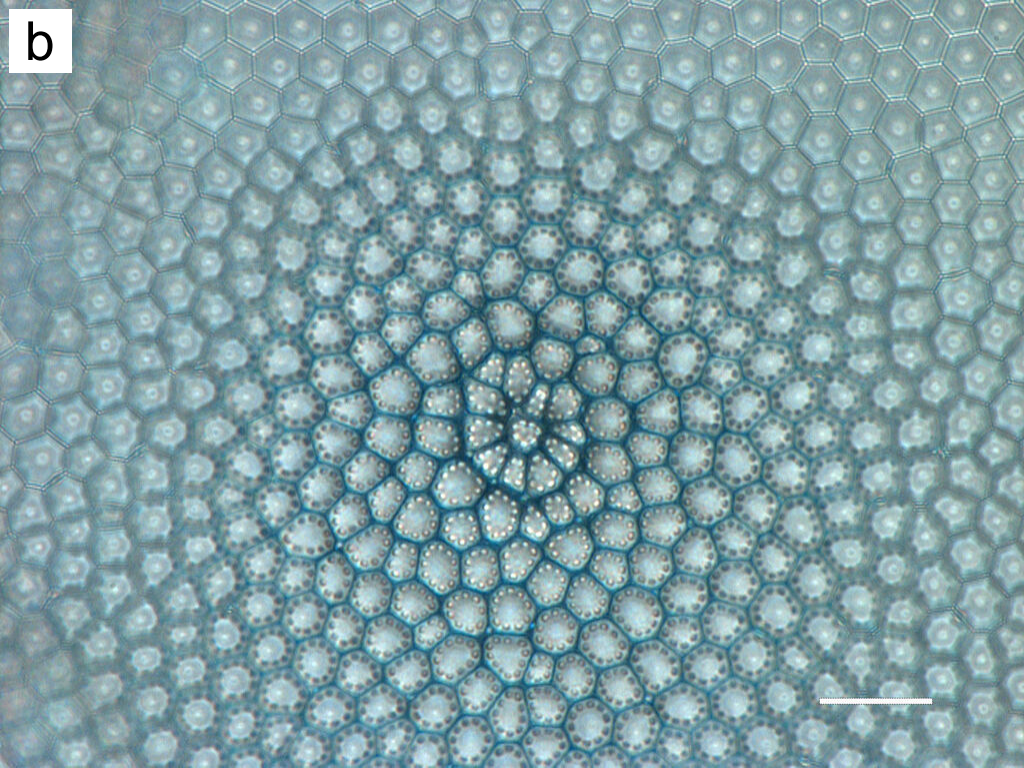
*Trigonium quinquelobatum*, external valve, cleaned cell, with distinctive central areolae rosette. Stack of five images in polarised light. LM. Scale bar: 10 µm;

**Figure 43c.**
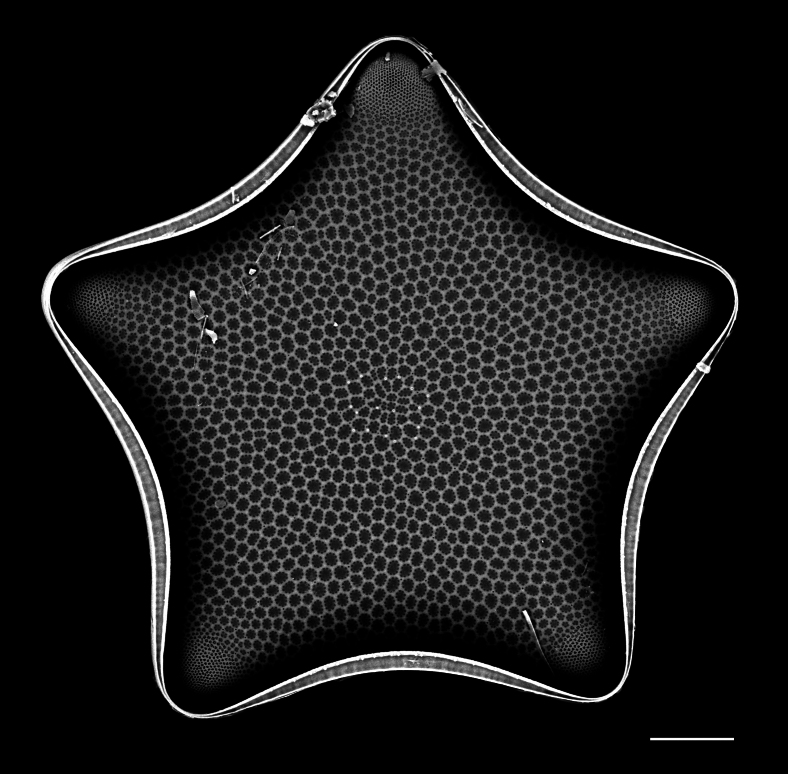
*Trigonium quinquelobatum*, external valve view. SEM. Scale bar: 20 µm;

**Figure 43d.**
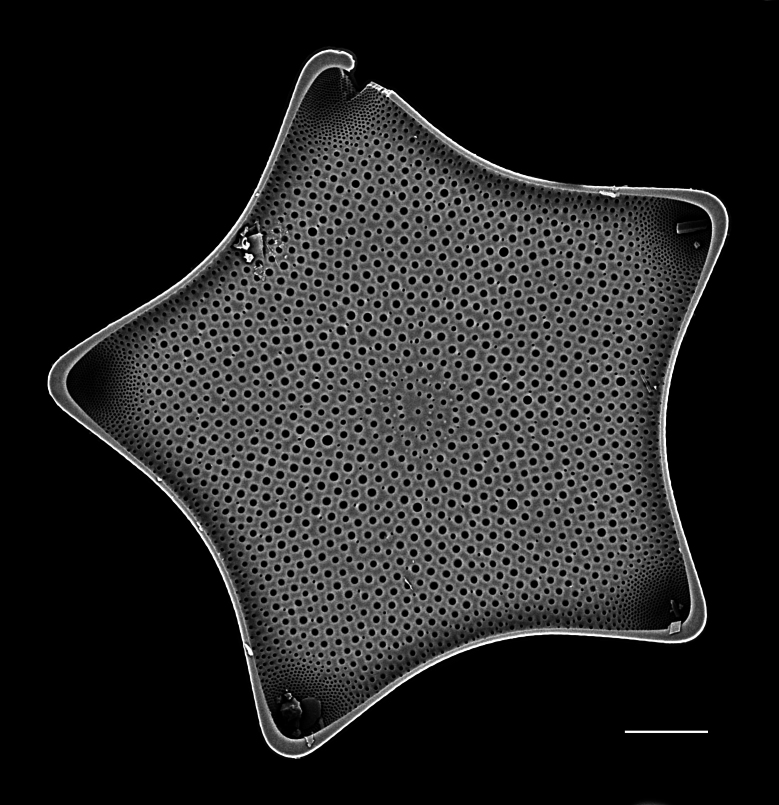
*Trigonium quinquelobatum*, internal valve view. SEM. Scale bar: 10 µm.

**Figure 44a.**
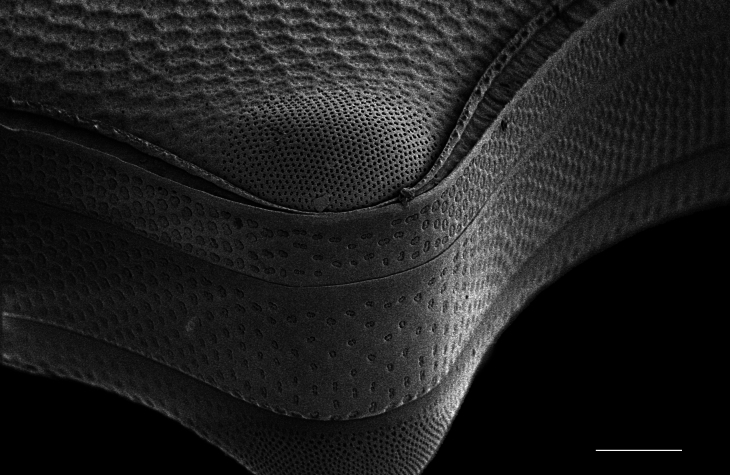
*Trigonium quinquelobatum*, external valve and girdle views. Large pseudocellus and girdle bands of an intact cell, partial cleaning. SEM. Scale bar: 15 µm;

**Figure 44b.**
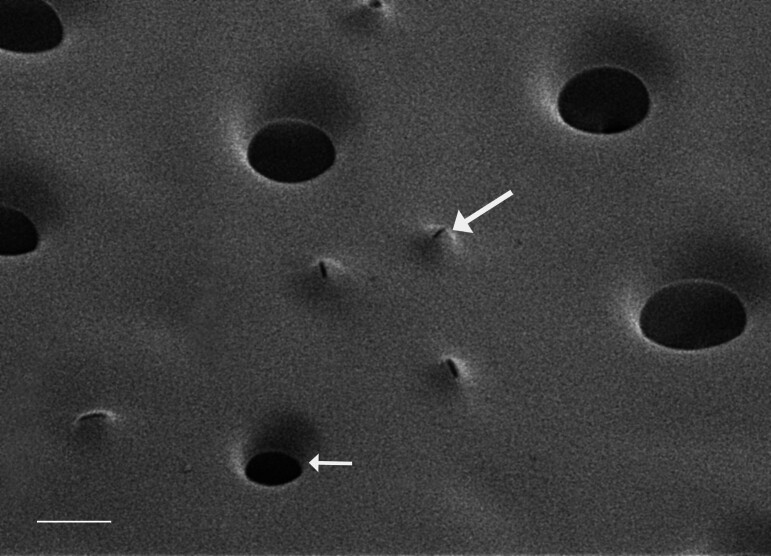
*Trigonium quinquelobatum*, internal valve, small arrow indicates the central areolae, larger arrow shows one of the stalkless rimoportulae. SEM. Scale bar: 1 µm;

**Figure 44c.**
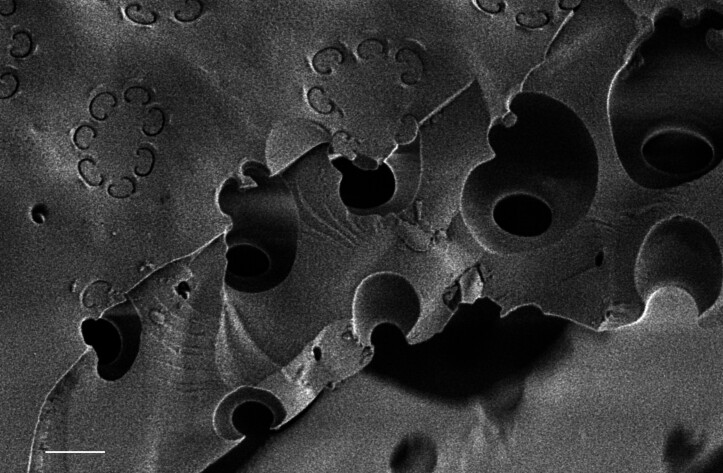
*Trigonium quinquelobatum*, cross-section of the valve showing the cribra and loculate chambers of a semi-cleaned frustule. SEM. Scale bar: 1 µm.

**Table 1.** Sampling sites, dates and methods. See Fig. 1 for map of site locations.

**Site name**	**Coordinates**	**Sampling dates**	**Methods**
Active Pass East (APE)	48.8770, -123.2976	05.VIII.2024	20-µm plankton net sample (0–10 m depth)
Active Pass West (APW)	48.8607, -123.3357	05.VIII.2024	20-µm plankton net sample (0–10 m depth)
Chivers Point (CP), Wallace Island, Trincomali Channel	48.9572, -123.5743	25.VI.2022, 27.IX.2025	20-µm plankton net sample (0–4 m depth)
Departure Bay (DB), Nanaimo, public beach	49.2004, -123.9676	06.V.2024	20-µm plankton net sample (0–1 m depth); *Z. marina*; tidal flat sand samples
Dionisio Point (DP), Galiano Island, east lagoon; Hul’qumi’num name: *quelus*'	49.0136, -123.5749	15.VII.2022, 08.V.2025	20-µm plankton net sample (0–1 m depth)
Dionisio Reefs (DR), Porlier Pass	49.0158, -123.5838	08.V.2025	Brushings from unidentified red alga collected at depth by scuba
Gabriola Pass (GP)	49.1291, -123.7030	07.IX.2012	20-µm plankton net sample (0–10 m depth)
Miners Bay Wharf (MBW), Active Pass, Mayne Island	48.8521, -123.3022	20.VII.2022, 31.X.2022	20-µm plankton net sample (0–3 m depth); brushings from the red alga *Scagelia americana*
Montague Harbour Marine Provincial Park (MHMPP), Galiano Island; Hul’qumi’num name: *sumnuw*	48.9007, -123.4079	08.I.2013, VII–VIII.2020, 15.XI.2020, 07.III.2021, 22.VII.2021, 07.XI.2021	Site of multi-year eelgrass (*Z. marina*) microbiota study; morphological investigations combined with molecular sequencing
Navy Channel (NC) (between Mayne Island and Pender Island	48.8144, -123.2685	05.VIII.2024	20-µm plankton net sample (0–10 m depth)
North Saltery Bay (NSB), Beach Access Site 45, Galiano Island	48.9907, -123.5801	24.VII.2024, 11.IX.2025	Sand and mud samples from a tidal flat
Pebble Beach, east side of Galiano Island (PB)	48.9498, -123.4827	25.VI.2020	20-µm plankton net sample (0–2 m depth)
Porlier Pass (PP)	49.0146, -123.5892	25.VI.2022, 18.X.2022, 16.III.2024, 15.IX.2025	20-µm plankton net sample (0–10 m depth)
Quadra Island, Sutil Channel (Q39)	50.0307, -125.0992	09.V.2024	64-µm plankton net sample (unknown depth); macroalgae samples
Quadra Island, beach in front of Hakai Institute labs (Q45)	50.1153, -125.2201	07.V.2024	Water scooped off the beach
Retreat Cove (RC); Hul’qumi’num name: *xetthecum*	48.9393, -123.5021	26.V.2023, 14.VII.2024	Macroalgae samples (*Ceramium pacificum*, *Costaria costata*, *Sarcodiotheca gaudichaudii*, *Ceramothamnion pacificum*,*Polyneura latissima*) collected by scuba during 2023 Galiano Island BioBlitz
Sidney Channel (SC)	48.6439, -123.3439	15.VII.2021	*Z. marina* sample dried on boat deck
Sidney public beach (SPB), Saanich Peninsula	48.6371, -123.4020	12.IX.2025	Brushings from *Z. marina* and from an unidentified filamentous red alga
Sidney Spit (SS), Sidney Island	48.6443, -123.3648	VII.2024	Brushings from *Z. marina*
Spanish Hills Wharf (SHW) (Spanish Hills Public Dock, North Galiano Island Dock), Trincomali Channel	48.9947, -123.5847	14.VII.2005, 21.VII.2006, 15.XII.2009, 26.X.2010, 20.XII.2010, 31.VII.2013, 13.VIII.2013, 20.XI.2019, 07.XII.2029, 30.V.2021, 03.VII.2021, 27.X.2021 (dock samples of *Obelia* sp.), 06.IV.2022, 15.IV.2022, 26.IV.2022, 25.VI.2022, 08.VII.2022, 15.VIII.2022; (almost every month sampling to VII.2025	20-µm plankton net samples (0–6 m depth); *Obelia* sp. (attached to the dock) sample
Strait of Georgia, east Galiano Island, nearshore (SGE-A)	49.0055, -123.5644	05.VII.2022, 26.IX.2022, 16.III.2024	20-µm plankton net sample (0–5 m depth)
Strait of Georgia, east Galiano Island, offshore (SGE-B)	49.0190, -123.5552	15.VII.2022, 26.IX.2022, 18.X.2022	20-µm plankton net sample (0–10 m depth)
Stuart Channel (SC), west of Thetis Island, adjacent to Fraser Point	49.0185, -123.7162	02.VIII.2023	20-µm plankton net sample (0–10 m depth)
Sturdies Bay (SB) ferry dock	48.8767, -123.3135	12.I.2006, 26.V.2023	20-µm plankton net sample taken along the dock walkway (0–3 m depth)
Trincomali Channel (TC)	49.0009, -123.6053	28.VII.2004, 14.VII.2005, 19.VII.2006, 11.VII.2008, 15.XI.2008, 19.VII.2012, 03.VII.2021, 16.III.2024	20-µm plankton net sample (0–10 m depth)

**Table 2.** Salish Sea diatom records by source: historical literature, molecular data and morphological data. Material that is uniquely contributed through this research is marked ‘WL’ (Webber Lab) and ‘IMERSS’, abbreviations corresponding to those used in the annotated checklist (Suppl. material 1). Accession numbers are provided for all molecular data submitted to the European Nucleotide Archive (ENA).

**Type**	**Source**	**Records**	**Unique taxa**
Literature	Various works cited in annotated checklist	2259	815
Molecular	IMERSS – 2023 Galiano BioBlitz (amplicon) (ENA accession: PRJEB102646)	1365	56
Molecular	Ecological genomics of a seasonally anoxic fjord; Saanich Inlet – data derived from the European Bioinformatics Institute (amplicon) (MGnify 2021)	474	43
Molecular	IMERSS – Plankton samples (amplicon) (ENA accession: PRJEB102646)	432	40
Molecular	*Zostera* epiphyte samples (amplicon) (Jackman et al. 2025) (ENA accession: PRJEB72893)	428	69
Molecular	IMERSS – *Scagelia* epiphyte sample (amplicon) (ENA accession: PRJEB102646)	63	41
Molecular	IMERSS – Diatom clones (Sanger) (ENA accession: ERZ29258993)	6	3
Morphology	Phytoplankton community composition links to environmental drivers across a fjord to shelf gradient on the central coast of British Columbia (Del Bel Belluz et al. 2024)	2354	45
Morphology	Harmful algae in the Strait of Georgia, 2015–2023 (Esenkulova and Salinas-Ruiz 2024)	1338	1
Morphology	WL – Morphological data (LM and SEM micrographs, iNaturalist observations) generated by Webber Lab (iNaturalist 2025)	317	174
Morphology	Continuous plankton recorder survey (CPR Survey) – Marine Biological Association (Lear 2022)	201	20
Morphology	University of British Columbia Herbarium (UBC) – Algae Collection (University of British Columbia Herbarium 2024)	58	5
Morphology	The Diatom Collection of Franz Josef Weinzierl at the Botanische Staatssammlung München (M) (Staatliche Naturwissenschaftliche Sammlungen Bayerns 2025)	17	16
Morphology	Harmful Algal Event Database (HAEDAT) (Provoost and Enevoldsen 2025)	12	2
Morphology	Brown University Herbarium (BRU) (Brown University Herbarium 2025)	6	4
Morphology	Michigan State University Herbarium (MSC) (Michigan State University Herbarium 2025)	6	5
Morphology	W.S. Turrell Herbarium, Miami University (MU) (W.S. Turrell Herbarium 2025)	6	5
Morphology	The New York Botanical Garden (NY) (Ramirez et al. 2025)	4	3
Morphology	University and Jepson Herbaria, University of California, Berkeley (UC) (Alexander and Gross 2024)	4	1
Morphology	Natural History Museum (London) (NHMUK) (Natural History Museum 2025)	2	2
Morphology	Florida Museum of Natural History (FLAS) (Florida Museum of Natural History 2025)	1	1
Morphology	University of New Hampshire Nature History Collections (NHA) (University of New Hampshire Natural History Collections 2025)	1	1

**Table 3.** New reports for the Salish Sea (2011–2026). Locations: Chivers Point (CP), Wallace Island; Departure Bay (DB), Nanaimo; Dionisio Reefs (DR), Porlier Pass; Strait of Georgia, east Galiano Island, offshore (SGE-B); Dionisio Point (DP), Galiano Island (Hulq’umi’num: *quelus*); Lochside Waterfront Park (LWP), Sidney, Vancouver Island; Miners Bay Wharf (MBW), Mayne Island; Montague Harbour Marine Provincial Park (MHMPP), Galiano Island (Hulq’umi’num: *sumnuw*); North Saltery Bay (NSB), Galiano Island; Porlier Pass (PP); Quadra Island, beach in front of Hakai Institute labs (Q45); Retreat Cove (RC), Galiano Island (Hulq’umi’num: *xetthecum*; Sidney Channel (SC); Spanish Hills Wharf (SHW); Trincomali Channel (TC); Sturdies Bay (SB), Galiano Island. Vouchers at Webber Lab (WL) are identified as light microscope (LM) slides and scanning electron microscope (SEM) stubs.

**Taxon**	**Location**	**Voucher**
Achnanthes groenlandica var. meridiana Giffen	DR, MHMPP	WL-SEM-108; WL-SEM-109; WL-SEM-110
Actinoptychus adriaticus var. pumila Grunow	CP	WL-SEM-90
*Andrzeja fenestrata* Mayama & Kryk	LWPMHMPP	WL-SEM-3-1; WL-SEM-60-3
*Attheya longicornis* R.M.Crawford & C.Gardner	SHW	WL-SEM-113
*Bacteriastrum hyalinum* Lauder	PP	WL-LM-51
Bacterosira cf. constricta (Gaarder) J.S.Park & J.H.Lee	SHW	WL-SEM-9
*Campylodiscus bicostatus* W.Smith ex Roper	MHMPP	WL-LM-42
Cocconeis costata var. hexagona Grunow	Q45, SB	WL-LM-24; WL-SEM-75
*Cocconeis kerguelensis* P.Petit	SHW	WL-LM-26
*Cocconeis notata* P.Petit	SHW	WL-LM-20
Cocconeis pseudomarginata var. intermedia Grunow	RC	WL-SEM-77
Cocconeis scutellum var. posidoniae M.De Stefano, D.Marino & L.Mazzella	MHMPP	WL-LM-37
*Cyclotella baltica* (Grunow) Håkansson	SHW	WL-SEM-91
*Didymosphenia geminata* (Lyngbye) Mart.Schmidt	PP	WL-LM-46
*Diploneis exemta* (A.W.F.Schmidt) Cleve	SHW	WL-SEM-92
*Extubocellulus spinifer* (Hargreaves & Guillard) Hasle, Stosch & Syvertsen	NSB	WL-SEM-93
*Fogedia krammeri* Witkowski, Lange-Bertalot, Kociolek & M.Kulikovskiy	MHMPP, SHW	WL-LM-43; WL-SEM-94
*Gomphonemopsis pseudexigua* Medlin	SHW	WL-SEM-118
*Gomphoseptatum aestuarii* (Cleve) Medlin	MBW	WL-SEM-103
*Gomphoseptatum pseudoseptatum* (Giffen) Witkowski, Lange-Bertalot & Metzeltin	MHMPP	WL-SEM-107
*Gyrosigma arcuatum* (Donkin) Sterrenburg	MHMPP, RC, SB	WL-SEM-3; WL-SEM-37; WL-SEM-116
*Homoeocladia spathulatoides* (Lobban, Ashworth, Calaor & E.C.Theriot) Lobban & Ashworth	MHMPP	WL-SEM-66
*Isthmia enervis* Ehrenberg	SC	WL-SEM-6
Licmophora cf. communis (Heiberg) Grunow	RC	WL-SEM-96
*Licmophora tincta* (C.Agardh) Grunow	SHW	WL-SEM-89
*Minidiscus proschkinae* (Makarova) J.S.Park & J.H.Lee	CP	WL-SEM-97
*Neocalyptrella robusta* (G.Norman ex Ralfs) Hernández-Becerril & Meave	SHW	WL-LM-50; WL-SEM-66-7
Parlibellus delognei f. ellipticus (Lobban) E.J.Cox	MHMPP, RC	WL-LM-29; WL-SEM-16
*Proschkinia complanatula* (Hustedt ex Simonsen) D.G.Mann	MHMPP	WL-SEM-105
*Seminavis robusta* D.B.Danielidis & D.G.Mann	RC	WL-LM-44
*Shionodiscus endoseriatus* Hasle & Fryxell) Alverson, Kang et Theriot	SGE-B	WL-SEM-72
*Shionodiscus frenguelliopsis* (Fryxell & Johansen) Alverson, Kang & Theriot	DB	WL-SEM-106
*Shionodiscus karianus* Georgiev & Gololobova	SHW	WL-SEM-111
*Shionodiscus oestrupii* (Ostenfeld) A.J.Alverson, S.-H.Kang & E.C.Theriot	RC, SGE-B	WL-SEM-18; WL-SEM-71
*Sundstroemia pungens* (A.Cleve) Medlin, Lundholm, Boonprakob & Moestrup	PP	WL-LM-45
*Tabellaria quadriseptata* B.M.Knudson	MBW	WL-SEM-74
*Thalassiosira allenii* H.Takano	MHMPP, PP	WL-SEM-63
*Thalassiosira curviseriata* Takano	MHMPP	WL-SEM-44
*Thalassiosira minima* Gaarder	MHMPP, NSB	WL-SEM-99; WL-SEM-100
*Thalassiosira oceanica* Hasle	PP	WL-SEM-65
Thalassiosira cf. visurgis Hustedt	MHMPP	WL-SEM-99
*Trigonium quinquelobatum* (Greville) A.Mann	DR	WL-LM-47; WL-SEM-102
