# Supplementary material for: Diatoms (Bacillariophyta) of the Salish Sea, Northeast Pacific: annotated checklist and new species reports
Source: Biodivers Data J. 2026 May 15;14:e189060. doi: 10.3897/BDJ.14.e189060 (PMC13197818; doi:10.3897/BDJ.14.e189060)
Supplement: Supplementary material 1 — Diatoms (Bacillariophyta) of the Salish Sea, Northeast Pacific (1866–2026): Annotated Checklist [file bdj-14-e189060-s001.pdf]

Diatoms (Bacillariophyta) of the Salish Sea, Northeast Pacific (1866–2026): Annotated Checklist

March 31, 2026

Mark Webber, Arjan van Asselt, Alice Chang, Andrew D.F. Simon

This annotated checklist synthesizes 11,469 diatom occurrence records from the Salish Sea bioregion, encompassing 924 unique taxa reported between 1866 and 2026. To ensure concision, each taxon is represented by a single record per data source, including literature, voucher specimens, validated iNaturalist observations, and molecular data (Table S1). Morphological records are linked to voucher codes and, where applicable, iNaturalist observation identifiers; molecular records are annotated with unique identifiers retrievable through the European Nucleotide Archive (ENA). Novel data generated in this study are designated as 'WL' (Webber Lab) and 'IMERSS' (Institute for Multidisciplinary Ecological Research in the Salish Sea). New records (42 taxa) for the Salish Sea are denoted by ✦, and new records for the Pacific coast of North America (2 taxa) by ✦✦. Habitat designations are as follows: marine (M), freshwater (F), freshwater–marine (FM), and brackish (B). Although freshwater taxa are included, the checklist is restricted to those sampled within the marine waters of the Salish Sea. For convenience, the checklist organizes all taxa within a three-class, morphology-based framework that separates centric diatoms (Coscinodiscophyceae), araphid pennates (Fragilariophyceae), and raphid pennates (Bacillariophyceae), following Round et al. (1990)—with one recently described taxon (*Andrzeja fenestrata*) listed at the end of the checklist as *incertae sedis*.

Coscinodiscophyceae (centrics)

Phylum: Bacillariophyta
- **Class: Coscinodiscophyceae**
  - **Subclass: Archaegladiopsophycidae**
    - **Order: Stephanopyxales**
      - **Family: Endictyaceae**
        - **(M)** ***Endictya oceanica*** **Ehrenberg**
          - **Records:** 1 **Literature:** Tempère and Peragallo (1908)

Phylum: Bacillariophyta
- **Class: Coscinodiscophyceae**
  - **Subclass: Archaegladiopsophycidae**
    - **Order: Stephanopyxales**
      - **Family: Stephanopyxidaceae**
        - **(M)** ***Eupyxidicula turris*** **(Greville) S.Blanco & C.E.Wetzel**
          - **Records:** 139 **Literature:** Buchanan (1966), Forbes and Waters (1993), Hobson and McQuoid (2001), McQuoid and Hobson (1997), McQuoid and Hobson (2001), Sancetta and Calvert (1988) **Morphology:** WL: iNat (4848947), Hakai (Hakai_phyto_QU39_2016-05-30_1m_12) **Molecular (amplicon):** EBI (ENA:SRS1690290) **Syn:** *Stephanopyxis turris* (Greville) Ralfs
        - **(M)** ***Stephanopyxis nipponica*** **Gran & Yendo**
          - **Records:** 16 **Literature:** Buchanan (1966), Chester et al. (1979), Clemens (1933), Forbes and Waters (1993), Gran and Angst (1931), Horner et al. (2005), Légaré (1957), Phifer (1932), Shim (1976), Waters et al. (1992) **Morphology:** WL: iNat (45809431), Hakai (Hakai_phyto_QU39_2020-03-18_5m_14)

(M) ***Stephanopyxis palmeriana*** **(Greville) Grunow**
Records: 3 **Literature:** Clemens (1933), Gran and Angst (1931), Phifer (1932)
Syn: *Creswellia palmeriana* Greville

Phylum: Bacillariophyta
Class: Coscinodiscophyceae
Subclass: Biddulphiophycidae
Order: Ardissoneales
Family: Ardissoneaceae
(M) ***Synedrosphenia fulgens*** **(Greville) Lobban & Ashworth**
Records: 1 **Literature:** Buchanan (1966) **Syn:** *Synedra fulgens* (Greville) W.Smith

Phylum: Bacillariophyta
Class: Coscinodiscophyceae
Subclass: Biddulphiophycidae
Order: Toxariales
Family: Toxariaceae
(M) ***Toxarium undulatum*** **Bailey**
Records: 2 **Literature:** Bailey and MacKay (1916), Clemens (1933) **Syn:** *Synedra undulata* (Bailey) W.Smith

Phylum: Bacillariophyta
Class: Coscinodiscophyceae
Subclass: Biddulphiophycidae
Order: Triceratiales
Family: Triceratiaceae
(M) ***Auliscus sculptus*** **(W.Smith) Brightwell**
Records: 5 **Literature:** Bailey and MacKay (1916), Clemens (1933), Lord (1866), Rao and Lewin (1976) **Morphology:** WL: iNat (263023692) **Syn:** *Auliscus caelatus* J.W.Bailey
(M) ***Triceratium montereyi*** **T.Brightwell**
Records: 2 **Literature:** Lord (1866), Tempère and Peragallo (1908)

Phylum: Bacillariophyta
Class: Coscinodiscophyceae
Subclass: Chaetocerotophycidae
Order: Chaetocerotales
Family: Chaetocerotaceae
(M) ***Bacteriastrum delicatulum*** **Cleve**
Records: 6 **Literature:** Chester et al. (1979), Clemens (1933), Gran and Angst (1931), Légaré (1957), Phifer (1932), Shim (1976)
(M) ***Bacteriastrum hyalinum*** **Lauder ✦**
Records: 1 **Morphology:** WL: iNat (315424735) Voucher (WL-LM-51)
(M) ***Chaetoceros affinis*** **Lauder**
Records: 17 **Literature:** Buchanan (1966), Clemens (1933), Forbes and Waters (1993), Gran and Angst (1931), Haigh et al. (1992), Légaré (1957), McQuoid and Hobson (1997), Phifer (1932), Roelofs (1983), Sancetta and Calvert (1988), Shim (1976) **Morphology:** WL: iNat (4876445) **Molecular (amplicon):** EBI (ENA:SRS2340124)
(M) ***Chaetoceros anastomosans*** **Grunow**
Records: 1 **Literature:** Gran and Angst (1931)
(M) ***Chaetoceros atlanticus*** **Cleve**
Records: 2 **Literature:** Clemens (1933), Gran and Angst (1931)
(M) ***Chaetoceros borealis*** **Bailey**

Records: 2 **Literature:** Bailey and MacKay (1916), Clemens (1933) **Syn:** *Chaetoceros boreale* Bailey

(M) ***Chaetoceros brevis*** **F.Schütt**
Records: 13 **Literature:** Chester et al. (1979), Légaré (1957), Shim (1976) **Molecular (amplicon):** EBI (ENA:SRS2340146)

(M) ***Chaetoceros ceratosporus*** **Ostenfeld**
Records: 37 **Literature:** Buchanan (1966), Shim (1976) **Morphology:** Hakai (Hakai_phyto_QU39_2017-11-16_1m_1)

(M) ***Chaetoceros cinctus*** **Gran**
Records: 89 **Literature:** Bailey and MacKay (1916), Clemens (1933), Gran and Angst (1931), Gucluer and Gross (1964), Hobson and McQuoid (2001), Shim (1976), Waters et al. (1992) **Morphology:** Hakai (Hakai_phyto_QU39_2017-05-17_1m_2) **Notes:** Gran and Angst (1931, p. 485) "consider *C. crucifer* as the Pacific representative of *C. cinctus* Gran"; *C. crucifer* H. H. Gran is a non-verified species (Guiry and Guiry 2025); placed under *C. cinctus* Gran for this historic record. **Syn:** *Chaetoceros crucifer* Gran

(M) ***Chaetoceros compressus*** **Lauder**
Records: 31 **Literature:** Buchanan (1966), Chester et al. (1979), Clemens (1933), Forbes and Waters (1993), Gran and Angst (1931), Gucluer and Gross (1964), Haigh et al.(1992), Harrison et al. (1991), Hobson and McQuoid (2001), McQuoid and Hobson (1997), McQuoid and Hobson (2001), Shim (1976), Sutherland et al. (2023), Waters et al. (1992) **Morphology:** Hakai (Hakai_phyto_QU39_2021-06-28_5m_5)

(M) ***Chaetoceros concavicornis*** **Mangin**
Records: 34 **Literature:** Buchanan (1966), Chester et al. (1979), Clemens (1933), Forbes and Waters (1993), Gran and Angst (1931), Hobson (1983), Johnson (1931), Légaré (1957), McQuoid and Hobson (1997), Phifer (1932), Sancetta and Calvert (1988), Shim (1976), Sutherland et al. (2023) **Morphology:** WL: iNat (4876451), Hakai (Hakai_phyto_QU39_2017-07-17_1m_1), IOC.UNESCO (HAEDAT:CA-37:CA-09-004:causative:8177)

(M) ***Chaetoceros constrictus*** **Gran**
Records: 5 **Literature:** Buchanan (1966), Chester et al. (1979), Haigh et al. (1992), Légaré (1957), Shim (1976)

(M) ***Chaetoceros contortus*** **F.Schütt**
Records: 32 **Literature:** Horner et al. (2005) **Molecular (amplicon):** IMERSS (ENA:ERS27217975)

(M) ***Chaetoceros convolutus*** **Castracane**
Records: 86 **Literature:** Buchanan (1966), Chester et al. (1979), Clemens (1933), Forbes and Waters (1993), Gran and Angst (1931), Harrison et al. (1991), Horner et al. (2005), Johnson (1931), Kennedy and LeBrasseur (1977), Légaré (1957), McQuoid and Hobson (1997), Phifer (1932), Shim (1976) **Morphology:** Hakai (Hakai_phyto_QU39_2018-10-04_1m_2), IOC.UNESCO (HAEDAT:CA-37:CA-17-010:causative:9352) **Syn:** *Chaetoceros brightwellii* Gran

(M) ***Chaetoceros costatus*** **Pavillard**
Records: 2 **Literature:** McQuoid and Hobson (1997), McQuoid and Hobson (2001)

(M) ***Chaetoceros crinitus*** **F.Schütt**
Records: 1 **Literature:** Clemens (1933)

(M) ***Chaetoceros criophilus*** **Castracane**
Records: 1 **Literature:** Bailey and MacKay (1916) **Notes:** orth. corr. '*Chaetoceros cryophyllum* Castracane'

(M) ***Chaetoceros curvisetus*** **Cleve**
Records: 4 **Literature:** Haigh et al. (1992), Hobson and McQuoid (2001), Légaré (1957) **Morphology:** WL: iNat (4876455)

(M) ***Chaetoceros danicus*** **Cleve**

Records: 55 **Literature:** Buchanan (1966), Chester et al. (1979), Clemens (1933), Gran and Angst (1931), Hobson and McQuoid (2001), Horner et al. (2005), Phifer (1932), Shim (1976), Waters et al. (1992) **Morphology:** Hakai (Hakai_phyto_QU39_2020-09-01_5m_6)

(M) ***Chaetoceros debilis*** **Cleve**

Records: 181 **Literature:** Buchanan (1966), Chester et al. (1979), Clemens (1933), Forbes and Waters (1993), Gran and Angst (1931), Gucluer and Gross (1964), Haigh et al. (1992), Hobson (1983), Hobson and McQuoid (2001), Horner et al. (2005), Johnson (1931), Légaré (1957), McQuoid and Hobson (1997), McQuoid and Hobson (2001), Parsons et al. (1967), Phifer (1932), Roelofs (1983), Sancetta and Calvert (1988), Shim (1976), Stockner and Cliff (1975), Stockner and Cliff (1976), Sutherland et al. (2023), Waters et al. (1992) **Morphology:** Hakai (Hakai_phyto_QU39_2018-10-04_1m_3) **Molecular (amplicon):** EBI (ENA:SRS962342), IMERSS (ENA:ERS27217975)

(M) ***Chaetoceros decipiens*** **Cleve**

Records: 53 **Literature:** Bailey and MacKay (1916), Buchanan (1966), Chester et al. (1979), Clemens (1933), Gran and Angst (1931), Hobson and McQuoid (2001), Horner et al. (2005), Johnson (1931), Légaré (1957), Phifer (1932), Shim (1976), Waters et al. (1992) **Morphology:** WL: iNat (4876188), Hakai (Hakai_phyto_QU39_2020-09-01_5m_8) **Molecular (amplicon):** EBI (ENA:SRS966792)

(M) ***Chaetoceros densus*** **(Cleve) Cleve**

Records: 2 **Literature:** Clemens (1933), Sancetta and Calvert (1988)

(M) ***Chaetoceros diadema*** **(Ehrenberg) Gran**

Records: 65 **Literature:** Bailey and MacKay (1916), Buchanan (1966), Clemens (1933), Forbes and Waters (1993), Gran and Angst (1931), Hobson and McQuoid (2001), Horner et al. (2005), McQuoid and Hobson (1997), McQuoid and Hobson (2001), Phifer (1932), Roelofs (1983), Sancetta and Calvert (1988), Shim (1976), Tempère and Peragallo (1908), Waters et al. (1992) **Morphology:** WL: iNat (263078068), Hakai (Hakai_phyto_QU39_2020-09-01_5m_9) **Molecular (amplicon):** IMERSS (ENA:ERS27217975) **Syn:** *Syndendrium diadema* Ehrenberg

(M) ***Chaetoceros dichaeta*** **Ehrenberg**

Records: 2 **Literature:** Bailey and MacKay (1916), Clemens (1933)

(M) ***Chaetoceros didymus*** **Ehrenberg**

Records: 51 **Literature:** Bailey and MacKay (1916), Buchanan (1966), Chester et al. (1979), Clemens (1933), Gran and Angst (1931), Gucluer and Gross (1964), Hobson (1983), Hobson and McQuoid (2001), Horner et al. (2005), Légaré (1957), McQuoid and Hobson (1997), McQuoid and Hobson (2001), Phifer (1932), Powys (1987), Roelofs (1983), Sancetta and Calvert (1988), Shim (1976), Sutherland et al. (2023), Waters et al. (1992) **Morphology:** WL: iNat (4849241), Hakai (Hakai_phyto_QU39_2020-05-14_5m_3)

(M) ***Chaetoceros difficilis*** **Cleve**

Records: 3 **Literature:** Hobson (1983), Shim (1976), Waters et al. (1992)

(M) ***Chaetoceros eibenii*** **Grunow**

Records: 3 **Literature:** Clemens (1933), Gran and Angst (1931), Phifer (1932)

(M) ***Chaetoceros externus*** **Gran**

Records: 1 **Literature:** Clemens (1933)

(M) ***Chaetoceros filiformis*** **Meunier**

Records: 1 **Literature:** Forbes and Waters (1993)

(M) ***Chaetoceros furcillatus*** **Bailey**

Records: 1 **Literature:** Sancetta and Calvert (1988)

(M) ***Chaetoceros gracilis*** **Pantocsek**

Records: 6 **Literature:** Buchanan (1966), Chester et al. (1979), Hobson (1983), Phifer (1932), Roelofs (1983), Sutherland et al. (2023)

(M) ***Chaetoceros incurvus*** **Bailey**

Records: 1 **Literature:** Lord (1866) **Notes:** orth. corr. '*Chaetoceros incurvum*'

(M) ***Chaetoceros ingolfianus*** **Ostenfeld**

Records: 1 **Literature:** Shim (1976)

(M) ***Chaetoceros laciniosus*** **F. Schütt**

Records: 90 **Literature:** Chester et al. (1979), Clemens (1933), Forbes and Waters (1993), Gran and Angst (1931), Hobson and McQuoid (2001), Horner et al. (2005), Légaré (1957), McQuoid and Hobson (1997), Phifer (1932), Shim (1976), Sutherland et al. (2023), Waters et al. (1992) **Morphology:** WL: iNat (4876214), Hakai (Hakai_phyto_QU39_2018-10-04_1m_4)

(M) ***Chaetoceros lauderi*** **Ralfs ex Lauder**

Records: 4 **Literature:** Buchanan (1966), McQuoid and Hobson (1997), Roelofs (1983), Waters et al. (1992)

(M) ***Chaetoceros lorenzianus*** **Grunow**

Records: 15 **Literature:** Buchanan (1966), Chester et al. (1979), Clemens (1933), Forbes and Waters (1993), Gran and Angst (1931), Gucluer and Gross (1964), Hobson and McQuoid (2001), Légaré (1957), McQuoid and Hobson (1997), McQuoid and Hobson (2001), Phifer (1932), Roelofs (1983), Sancetta and Calvert (1988), Shim (1976) **Morphology:** WL: iNat (263082372)

(M) ***Chaetoceros mitra*** **(Bailey) Cleve**

Records: 2 **Literature:** Hobson and McQuoid (2001) **Morphology:** Hakai (Hakai_phyto_QU39_2018-07-31_1m_5)

(M) ***Chaetoceros neogracilis*** **VanLandingham**

Records: 2 **Literature:** Clemens (1933), Gran and Angst (1931) **Syn:** *Chaetoceros gracilis* F.Schütt, nom. illeg.

(M) ***Chaetoceros pavillardii*** **J.Ikari**

Records: 1 **Literature:** Sancetta and Calvert (1988)

(M) ***Chaetoceros pelagicus*** **Cleve**

Records: 2 **Literature:** Buchanan (1966), Forbes and Waters (1993)

(M) ***Chaetoceros peruvianus*** **Brightwell**

Records: 2 **Literature:** Bailey and MacKay (1916) **Morphology:** WL: iNat (141899549) Voucher (WL-LM-2)

(M) ***Chaetoceros protuberans*** **Lauder**

Records: 1 **Literature:** Buchanan (1966) **Syn:** *Chaetoceros didymus* var. *protuberans* (H.S.Lauder) Gran & K.Yendo

(M) ***Chaetoceros pseudocrinitus*** **Ostenfeld**

Records: 5 **Literature:** Clemens (1933), Forbes and Waters (1993), Gran and Angst (1931), Phifer (1932), Shim (1976) **Syn:** *Chaetoceros ingolfianus* Ostenfeld (as '*Chaetoceras ingolfianum*'), *Chaetoceros pseudocrinitus* Ostenfeld

(M) ***Chaetoceros pseudocurvisetus*** **Mangin**

Records: 1 **Literature:** McQuoid and Hobson (1997)

(M) ***Chaetoceros radicans*** **F. Schütt**

Records: 63 **Literature:** Buchanan (1966), Chester et al. (1979), Clemens (1933), Forbes and Waters (1993), Gran and Angst (1931), Gucluer and Gross (1964), Haigh et al. (1992), Hobson and McQuoid (2001), Horner et al. (2005), Légaré (1957), McQuoid and Hobson (1997), McQuoid and Hobson (2001), Phifer (1932), Powys (1987), Roelofs (1983), Sancetta and Calvert (1988), Shim (1976), Sutherland et al. (2023), Waters et al. (1992) **Morphology:** WL: iNat (4876457), Hakai (Hakai_phyto_QU39_2017-05-17_1m_4) **Molecular (amplicon):** EBI (ENA:SRS2340158) **Syn:** *Chaetoceros scolopendra* Cleve

(M) ***Chaetoceros rostratus*** **Ralfs**

Records: 1 **Literature:** Forbes and Waters (1993)

(M) ***Chaetoceros saltans*** **Cleve**

Records: 2 **Literature:** Bailey and MacKay (1916), Clemens (1933)

(M) ***Chaetoceros secundus*** **Cleve**

Records: 3 **Literature:** Chester et al. (1979), Clemens (1933), Gran and Angst (1931)

(M) ***Chaetoceros seiracanthus*** **Gran**

Records: 69 **Literature:** Clemens (1933), Gran and Angst (1931), Gucluer and Gross (1964), McQuoid and Hobson (1997), McQuoid and Hobson (2001), Roelofs (1983) **Morphology:** Hakai (Hakai_phyto_QU39_2017-10-11_1m_11)

(M) ***Chaetoceros similis*** **Cleve**

Records: 67 **Literature:** Chester et al. (1979), Clemens (1933), Forbes and Waters (1993), Gran and Angst (1931), Hobson and McQuoid (2001), Horner et al. (2005), Légaré (1957), McQuoid and Hobson (1997), Phifer (1932), Waters et al. (1992) **Morphology:** Hakai (Hakai_phyto_QU39_2018-05-15_1m_9) **Molecular (amplicon):** IMERSS (ENA:ERS27217975)

(M) ***Chaetoceros simplex*** **Ostenfeld**

Records: 19 **Literature:** Forbes and Waters (1993), Hobson and McQuoid (2001), Shim (1976) **Morphology:** Hakai (Hakai_phyto_QU39_2018-05-15_1m_10)

(M) ***Chaetoceros socialis*** **H.S. Lauder**

Records: 80 **Literature:** Bailey and MacKay (1916), Buchanan (1966), Chester et al. (1979), Clemens (1933), Forbes and Waters (1993), Gran and Angst (1931), Hobson and McQuoid (2001), Horner et al. (2005), Parsons et al. (1967), Shim (1976), Sutherland et al. (2023), Waters et al. (1992) **Morphology:** WL: iNat (4876466), Hakai (Hakai_phyto_QU39_2021-07-27_5m_10) **Notes:** orth. corr. *Chaetoceros sociale* Lauder **Syn:** *Chaetoceros radians* F.Schütt, *Chaetoceros socialis* Lauder

(M) ***Chaetoceros subsecundus*** **(Grunow ex Van Heurck) Hustedt**

Records: 2 **Literature:** Chester et al. (1979), Gucluer and Gross (1964)

(M) ***Chaetoceros subtilis*** **Cleve**

Records: 30 **Literature:** Bailey and MacKay (1916), Buchanan (1966), Clemens (1933), Forbes and Waters (1993), Hobson and McQuoid (2001), Horner et al. (2005), Shim (1976), Sutherland et al. (2023) **Morphology:** Hakai (Hakai_phyto_QU39_2021-07-27_5m_11)

(M) ***Chaetoceros tenuissimus*** **Meunier**

Records: 77 **Literature:** Hobson and McQuoid (2001) **Morphology:** Hakai (Hakai_phyto_QU39_2016-04-28_1m_31)

(M) ***Chaetoceros teres*** **Cleve**

Records: 7 **Literature:** Buchanan (1966), Chester et al. (1979), Clemens (1933), Gran and Angst (1931), Horner et al. (2005), Légaré (1957), Phifer (1932)

(M) ***Chaetoceros tetrastichon*** **Cleve**

Records: 1 **Literature:** Shim (1976)

(M) ***Chaetoceros tortissimus*** **Gran**

Records: 2 **Literature:** Chester et al. (1979), Waters et al. (1992)

(M) ***Chaetoceros vanheurckii*** **Gran**

Records: 11 **Literature:** Clemens (1933), Gran and Angst (1931), Horner et al. (2005), Légaré (1957), McQuoid and Hobson (1997), McQuoid and Hobson (2001), Phifer (1932), Roelofs (1983), Sancetta and Calvert (1988), Shim (1976), Waters et al. (1992) **Notes:** Waters et al. (1992) indicate that their identification of *C. vanheurckii* might be *C. constrictum*

(M) ***Chaetoceros vistulae*** **Apstein**

Records: 3 **Literature:** Buchanan (1966), Chester et al. (1979), Forbes and Waters (1993)

(F) ***Chaetoceros wighamii*** **Brightwell**

Records: 1 **Literature:** Forbes and Waters (1993)

(M) ***Chaetoceros willei*** **Gran**

Records: 3 **Literature:** Buchanan (1966), Forbes and Waters (1993), Shim (1976)

Phylum: Bacillariophyta

Class: Coscinodiscophyceae

Subclass: Chaetocerotophycidae

Order: Chaetocerotales

Family: Leptocylindraceae

(M) *Leptocylindrus danicus* Cleve

Records: 148 **Literature:** Buchanan (1966), Chester et al. (1979), Clemens (1933), Forbes and Waters (1993), Gran and Angst (1931), Haigh et al. (1992), Hobson (1983), Hobson and McQuoid (2001), Horner et al. (2005), Légaré (1957), Phifer (1932), Roelofs (1983), Shim (1976), Stockner and Cliff (1975), Stockner and Cliff (1976), Waters et al. (1992) **Morphology:** WL: iNat (50001446), Hakai (Hakai_phyto_QU39_2016-04-28_1m_33) **Molecular (amplicon):** EBI (ENA:SRS969435), IMERSS (ENA:ERS27217975)

(M) *Leptocylindrus minimus* Gran

Records: 26 **Literature:** Buchanan (1966), Chester et al. (1979), Clemens (1933), Forbes and Waters (1993), Gran and Angst (1931), Horner et al. (2005), Phifer (1932), Shim (1976), Waters et al. (1992) **Morphology:** WL: iNat (50302063) **Molecular (amplicon):** EBI (ENA:SRS1690237)

(M) *Tenuicylindrus belgicus* (Meunier) D.Nanjappa & A.Zingone

Records: 28 **Molecular (amplicon):** EBI (ENA:SRS2340124), IMERSS (ENA:ERS27217975)

Phylum: Bacillariophyta

Class: Coscinodiscophyceae

Subclass: Chaetocerotophycidae

Order: Hemiaulales

Family: Hemiaulaceae

(M) *Cerataulina bicornis* (Ehrenberg) Hasle

Records: 49 **Morphology:** Hakai (Hakai_phyto_QU39_2018-10-04_1m_1) **Syn:** *Syringidium bicorne* Ehrenberg

(M) *Cerataulina pelagica* (Cleve) Hendey

Records: 76 **Literature:** Buchanan (1966), Chester et al. (1979), Clemens (1933), Forbes and Waters (1993), Gran and Angst (1931), Horner et al. (2005), Phifer (1932), Shim (1976), Stockner and Cliff (1975), Stockner and Cliff (1976), Stockner and Cliff (1979), Waters et al. (1992) **Morphology:** WL: iNat (9290781), Hakai (Hakai_phyto_QU39_2021-03-26_5m_48) **Molecular (amplicon):** IMERSS (ENA:ERS27217975) **Syn:** *Cerataulina bergonii* Ostenfeld

(M) *Eucampia groenlandica* Cleve

Records: 30 **Morphology:** Hakai (Hakai_phyto_QU39_2021-06-28_5m_58)

(M) *Eucampia zodiacus* Ehrenberg

Records: 70 **Literature:** Bailey and MacKay (1916), Buchanan (1966), Chester et al. (1979), Clemens (1933), Forbes and Waters (1993), Hobson and McQuoid (2001), Johnson (1931), Légaré (1957), Phifer (1932), Sancetta and Calvert (1988), Shim (1976), Stockner and Cliff (1975), Stockner and Cliff (1976), Waters et al. (1992) **Morphology:** WL: iNat (4848935), Hakai (Hakai_phyto_QU39_2021-07-27_5m_15) **Notes:** orth. corr. '*Ethmodiscus zodiacus*'

(M) *Hemiaulus chinensis* Greville

Records: 4 **Literature:** Shim (1976) **Morphology:** WL: iNat (201768692) **Molecular (amplicon):** EBI (ENA:SRS966615) **Syn:** *Hemiaulus sinensis* Greville

(M) *Hemiaulus hauckii* Grunow ex Van Heurck

Records: 2 **Morphology:** Hakai (Hakai_phyto_QU39_2019-09-04_5m_37)

Phylum: Bacillariophyta
Class: Coscinodiscophyceae
Subclass: Coscinodiscophycidae
Order: Arachnoidiscales
Family: Arachnoidiscaceae
(M) ***Arachnoidiscus ehrenbergii*** **Bailey**
Records: 8 **Literature:** Bailey and MacKay (1916), Clemens (1933), Gran and Angst (1931), Lord (1866), Shim (1976) **Morphology:** WL: iNat (248975017) Voucher (WL-SEM-5), M (184750 / 462228 / E 5570)
(M) ***Arachnoidiscus indicus*** **Ehrenberg**
Records: 1 **Literature:** Tempère and Peragallo (1908)

Phylum: Bacillariophyta
Class: Coscinodiscophyceae
Subclass: Coscinodiscophycidae
Order: Asterolamprales
Family: Asterolampraceae
(M) ***Asterolampra marylandica*** **Ehrenberg**
Records: 1 **Literature:** Buchanan (1966)
(M) ***Asteromphalus arachne*** **(Brébisson) Ralfs**
Records: 1 **Literature:** Bailey and MacKay (1916)
(M) ***Asteromphalus heptactis*** **(Brébisson) Ralfs**
Records: 12 **Literature:** Chester et al. (1979), Clemens (1933), Gran and Angst (1931), McQuoid and Hobson (1997), McQuoid and Hobson (2001), Phifer (1932), Sancetta and Calvert (1988), Shim (1976) **Morphology:** WL: iNat (4874751)
(M) ***Liriogramma*** **cf.** ***sarcophagus*** **(Wallich) D.Z.Lan**
Records: 1 **Literature:** Waters et al. (1992)

Phylum: Bacillariophyta
Class: Coscinodiscophyceae
Subclass: Coscinodiscophycidae
Order: Aulacoseirales
Family: Aulacoseiraceae
(F) ***Aulacoseira ambigua*** **(Grunow) Simonsen**
Records: 1 **Literature:** Roelofs (1983) **Syn:** *Melosira ambigua* (Grunow) O.F.Müller
(F) ***Aulacoseira crenulata*** **(Ehrenberg) Thwaites**
Records: 1 **Literature:** Tempère and Peragallo (1908) **Syn:** *Melosira crenulata* (Ehrenberg) Kützing
(F) ***Aulacoseira distans*** **(Ehrenberg) Simonsen**
Records: 1 **Literature:** Roelofs (1983) **Syn:** *Melosira distans* (Ehrenberg) Kützing
(F) ***Aulacoseira granulata*** **(Ehrenberg) Simonsen**
Records: 1 **Literature:** Roelofs (1983) **Syn:** *Melosira granulata* (Ehrenberg) Ralfs
(F) ***Aulacoseira granulata*** **var.** ***angustissima*** **(O.Müller) Simonsen**
Records: 1 **Literature:** Shim (1976) **Syn:** *Melosira granulata* var. *angustissima* O.Müller
(F) ***Aulacoseira islandica*** **(O.Müller) Simonsen**
Records: 2 **Literature:** Roelofs (1983), Shim (1976) **Syn:** *Melosira islandica* O.Müller
(F) ***Aulacoseira islandica*** **ssp.** ***helvetica*** **(O.Müller) Simonsen**
Records: 1 **Literature:** Shim (1976) **Syn:** *Melosira islandica* ssp. *helvetica* O.Müller
(F) ***Aulacoseira italica*** **(Ehrenberg) Simonsen**

Records: 2 **Literature:** Roelofs (1983), Shim (1976) **Syn:** *Melosira italica* (Ehrenberg) Kützing

(F) ***Aulacoseira subarctica*** **(O.Müller) E.Y.Haworth**

Records: 2 **Literature:** Shim (1976) **Morphology:** WL: iNat (262735091) **Syn:** *Melosira italica* ssp. *subarctica* O.Müller

Phylum: Bacillariophyta
Class: Coscinodiscophyceae
Subclass: Coscinodiscophycidae
Order: Biddulphiales
Family: Attheyaceae

(M) ***Attheya longicornis*** **R.M.Crawford & C.Gardner ✦**

Records: 1 **Morphology:** WL: iNat (263094504) Voucher (WL-SEM-113)

(M) ***Attheya septentrionalis*** **(Østrup) R.M.Crawford**

Records: 5 **Literature:** Buchanan (1966), Forbes and Waters (1993), Sutherland et al. (2023), Waters et al. (1992) **Molecular (amplicon):** EBI (ENA:JN162768) **Syn:** *Chaetoceros septentrionalis* Østrup

Phylum: Bacillariophyta
Class: Coscinodiscophyceae
Subclass: Coscinodiscophycidae
Order: Biddulphiales
Family: Biddulphiaceae

(M) ***Biddulphia biddulphiana*** **(J.E. Smith) Boyer**

Records: 3 **Literature:** Clemens (1933), Shim (1976) **Morphology:** WL: iNat (4876149) **Syn:** *Biddulphia pulchella* S.F.Gray, nom. illeg.

(M) ***Isthmia enervis*** **Ehrenberg ✦**

Records: 1 **Morphology:** WL: iNat (204882487) Voucher (WL-SEM-6)

(M) ***Isthmia nervosa*** **Kützing**

Records: 9 **Literature:** Clemens (1933), Lord (1866), Shim (1976), Tempère and Peragallo (1908) **Morphology:** WL: iNat (194751473) Voucher (WL-SEM-117)

(M) ***Leudugeria janischii*** **(Grunow) Tempère ex Van Heurck**

Records: 1 **Literature:** Bailey and MacKay (1916) **Syn:** *Euodia janischii* Grunow

(M) ***Neobrightwellia alternans*** **(Bailey) M.P.Ashworth & P.A.Sims**

Records: 4 **Literature:** Bailey and MacKay (1916), Rao and Lewin (1976), Tempère and Peragallo (1908), Tynni (1986) **Syn:** *Biddulphia alternans* (Bailey) Van Heurck, *Triceratium alternans* Bailey, *Trigonium alternans* (Bailey) A. Mann

Phylum: Bacillariophyta
Class: Coscinodiscophyceae
Subclass: Coscinodiscophycidae
Order: Corethrales
Family: Corethraceae

(M) ***Corethron hystrix*** **Hensen**

Records: 16 **Literature:** Bailey and MacKay (1916), Chester et al. (1979), Clemens (1933), Gran and Angst (1931), Horner et al. (2005), Légaré (1957), Phifer (1932), Stockner and Cliff (1975), Stockner and Cliff (1976) **Morphology:** WL: iNat (49988677), MBA (265AT-1-10681)

(M) ***Corethron pennatum*** **(Grunow) Ostenfeld**

Records: 87 **Literature:** Forbes and Waters (1993), Haigh et al. (1992), Shim (1976) **Morphology:** Hakai (Hakai_phyto_QU39_2021-12-14_5m_1) **Syn:** *Corethron criophilum* Castracane

Phylum: Bacillariophyta
Class: Coscinodiscophyceae
Subclass: Coscinodiscophycidae
Order: Coscinodiscales
Family: Aulacodiscaceae
(M) ***Aulacodiscus kittonii*** **Arnott ex Ralfs**
Records: 2 **Literature:** Shim (1976) **Morphology:** WL: iNat (264541292)
(M) ***Aulacodiscus oregonus*** **Harvey & Bailey**
Records: 17 **Literature:** Bailey and MacKay (1916), Clemens (1933), Gran and Angst (1931), Tempère and Peragallo (1908) **Molecular (amplicon):** IMERSS (ENA:ERS27214058)

Phylum: Bacillariophyta
Class: Coscinodiscophyceae
Subclass: Coscinodiscophycidae
Order: Coscinodiscales
Family: Coscinodiscaceae
(M) ***Coscinodiscus apiculatus*** **Ehrenberg**
Records: 1 **Literature:** Waters et al. (1992)
(M) ***Coscinodiscus argus*** **Ehrenberg**
Records: 1 **Literature:** Rao and Lewin (1976)
(M) ***Coscinodiscus asteromphalus*** **Ehrenberg**
Records: 8 **Literature:** Bailey and MacKay (1916), Chester et al. (1979), Peck and Harrington (1897), Shim (1976), Tempère and Peragallo (1908), Tynni (1986), Waters et al. (1992) **Morphology:** WL: iNat (257886598)
(M) ***Coscinodiscus asteromphalus*** **var.** ***hybridus*** **Grunow**
Records: 1 **Literature:** Tempère and Peragallo (1908) **Notes:** orth. corr. '*Coscinodiscus asteromphalus* var. *hybrida*'
(M) ***Coscinodiscus centralis*** **Ehrenberg**
Records: 5 **Literature:** Bailey and MacKay (1916), Buchanan (1966), Chester et al. (1979), Horner et al. (2005), Johnson (1931)
(M) ***Coscinodiscus centralis*** **var.** ***pacifica*** **Gran & Angst**
Records: 6 **Literature:** Chester et al. (1979), Clemens (1933), Gran and Angst (1931), Phifer (1932), Roelofs (1983), Shim (1976)
(M) ***Coscinodiscus concinnus*** **W.Smith**
Records: 9 **Literature:** Bailey and MacKay (1916), Buchanan (1966), Chester et al. (1979), Clemens (1933), Gran and Angst (1931), Gucluer and Gross (1964), McQuoid and Hobson (1997), Phifer (1932) **Morphology:** MBA (92AT-5-165)
(M) ***Coscinodiscus curvatulus*** **Grunow**
Records: 12 **Literature:** Bailey and MacKay (1916), Buchanan (1966), Chester et al. (1979), Clemens (1933), Gran and Angst (1931), Gucluer and Gross (1964), McQuoid and Hobson (1997), Phifer (1932), Rao and Lewin (1976), Roelofs (1983), Shim (1976) **Morphology:** WL: iNat (294137955)
(M) ***Coscinodiscus curvatulus*** **var.** ***minor*** **(Ehrenberg) Grunow**
Records: 1 **Literature:** Roelofs (1983)
(M) ***Coscinodiscus debilis*** **Grove**
Records: 1 **Literature:** Bailey and MacKay (1916)
(M) ***Coscinodiscus divisus*** **Grunow**
Records: 3 **Literature:** Bailey and MacKay (1916), Clemens (1933), Rao and Lewin (1976)
(M) ***Coscinodiscus gigas*** **Ehrenberg**
Records: 4 **Literature:** McQuoid and Hobson (1997), McQuoid and Hobson (2001), Sancetta and Calvert (1988), Shim (1976)
(M) ***Coscinodiscus granii*** **L.F. Gough**

Records: 34 **Literature:** Buchanan (1966), Chester et al. (1979), Clemens (1933), Forbes and Waters (1993), Gucluer and Gross (1964), Hobson and McQuoid (2001), McQuoid and Hobson (1997), McQuoid and Hobson (2001), Phifer (1932), Shim (1976) **Morphology:** WL: iNat (9290897) **Molecular (amplicon):** EBI (ENA:SRS966881), IMERSS (ENA:ERS27217975); **Molecular (Sanger):** IMERSS (ENA: ERZ29258993)

(M) ***Coscinodiscus griseus*** **Greville**
Records: 1 **Literature:** Bailey and MacKay (1916)

(M) ***Coscinodiscus marginatolineatus*** **A.W.F.Schmidt**
Records: 1 **Literature:** Shim (1976)

(M) ***Coscinodiscus marginatus*** **Ehrenberg**
Records: 5 **Literature:** Chester et al. (1979), Clemens (1933), Roelofs (1983), Shim (1976) **Morphology:** WL: iNat (225592076) Voucher (WL-LM-9)

(M) ***Coscinodiscus obscurus*** **A.W.F.Schmidt**
Records: 2 **Literature:** Roelofs (1983), Shim (1976)

(M) ***Coscinodiscus oculus-iridis*** **(Ehrenberg) Ehrenberg**
Records: 6 **Literature:** Bailey and MacKay (1916), Chester et al. (1979), Peck and Harrington (1897), Roelofs (1983), Tempère and Peragallo (1908), Waters et al. (1992) **Notes:** orth. corr. '*Coxinodiscus ovulus iridis*'

(M) ***Coscinodiscus patera*** **Castracane**
Records: 1 **Literature:** Bailey and MacKay (1916)

(M) ***Coscinodiscus pavillardii*** **Forti**
Records: 1 **Literature:** Roelofs (1983) **Syn:** *Coscinodiscus perforatus* var. *pavillardii* (Forti) Hustedt (as '*pavillardi*')

(M) ***Coscinodiscus perforatus*** **Ehrenberg**
Records: 4 **Literature:** Clemens (1933), Roelofs (1983), Shim (1976), Tynni (1986)

(M) ***Coscinodiscus radiatus*** **Ehrenberg**
Records: 48 **Literature:** Bailey and MacKay (1916), Buchanan (1966), Chester et al. (1979), Clemens (1933), Lord (1866), McQuoid and Hobson (1997), McQuoid and Hobson (2001), Rao and Lewin (1976), Roelofs (1983), Sancetta and Calvert (1988), Shim (1976), Tynni (1986), Waters et al. (1992) **Morphology:** WL: iNat (40702227) **Molecular (amplicon):** EBI (ENA:FJ864276), IMERSS (ENA:ERS27217975) **Notes:** orth. corr. '*Coxinodiscus radiatus*'

(M) ***Coscinodiscus radiosus*** **Grunow**
Records: 1 **Literature:** Bailey and MacKay (1916)

(M) ***Coscinodiscus rothii*** **(Ehrenberg) Grunow**
Records: 4 **Literature:** Bailey and MacKay (1916), Clemens (1933), Rao and Lewin (1976), Shim (1976)

(M) ***Coscinodiscus subbulliens*** **Jørgensen**
Records: 1 **Literature:** Clemens (1933)

(M) ***Coscinodiscus sublineatus*** **Grunow**
Records: 1 **Literature:** Rao and Lewin (1976)

(M) ***Coscinodiscus subtilis*** **Ehrenberg**
Records: 2 **Literature:** Bailey and MacKay (1916), Lord (1866) **Notes:** orth. corr. '*Coxinodiscus subtilis*'

(M) ***Coscinodiscus wailesii*** **Gran & Angst**
Records: 16 **Literature:** Buchanan (1966), Chester et al. (1979), Clemens (1933), Gran and Angst (1931), Horner et al. (2005), Johnson (1931), Légaré (1957), Phifer (1932), Roelofs (1983), Shim (1976) **Morphology:** WL: iNat (44884072), MBA (154AT-5-976)

Phylum: Bacillariophyta
Class: Coscinodiscophyceae
Subclass: Coscinodiscophycidae
Order: Coscinodiscales
Family: Heliopeltaceae

(M) *Actinoptychus adriaticus* Grunow
Records: 1 **Literature:** Buchanan (1966)

(M) *Actinoptychus adriaticus* var. *pumila* Grunow ✦
Records: 1 **Morphology:** WL: iNat (185626241) Voucher (WL-SEM-90)

(M) *Actinoptychus areolatus* (Ehrenberg) A.W.F.Schmidt
Records: 1 **Literature:** Tempère and Peragallo (1908)

(F) *Actinoptychus maculatus* A.W.F.Schmidt
Records: 1 **Literature:** Shim (1976)

(M) *Actinoptychus senarius* (Ehrenberg) Ehrenberg
Records: 26 **Literature:** Bailey and MacKay (1916), Buchanan (1966), Chester et al. (1979), Clemens (1933), Gran and Angst (1931), Gucluer and Gross (1964), Horner et al. (2005), Lord (1866), McQuoid and Hobson (1997), McQuoid and Hobson (2001), Phifer (1932), Rao and Lewin (1976), Roelofs (1983), Shim (1976), Tempère and Peragallo (1908), Tynni (1986) **Morphology:** WL: iNat (4874664), NHMUK (Diatom BM Adams TS.629) **Molecular (amplicon):** EBI (ENA:SRS966986) **Syn:** *Actinoptychus undulatus* A.Schmidt

(M) *Actinoptychus splendens* (Shadbolt) Ralfs
Records: 10 **Literature:** Buchanan (1966), Chester et al. (1979), Clemens (1933), Gran and Angst (1931), McQuoid and Hobson (1997), McQuoid and Hobson (2001), Roelofs (1983), Sancetta and Calvert (1988), Shim (1976) **Morphology:** WL: iNat (187951430) Voucher (WL-SEM-85, WL-LM-8) **Syn:** *Actinosphaenia splendens* Shadbolt

(M) *Actinoptychus vulgaris* Schumann
Records: 1 **Literature:** Tempère and Peragallo (1908)

Phylum: Bacillariophyta
Class: Coscinodiscophyceae
Subclass: Coscinodiscophycidae
Order: Coscinodiscales
Family: Hemidiscaceae

(M) *Actinocyclus curvatulus* Janisch
Records: 3 **Literature:** McQuoid and Hobson (2001), Sancetta and Calvert (1988), Tempère and Peragallo (1908)

(M) *Actinocyclus kuetzingii* (A.W.F.Schmidt) Simonsen
Records: 4 **Literature:** Bailey and MacKay (1916), Clemens (1933), Rao and Lewin (1976), Shim (1976) **Syn:** *Coscinodiscus kuetzingii* A.W.F.Schmidt (as '*kützingii*')

(M) *Actinocyclus nebulosus* M.Peragallo
Records: 1 **Literature:** Tempère and Peragallo (1908)

(M) *Actinocyclus normanii* (W.Gregory ex Greville) Hustedt
Records: 1 **Literature:** Shim (1976) **Syn:** *Coscinodiscus normanii* W.Gregory ex Greville

(M) *Actinocyclus octonarius* Ehrenberg
Records: 3 **Literature:** Clemens (1933), Gran and Angst (1931) **Morphology:** WL: iNat (141130114) Voucher (WL-LM-2) **Syn:** *Actinocyclus ehrenbergii* Ralfs

(M) *Actinocyclus octonarius* var. *tenellus* (Brébisson) Hendey
Records: 2 **Literature:** Tempère and Peragallo (1908) **Morphology:** WL: iNat (325528270) **Syn:** *Actinocyclus tenellus* Brébisson

(M) *Actinocyclus subtilis* (W.Gregory) Ralfs
Records: 4 **Literature:** Bailey and MacKay (1916), Clemens (1933), Lord (1866), Roelofs (1983)

Phylum: Bacillariophyta
Class: Coscinodiscophyceae
Subclass: Coscinodiscophycidae
Order: Eupodiscales
Family: Eupodiscaceae
(M) *Amphipentas pentacrinus* Ehrenberg
Records: 1 **Literature:** Rao and Lewin (1976) **Notes:** Rao and Lewin (1976) report the unrecognized *Triceratium pentacrinus* (Ehrenberg) Wallich f. *quadratum* Hustedt **Syn:** *Triceratium pentacrinus* (Ehrenberg) Wallich;
(M) *Cerataulus granulatus* P.A.Sims & D.M.Williams
Records: 3 **Literature:** Bailey and MacKay (1916), Clemens (1933), Tempère and Peragallo (1908) **Syn:** *Biddulphia granulata* Roper, nom. illeg.
(M) *Pleurosira laevis* (Ehrenberg) Compère
Records: 6 **Literature:** Clemens (1933), Gran and Angst (1931), Johnson (1931), Légaré (1957), Lord (1866), Phifer (1932) **Syn:** *Biddulphia laevis* Ehrenberg
(M) *Zygoceros rhombus* Ehrenberg
Records: 2 **Literature:** Clemens (1933) **Morphology:** MBA (230VJ-5-161) **Syn:** *Biddulphia rhombus* (Ehrenberg) W.Smith

Phylum: Bacillariophyta
Class: Coscinodiscophyceae
Subclass: Coscinodiscophycidae
Order: Eupodiscales
Family: Odontellaceae
(M) *Amphitetras antediluviana* Ehrenberg
Records: 4 **Literature:** Bailey and MacKay (1916), Lord (1866), Shim (1976), Tempère and Peragallo (1908) **Syn:** *Biddulphia antediluviana* (Ehrenberg) Van Heurck, *Triceratium antediluvianum* (Ehrenberg) Grunow
(M) *Hobaniella longicruris* (Greville) P.A.Sims & D.M.Williams
Records: 67 **Literature:** Buchanan (1966), Chester et al. (1979), Clemens (1933), Forbes and Waters (1993), Gran and Angst (1931), Horner et al. (2005), Johnson (1931), Légaré (1957), McQuoid and Hobson (1997), McQuoid and Hobson (2001), Phifer (1932), Roelofs (1983), Shim (1976), Stockner and Cliff (1976), Tempère and Peragallo (1908) **Morphology:** WL: iNat (4873117), Hakai (Hakai_phyto_QU39_2021-05-19_5m_10) **Molecular (amplicon):** EBI (ENA:SRS966872), IMERSS (ENA:ERS27217975) **Syn:** *Biddulphia longicuris* Greville, *Biddulphia longicruris* var. *hyalina* (J.L.B.Schröder) Cupp, *Odontella longicruris* (Greville) M.A.Hoban
(M) *Odontella aurita* (Lyngbye) C.Agardh
Records: 77 **Literature:** Bailey and MacKay (1916), Buchanan (1966), Chester et al. (1979), Clemens (1933), Gran and Angst (1931), Gucluer and Gross (1964), Horner et al. (2005), Johnson (1931), Légaré (1957), Lord (1866), McQuoid and Hobson (1997), McQuoid and Hobson (2001), Phifer (1932), Roelofs (1983), Sancetta and Calvert (1988), Shim (1976), Tempère and Peragallo (1908), Tynni (1986), Waters et al. (1992) **Morphology:** WL: iNat (50022943), MBA (268AT-1-157) **Molecular (amplicon):** EBI (ENA:SRS966853), IMERSS (ENA:ERS21395352) **Notes:** since Lord (1866), a very commonly reported diatom that has undergone numerous names and redefinitions to the early 1990s **Syn:** *Biddulphia aurita* (Lyngbye) Brébisson
(M) *Odontella edwardsii* (Febiger ex Grunow) Grunow

Records: 1 **Literature:** Tempère and Peragallo (1908) **Syn:** *Biddulphia edwardsii* Febiger ex Grunow

(M) ***Odontella litigiosa*** **(Van Heurck) Hoban**

Records: 2 **Literature:** Bailey and MacKay (1916), Clemens (1933) **Syn:** *Biddulphia polymorpha* (Montagne) Wolle, *Cerataulus polymorphus* (Montagne) Van Heurck

(M) ***Odontella obtusa*** **Kützing**

Records: 7 **Literature:** Bailey and MacKay (1916), Buchanan (1966), Clemens (1933), Rao and Lewin (1976), Shim (1976), Tempère and Peragallo (1908) **Morphology:** WL: iNat (203564017) Voucher (WL-SEM-47) **Syn:** *Biddulphia aurita* var. *obtusa* (Kützing) Denys, *Biddulphia obtusa* (Kützing) Ralfs, *Biddulphia roperiana* Greville,

(M) ***Odontella turgida*** **(Ehrenberg) Kützing**

Records: 4 **Literature:** Bailey and MacKay (1916), Clemens (1933), Tempère and Peragallo (1908), Tynni (1986) **Syn:** *Biddulphia turgida* W. Smith, *Cerataulus turgidus* (Ehrenberg) Ehrenberg, *Denticella turgida* Ehrenberg

(M) ***Pseudictyota dubia*** **(Brightwell) P.A.Sims & D.M.Williams**

Records: 1 **Literature:** Shim (1976) **Syn:** *Biddulphia dubia* (Brightwell) Cleve

Phylum: Bacillariophyta

Class: Coscinodiscophyceae

Subclass: Coscinodiscophycidae

Order: Eupodiscales

Family: Parodontellaceae

(M) ***Trieres mobiliensis*** **(J.W.Bailey) Ashworth & E.C.Theriot**

Records: 2 **Literature:** Clemens (1933), Gran and Angst (1931)

Phylum: Bacillariophyta

Class: Coscinodiscophyceae

Subclass: Coscinodiscophycidae

Order: Lithodesmiales

Family: Bellerocheaceae

(M) ***Bellerochea malleus*** **(Brightwell) Van Heurck**

Records: 1 **Literature:** Chester et al. (1979)

Phylum: Bacillariophyta

Class: Coscinodiscophyceae

Subclass: Coscinodiscophycidae

Order: Lithodesmiales

Family: Lithodesmiaceae

(M) ***Ditylum brightwellii*** **(T. West) Grunow**

Records: 227 **Literature:** Bailey and MacKay (1916), Buchanan (1966), Chester et al. (1979), Clemens (1933), Forbes and Waters (1993), Gran and Angst (1931), Gucluer and Gross (1964), Haigh et al. (1992), Hobson and McQuoid (2001), Horner et al. (2005), Johnson (1931), Légaré (1957), McQuoid and Hobson (1997), McQuoid and Hobson (2001), Phifer (1932), Roelofs (1983), Sancetta and Calvert (1988), Shim (1976), Stockner and Cliff (1975), Stockner and Cliff (1976), Waters et al. (1992) **Morphology:** WL: iNat (4848365), Hakai (Hakai_phyto_QU39_2021-07-27_5m_14), MBA (253AT-5-168) **Molecular (amplicon):** EBI (ENA:SRS1690262), IMERSS (WL-18S-I5); **Molecular (Sanger):** IMERSS (ENA: ERZ29258993)

(M) ***Lithodesmium undulatum*** **Ehrenberg**

Records: 5 **Literature:** Bailey and MacKay (1916), Clemens (1933), Gran and Angst (1931), Shim (1976), Waters et al. (1992) **Syn:** *Triceratium undulatum* Brightwell

(M) ***Tropidoneis maxima*** **(W.Gregory) Cleve**

Records: 1 **Literature:** Bailey and MacKay (1916) **Syn:** *Amphiprora maxima* W.Gregory

Phylum: Bacillariophyta
Class: Coscinodiscophyceae
Subclass: Coscinodiscophycidae
Order: Stellarimales
Family: Stellarimaceae
(M) ***Stellarima microtrias*** **(Ehrenberg) G.R.Hasle & P.A.Sims**
Records: 6 **Molecular (amplicon):** EBI (ENA:SRS2340126)
(M) ***Stellarima stellaris*** **(Roper) G.R.Hasle & P.A.Sims**
Records: 6 **Literature:** Bailey and MacKay (1916), Chester et al. (1979), Phifer (1932), Roelofs (1983), Sancetta and Calvert (1988), Shim (1976) **Syn:** *Coscinodiscus stellaris* Roper

Phylum: Bacillariophyta
Class: Coscinodiscophyceae
Subclass: Coscinodiscophycidae
Order: Stellarimales
Family: Trigoniaceae
(M) ***Trigonium arcticum*** **(Brightwell) Cleve**
Records: 10 **Literature:** Bailey and MacKay (1916), Clemens (1933), Gran and Angst (1931), Shim (1976), Tempère and Peragallo (1908) **Morphology:** WL: iNat (50128873) Voucher (WL-SEM-1,WL-SEM-8) **Syn:** *Biddulphia arctica* (Brightwell) Boyer, *Triceratium arcticum* Brightwell
(M) ***Trigonium arcticum*** **var.** ***quadratum*** **(Grunow ex Tempère & H.Paragallo) Desikachary & Desikachary**
Records: 1 **Literature:** Tempère and Peragallo (1908) **Syn:** *Triceratium arcticum* f. *quadratum* Grunow ex Tempère & H.Paragallo (as '*quadrata*')
(M) ***Trigonium quinquelobatum*** **(Greville) A.Mann ✦✦**
Records: 1 **Morphology:** WL: iNat (300280287) Voucher (WL-SEM-102, WL-LM-47) **Syn:** *Triceratium quinquelobatum* Greville, *Trigonium arcticum* var. *quinquelobatum* (Greville) Desikachary & Ranjitha

Phylum: Bacillariophyta
Class: Coscinodiscophyceae
Subclass: Cymatosirophycidae
Order: Cymatosirales
Family: Cymatosiraceae
(M) ***Arcocellulus cornucervis*** **Hasle, Stosch & Syvertsen**
Records: 2 **Molecular (amplicon):** IMERSS (ENA:ERS27630061)
(M) ***Extubocellulus spinifer*** **(Hargreaves & Guillard) Hasle, Stosch & Syvertsen ✦**
Records: 3 **Morphology:** WL: iNat (311561542) Voucher (WL-SEM-93) **Molecular (amplicon):** IMERSS (ENA:ERS21395345)
(M) ***Campylosira cymbelliformis*** **(A.W.F.Schmidt) Grunow ex Van Heurck**
Records: 1 **Literature:** Roelofs (1983)
(M) ***Leyanella arenaria*** **G.R.Hasle, H.A.von Stosch & E.E.Syvertsen**
Records: 2 **Literature:** Forbes and Waters (1993), Waters et al. (1992)
(M) ***Plagiogrammopsis vanheurckii*** **(Grunow) Hasle, Stosch & Syvertsen**
Records: 3 **Literature:** McQuoid and Hobson (1997), McQuoid and Hobson (2001), Shim (1976) **Syn:** *Plagiogramma vanheurckii* Grunow

Phylum: Bacillariophyta
Class: Coscinodiscophyceae
Subclass: Melosirophycidae
Order: Melosirales
Family: Hyalodiscaceae

(M) *Hyalodiscus laevis* Ehrenberg
Records: 4 **Literature:** Bailey and MacKay (1916), Lord (1866), Tempère and Peragallo (1908) **Morphology:** M (184908 / 463128 / E 6386)

(M) *Hyalodiscus scoticus* (Kützing) Grunow
Records: 9 **Literature:** Tempère and Peragallo (1908) **Morphology:** WL: iNat (144093594) Voucher (WL-SEM-24, WL-LM-39) **Syn:** *Hyalodiscus subtilis* Bailey

(M) *Hyalodiscus whitneyi* Ehrenberg
Records: 1 **Literature:** Bailey and MacKay (1916)

(M) *Podosira hormoides* (Montagne) Kützing
Records: 2 **Literature:** Lord (1866), Tempère and Peragallo (1908)

(M) *Podosira maxima* (Kützing) Grunow
Records: 1 **Literature:** Tempère and Peragallo (1908)

(M) *Podosira stelligera* (Bailey) A.Mann
Records: 1 **Literature:** Clemens (1933) **Molecular (amplicon):** IMERSS (ENA:ERS21395346) **Syn:** *Hyalodiscus stelliger* Bailey

Phylum: Bacillariophyta
Class: Coscinodiscophyceae
Subclass: Melosirophycidae
Order: Melosirales
Family: Melosiraceae

(M) *Melosira* cf. *octogona* A.W.F.Schmidt
Records: 4 **Molecular (amplicon):** EBI (ENA:ERS2393289)

(M) *Melosira hyperborea* Grunow
Records: 1 **Literature:** Waters et al. (1992) **Syn:** *Melosira arctica* Dickiesyn or *Melosira hyperborea* Schütt, nom. illeg. (authority not specified)

(M) *Melosira inflexa* (Roth) Guiry
Records: 15 **Literature:** Bailey and MacKay (1916), Buchanan (1966), Clemens (1933), Gran and Angst (1931), Johnson (1931), Légaré (1957), McQuoid and Hobson (1997), McQuoid and Hobson (2001), Roelofs (1983), Shim (1976), Stockner and Cliff (1975), Stockner and Cliff (1976), Tynni (1986) **Morphology:** WL: iNat (229272465) **Syn:** *Melosira borreri* Greville, *Melosira moniliformis* C.Agardh

(B) *Melosira lineata* (Dillwyn) C.Agardh
Records: 7 **Literature:** Bailey and MacKay (1916), Buchanan (1966), Clemens (1933), Gran and Angst (1931), Stockner and Cliff (1976), Tynni (1986), Waters et al. (1992) **Syn:** *Melosira juergensii* C.Agardh (as '*jurgensii*' by Clemens 1933)

(F) *Melosira moniliformis* var. *hispida* (Castracane) Hustedt
Records: 1 **Literature:** Tynni (1986)

(M) *Melosira nummuloides* C. Agardh.
Records: 18 **Literature:** Bailey and MacKay (1916), Buchanan (1966), Clemens (1933), Lord (1866), Shim (1976), Stockner and Cliff (1976), Waters et al. (1992) **Morphology:** WL: iNat (4873142) **Molecular (amplicon):** IMERSS (ENA:ERS21395345)

(F) *Melosira undulata* (Ehrenberg) Kützing
Records: 2 **Literature:** Clemens (1933), Tempère and Peragallo (1908)

(F) *Melosira varians* C.Agardh
Records: 2 **Literature:** Bailey and MacKay (1916), Shim (1976)

Phylum: Bacillariophyta
Class: Coscinodiscophyceae
Subclass: Paraliophycidae
Order: Paraliales
Family: Paraliaceae

(M) *Paralia longispina* S.Konno & R.W.Jordan
Records: 15 **Molecular (amplicon):** EBI (ENA:SRS2340157), IMERSS (ENA:ERS21395368)

(M) *Paralia marina* (W.Smith) Heiberg
Records: 2 **Literature:** Lord (1866), McQuoid and Hobson (1997) **Syn:** *Melosira marina* (W.Smith) Janisch

(M) *Paralia sulcata* (Ehrenberg) Cleve
Records: 45 **Literature:** Bailey and MacKay (1916), Buchanan (1966), Chester et al. (1979), Clemens (1933), Forbes and Waters (1993), Gran and Angst (1931), Gucluer and Gross (1964), Horner et al. (2005), Légaré (1957), McQuoid and Hobson (2001), Phifer (1932), Powys (1987), Roelofs (1983), Sancetta and Calvert (1988), Shim (1976), Tempère and Peragallo (1908), Tynni (1986), Waters et al. (1992) **Morphology:** WL: iNat (4873115), Hakai (Hakai_phyto_QU39_2021-12-08_5m_4), MBA (250AT-5-101) **Molecular (amplicon):** EBI (ENA:SRS2340122) **Syn:** *Melosira sulcata* (Ehrenberg) Kützing

(M) *Paralia sulcata* var. *coronata* (Ehrenberg) G.W.Andrews
Records: 2 **Literature:** Bailey and MacKay (1916), Tempère and Peragallo (1908) **Syn:** *Melosira sulcata* f. *coronata* (Ehrenberg) Peragallo & Peragallo (as '*Melosira sulcata* var. *genuina* f. *coronata*')

Phylum: Bacillariophyta
Class: Coscinodiscophyceae
Subclass: Paraliophycidae
Order: Paraliales
Family: Radialiplicataceae

(F) *Ellerbeckia clavigera* (Grunow) Crawford & Sims
Records: 1 **Literature:** Tynni (1986) **Syn:** *Melosira polaris* Grunow

(M) *Ellerbeckia sol* (Ehrenberg) R.M.Crawford & P.A.Sims
Records: 8 **Literature:** Bailey and MacKay (1916), Clemens (1933), Roelofs (1983), Shim (1976), Tempère and Peragallo (1908) **Morphology:** WL: iNat (190960048) Voucher (WL-SEM-20, WL-SEM-36, WL-LM-4) **Syn:** *Melosira sol* (Ehrenberg) Kützing

Phylum: Bacillariophyta
Class: Coscinodiscophyceae
Subclass: Rhizosoleniophycidae
Order: Probosciales
Family: Probosciaceae

(M) *Proboscia alata* (Brightwell) Sundström
Records: 9 **Literature:** Chester et al. (1979), Gran and Angst (1931), Hobson and McQuoid (2001), Hollibaugh et al. (1980), Johnson (1931), Phifer (1932), Shim (1976) **Morphology:** MBA (250AT-5-1000) **Syn:** *Rhizosolenia alata* Brightwell, *Rhizosolenia alata* f. *gracillima* (Cleve) Grunow

(M) *Proboscia subarctica* K.Takahashi, R.W.Jordan & J.Priddle
Records: 2 **Literature:** Clemens (1933), Shim (1976) **Notes:** *P. subarctica* is a replacement name for *Rhizosolenia alata* f. *curvirostris* Gran, that epithet being blocked at species rank in *Proboscia* by *P. curvirostris* (A.P.Jousé) R.W.Jordan & J.Priddle **Syn:** *Rhizosolenia alata* f. *curvirostris*

Phylum: Bacillariophyta

Class: Coscinodiscophyceae

Subclass: Rhizosoleniophycidae

Order: Rhizosoleniales

Family: Rhizosoleniaceae

(M) *Dactyliosolen blavyanus* (H.Peragallo) Hasle

Records: 1 **Literature:** Horner et al. (2005)

(M) *Dactyliosolen fragilissimus* (Bergon) Hasle

Records: 9 **Literature:** Buchanan (1966), Clemens (1933), Forbes and Waters (1993), Hobson and McQuoid (2001), Horner et al. (2005), Phifer (1932), Shim (1976), Waters et al. (1992) **Morphology:** WL: iNat (50047033) **Syn:** *Rhizosolenia fragilissima* Bergon

(M) *Dactyliosolen mediterraneus* (H.Peragallo) H.Peragallo

Records: 4 **Literature:** Buchanan (1966), Clemens (1933), Gran and Angst (1931), Shim (1976) **Syn:** *Leptocylindrus mediterraneus* (H.Peragallo) Hasle

(M) *Dactyliosolen phuketensis* (B.G.Sundström) G.R.Hasle

Records: 89 **Morphology:** Hakai (Hakai_phyto_QU39_2017-10-11_1m_15) **Syn:** *Rhizosolenia phuketensis* B.G.Sundström

(M) *Guinardia delicatula* (Cleve) Hasle

Records: 118 **Literature:** Buchanan (1966), Chester et al. (1979), Clemens (1933), Forbes and Waters (1993), Gran and Angst (1931), Hobson and McQuoid (2001), Horner et al. (2005), Johnson (1931), Légaré (1957), Phifer (1932), Shim (1976), Waters et al. (1992) **Morphology:** WL: iNat (4873172), Hakai (Hakai_phyto_QU39_2021-07-27_5m_16) **Molecular (amplicon):** IMERSS (ENA:ERS27217975) **Syn:** *Rhizosolenia delicatula* Cleve

(M) *Guinardia flaccida* (Castracane) H.Peragallo

Records: 1 **Literature:** Shim (1976)

(M) *Guinardia striata* (Stolterfoth) Hasle

Records: 12 **Literature:** Buchanan (1966), Chester et al. (1979), Clemens (1933), Gran and Angst (1931), Hollibaugh et al. (1980), Johnson (1931), Légaré (1957), Phifer (1932), Stockner and Cliff (1975), Stockner and Cliff (1976), Waters et al. (1992) **Morphology:** WL: iNat (4873173) **Syn:** *Rhizosolenia stolterfothii* H.Peragallo

(M) *Neocalyptrella robusta* (G.Norman ex Ralfs) Hernández-Becerril & Meave ✦

Records: 1 **Morphology:** WL: iNat (48460936) Voucher (WL-SEM-66-7, WL-LM-50)

(M) *Pseudosolenia calcar-avis* (Schultze) B.G.Sundström

Records: 1 **Literature:** Shim (1976) **Syn:** *Rhizosolenia calcar*-avis Schultze (as '*calcar avis*')

(M) *Rhizosolenia hebetata* J.W.Bailey

Records: 3 **Literature:** Bailey and MacKay (1916), Buchanan (1966), Clemens (1933)

(M) *Rhizosolenia hebetata* f. *semispina* (Hensen) Gran

Records: 13 **Literature:** Chester et al. (1979), Clemens (1933), Forbes and Waters (1993), Gran and Angst (1931), Johnson (1931), Légaré (1957), Phifer (1932), Sancetta and Calvert (1988), Shim (1976) **Morphology:** MBA (154AT-1-108)

(M) *Rhizosolenia imbricata* Brightwell

Records: 2 **Molecular (amplicon):** EBI (ENA:SRS966492)

(M) *Rhizosolenia simplex* G.Karsten

Records: 1 **Literature:** Chester et al. (1979)

(M) *Rhizosolenia styliformis* T.Brightwell

Records: 8 **Literature:** Bailey and MacKay (1916), Clemens (1933), Gran and Angst (1931), Légaré (1957), Phifer (1932), Sancetta and Calvert (1988) **Morphology:** MBA (37AT-41-107)

(M) *Sundstroemia pungens* (A.Cleve) Medlin, Lundholm, Boonprakob & Moestrup ✦

Records: 6 **Morphology:** WL: iNat (4873038) **Molecular (amplicon):** EBI (ENA:SRS969492) **Syn:** *Rhizosolenia pungens* A.Cleve

(M) *Sundstroemia setigera* (Brightwell) Medlin

Records: 1516 **Literature:** Bailey and MacKay (1916), Buchanan (1966), Chester et al. (1979), Clemens (1933), Gran and Angst (1931), Gucluer and Gross (1964), Hobson and McQuoid (2001), Horner et al. (2005), Phifer (1932), Sancetta and Calvert (1988), Shim (1976), Stockner and Cliff (1976), Waters et al. (1992) **Morphology:** Hakai (Hakai_phyto_QU39_2021-07-27_5m_18), MBA (253AT-5-187), PSF (GO1-8-31-2021-0-rhise) **Molecular (amplicon):** EBI (ENA:SRS966454), IMERSS (ENA:ERS27217975) **Syn:** *Rhizosolenia setigera* Brightwell

(M) *Sundstroemia similoides* (A.Cleve) Medlin, Lundholm, Boonprakob & Moestrup

Records: 1 **Molecular (amplicon):** EBI (ENA:SRS966492) **Syn:** *Rhizosolenia similoides* A.Cleve

Phylum: Bacillariophyta
Class: Coscinodiscophyceae
Subclass: Thalassiosirophycidae
Order: Stephanodiscales
Family: Stephanodiscaceae

(M) *Cyclotella baltica* (Grunow) Håkansson ✦

Records: 1 **Morphology:** WL: iNat (265283095) Voucher (WL-SEM-91)

(M) *Cyclotella caspia* Grunow

Records: 4 **Literature:** Buchanan (1966), Forbes and Waters (1993), Sancetta (1989), Sancetta (1990) **Notes:** reported as '*Cyclotella* cf. *caspia*' by Buchanan (1966)

(FM) *Cyclotella litoralis* Lange & Syvertsen

Records: 17 **Literature:** McQuoid and Hobson (1997), McQuoid and Hobson (2001), Sancetta (1990) **Morphology:** WL: iNat (49991081) **Molecular (amplicon):** IMERSS (ENA:ERS27214058)

(F) *Cyclotella striata* (Kützing) Grunow

Records: 6 **Literature:** Bailey and MacKay (1916), Clemens (1933), Roelofs (1983), Shim (1976), Tempère and Peragallo (1908), Tynni (1986)

(F) *Discostella stelligera* (Cleve & Grunow) Houk & Klee

Records: 1 **Literature:** Roelofs (1983) **Syn:** *Cyclotella stelligera* (Cleve & Grunow) Van Heurck

(F) *Lindavia comta* (Kützing) T.Nakov & al.

Records: 2 **Literature:** Roelofs (1983), Shim (1976) **Syn:** *Cyclotella comta* Kützing

(F) *Pantocsekiella ocellata* (Pantocsek) K.T.Kiss & Ács

Records: 1 **Literature:** Roelofs (1983) **Syn:** *Cyclotella ocellata* Pantocsek

(F) *Stephanodiscus astraea* (Kützing) Grunow

Records: 1 **Literature:** Roelofs (1983)

Phylum: Bacillariophyta
Class: Coscinodiscophyceae
Subclass: Thalassiosirophycidae
Order: Thalassiosirales
Family: Lauderiaceae

(M) *Lauderia annulata* Cleve

Records: 6 **Literature:** Bailey and MacKay (1916), Chester et al. (1979), Clemens (1933), Gran and Angst (1931), Hobson and McQuoid (2001)

Morphology: WL: iNat (253842319) Voucher (WL-SEM-82) **Syn:** *Lauderia borealis* Gran

Phylum: Bacillariophyta
Class: Coscinodiscophyceae
Subclass: Thalassiosirophycidae
Order: Thalassiosirales
Family: Skeletonemataceae

(M) *Skeletonema* cf. *dohrnii* Sarno & Kooistra
Records: 18 **Molecular (amplicon):** IMERSS (ENA:ERS27217975)

(M) *Skeletonema costatum* (Greville) Cleve
Records: 63 **Literature:** Chester et al. (1979), Clemens (1933), Forbes and Waters (1993), Gran and Angst (1931), Haigh et al. (1992), Hollibaugh et al. (1980), Horner et al. (2005), Johnson (1931), Kennedy and LeBrasseur (1977), McQuoid and Hobson (1997), McQuoid and Hobson (2001), Phifer (1932), Powys (1987), Sancetta and Calvert (1988), Sancetta (1990), Shim (1976), Stockner and Cliff (1975), Stockner and Cliff (1976), Stockner and Cliff (1979), Waters et al. (1992) **Morphology:** MBA (250AT-5-102)

(M) *Skeletonema japonicum* Zingone & Sarno
Records: 78 **Molecular (amplicon):** IMERSS (ENA:ERS27214058)

(M) *Skeletonema marinoi* Sarno & Zingone
Records: 177 **Literature:** Godhe et al. (2006) **Morphology:** Hakai (Hakai_phyto_QU39_2016-04-28_1m_35) **Molecular (amplicon):** IMERSS (ENA:ERS21395345)

(M) *Skeletonema mediterraneum* Brun
Records: 1 **Literature:** Tempère and Peragallo (1908)

Phylum: Bacillariophyta
Class: Coscinodiscophyceae
Subclass: Thalassiosirophycidae
Order: Thalassiosirales
Family: Thalassiosiraceae

(M) *Bacterosira* cf. *constricta* (Gaarder) J.S.Park & J.H.Lee ✦
Records: 10 **Morphology:** WL: iNat (259444260) Voucher (WL-SEM-9) **Molecular (amplicon):** IMERSS (ENA:ERS27217975)

(M) *Conticribra guillardii* (Hasle) Stachura-Suchoples & D.M.Williams
Records: 1 **Literature:** Waters et al. (1992) **Syn:** *Thalassiosira guillardii* Hasle

(M) *Conticribra weissflogii* (Grunow) Stachura-Suchoples & D.M.Williams
Records: 1 **Literature:** Waters et al. (1992) **Syn:** *Thalassiosira weissflogii* (Grunow) G.A.Fryxell & Hasle

(M) *Detonula pumila* (Castracane) Gran
Records: 93 **Literature:** Forbes and Waters (1993), Sancetta and Calvert (1988), Sancetta (1990), Shim (1976), Stockner and Cliff (1975), Stockner and Cliff (1976), Waters et al. (1992) **Morphology:** WL: iNat (5197581), Hakai (Hakai_phyto_QU39_2017-10-11_1m_16), iNat (214417035) **Molecular (amplicon):** IMERSS (ENA:ERS27217975) **Syn:** *Lauderia pumila* Castracane

(M) *Minidiscus* cf. *spinulatus*
Records: 13 **Molecular (amplicon):** IMERSS (ENA:ERS27214058)

(M) *Minidiscus chilensis* Rivera
Records: 12 **Literature:** Sancetta and Calvert (1988), Sancetta (1990) **Morphology:** WL: iNat (171845682) Voucher (WL-SEM-28) **Molecular (amplicon):** IMERSS (ENA:ERS27217975)

(M) *Minidiscus proschkinae* (Makarova) J.S.Park & J.H.Lee ✦
Records: 1 **Morphology:** WL: iNat (260112129) Voucher (WL-SEM-97) **Molecular (amplicon):** IMERSS (ENA:ERS27217975)

(M) *Minidiscus trioculatus* (F.J.R.Taylor) Hasle

Records: 10 **Literature:** Sancetta and Calvert (1988), Sancetta (1990), Waters et al. (1992) **Morphology:** WL: iNat (4873137) **Molecular (amplicon):** IMERSS (ENA:ERS21395345)

(M) ***Planktoniella sol*** **(G.C. Wallich) Schütt**

Records: 6 **Literature:** Clemens (1933), Gran and Angst (1931), Shim (1976), Stockner and Cliff (1975), Stockner and Cliff (1976) **Morphology:** WL: iNat (4873113)

(M) ***Shionodiscus bioculatus*** **(Grunow) Alverson, Kang & Theriot**

Records: 12 **Literature:** Chester et al. (1979), Clemens (1933), Gran and Angst (1931) **Molecular (amplicon):** IMERSS (ENA:ERS27217975) **Syn:** *Thalassiosira bioculata* (Grunow) Ostenfeld

(M) ***Shionodiscus endoseriatus*** **(Hasle & Fryxell) Alverson, Kang & Theriot ✦**

Records: 1 **Morphology:** WL: iNat (259613928) Voucher (WL-SEM-72)

(M) ***Shionodiscus frenguelliopsis*** **(Fryxell & Johansen) Alverson, Kang & Theriot ✦**

Records: 1 **Morphology:** WL: iNat (301822368) Voucher (WL-SEM-106)

(M) ***Shionodiscus karianus*** **Georgiev & Gololobova ✦**

Records: 1 **Morphology:** WL: iNat (322791476) Voucher (Wl-SEM-111)

(M) ***Shionodiscus oestrupii*** **(Ostenfeld) A.J.Alverson, S.H.Kang & E.C.Theriot ✦**

Records: 2 **Morphology:** WL: iNat (142951511) Voucher (WL-SEM-18, WL-SEM-71)

(M) ***Shionodiscus poroirregulatus*** **(Hasle & Heimdal) A.J.Alverson, S.-H.E.C.**

Records: 3 **Literature:** Sancetta (1990) **Morphology:** WL: iNat (202460107) Voucher (WL-SEM-60, WL-SEM-119) **Syn:** *Thalassiosira poroirregulata* Hasle & Heimdal (as '*poro-irregulata*')

(M) ***Shionodiscus ritscheri*** **(Hustedt) Alverson, Kang & Theriot**

Records: 9 **Molecular (amplicon):** IMERSS (ENA:ERS27217975)

(M) ***Shionodiscus trifultus*** **(G.Fryxell) A.J.Alverson, S.H.Kang & E.C.Theriot**

Records: 2 **Literature:** Sancetta and Calvert (1988) **Morphology:** WL: iNat (143402069) Voucher (WL-SEM-52) **Syn:** *Thalassiosira trifulta* G.Fryxell

(M) ***Thalassiosira aestivalis*** **Gran**

Records: 16 **Literature:** Chester et al. (1979), Clemens (1933), Forbes and Waters (1993), Gran and Angst (1931), Haigh et al. (1992), Hollibaugh et al. (1980), Horner et al. (2005), McQuoid and Hobson (1997), Phifer (1932), Sancetta and Calvert (1988), Sancetta (1990), Shim (1976), Stockner and Cliff (1975), Stockner and Cliff (1976), Waters et al. (1992) **Morphology:** WL: iNat (264387996)

(M) ***Thalassiosira allenii*** **H.Takano ✦**

Records: 1 **Morphology:** WL: iNat (259835781) Voucher (WL-SEM-63)

(M) ***Thalassiosira angstii*** **(Gran) Makarova**

Records: 1 **Literature:** Forbes and Waters (1993)

(M) ***Thalassiosira angulata*** **(W.Gregory) Hasle**

Records: 2 **Literature:** Forbes and Waters (1993), Waters et al. (1992)

(M) ***Thalassiosira angustelineata*** **(A.W.F.Schmidt) G.Fryxell & Hasle**

Records: 38 **Literature:** Bailey and MacKay (1916), Buchanan (1966), Chester et al. (1979), Clemens (1933), Forbes and Waters (1993), Gran and Angst (1931), Hobson (1983), Hollibaugh et al. (1980), Horner et al. (2005), McQuoid and Hobson (1997), McQuoid and Hobson (2001), Phifer (1932), Roelofs (1983), Sancetta and Calvert (1988), Sancetta (1990), Shim (1976), Waters et al. (1992) **Morphology:** WL: iNat (9291115), Hakai (Hakai_phyto_QU39_2019-03-20_1m_15) **Molecular (amplicon):** IMERSS (ENA:ERS27217975) **Syn:** *Coscinosira polychorda* (Gran) Gran

(M) ***Thalassiosira antarctica*** **Comber**

Records: 9 **Molecular (amplicon):** IMERSS (ENA:ERS27217975)

(M) ***Thalassiosira baltica*** **(Grunow) Ostenfeld**

Records: 31 **Literature:** Forbes and Waters (1993), Waters et al. (1992) **Molecular (amplicon):** IMERSS (ENA:ERS21395352)

(M) ***Thalassiosira binata*** **Fryxell**
Records: 2 **Literature:** Sancetta (1990), Waters et al. (1992)

(M) ***Thalassiosira*** **cf.** ***normanhendeyi*** **D.M.Williams**
Records: 1 **Molecular (amplicon):** IMERSS (ENA:ERS21395352)

(B) ***Thalassiosira*** **cf.** ***visurgis*** **Hustedt ✦**
Records: 1 **Morphology:** WL: iNat (260003450) Voucher (WL-SEM-99)

(M) ***Thalassiosira condensata*** **Cleve**
Records: 7 **Literature:** Chester et al. (1979), Clemens (1933), Gran and Angst (1931), Johnson (1931), Légaré (1957), Phifer (1932), Stockner and Cliff (1975)

(M) ***Thalassiosira conferta*** **Hasle**
Records: 7 **Literature:** Forbes and Waters (1993), Sancetta (1990), Waters et al. (1992) **Morphology:** WL: iNat (143685844) Voucher (WL-SEM-61)

(M) ***Thalassiosira curviseriata*** **Takano ✦**
Records: 1 **Morphology:** WL: iNat (142613095) Voucher (WL-SEM-44) **Molecular (amplicon):** IMERSS (ENA:ERS27630061)

(M) ***Thalassiosira decipiens*** **(Grunow ex Van Heurck) Jørgensen**
Records: 13 **Literature:** Bailey and MacKay (1916), Buchanan (1966), Chester et al. (1979), Clemens (1933), Forbes and Waters (1993), Gran and Angst (1931), Gucluer and Gross (1964), Haigh et al. (1992), Hobson (1983), Légaré (1957), Phifer (1932), Roelofs (1983), Shim (1976)

(M) ***Thalassiosira eccentrica*** **(Ehrenberg) Cleve**
Records: 62 **Literature:** Buchanan (1966), Clemens (1933), Forbes and Waters (1993), Gran and Angst (1931), Hobson and McQuoid (2001), Horner et al. (2005), Légaré (1957), McQuoid and Hobson (1997), McQuoid and Hobson (2001), Rao and Lewin (1976), Sancetta and Calvert (1988), Shim (1976), Tempère and Peragallo (1908), Tynni (1986), Waters et al. (1992) **Morphology:** WL: iNat (142633545) Voucher (WL-SEM-51, WL-SEM-61) **Molecular (amplicon):** IMERSS (ENA:ERS27217975); **Molecular (Sanger):** IMERSS (ENA: ERZ29258993) **Syn:** *Coscinodiscus eccentricus* Ehrenberg

(M) ***Thalassiosira elsayedii*** **Fryxell**
Records: 1 **Literature:** Shim (1976)

(M) ***Thalassiosira gravida*** **Cleve**
Records: 122 **Literature:** Bailey and MacKay (1916), Chester et al. (1979), Clemens (1933), Forbes and Waters (1993), Haigh et al. (1992), Hobson (1983), Hobson and McQuoid (2001), Horner et al. (2005), Johnson (1931), McQuoid and Hobson (1997), McQuoid and Hobson (2001), Phifer (1932), Sancetta and Calvert (1988), Sancetta (1990), Shim (1976), Waters et al. (1992) **Morphology:** WL: iNat (4849091) Voucher (WL-SEM-2, WL-SEM-13), Hakai (Hakai_phyto_QU39_2021-07-27_5m_20) **Molecular (amplicon):** EBI (ENA:GQ330467), IMERSS (ENA:ERS27217975) **Syn:** *Thalassiosira rotula* Meunier

(M) ***Thalassiosira incerta*** **I.V.Makarova**
Records: 1 **Literature:** Waters et al. (1992)

(M) ***Thalassiosira kushirensis*** **H.Takano**
Records: 1 **Literature:** Sancetta (1990)

(M) ***Thalassiosira leptopus*** **(Grunow) Hasle & G.Fryxell**
Records: 6 **Literature:** Bailey and MacKay (1916), Buchanan (1966), Chester et al. (1979), Forbes and Waters (1993), Tempère and Peragallo (1908), Waters et al. (1992) **Syn:** *Coscinodiscus lineatus* Ehrenberg

(M) ***Thalassiosira lineata*** **Jousé**
Records: 2 **Literature:** Chester et al. (1979), Waters et al. (1992)

(M) ***Thalassiosira lundiana*** **Fryxell**
Records: 4 **Literature:** Sancetta (1990) **Morphology:** WL: iNat (143526054) Voucher (WL-SEM-57, WL-SEM-70)

(M) *Thalassiosira mendiolana* Hasle & Heimdal
Records: 3 **Literature:** Forbes and Waters (1993), Waters et al. (1992) **Morphology:** WL: iNat (327005097)

(M) *Thalassiosira minima* Gaarder ✦
Records: 1 **Morphology:** WL: iNat (259931970) Voucher (WL-SEM-99, WL-SEM-100)

(M) *Thalassiosira minuscula* Krasske
Records: 10 **Molecular (amplicon):** IMERSS (ENA:ERS21395352)

(M) *Thalassiosira nordenskioeldii* Cleve
Records: 107 **Literature:** Bailey and MacKay (1916), Buchanan (1966), Chester et al. (1979), Clemens (1933), Forbes and Waters (1993), Gucluer and Gross (1964), Haigh et al. (1992), Hobson (1983), Hobson and McQuoid (2001), Horner et al. (2005), Légaré (1957), McQuoid and Hobson (1997), McQuoid and Hobson (2001), Phifer (1932), Rao and Lewin (1976), Roelofs (1983), Sancetta and Calvert (1988), Sancetta (1990), Shim (1976), Stockner and Cliff (1975), Stockner and Cliff (1976), Sutherland et al. (2023), Waters et al. (1992) **Morphology:** WL: iNat (111064233), Hakai (Hakai_phyto_QU39_2017-10-11_1m_23) **Molecular (amplicon):** IMERSS (ENA:ERS21395352)

(M) *Thalassiosira oceanica* Hasle ✦
Records: 15 **Morphology:** WL: iNat (259589003) Voucher (WL-SEM-65) **Molecular (amplicon):** EBI (ENA:SRS968559)

(M) *Thalassiosira pacifica* Gran & Angst
Records: 74 **Literature:** Buchanan (1966), Chester et al. (1979), Clemens (1933), Forbes and Waters (1993), Gran and Angst (1931), Hobson and McQuoid (2001), Horner et al. (2005), Johnson (1931), Phifer (1932), Sancetta and Calvert (1988), Sancetta (1990), Shim (1976), Stockner and Cliff (1975), Stockner and Cliff (1976), Waters et al. (1992) **Morphology:** WL: iNat (143431132) Voucher (WL-SEM-44, WL-SEM-84), Hakai (Hakai_phyto_QU39_2018-03-20_1m_17) **Molecular (amplicon):** IMERSS (ENA:ERS21395356)

(M) *Thalassiosira plicata* H.J.Schrader
Records: 2 **Literature:** McQuoid and Hobson (1997), Sancetta (1990)

(M) *Thalassiosira pseudonana* Hasle & Heimdal
Records: 1 **Literature:** Waters et al. (1992)

(M) *Thalassiosira punctigera* (Castracane) Hasle
Records: 37 **Literature:** Bailey and MacKay (1916), Chester et al. (1979), Clemens (1933), Gran and Angst (1931), Hobson and McQuoid (2001), Horner et al. (2005), Phifer (1932), Roelofs (1983), Sancetta and Calvert (1988), Sancetta (1990), Shim (1976), Waters et al. (1992) **Morphology:** WL: iNat (50064842) Voucher (WL-SEM-31) **Molecular (amplicon):** IMERSS (ENA:ERS27217975) **Syn:** *Coscinodiscus angstii* Gran, *Coscinodiscus angstii* var. *granulomarginata* H.H.Gran & E.C.Angst, *Ethmodiscus punctiger* Castracane, *Thalassiosira angstii* (Gran) Makarova

(M) *Thalassiosira subtilis* (Ostenfeld) Gran
Records: 3 **Literature:** Bailey and MacKay (1916), Chester et al. (1979), Clemens (1933)

(M) *Thalassiosira symmetrica* G.A.Fryxell & Hasle
Records: 1 **Literature:** Waters et al. (1992)

(M) *Thalassiosira tealata* H.Takano
Records: 9 **Literature:** Bérard-Therriault et al. (1987), Bérard-Therriault et al. (1999), Harris et al. (1995), Hasle and Syvertsen (1997), Hernández-Becerril and Tapia Peña (1995), Hoppenrath et al. (2007), Li et al. (2014), Takano (1980) **Morphology:** WL: iNat (259977250) Voucher (WL-SEM-99, WL-SEM-101)

(M) *Thalassiosira tenera* Proshkina-Lavrenko
Records: 2 **Literature:** Sancetta (1990), Waters et al. (1992)

Fragilariophyceae (araphid pennates)

Phylum: Bacillariophyta
Class: Fragilariophyceae
Subclass: Fragilariophycidae
Order: Cyclophorales
Family: Entopylaceae
(M) *Entopyla australis* var. *gigantea* (Greville) Fricke
Records: 1 **Literature:** Tempère and Peragallo (1908)
(M) *Gephyria media* Walker-Arnott
Records: 1 **Literature:** Lord (1866)

Phylum: Bacillariophyta
Class: Fragilariophyceae
Subclass: Fragilariophycidae
Order: Fragilariales
Family: Fragilariaceae
(M) *Asterionellopsis glacialis* (Castracane) Round
Records: 90 **Literature:** Buchanan (1966), Chester et al. (1979), Clemens (1933), Forbes and Waters (1993), Gran and Angst (1931), Hobson and McQuoid (2001), Hollibaugh et al. (1980), Horner et al. (2005), Johnson (1931), Légaré (1957), Phifer (1932), Sancetta and Calvert (1988), Shim (1976), Stockner and Cliff (1976) **Morphology:** WL: iNat (4848898), Hakai (Hakai_phyto_QU39_2019-08-15_5m_1), MBA (250AT-5-10615) **Molecular (amplicon):** EBI (ENA:SRS964122) **Syn:** *Asterionella glacialis* Castracane
(FM) *Fragilaria capucina* Desmazières
Records: 5 **Literature:** Bailey and MacKay (1916), Clemens (1933), Roelofs (1983), Shim (1976) **Morphology:** WL: iNat (5197628) **Syn:** *Staurosira capucina* Desm.
(FM) *Fragilaria crotonensis* Kitton
Records: 6 **Literature:** Buchanan (1966), Légaré (1957), Shim (1976), Stockner and Cliff (1975), Stockner and Cliff (1976), Tynni (1986)
(M) *Fragilaria islandica* Grunow ex Van Heurck
Records: 3 **Literature:** Bailey and MacKay (1916), Clemens (1933), Shim (1976)
(F) *Fragilaria marina* var. *parva* Tempère & Peragallo
Records: 1 **Literature:** Tempère and Peragallo (1908)
(F) *Fragilaria rumpens* (Kützing) G.W.F.Carlson
Records: 1 **Literature:** Roelofs (1983) **Syn:** *Synedra rumpens* Kützing
(M) *Fragilaria striatula* Lyngbye
Records: 9 **Literature:** Bailey and MacKay (1916), Clemens (1933), Gran and Angst (1931), Johnson (1931), Légaré (1957), Rao and Lewin (1976), Shim (1976), Stockner and Cliff (1975) **Morphology:** WL: iNat (243251313) Voucher (WL-SEM-53, WL-LM-41)
(F) *Fragilaria vaucheriae* (Kützing) J.B.Petersen
Records: 1 **Literature:** Roelofs (1983)
(F) *Fragilariforma bicapitata* (A.Mayer) D.M.Williams & Round
Records: 1 **Literature:** Roelofs (1983) **Syn:** *Fragilaria bicapitata* A.Mayer
(F) *Fragilariforma virescens* var. *subsalina* (Grunow) Bukhtiyarova
Records: 1 **Literature:** Rao and Lewin (1976) **Syn:** *Fragilaria virescens* var. *subsalina* Grunow
(M) *Gedaniella panicellus* Chunlian Li, S.X.Yu & Witkowski
Records: 13 **Molecular (amplicon):** IMERSS (ENA:ERS27214058)

(F) ***Odontidium anceps*** **(Ehrenberg) Ralfs**
Records: 1 **Literature:** Roelofs (1983) **Syn:** *Diatoma anceps* (Ehrenberg) Kirchner
(F) ***Odontidium hyemale*** **(Roth) Kützing**
Records: 2 **Literature:** Roelofs (1983), Tempère and Peragallo (1908) **Syn:** *Diatoma hyemalis* (Roth) Heiberg (as '*hiemale*')
(F) ***Odontidium mesodon*** **(Ehrenberg) Kützing**
Records: 1 **Literature:** Roelofs (1983) **Syn:** *Diatoma hiemale* var. *mesodon* (Ehrbg.) Grun., *Diatoma mesodon* (Ehrenberg) Kützing
(F) ***Punctastriata lancettula*** **(Schumann) P.B.Hamilton & Siver**
Records: 2 **Literature:** Rao and Lewin (1976), Tempère and Peragallo (1908) **Syn:** *Fragilaria lancettula* Schumann, *Fragilaria pinnata* var. *lancettula* (Schumann) Hustedt

Phylum: Bacillariophyta
Class: Fragilariophyceae
Subclass: Fragilariophycidae
Order: Fragilariales
Family: Staurosiraceae
(M) ***Martyana schulzii*** **(C.Brockmann) Snoeijs**
Records: 1 **Literature:** Tynni (1986) **Syn:** *Opephora schulzii* (C.Brockmann) Simonsen (as '*schulzi*')
(M) ***Opephora marina*** **(W.Gregory) P.Petit**
Records: 2 **Literature:** Rao and Lewin (1976), Roelofs (1983)
(M) ***Opephora pacifica*** **(Grunow) P.Petit**
Records: 4 **Literature:** Bailey and MacKay (1916), Rao and Lewin (1976), Roelofs (1983), Tynni (1986)
(M) ***Opephora schwartzii*** **(Grunow) Petit ex Pelletan**
Records: 1 **Literature:** Roelofs (1983)
(F) ***Pseudostaurosira brevistriata*** **(Grunow) D.M.Williams & Round**
Records: 2 **Literature:** Roelofs (1983), Tynni (1986) **Syn:** *Fragilaria brevistriata* Grunow
(M) ***Pseudostaurosira*** **cf.** ***paucistriata*** **(Chunlian Li & Witkowski) E.A.Morales, C.E.Wetzel & Ector**
Records: 13 **Molecular (amplicon):** IMERSS (ENA:ERS21395352)
(F) ***Staurosira construens*** **Ehrenberg**
Records: 1 **Literature:** Roelofs (1983) **Syn:** *Fragilaria construens* (Ehrenberg) Grunow
(F) ***Staurosira leptostauron*** **(Ehrenberg) Kulikovskiy & Genkal**
Records: 1 **Literature:** Roelofs (1983) **Syn:** *Fragilaria leptostauron* (Ehrenberg) Hustedt
(B) ***Staurosira subsalina*** **(Hustedt) Lange-Bertalot**
Records: 1 **Literature:** Roelofs (1983) **Syn:** *Fragilaria construens* var. *subsalina* Hustedt
(F) ***Staurosira venter*** **(Ehrenberg) Cleve & J.D.Möller**
Records: 3 **Literature:** Rao and Lewin (1976), Roelofs (1983), Tynni (1986) **Syn:** *Fragilaria construens* var. *venter* (Ehrenberg) Grunow
(F) ***Staurosirella crux*** **(Ehrenberg) Van de Vijver & Kusber**
Records: 1 **Literature:** Tempère and Peragallo (1908) **Syn:** *Fragilaria harrisonii* (W.Smith) Grunow
(F) ***Staurosirella martyi*** **(Héribaud) Morales & Manoylov**
Records: 1 **Literature:** Roelofs (1983) **Syn:** *Opephora martyi* Héribaud
(F) ***Staurosirella mutabilis*** **(W.Smith) E.Morales & Van de Vijver**
Records: 1 **Literature:** Bailey and MacKay (1916) **Syn:** *Fragilaria mutabilis* (W.Smith) Grunow
(F) ***Staurosirella pinnata*** **(Ehrenberg) D.M.Williams & Round**

Records: 3 **Literature:** Rao and Lewin (1976), Roelofs (1983), Tynni (1986)
Syn: *Fragilaria pinnata* Ehrenberg

Phylum: Bacillariophyta
Class: Fragilariophyceae
Subclass: Fragilariophycidae
Order: Licmophorales
Family: Licmophoraceae

(M) *Licmophora abbreviata* C.Agardh
Records: 5 **Literature:** Chester et al. (1979), Shim (1976), Stockner and Cliff (1975), Stockner and Cliff (1976) **Morphology:** WL: iNat (195109966) Voucher (WL-SEM-81)

(M) *Licmophora californica* Grunow
Records: 1 **Literature:** Bailey and MacKay (1916)

(M) *Licmophora* cf. *communis* (Heiberg) Grunow ✦
Records: 1 **Morphology:** WL: iNat (263351671) Voucher (WL-SEM-96)

(M) *Licmophora ehrenbergii* (Kützing) Grunow
Records: 5 **Literature:** Lord (1866), Rao and Lewin (1976), Roelofs (1983), Shim (1976) **Morphology:** WL: iNat (195022691) Voucher (WL-SEM-88)
Syn: *Podosphenia ehrenbergii* Kützing

(M) *Licmophora flabellata* (Greville) C.Agardh
Records: 6 **Literature:** Bailey and MacKay (1916), Clemens (1933)
Morphology: WL: iNat (50058346)

(M) *Licmophora gracilis* (Ehrenberg) Grunow
Records: 11 **Literature:** Bailey and MacKay (1916), Clemens (1933), Rao and Lewin (1976), Roelofs (1983) **Morphology:** WL: iNat (253801892) Voucher (WL-LM-20), BRU (ABRU00000263), MSC (MSC0183291), MU (000179639), NY (02113681) **Molecular (amplicon):** EBI (ENA:ERS2393264)

(M) *Licmophora gracilis* var. *anglica* (Kützing) H.Peragallo & M.Peragallo
Records: 1 **Literature:** Bailey and MacKay (1916) **Syn:** *Licmophora anglica* (Kützing) Grunow

(M) *Licmophora hyalina* (Kützing) Grunow
Records: 1 **Literature:** Bailey and MacKay (1916)

(M) *Licmophora juergensii* C.Agardh
Records: 2 **Literature:** Bailey and MacKay (1916) **Molecular (amplicon):** IMERSS (ENA:ERS27630061)

(M) *Licmophora lyngbyei* (Kützing) Grunow
Records: 4 **Literature:** Bailey and MacKay (1916), Buchanan (1966), Clemens (1933), Waters et al. (1992)

(M) *Licmophora paradoxa* (Lyngbye) C.Agardh
Records: 36 **Literature:** Bailey and MacKay (1916), Shim (1976)
Morphology: WL: iNat (193252391) **Molecular (amplicon):** IMERSS (ENA:ERS21395352)

(M) *Licmophora tincta* (C.Agardh) Grunow ✦
Records: 2 **Morphology:** WL: iNat (195808793) Voucher (WL-SEM-87, WL-SEM-89)

Phylum: Bacillariophyta

Class: Fragilariophyceae

Subclass: Fragilariophycidae

Order: Licmophorales

Family: Ulnariaceae

(F) *Ctenophora pulchella* (Kützing) D.M.Williams & Round
Records: 3 **Literature:** Bailey and MacKay (1916), Tempère and Peragallo (1908), Tynni (1986) **Syn:** *Synedra pulchella* (Kützing) Kützing

(F) *Hannaea arcus* (Ehrenberg) R.M.Patrick
Records: 3 **Literature:** Buchanan (1966), Roelofs (1983), Stockner and Cliff (1976) **Syn:** *Fragilaria arcus* (Ehrenberg) Cleve

(F) *Hannaea arcus* var. *amphioxys* (Rabenhorst) R.M.Patrick
Records: 1 **Literature:** Roelofs (1983)

(M) *Synedra affinis* var. *fasciculata* (Lyngbye) Grunow
Records: 2 **Morphology:** MSC (MSC0184978), MU (000179641)

(M) *Synedra camtschatica* Grunow
Records: 3 **Literature:** Rao and Lewin (1976) **Morphology:** WL: iNat (189257335)

(M) *Synedra finnmarchica* (Cleve & Grunow) D.M.Williams & Karthick
Records: 1 **Literature:** Rao and Lewin (1976) **Syn:** *Synedra camtschatica* var. *finnmarchica* Cleve & Grunow (as '*kamtschatica*')

(B) *Synedra gaillonii* (Bory) Ehrenberg
Records: 3 **Literature:** Bailey and MacKay (1916), Clemens (1933) **Morphology:** WL: iNat (295852109)

(M) *Synedra kerguelensis* Heiden
Records: 1 **Literature:** Shim (1976)

(F) *Synedra tabulata* var. *delicatula* (Grunow) R.Ross
Records: 2 **Literature:** Bailey and MacKay (1916), Clemens (1933) **Syn:** *Synedra affinis* var. *tabulata* Grunow

(F) *Tabularia affinis* (Kützing) Snoeijs
Records: 4 **Literature:** Bailey and MacKay (1916), Lord (1866) **Morphology:** BRU (ABRU00000417) **Syn:** *Synedra affinis* Kützing

(M) *Tabularia fasciculata* (C.Agardh) D.M.Williams & Round
Records: 13 **Literature:** Rao and Lewin (1976), Roelofs (1983) **Morphology:** MSC (MSC0184988), MU (000179640) **Molecular (amplicon):** IMERSS (ENA:ERS27217975) **Syn:** *Synedra fasciculata* (C.Agardh) Kützing, nom. illeg., *Synedra fasciculata* var. *truncata* (Grev.) R.M.Patrick

(M) *Tabularia investiens* (W.Smith) D.M.Williams & Round
Records: 3 **Literature:** Bailey and MacKay (1916), Clemens (1933) **Morphology:** WL: iNat (252835907) Voucher (WL-SEM-14) **Syn:** *Synedra investiens* W.Smith

(M) *Tabularia tabulata* (C.Agardh) Snoeijs
Records: 13 **Literature:** Rao and Lewin (1976), Roelofs (1983), Tynni (1986) **Molecular (amplicon):** IMERSS (ENA:ERS21395345) **Syn:** *Synedra tabulata* (C.Agardh) Kützing

(F) *Ulnaria acus* (Kützing) Aboal
Records: 3 **Literature:** Bailey and MacKay (1916), Roelofs (1983), Shim (1976) **Syn:** *Synedra acus* Kützing

(F) *Ulnaria aequalis* (Kützing) D.M.Williams & Van de Vijver
Records: 1 **Literature:** Tempère and Peragallo (1908) **Syn:** *Synedra ulna* var. *aequalis* Rab.

(M) *Ulnaria amphirhynchus* (Ehrenberg) Compère & Bukhtiyarova
Records: 1 **Literature:** Shim (1976) **Syn:** *Synedra ulna* var. *amphirhynchus* (Ehrenberg) Grunow

(F) *Ulnaria danica* (Kützing) Compère & Bukhtiyarova

Records: 1 **Literature:** Tempère and Peragallo (1908) **Syn:** *Synedra ulna* var. *danica* (Kützing) Van Heurck

(F) ***Ulnaria goulardii*** **(Brébisson ex Cleve & Grunow) D.M.Williams, Potapova & C.E.Wetzel**

Records: 1 **Literature:** Tempère and Peragallo (1908) **Syn:** *Synedra goulardii* Brébisson ex Cleve & Grunow

(F) ***Ulnaria lanceolata*** **(Kützing) Compère**

Records: 1 **Literature:** Tempère and Peragallo (1908) **Syn:** *Synedra ulna* var. *lanceolata* Grunow

(F) ***Ulnaria ulna*** **(Nitzsch) Compère**

Records: 5 **Literature:** Bailey and MacKay (1916), Clemens (1933), Roelofs (1983), Shim (1976), Tempère and Peragallo (1908) **Syn:** *Synedra ulna* (Nitzsch) Ehrenberg

Phylum: Bacillariophyta

Class: Fragilariophyceae

Subclass: Fragilariophycidae

Order: Rhabdonematales

Family: Grammatophoraceae

(M) ***Grammatophora angulosa*** **Ehrenberg**

Records: 8 **Literature:** Bailey and MacKay (1916), Rao and Lewin (1976), Roelofs (1983), Shim (1976), Tynni (1986) **Morphology:** WL: iNat (257265591) Voucher (WL-SEM-112, WL-LM-49)

(M) ***Grammatophora*** **cf.** ***arctica*** **Cleve**

Records: 1 **Literature:** Roelofs (1983)

(M) ***Grammatophora hamulifera*** **Kützing**

Records: 3 **Literature:** Bailey and MacKay (1916), Clemens (1933) **Morphology:** WL: iNat (296020829) **Syn:** *Grammatophora angulosa* var. *hamulifera* (Ktz.) Grunow

(M) ***Grammatophora macilenta*** **W.Smith**

Records: 3 **Literature:** Bailey and MacKay (1916), Roelofs (1983) **Morphology:** WL: iNat (296108781) **Syn:** *Grammatophora oceanica* var. *macilenta* (W.Smith) Grunow

(M) ***Grammatophora marina*** **(Lyngbye) Kützing**

Records: 12 **Literature:** Bailey and MacKay (1916), Buchanan (1966), Chester et al. (1979), Clemens (1933), Légaré (1957), Lord (1866), McQuoid and Hobson (1997), McQuoid and Hobson (2001), Roelofs (1983), Shim (1976) **Morphology:** WL: iNat (202250545) Voucher (WL-LM-6)

(M) ***Grammatophora maxima*** **Grunow**

Records: 4 **Literature:** Bailey and MacKay (1916), Clemens (1933), Roelofs (1983), Shim (1976)

(M) ***Grammatophora oceanica*** **Ehrenberg**

Records: 6 **Literature:** Bailey and MacKay (1916), Clemens (1933), Roelofs (1983) **Morphology:** WL: iNat (190090670)

(M) ***Grammatophora oceanica*** **f.** ***vulgaris*** **(Grunow) Hustedt**

Records: 1 **Literature:** Bailey and MacKay (1916)

(M) ***Grammatophora oceanica*** **var.** ***communis*** **Grunow**

Records: 1 **Literature:** Bailey and MacKay (1916)

(M) ***Grammatophora oceanica*** **var.** ***subtilissima*** **(Bailey) Grunow.**

Records: 1 **Morphology:** WL: iNat (263332652)

(M) ***Grammatophora serpentina*** **Ehrenberg**

Records: 3 **Literature:** Bailey and MacKay (1916), Clemens (1933), Lord (1866)

Phylum: Bacillariophyta
Class: Fragilariophyceae
Subclass: Fragilariophycidae
Order: Rhabdonematales
Family: Rhabdonemataceae

(M) *Hyalosira delicatula* Kützing
Records: 1 **Literature:** Shim (1976) **Syn:** *Striatella delicatula* (Kützing) Grunow ex Van Heurck

(M) *Rhabdonema adriaticum* Kützing
Records: 2 **Literature:** Clemens (1933), Shim (1976)

(M) *Rhabdonema arcuatum* (Lyngbye) Kützing
Records: 37 **Literature:** Bailey and MacKay (1916), Buchanan (1966), Clemens (1933), Lord (1866), Shim (1976), Tempère and Peragallo (1908) **Morphology:** WL: iNat (4856581) **Molecular (amplicon):** IMERSS (ENA:ERS21395359)

(F) *Rhabdonema arcuatum* var. *robustum* (Grunow) Hustedt
Records: 2 **Literature:** Rao and Lewin (1976) **Morphology:** M (187510 / 463949 / E 7127)

(M) *Rhabdonema arcuatum* var. *subrostratum* (Fricke) Hustedt
Records: 1 **Morphology:** M (186989 / 461905 / E 5278) **Syn:** *Rhabdonema subrostratum* Fricke

(M) *Rhabdonema biquadratum* Tempère & Brun
Records: 1 **Literature:** Tempère and Peragallo (1908)

(FM) *Rhabdonema minutum* Kützing
Records: 2 **Literature:** Bailey and MacKay (1916), Clemens (1933)

Phylum: Bacillariophyta
Class: Fragilariophyceae
Subclass: Fragilariophycidae
Order: Rhabdonematales
Family: Tabellariaceae

(FM) *Asterionella bleakeleyi* W.Smith
Records: 3 **Literature:** Forbes and Waters (1993), Shim (1976) **Morphology:** WL: iNat (112011487)

(F) *Asterionella formosa* Hassall
Records: 6 **Literature:** Bailey and MacKay (1916), Forbes and Waters (1993), Shim (1976), Stockner and Cliff (1976), Waters et al. (1992) **Morphology:** WL: iNat (167669456)

(FM) *Asterionella notata* Grunow ex Van Heurck
Records: 1 **Literature:** Bailey and MacKay (1916)

(F) *Diatoma elongata* (Lyngbye) C.Agardh
Records: 1 **Literature:** Roelofs (1983)

(FMB) *Diatoma vulgaris* Bory
Records: 2 **Literature:** Bailey and MacKay (1916), Clemens (1933)

(F) *Meridion circulare* (Greville) C.Agardh
Records: 2 **Literature:** Buchanan (1966), Shim (1976)

(F) *Meridion constrictum* Ralfs
Records: 1 **Literature:** Tempère and Peragallo (1908)

(F) *Tabellaria* cf. *quadriseptata* B.M.Knudson ✦
Records: 1 **Morphology:** WL: iNat (259219380) Voucher (WL-SEM-74)

(F) *Tabellaria fenestrata* (Lyngbye) Kützing
Records: 5 **Literature:** Buchanan (1966), Roelofs (1983), Shim (1976), Tempère and Peragallo (1908), Waters et al. (1992)

(F) *Tabellaria flocculosa* (Roth) Kützing

Records: 6 **Literature:** Bailey and MacKay (1916), Buchanan (1966), Clemens (1933), Rao and Lewin (1976), Roelofs (1983), Shim (1976) **Syn:** *Tabellaria fenestrata* var. *intermedia* Grunow

(F) *Tabellaria flocculosa* var. *asterionelloides* (Grunow) Knudson

Records: 1 **Literature:** Shim (1976) **Syn:** *Tabellaria fenestrata* var. *asterionelloides* Grunow

Phylum: Bacillariophyta

Class: Fragilariophyceae

Subclass: Fragilariophycidae

Order: Thalassionematales

Family: Thalassionemataceae

(M) *Thalassionema bacillare* (Heiden) Kolbe

Records: 1 **Literature:** Forbes and Waters (1993) **Notes:** orth. corr. '*Thalassionema bacillaris*'

(M) *Thalassionema frauenfeldii* (Grunow) Tempère & Peragallo

Records: 7 **Literature:** Bailey and MacKay (1916), Buchanan (1966), Chester et al. (1979), Gran and Angst (1931), Légaré (1957), Roelofs (1983), Shim (1976) **Syn:** *Thalassiothrix frauenfeldii* (Grunow) Grunow

(M) *Thalassionema nitzschioides* (Grunow) Mereschkowsky

Records: 225 **Literature:** Bailey and MacKay (1916), Buchanan (1966), Chester et al. (1979), Clemens (1933), Forbes and Waters (1993), Hobson (1983), Hobson and McQuoid (2001), Horner et al. (2005), Johnson (1931), Légaré (1957), McQuoid and Hobson (1997), McQuoid and Hobson (2001), Phifer (1932), Roelofs (1983), Sancetta and Calvert (1988), Shim (1976), Stockner and Cliff (1975), Stockner and Cliff (1976), Waters et al. (1992) **Morphology:** WL: iNat (50162746), Hakai (Hakai_phyto_QU39_2021-07-27_5m_19), MBA (154AT-5-117)

(M) *Thalassiothrix longissima* Cleve & Grunow

Records: 13 **Literature:** Chester et al. (1979), Clemens (1933), Gucluer and Gross (1964), Roelofs (1983) **Morphology:** MBA (268AT-5-116)

Phylum: Bacillariophyta

Class: Fragilariophyceae

Subclass: Urneidophycidae

Order: Plagiogrammales

Family: Plagiogrammaceae

(M) *Dimeregramma marinum* (W.Gregory) Ralfs

Records: 2 **Literature:** Bailey and MacKay (1916), Clemens (1933)

(M) *Glyphodesmis distans* (W.Gregory) Grunow

Records: 1 **Literature:** Roelofs (1983)

(M) *Glyphodesmis williamsonii* (W.Smith) Grunow

Records: 2 **Literature:** Bailey and MacKay (1916), Clemens (1933)

(M) *Plagiogramma acutum* (Hustedt) Chunlian Li, Ashworth & J.Witkowski

Records: 2 **Molecular (amplicon):** EBI (ENA:KF768027)

(M) *Plagiogramma acutumontgo* (B.S.Gray Jr. & Kaczmarska) Chunlian Li, Ashworth & J.Witkowski

Records: 1 **Molecular (amplicon):** EBI (ENA:KX586261) **Syn:** *Dimeregramma acutumontgo* B.S.Gray Jr. & Kaczmarska

(M) *Plagiogramma gregorianum* Greville

Records: 2 **Literature:** Bailey and MacKay (1916), Clemens (1933)

(M) *Plagiogramma interruptum* (W.Gregory) Ralfs

Records: 1 **Literature:** Bailey and MacKay (1916)

(M) *Plagiogramma minus* (W.Gregory) Chunlian Li, Ashworth & J.Witkowski

Records: 5 **Literature:** Bailey and MacKay (1916), Clemens (1933), Rao and Lewin (1976), Roelofs (1983) **Morphology:** WL: iNat (263208454) **Syn:** *Dimeregramma minus* (W.Gregory) Ralfs (as '*minor*')

(M) ***Plagiogramma pulchellum*** **Greville**

Records: 2 **Literature:** Bailey and MacKay (1916), Tempère and Peragallo (1908)

(M) ***Plagiogramma staurophorum*** **(W.Gregory) Heiberg**

Records: 5 **Literature:** Rao and Lewin (1976), Roelofs (1983), Tynni (1986) **Morphology:** WL: iNat (190225682) Voucher (WL-SEM-69, WL-LM-29), M (187318 / 463062 / E 6325)

(M) ***Plagiogramma tsawwassen*** **Kaczmarska & B.S.Gray**

Records: 7 **Molecular (amplicon):** EBI (ENA:KX586249), IMERSS (ENA:ERS21395356)

(M) ***Psammogramma vigoensis*** **Shin.Sato & Medlin**

Records: 13 **Molecular (amplicon):** IMERSS (ENA:ERS27214058)

Phylum: Bacillariophyta
Class: Fragilariophyceae
Subclass: Urneidophycidae
Order: Rhaphoneidales
Family: Asterionellopsidaceae

(M) ***Asteroplanus karianus*** **(Grunow) C.Gardner & R.M.Crawford**

Records: 7 **Literature:** Clemens (1933), Forbes and Waters (1993), Gran and Angst (1931), Phifer (1932), Shim (1976), Stockner and Cliff (1975), Stockner and Cliff (1976) **Syn:** *Asterionella kariana* Grunow

Phylum: Bacillariophyta
Class: Fragilariophyceae
Subclass: Urneidophycidae
Order: Rhaphoneidales
Family: Psammodiscaceae

(M) ***Psammodiscus nitidus*** **(W.Gregory) Round & D.G.Mann**

Records: 3 **Literature:** Chester et al. (1979), Rao and Lewin (1976), Roelofs (1983) **Syn:** *Coscinodiscus nitidus* W.Gregory

Phylum: Bacillariophyta
Class: Fragilariophyceae
Subclass: Urneidophycidae
Order: Rhaphoneidales
Family: Rhaphoneidaceae

(M) ***Delphineis angustata*** **(Pantocsek) G.W.Andrews**

Records: 1 **Literature:** Roelofs (1983) **Syn:** *Rhaphoneis angustata* Pantocsek

(M) ***Delphineis minutissima*** **(Hustedt) Simonsen**

Records: 1 **Literature:** Tynni (1986) **Syn:** *Rhaphoneis minutissima* Hustedt

(M) ***Delphineis surirella*** **(Ehrenberg) G.W.Andrews**

Records: 1 **Literature:** Shim (1976) **Syn:** *Rhaphoneis surirella* (Ehrenberg) Grunow

(M) ***Rhaphoneis amphiceros*** **(Ehrenberg) Ehrenberg**

Records: 2 **Literature:** Rao and Lewin (1976), Shim (1976)

(M) ***Rhaphoneis caduceus*** **(Ehrenberg) Heurck**

Records: 1 **Literature:** Roelofs (1983)

Phylum: Bacillariophyta
Class: Fragilariophyceae
Subclass: Urneidophycidae
Order: Striatellales
Family: Striatellaceae
(M) *Striatella unipunctata* (Lyngbye) C.Agardh
Records: 4 **Literature:** Buchanan (1966), Clemens (1933), Waters et al. (1992) **Morphology:** Hakai (Hakai_phyto_QU39_2017-08-15_1m_46) **Syn:** *Fragilaria unipunctata* Lyngbye

Bacillariophyceae (raphid pennates)

Phylum: Bacillariophyta
Class: Bacillariophyceae
Subclass: incertae sedis
Order: incertae sedis
Family: incertae sedis
(F) *Actinoneis lorenziana* (Grunow) Mereschkowsky
Records: 1 **Literature:** Bailey and MacKay (1916) **Syn:** *Achnanthes lorenziana* (Grunow) Cleve
(F) *Ambo balticus* (Simonsen) Witkowski, Lange-Bertalot & Ashworth
Records: 1 **Literature:** Rao and Lewin (1976) **Syn:** *Anaulus balticus* Simonsen
(M) *Drepanotheca bivittata* (Grunow & Pantoscek) Schrader
Records: 2 **Literature:** Bailey and MacKay (1916), Clemens (1933) **Syn:** *Eunotogramma bivittata* Grunow & Pantocsek (as '*bivittatum*')
(M) *Libellus grevillei* (C.Agardh) Cleve
Records: 2 **Literature:** Bailey and MacKay (1916) **Morphology:** FLAS (FLAS A766) **Syn:** *Navicula grevillei* (Agardh) Heiberg

Phylum: Bacillariophyta
Class: Bacillariophyceae
Subclass: Bacillariophycidae
Order: Bacillariales
Family: Bacillariaceae
(B) *Bacillaria paxillifera* (O.F.Müller) T.Marsson
Records: 18 **Literature:** Bailey and MacKay (1916), Buchanan (1966), Clemens (1933), Gran and Angst (1931), Horner et al. (2005), McQuoid and Hobson (1997), McQuoid and Hobson (2001), Phifer (1932), Roelofs (1983), Sancetta and Calvert (1988), Shim (1976) **Morphology:** WL: iNat (192871056), MBA (37AT-41-153) **Molecular (amplicon):** EBI (ENA:ERS2393309), IMERSS (ENA:ERS27630061) **Syn:** *Bacillaria paxillifer* (O.F.Müll.) Hendey, *Nitzschia paradoxa* Gmelin, *Nitzschia paradoxa* Grunow, *Vibrio paxillifer* O.F.Müller
(M) *Bacillaria socialis* (W.Gregory) Ralfs
Records: 5 **Literature:** Bailey and MacKay (1916), Roelofs (1983), Shim (1976), Tempère and Peragallo (1908) **Morphology:** WL: iNat (193066116) Voucher (WL-LM-36) **Syn:** *Nitzschia socialis* W.Gregory
(M) *Bacillaria socialis* var. *baltica* (Grunow) De Toni
Records: 2 **Literature:** Bailey and MacKay (1916) **Morphology:** WL: iNat (332753303) Voucher (WL-SEM-11-2) **Syn:** *Nitzschia socialis* var. *baltica* Grunow
(M) *Cylindrotheca closterium* (Ehrenberg) Reimann & J.C.Lewin

Records: 264 **Literature:** Buchanan (1966), Chester et al. (1979), Clemens (1933), Forbes and Waters (1993), Gran and Angst (1931), Hobson (1983), Hobson and McQuoid (2001), Horner et al. (2005), Phifer (1932), Shim (1976), Stockner and Cliff (1975), Stockner and Cliff (1976), Waters et al. (1992) **Morphology:** WL: iNat (4876167), Hakai (Hakai_phyto_QU39_2021-07-27_5m_12), MBA (195VJ-5-177) **Molecular (amplicon):** EBI (ENA:SRS2340158), IMERSS (ENA:ERS21395346) **Syn:** *Ceratoneis closterium* Ehrenberg (as '*Closterium*'), *Nitzschia closterium* (Ehrenberg) W.Smith

(M) ***Cylindrotheca fusiformis*** **Reimann & J.C.Lewin**
Records: 2 **Literature:** Forbes and Waters (1993), Waters et al. (1992)

(M) ***Fragilariopsis cylindriformis*** **(Hasle) Hasle**
Records: 1 **Literature:** Waters et al. (1992) **Syn:** *Nitzschia cylindriformis* Hasle

(M) ***Fragilariopsis cylindrus*** **(Grunow ex Cleve) Helmcke & Krieger**
Records: 2 **Literature:** Rao and Lewin (1976), Roelofs (1983) **Syn:** *Fragilaria cylindrus* Grunow ex Cleve

(M) ***Fragilariopsis doliolus*** **(Wallich) Medlin & P.A.Sims**
Records: 1 **Literature:** McQuoid and Hobson (2001) **Syn:** *Synedra doliolus* Wallich

(M) ***Fragilariopsis oceanica*** **(Cleve) Hasle**
Records: 2 **Morphology:** WL: iNat (5197620), UBC (A084355) **Syn:** *Fragilaria oceanica* Cleve

(F) ***Hantzschia amphioxys*** **var.** ***major*** **Grunow**
Records: 1 **Literature:** Tempère and Peragallo (1908)

(FM) ***Hantzschia elongata*** **(Hantzsch) Grunow**
Records: 1 **Literature:** Tempère and Peragallo (1908) **Syn:** *Hantzschia amphioxys* var. *elongata* Grunow

(M) ***Hantzschia virgata*** **(Roper) Grunow**
Records: 2 **Literature:** Tempère and Peragallo (1908) **Morphology:** M (186421 / 460878 / E 4344)

(M) ***Hantzschia virgata*** **var.** ***gracilis*** **Hustedt**
Records: 1 **Literature:** Shim (1976)

(M) ***Homoeocladia angularis*** **(W.Smith) Kuntze**
Records: 5 **Literature:** Bailey and MacKay (1916), Clemens (1933), Lord (1866), Roelofs (1983), Shim (1976) **Syn:** *Nitzschia angularis* W.Smith

(M) ***Homoeocladia*** **cf.** ***nanodissipata*** **(Chunlian Li & Witkowski) Lobban & Ashworth**
Records: 13 **Molecular (amplicon):** IMERSS (ENA:ERS27214058)

(M) ***Homoeocladia*** **cf.** ***volvendirostrata*** **(Ashworth, Dąbek & Witkowski) Lobban & Ashworth**
Records: 2 **Molecular (amplicon):** IMERSS (ENA:ERS21395352)

(M) ***Homoeocladia distans*** **(W.Gregory) Kuntze**
Records: 1 **Literature:** Rao and Lewin (1976) **Syn:** *Nitzschia distans* W.Gregory

(M) ***Homoeocladia spathulatoides*** **Lobban, C.S. & Ashworth, M.P. ✦**
Records: 1 **Morphology:** WL: iNat (193640716) Voucher (WL-SEM-66) **Syn:** *Nitzschia spathulata* Brébisson ex W.Smith

(M) ***Neodenticula seminae*** **(Simonsen & T.Kanaya) Akiba & Yanagisawa**
Records: 6 **Literature:** Shim (1976) **Morphology:** MBA (195VJ-5-1568) **Syn:** *Denticula seminae* Simonsen & T.Kanaya

(M) ***Nitzschia bicapitata*** **Cleve**
Records: 2 **Literature:** Forbes and Waters (1993), Waters et al. (1992)

(M) ***Nitzschia bilobata*** **W.Smith**

Records: 7 **Literature:** Buchanan (1966), Roelofs (1983), Stockner and Cliff (1975), Stockner and Cliff (1976), Tempère and Peragallo (1908), Tynni (1986), Waters et al. (1992)

(M) ***Nitzschia bilobata*** **var.** ***minor*** **Grunow**
Records: 1 **Literature:** Tempère and Peragallo (1908)

(M) ***Nitzschia brevirostris*** **Hustedt**
Records: 1 **Literature:** Roelofs (1983)

(M) ***Nitzschia*** **cf.** ***adhaerens*** **Mucko & Bosak**
Records: 9 **Molecular (amplicon):** IMERSS (ENA:ERS27217975)

(M) ***Nitzschia*** **cf.** ***aequorea*** **Hustedt**
Records: 26 **Molecular (amplicon):** IMERSS (ENA:ERS21395352)

(F) ***Nitzschia*** **cf.** ***dissipata*** **(Kützing) Rabenhorst**
Records: 45 **Molecular (amplicon):** IMERSS (ENA:ERS21395352)

(F) ***Nitzschia*** **cf.** ***inconspicua*** **Grunow**
Records: 1 **Molecular (amplicon):** IMERSS (ENA:ERS21395365)

(M) ***Nitzschia*** **cf.** ***ligowskii*** **Witkowski, Lange-Bertalot, Kociolek & Brzezinska**
Records: 18 **Molecular (amplicon):** IMERSS (ENA:ERS21395346)

(M) ***Nitzschia*** **cf.** ***majuscula*** **Grunow**
Records: 1 **Literature:** Roelofs (1983)

(F) ***Nitzschia*** **cf.** ***supralitorea*** **Lange-Bertalot**
Records: 5 **Molecular (amplicon):** IMERSS (ENA:ERS21395345)

(M) ***Nitzschia droebakensis*** **Hasle**
Records: 1 **Literature:** Waters et al. (1992)

(M) ***Nitzschia dubiiformis*** **Hustedt**
Records: 9 **Molecular (amplicon):** IMERSS (ENA:ERS27217975)

(B) ***Nitzschia filiformis*** **(W.Smith) Van Heurck**
Records: 1 **Literature:** Clemens (1933) **Syn:** *Homoeocladia filiformis* W.Smith

(FM) ***Nitzschia frustulum*** **(Kützing) Grunow**
Records: 1 **Literature:** Lobban (1985)

(M) ***Nitzschia habirshawii*** **Febiger ex Cleve & Möller**
Records: 1 **Literature:** Tempère and Peragallo (1908) **Syn:** *Nitzschia sigma* var. *habirshawii* (Febiger ex Cleve & Möller) Grunow

(M) ***Nitzschia hybrida*** **Grunow**
Records: 1 **Literature:** Bailey and MacKay (1916)

(F) ***Nitzschia impressa*** **Hustedt**
Records: 1 **Literature:** Waters et al. (1992)

(M) ***Nitzschia incurva*** **Grunow**
Records: 1 **Literature:** Tynni (1986)

(M) ***Nitzschia insignis*** **W.Gregory**
Records: 3 **Literature:** Bailey and MacKay (1916), Clemens (1933), Shim (1976)

(M) ***Nitzschia interruptestriata*** **Simonsen**
Records: 1 **Literature:** Shim (1976)

(FM) ***Nitzschia linearis*** **W.Smith**
Records: 1 **Literature:** Tempère and Peragallo (1908)

(M) ***Nitzschia longa*** **Grunow**
Records: 1 **Literature:** Bailey and MacKay (1916)

(M) ***Nitzschia longissima*** **(Brébisson ex Kützing) Grunow**
Records: 13 **Literature:** Bailey and MacKay (1916), Buchanan (1966), Chester et al. (1979), Clemens (1933), Forbes and Waters (1993), Gran and Angst (1931), Lobban (1985), Phifer (1932), Roelofs (1983), Shim (1976), Stockner and Cliff (1976), Waters et al. (1992) **Morphology:** WL: iNat (193648814) Voucher (WL-LM-31) **Syn:** *Nitzschiella longissima* (Brébisson ex Kützing) Rabenhorst

(M) ***Nitzschia medioconstricta*** **Hustedt**
Records: 9 **Molecular (amplicon):** IMERSS (ENA:ERS21395352)

(F) ***Nitzschia microcephala*** **Grunow**
Records: 1 **Literature:** Roelofs (1983)
(M) ***Nitzschia mitchelliana*** **Greenleaf**
Records: 1 **Literature:** Buchanan (1966)
(M) ***Nitzschia norvegica*** **Hasle**
Records: 1 **Literature:** Waters et al. (1992)
(F) ***Nitzschia obtusa*** **W.Smith**
Records: 1 **Literature:** Buchanan (1966)
(M) ***Nitzschia pacifica*** **Cupp**
Records: 2 **Literature:** Buchanan (1966), Roelofs (1983)
(F) ***Nitzschia palea*** **(Kützing) W.Smith**
Records: 1 **Literature:** Rao and Lewin (1976) **Molecular (amplicon):** IMERSS (ENA:ERS27217975)
(F) ***Nitzschia paleaeformis*** **Hustedt**
Records: 1 **Molecular (amplicon):** IMERSS (ENA:ERS21395359)
(F) ***Nitzschia panduriformis*** **var.** ***minor*** **Grunow**
Records: 1 **Literature:** Bailey and MacKay (1916) **Notes:** material referable to *Nitzschia panduriformis* var. *minor* Grunow, which has no valid combination in *Psammodictyon panduriforme*
(M) ***Nitzschia perindistincta*** **Cholnoky**
Records: 27 **Literature:** Lobban (1985) **Molecular (amplicon):** IMERSS (ENA:ERS27214058) **Syn:** *Nitzschia fontifuga* Cholnoky
(F) ***Nitzschia pusilla*** **Grunow**
Records: 1 **Literature:** Rao and Lewin (1976) **Molecular (amplicon):** IMERSS (ENA:ERS27630061) **Syn:** *Nitzschia kuetzingiana* Hilse (as '*kützingiana*')
(B) ***Nitzschia reversa*** **W.Smith**
Records: 2 **Literature:** Bailey and MacKay (1916) **Morphology:** WL: iNat (50303688) **Syn:** *Nitzschia longissima* var. *reversa* Grunow
(F) ***Nitzschia scalpelliformis*** **Grunow**
Records: 1 **Literature:** Tynni (1986) **Syn:** *Nitzschia obtusa* var. *scalpelliformis* (Grunow) Grunow
(M) ***Nitzschia sicula*** **(Castracane) Hustedt**
Records: 1 **Literature:** Bailey and MacKay (1916) **Syn:** *Pseudo-nitzschia migrans* Cleve.
(B) ***Nitzschia sigma*** **(Kützing) W.Smith**
Records: 5 **Literature:** Bailey and MacKay (1916), Lord (1866), Roelofs (1983), Tempère and Peragallo (1908), Tynni (1986)
(M) ***Nitzschia sigma*** **var.** ***rigida*** **Grunow ex Van Heurck**
Records: 2 **Literature:** Bailey and MacKay (1916), Tempère and Peragallo (1908) **Syn:** *Nitzschia rigida* (Kützing) H.Peragallo & M.Peragallo
(M) ***Nitzschia socialis*** **var.** ***massiliensis*** **Grunow**
Records: 1 **Literature:** Bailey and MacKay (1916)
(M) ***Nitzschia traheaformis*** **Chunlian Li, Witkowski & Yu**
Records: 2 **Molecular (amplicon):** IMERSS (ENA:ERS27630061)
(M) ***Nitzschia tubicola*** **Grunow**
Records: 1 **Literature:** Lobban (1985)
(FM) ***Nitzschia vermicularis*** **(Kützing) Hantzsch**
Records: 4 **Literature:** Bailey and MacKay (1916), Buchanan (1966), Roelofs (1983), Shim (1976)
(M) ***Psammodictyon constrictum*** **(W.Gregory) D.G.Mann**
Records: 2 **Literature:** Bailey and MacKay (1916), Clemens (1933) **Syn:** *Nitzschia constricta* (W.Gregory) Grunow, nom. illeg.
(M) ***Psammodictyon panduriforme*** **(W.Gregory) D.G.Mann**
Records: 2 **Literature:** Clemens (1933), Shim (1976) **Syn:** *Nitzschia panduriformis* W.Gregory

(M) ***Psammodictyon panduriforme* var. *continuum* (Grunow) Snoeijis**
Records: 1 **Literature:** Bailey and MacKay (1916) **Syn:** *Nitzschia panduriformis* var. *continua* Grunow
(M) ***Psammodictyon panduriforme* var. *delicatulum* (Grunow) M.Poulin**
Records: 1 **Literature:** Bailey and MacKay (1916) **Syn:** *Nitzschia panduriformis* var. *delicatula* Grunow
(M) ***Pseudo-nitzschia americana* (Hasle) G.A.Fryxell**
Records: 3 **Literature:** Forbes and Waters (1993), Waters et al. (1992) **Molecular (amplicon):** EBI (ENA:HM007522) **Syn:** *Nitzschia americana* Hasle
(M) ***Pseudo-nitzschia delicatissima* (Cleve) Heiden**
Records: 16 **Literature:** Buchanan (1966), Clemens (1933), Forbes and Waters (1993), Gran and Angst (1931), Hobson (1983), Hobson and McQuoid (2001), Hollibaugh et al. (1980), Phifer (1932), Roelofs (1983), Shim (1976), Waters et al. (1992) **Morphology:** MBA (160AT-5-10623) **Molecular (amplicon):** EBI (ENA:HM007523) **Syn:** *Nitzschia actydrophila* Hasle, *Nitzschia delicatissima* Cleve
(M) ***Pseudo-nitzschia granii* var. *curvata* (Grunow) H.Peragallo & M.Peragallo**
Records: 1 **Literature:** Forbes and Waters (1993)
(M) ***Pseudo-nitzschia linea* Lundholm, Hasle & G.A.Fryxell**
Records: 9 **Molecular (amplicon):** IMERSS (ENA:ERS27217975)
(M) ***Pseudo-nitzschia lineola* (Cleve) Hasle**
Records: 2 **Literature:** Forbes and Waters (1993), Waters et al. (1992) **Syn:** *Nitzschia lineola* Cleve
(M) ***Pseudo-nitzschia multiseries* (Hasle) Hasle**
Records: 19 **Literature:** Hobson and McQuoid (2001) **Molecular (amplicon):** EBI (ENA:HM007506)
(M) ***Pseudo-nitzschia obtusa* (Hasle) Hasle & Lundholm**
Records: 1 **Literature:** Waters et al. (1992) **Syn:** *Nitzschia seriata* f. *obtusa* Hasle
(M) ***Pseudo-nitzschia pseudodelicatissima* (Hasle) Hasle**
Records: 3 **Literature:** Forbes and Waters (1993), Horner et al. (2005), Shim (1976) **Syn:** *Nitzschia delicatula* Hasle, nom. illeg.
(M) ***Pseudo-nitzschia pungens* (Grunow ex Cleve) G.R.Hasle**
Records: 107 **Literature:** Buchanan (1966), Chester et al. (1979), Forbes and Waters (1993), Hollibaugh et al. (1980), Horner et al. (2005), Roelofs (1983), Sancetta and Calvert (1988), Shim (1976), Waters et al. (1992) **Molecular (amplicon):** EBI (ENA:HM007433) **Syn:** *Nitzschia pungens* Grunow ex Cleve
(M) ***Pseudo-nitzschia seriata* (Cleve) H.Peragallo**
Records: 27 **Literature:** Bailey and MacKay (1916), Buchanan (1966), Chester et al. (1979), Clemens (1933), Forbes and Waters (1993), Gran and Angst (1931), Johnson (1931), Phifer (1932), Shim (1976), Stockner and Cliff (1975), Stockner and Cliff (1976), Waters et al. (1992) **Morphology:** MBA (268AT-5-10624) **Molecular (amplicon):** EBI (ENA:HM007521) **Syn:** *Nitzschia seriata* Cleve
(M) ***Pseudo-nitzschia subcurvata* (Hasle) G.A.Fryxell**
Records: 1 **Literature:** Waters et al. (1992) **Syn:** *Nitzschia subcurvata* G.R.Hasle
(M) ***Pseudo-nitzschia subfraudulenta* (Hasle) Hasle**
Records: 1 **Literature:** Forbes and Waters (1993) **Notes:** reports may refer to *N. fraudulenta* Cleve **Syn:** *Nitzschia subfraudulenta* Hasle; Forbes and Waters (1992)
(M) ***Pseudo-nitzschia subpacifica* (Hasle) Hasle**
Records: 1 **Literature:** Forbes and Waters (1993) **Syn:** *Nitzschia subpacifica* Hasle
(M) ***Tryblionella acuminata* W.Smith**

Records: 4 **Literature:** Buchanan (1966), Roelofs (1983), Tempère and Peragallo (1908), Tynni (1986) **Syn:** *Nitzschia acuminata* (W.Smith) Grunow

(F) *Tryblionella angustata* W.Smith
Records: 1 **Literature:** Bailey and MacKay (1916) **Syn:** *Nitzschia angustata* (W.Smith) Grunow

(M) *Tryblionella coarctata* (Grunow) D.G.Mann
Records: 3 **Literature:** Bailey and MacKay (1916), Shim (1976), Tynni (1986) **Syn:** *Nitzschia punctata* var. *coarctata* (Grunow) Hustedt

(M) *Tryblionella gaoana* Witkowski & Chunlian Li
Records: 4 **Molecular (amplicon):** IMERSS (ENA:ERS21395345)

(M) *Tryblionella granulata* (Grunow) D.G.Mann
Records: 1 **Literature:** Roelofs (1983) **Syn:** *Nitzschia granulata* Grunow

(FM) *Tryblionella hantzschiana* Grunow
Records: 3 **Literature:** Bailey and MacKay (1916), Roelofs (1983), Tynni (1986) **Syn:** *Nitzschia tryblionella* Hantzsch

(M) *Tryblionella hungarica* (Grunow) Frenguelli
Records: 1 **Literature:** Tynni (1986) **Syn:** *Nitzschia hungarica* Grunow

(M) *Tryblionella marginulata* (Grunow) D.G.Mann
Records: 1 **Literature:** Roelofs (1983) **Syn:** *Nitzschia marginulata* Grunow

(M) *Tryblionella plana* (W.Smith) Pelletan
Records: 1 **Literature:** Tempère and Peragallo (1908) **Syn:** *Nitzschia plana* W.Smith

(FM) *Tryblionella punctata* W.Smith
Records: 2 **Literature:** Clemens (1933), Roelofs (1983) **Syn:** *Nitzschia punctata* (W.Smith) Grunow

(M) *Tryblionella victoriae* Grunow
Records: 1 **Literature:** Tempère and Peragallo (1908) **Syn:** *Nitzschia tryblionella* var. *victoriae* (Grunow) Grunow

Phylum: Bacillariophyta
Class: Bacillariophyceae
Subclass: Bacillariophycidae
Order: Cocconeidales
Family: Achnanthidiaceae

(F) *Achnantheiopsis fragilarioides* (J.B.Petersen) Lange-Bertalot
Records: 1 **Literature:** Roelofs (1983) **Syn:** *Achnanthes fragilarioides* J.B.Petersen

(F) *Achnanthidium affine* (Grunow) Czarnecki
Records: 1 **Literature:** Roelofs (1983) **Syn:** *Achnanthes affinis* Grunow

(F) *Achnanthidium exile* (Kützing) Heiberg
Records: 1 **Literature:** Bailey and MacKay (1916) **Syn:** *Achnanthes exilis* Kützing

(F) *Achnanthidium lineare* W.Smith
Records: 1 **Literature:** Roelofs (1983) **Syn:** *Achnanthes linearis* (W.Smith) Grunow

(F) *Achnanthidium minutissimum* (Kützing) Czarnecki
Records: 2 **Literature:** Bailey and MacKay (1916), Roelofs (1983) **Syn:** *Achnanthes minutissima* Kützing

(F) *Achnanthidium trinode* Ralfs
Records: 1 **Literature:** Tempère and Peragallo (1908) **Syn:** *Navicula trinodis* W.Smith, nom. illeg.

(F) *Gololobovia obliqua* (W.Gregory) Kulikovskiy, Glushchenko & Kociolek
Records: 1 **Literature:** Rao and Lewin (1976) **Syn:** *Achnanthes obliqua* (W.Gregory) Hustedt

(F) *Karayevia* sp. Round & Bukhtiyarova ex Round
Records: 7 **Molecular (amplicon):** IMERSS (ENA:ERS21395345)

(F) ***Lemnicola*** **sp. Round & Basson**
Records: 2 **Molecular (amplicon):** IMERSS (ENA:ERS21395352)
(F) ***Planothidium capitatum*** **(O.Müller) Van de Vijver, Kopalová, C.E.Wetzel & Ector**
Records: 1 **Literature:** Rao and Lewin (1976) **Syn:** *Achnanthes lanceolata* f. *capitata* (O.F.Müller) Hustedt
(F) ***Planothidium diplopunctatum*** **(Simonsen) Witkowski & Lange-Bertalot**
Records: 1 **Literature:** Rao and Lewin (1976) **Syn:** *Achnanthes diplopunctata* Simonsen
(F) ***Planothidium dubium*** **(Grunow) Round & Bukhtiyarova**
Records: 1 **Literature:** Roelofs (1983) **Syn:** *Achnanthes lanceolata* var. *dubia* Grunow
(F) ***Planothidium hauckianum*** **(Grunow) Bukhtiyarova**
Records: 3 **Literature:** Rao and Lewin (1976), Roelofs (1983), Tynni (1986) **Syn:** *Achnanthes hauckiana* Grunow
(F) ***Planothidium hauckianum*** **var.** ***rostratum*** **(P.Schulz ex Hustedt) Bukhtiyarova**
Records: 1 **Literature:** Tynni (1986) **Syn:** *Achnanthes hauckiana* var. *rostrata* P.Schulz ex Hustedt
(F) ***Planothidium haynaldii*** **(Schaarschmidt) Lange-Bertalot**
Records: 1 **Literature:** Tempère and Peragallo (1908) **Syn:** *Achnanthes lanceolata* var. *haynaldii* (Schaarschmidt) Cleve
(F) ***Planothidium lanceolatum*** **(Brébisson ex Kützing) Lange-Bertalot**
Records: 4 **Literature:** Bailey and MacKay (1916), Roelofs (1983), Shim (1976) **Morphology:** WL: iNat (262740438) **Syn:** *Achnanthes lanceolata* (Brébisson ex Kützing) Grunow, *Achnanthidium lanceolatum* Brébisson ex Kützing
(F) ***Psammothidium kryophilum*** **(J.B.Petersen) E.Reichardt**
Records: 1 **Literature:** Roelofs (1983) **Syn:** *Achnanthes kryophila* J.B.Petersen
(B) ***Psammothidium subsalsum*** **(J.B.Petersen) Kulikowskiy, Witkowski & Pliński**
Records: 1 **Literature:** Roelofs (1983) **Syn:** *Achnanthes subsalsoides* Hustedt

Phylum: Bacillariophyta
Class: Bacillariophyceae
Subclass: Bacillariophycidae
Order: Cocconeidales
Family: Cocconeidaceae
(M) ***Amphicocconeis disculoides*** **(Hustedt) Stefano & Marino**
Records: 3 **Literature:** Roelofs (1983), Shim (1976), Tynni (1986) **Syn:** *Cocconeis disculoides* Hustedt
(M) ***Campyloneis grevillei*** **(W.Smith) Grunow & Eulenstein**
Records: 2 **Literature:** Bailey and MacKay (1916), Clemens (1933) **Syn:** *Campyloneis grevillei* var. *argus* (Grunow) Cleve
(M) ***Cocconeis antiqua*** **Tempère & Brun**
Records: 1 **Literature:** Tempère and Peragallo (1908)
(M) ***Cocconeis britannica*** **Naegeli ex Kützing**
Records: 1 **Literature:** Tempère and Peragallo (1908)
(M) ***Cocconeis californica*** **Grunow**
Records: 2 **Literature:** Rao and Lewin (1976), Tynni (1986)
(M) ***Cocconeis clandestina*** **A.W.F.Schmidt**
Records: 3 **Literature:** Roelofs (1983), Shim (1976), Tynni (1986)
(M) ***Cocconeis costata*** **W.Gregory**
Records: 9 **Literature:** Bailey and MacKay (1916), Clemens (1933), McQuoid and Hobson (1997), Rao and Lewin (1976), Roelofs (1983), Shim (1976), Tempère and Peragallo (1908) **Morphology:** WL: iNat (259737079), M (184750 / 462231 / E 5573)
(M) ***Cocconeis costata*** **var.** ***hexagona*** **Grunow ✦**

Records: 3 **Morphology:** WL: iNat (259359045) Voucher (WL-SEM-75, WL-LM-24)

(M) ***Cocconeis decipiens*** **Cleve**
Records: 2 **Literature:** McQuoid and Hobson (2001), Shim (1976)

(M) ***Cocconeis diaphana*** **W.Smith**
Records: 1 **Literature:** Lord (1866)

(M) ***Cocconeis dirupta*** **W.Gregory**
Records: 7 **Literature:** Bailey and MacKay (1916), Clemens (1933), Lord (1866), Shim (1976) **Morphology:** WL: iNat (263109390), M (184750 / 462232 / E 5574) **Syn:** *Cocconeis dirupta* Meunier

(M) ***Cocconeis dirupta*** **var.** ***flexella*** **(Janisch & Rabenhorst) Grunow**
Records: 2 **Literature:** Bailey and MacKay (1916), Tempère and Peragallo (1908)

(F) ***Cocconeis disculus*** **(Schumann) Cleve**
Records: 2 **Literature:** Rao and Lewin (1976), Shim (1976)

(FM) ***Cocconeis distans*** **W.Gregory**
Records: 4 **Literature:** Bailey and MacKay (1916), Clemens (1933), Lord (1866), Rao and Lewin (1976) **Syn:** *Cocconeis granulifera* Greville

(F) ***Cocconeis euglypta*** **Ehrenberg**
Records: 2 **Literature:** Rao and Lewin (1976), Roelofs (1983) **Syn:** *Cocconeis placentula* var. *euglypta* (Ehrenberg) Cleve

(M) ***Cocconeis fasciolata*** **(Ehrenberg) N.E.Brown**
Records: 3 **Literature:** Rao and Lewin (1976) **Morphology:** WL: iNat (259732583) Voucher (WL-SEM-83, WL-LM-28) **Syn:** *Cocconeis costata* var. *pacifica* (Grunow) Cleve

(F) ***Cocconeis fluviatilis*** **J.H.Wallace**
Records: 1 **Literature:** Roelofs (1983)

(M) ***Cocconeis grata*** **A.W.F.Schmidt ex Romero & Riaux-Gobin**
Records: 2 **Literature:** Bailey and MacKay (1916), Clemens (1933)

(M) ***Cocconeis heteroidea*** **var.** ***curvirotunda*** **(Tempère & J.-J.Brun) Cleve**
Records: 1 **Literature:** Bailey and MacKay (1916) **Syn:** *Cocconeis curvirotunda* Tempère & Brun

(M) ***Cocconeis interrupta*** **Grunow**
Records: 2 **Literature:** Bailey and MacKay (1916), Clemens (1933)

(M) ***Cocconeis kerguelensis*** **P.Petit ✦**
Records: 1 **Morphology:** WL: iNat (259379467) Voucher (WL-LM-26)

(M) ***Cocconeis lineata*** **Ehrenberg**
Records: 2 **Literature:** Clemens (1933), Tempère and Peragallo (1908) **Syn:** *Cocconeis placentula* var. *lineata* (Ehrenberg) Van Heurck

(FM) ***Cocconeis maxima*** **(Grunow) H.Peragallo & M.Peragallo**
Records: 2 **Literature:** Rao and Lewin (1976), Tempère and Peragallo (1908)

(M) ***Cocconeis molesta*** **Kützing**
Records: 1 **Literature:** Clemens (1933)

(F) ***Cocconeis molesta*** **var.** ***amygdalina*** **(Brébisson ex Van Heurck) Cleve**
Records: 1 **Literature:** Bailey and MacKay (1916)

(FM) ***Cocconeis notata*** **P.Petit ✦**
Records: 1 **Morphology:** WL: iNat (259450952) Voucher (WL-LM-20)

(M) ***Cocconeis ornata*** **W.Gregory**
Records: 1 **Literature:** Shim (1976)

(F) ***Cocconeis pediculus*** **Ehrenberg**
Records: 1 **Literature:** Bailey and MacKay (1916)

(M) ***Cocconeis pellucida*** **Grunow**
Records: 7 **Literature:** Rao and Lewin (1976), Roelofs (1983), Shim (1976), Tempère and Peragallo (1908), Tynni (1986) **Morphology:** M (184750 / 462229 / E 5571)

(FM) ***Cocconeis pinnata*** **W.Gregory ex Greville**

Records: 3 **Literature:** Bailey and MacKay (1916), Clemens (1933) **Morphology:** WL: iNat (264547415)

(F) ***Cocconeis placentula*** **Ehrenberg**
Records: 8 **Literature:** Lord (1866), Rao and Lewin (1976), Roelofs (1983), Tempère and Peragallo (1908) **Morphology:** BRU (ABRU00000062), MSC (MSC0182075), MU (000179518), NY (02113174)

(M) ***Cocconeis placentula*** **var.** ***klinoraphis*** **Geitler**
Records: 1 **Literature:** Shim (1976)

(M) ***Cocconeis pseudomarginata*** **W.Gregory**
Records: 5 **Literature:** Bailey and MacKay (1916), Clemens (1933), Lord (1866), Roelofs (1983), Shim (1976) Notes: orth. corr. '*Cononeis pseudomarginata*'

(M) ***Cocconeis pseudomarginata*** **var.** ***intermedia*** **Grunow ✦**
Records: 1 **Morphology:** WL: iNat (259455426) Voucher (WL-SEM-77)

(M) ***Cocconeis riparia*** **J.-J.Brun**
Records: 1 **Literature:** Bailey and MacKay (1916) **Syn:** *Cocconeis scutellum* var. *riparia* (Brun in Schmidt et al.) Peragallo & Peragallo

(M) ***Cocconeis scutellum*** **Ehrenberg**
Records: 11 **Literature:** Bailey and MacKay (1916), Clemens (1933), Lord (1866), McQuoid and Hobson (1997), Rao and Lewin (1976), Roelofs (1983), Shim (1976), Tempère and Peragallo (1908), Tynni (1986) **Morphology:** iNat (214411213), M (184750 / 462230 / E 5572) **Syn:** *Cocconeis scutellum* var. *baldjikiana* (Grunow) Cleve

(M) ***Cocconeis scutellum*** **var.** ***adjuncta*** **A.W.F.Schmidt**
Records: 1 **Literature:** Bailey and MacKay (1916)

(M) ***Cocconeis scutellum*** **var.** ***morrisii*** **(W.Smith ex Greville) H.Peragalllo & M.Peragallo**
Records: 2 **Literature:** Bailey and MacKay (1916), Tynni (1986) **Syn:** *Cocconeis morrisii* W.Smith ex Greville, *Cocconeis scutellum* var. *baldjikiana* (Grunow) Cleve

(FM) ***Cocconeis scutellum*** **var.** ***parva*** **(Grunow) Cleve**
Records: 3 **Literature:** Bailey and MacKay (1916), Shim (1976), Waters et al. (1992)

(M) ***Cocconeis scutellum*** **var.** ***posidoniae*** **M.De Stefano, D.Marino & L.Mazzella ✦**
Records: 1 **Morphology:** WL: iNat (259478500) Voucher (WL-LM-37)

(M) ***Cocconeis sigmoradians*** **Tempère & Brun**
Records: 1 **Literature:** Tempère and Peragallo (1908)

(M) ***Cocconeis stauroneiformis*** **H.Okuno**
Records: 9 **Literature:** Bailey and MacKay (1916) **Morphology:** WL: iNat (259496100) Voucher (WL-SEM-67) **Molecular (amplicon):** IMERSS (ENA:ERS21395345) **Syn:** *Cocconeis scutellum* var. *stauroneiformis* Grunow, nom. illeg.

(FM) ***Cocconeis sublittoralis*** **Hendey**
Records: 1 **Literature:** Roelofs (1983)

(M) ***Cocconeis versicolor*** **Brun**
Records: 1 **Literature:** Tempère and Peragallo (1908)

Phylum: Bacillariophyta
Class: Bacillariophyceae
Subclass: Bacillariophycidae
Order: Cymbellales
Family: Anomoeoneidaceae

(F) ***Adlafia minuscula*** **(Grunow) Lange-Bertalot**
Records: 1 **Literature:** Bailey and MacKay (1916) **Syn:** *Navicula minuscula* Grunow

(B) ***Anomoeoneis costata*** **(Kützing) Hustedt**
Records: 1 **Literature:** Tempère and Peragallo (1908) **Syn:** *Navicula bohemica* Ehrenberg

(B) ***Staurophora amphioxys*** **(W.Gregory) D.G.Mann**
Records: 3 **Literature:** Bailey and MacKay (1916), Rao and Lewin (1976), Tempère and Peragallo (1908) **Syn:** *Stauroneis amphioxys* W.Gregory, *Stauroneis gregoryi* Ralfs

(B) ***Staurophora dubitabilis*** **(Hustedt) Clavero & Hernández-Mariné**
Records: 1 **Literature:** Rao and Lewin (1976) **Syn:** *Stauroneis dubitabilis* Hustedt

(B) ***Staurophora elata*** **(Hustedt ex Simonsen) D.G.Mann**
Records: 1 **Literature:** Rao and Lewin (1976) **Syn:** *Stauroneis elata* Hustedt ex Simonsen

(B) ***Staurophora salina*** **(W.Smith) Mereschkowsky**
Records: 1 **Literature:** Tempère and Peragallo (1908)

(B) ***Staurophora wislouchii*** **(Poretzsky & Anisimova) D.G.Mann**
Records: 1 **Literature:** Rao and Lewin (1976) **Syn:** *Stauroneis wislouchii* V.S.Poretzky & Anisimova

Phylum: Bacillariophyta
Class: Bacillariophyceae
Subclass: Bacillariophycidae
Order: Cymbellales
Family: Cymbellaceae

(FM) ***Cymbella angusta*** **(Gregory) Gusliakov, nom. illeg.**
Records: 3 **Literature:** Bailey and MacKay (1916), Clemens (1933), Rao and Lewin (1976) **Syn:** *Amphora angusta* W.Gregory

(F) ***Cymbella aspera*** **(Ehrenberg) Cleve**
Records: 3 **Literature:** Bailey and MacKay (1916), Shim (1976), Tempère and Peragallo (1908) **Syn:** *Cymbella gastroides* (Kützing) Brébisson & Godey

(F) ***Cymbella cistula*** **(Ehrenberg) O.Kirchner**
Records: 2 **Literature:** Shim (1976), Tempère and Peragallo (1908)

(F) ***Cymbella lanceolata*** **C.Agardh**
Records: 3 **Literature:** Bailey and MacKay (1916), Rao and Lewin (1976), Roelofs (1983) **Syn:** *Navicula lanceolata* (C.Agardh) Kützing, nom. illeg.

(F) ***Cymbella mexicana*** **(Ehrenberg) Cleve**
Records: 2 **Literature:** Roelofs (1983), Tempère and Peragallo (1908)

(F) ***Cymbella producta*** **M.Peragallo**
Records: 1 **Literature:** Tempère and Peragallo (1908)

(F) ***Cymbopleura anglica*** **(Lagerstedt) Krammer**
Records: 1 **Literature:** Tempère and Peragallo (1908) **Syn:** *Cymbella anglica* Lagerstedt

(F) ***Cymbopleura cuspidata*** **(Kützing) Krammer**
Records: 1 **Literature:** Tempère and Peragallo (1908)

(F) ***Cymbopleura heteropleura*** **(Ehrenberg) Z.X.Shi**
Records: 1 **Literature:** Tempère and Peragallo (1908) **Syn:** *Cymbella heteropleura* (Ehrenberg) Kützing

(F) ***Cymbopleura inaequalis*** **(Ehrenberg) Krammer**
Records: 1 **Literature:** Tempère and Peragallo (1908) **Syn:** *Cymbella ehrenbergii* Kützing
(F) ***Didymosphenia geminata*** **(Lyngbye) Mart.Schmidt ✦**
Records: 2 **Morphology:** WL: iNat (264211715)
(F) ***Encyonopsis microcephala*** **(Grunow) Krammer**
Records: 1 **Literature:** Roelofs (1983) **Syn:** *Cymbella microcephala* Grunow
(F) ***Placoneis elginensis*** **(W.Gregory) E.J.Cox**
Records: 1 **Literature:** Tempère and Peragallo (1908)

Phylum: Bacillariophyta
Class: Bacillariophyceae
Subclass: Bacillariophycidae
Order: Cymbellales
Family: Gomphonemataceae
(F) ***Encyonema cespitosum*** **Kützing**
Records: 1 **Literature:** Tempère and Peragallo (1908)
(F) ***Encyonema gibbum*** **(Bailey) Krammer**
Records: 1 **Literature:** Tempère and Peragallo (1908) **Syn:** *Cymbella gibba* Bailey
(F) ***Encyonema gracile*** **Rabenhorst**
Records: 1 **Literature:** Bailey and MacKay (1916)
(F) ***Encyonema minutum*** **(Hilse) D.G.Mann**
Records: 1 **Literature:** Roelofs (1983) **Syn:** *Cymbella minuta* Hilse
(F) ***Encyonema silesiacum*** **(Bleisch) D.G.Mann**
Records: 1 **Literature:** Roelofs (1983) **Syn:** *Cymbella minuta* var. *silesiaca* (Bleisch) Reimer
(F) ***Encyonema ventricosum*** **(C.Agardh) Grunow**
Records: 1 **Literature:** Tempère and Peragallo (1908)
(F) ***Gomphadelpha herculeana*** **(Ehrenb.) R.Jahn & N.Abarca**
Records: 1 **Literature:** Tempère and Peragallo (1908) **Syn:** *Gomphonema herculeanum* Ehrenberg
(F) ***Gomphadelpha herculeana*** **var.** ***robusta*** **(Grunow) R.Jahn & N.Abarca**
Records: 1 **Literature:** Tempère and Peragallo (1908) **Syn:** *Gomphonema herculeanum* var. *robustum* Grunow
(F) ***Gomphonema acuminatum*** **Ehrenberg**
Records: 3 **Literature:** Roelofs (1983), Tempère and Peragallo (1908)
Morphology: WL: iNat (9291305)
(F) ***Gomphonema capitatum*** **Ehrenberg**
Records: 1 **Literature:** Tempère and Peragallo (1908) **Syn:** *Gomphonema constrictum* var. *capitatum* (Ehrenberg) Grunow
(F) ***Gomphonema constrictum*** **Ehrenberg**
Records: 2 **Literature:** Roelofs (1983), Tempère and Peragallo (1908)
(F) ***Gomphonema intricatum*** **Kützing**
Records: 1 **Literature:** Bailey and MacKay (1916)
(F) ***Gomphonema longiceps*** **f.** ***suecicum*** **(Grunow) Hustedt**
Records: 1 **Literature:** Tempère and Peragallo (1908) **Syn:** *Gomphonema montanum* var. *suecicum* Grunow (as '*suecica*')
(F) ***Gomphonema subcapitatum*** **(Grunow) E.Reichardt & Levkov**
Records: 1 **Literature:** Tempère and Peragallo (1908) **Syn:** *Gomphonema constrictum* var. *subcapitatum* Grunow
(F) ***Gomphonema subclavatum*** **(Grunow) Grunow**
Records: 1 **Literature:** Bailey and MacKay (1916)
(F) ***Gomphonema ventricosum*** **W.Gregory**
Records: 1 **Literature:** Tempère and Peragallo (1908)

Phylum: Bacillariophyta
Class: Bacillariophyceae
Subclass: Bacillariophycidae
Order: Cymbellales
Family: Rhoicospheniaceae

(M) *Chelonicola* sp. Majewska, De Stefano & Van de Vijver
Records: 1 **Molecular (amplicon):** IMERSS (ENA:ERS27630062)

(M) *Gomphonemopsis exigua* (Kützing) Medlin
Records: 2 **Literature:** Bailey and MacKay (1916) **Morphology:** WL: iNat (189517016) **Syn:** *Gomphonema exiguum* Kützing

(M) *Gomphonemopsis pseudexigua* Medlin ✦
Records: 14 **Morphology:** WL: iNat (189577227) Voucher (WL-SEM-118) **Molecular (amplicon):** IMERSS (ENA:ERS27214058)

(M) *Gomphoseptatum aestuarii* (Cleve) Medlin ✦
Records: 1 **Morphology:** WL: iNat (258910079) Voucher (WL-SEM-103)

(M) *Gomphoseptatum pseudoseptatum* (Giffen) Witkowski, Lange-Bertalot & Metzeltin ✦
Records: 1 **Morphology:** WL: iNat (258910629) Voucher (WL-SEM-107)

(M) *Rhoicosphenia abbreviata* (C.Agardh) Lange-Bertalot
Records: 8 **Literature:** Bailey and MacKay (1916), Chester et al. (1979), McQuoid and Hobson (1997), McQuoid and Hobson (2001), Rao and Lewin (1976), Roelofs (1983), Shim (1976), Tempère and Peragallo (1908) **Syn:** *Rhoicosphenia curvata* (Kützing) Grunow

(M) *Rhoicosphenia genuflexa* (Kützing) Medlin
Records: 1 **Literature:** Bailey and MacKay (1916) **Notes:** Bailey and MacKay (1916) report *Rhoiconeis genuflexa* (Ktz.) Grun. ≠ *Rhoicosphenia genuflexa* (assumed to be a typo) **Syn:** *Rhoikoneis genuflexa* (Ktz.) Grun.

(M) *Rhoicosphenia marina* (Kützing) M.Schmidt
Records: 5 **Literature:** Clemens (1933), Lord (1866), Tempère and Peragallo (1908) **Morphology:** WL: iNat (189880467) **Syn:** *Gomphonema marinum* (Kützing) W.Smith, *Rhoicosphenia curvata* var. *marina* (Kützing) Grunow

Phylum: Bacillariophyta
Class: Bacillariophyceae
Subclass: Bacillariophycidae
Order: Lyrellales
Family: Lyrellaceae

(M) *Lyrella abrupta* (W.Gregory) D.G.Mann
Records: 2 **Literature:** Bailey and MacKay (1916), Clemens (1933) **Syn:** *Navicula abrupta* (W.Gregory) Donkin

(M) *Lyrella californica* (Greville) D.G.Mann
Records: 1 **Literature:** Tempère and Peragallo (1908)

(M) *Lyrella clavata* (W.Gregory) D.G.Mann
Records: 3 **Literature:** Bailey and MacKay (1916), Tempère and Peragallo (1908) **Morphology:** WL: iNat (263508735) **Syn:** *Navicula clavata* W.Gregory

(M) *Lyrella clavata* var. *caribaea* (Cleve) Siqueiros Beltrones
Records: 1 **Literature:** Bailey and MacKay (1916) **Syn:** *Navicula clavala* var. *caribaea*

(M) *Lyrella david-mannii* Witkowski, Lange-Bertalot & Metzeltin
Records: 1 **Literature:** Tempère and Peragallo (1908) **Syn:** *Navicula lyra* var. *elliptica* A.W.F.Schmidt

(M) *Lyrella dilatata* (A.W.F.Schmidt) E.Nevrova, Witkowski, M.Kulikovskiy & Lange-Bertalot
Records: 1 **Literature:** Tempère and Peragallo (1908) **Syn:** *Navicula lyra* var. *dilatata* A.W.F.Schmidt

(M) *Lyrella hennedyi* (W.Smith) Stickle & D.G.Mann

Records: 2 **Literature:** Bailey and MacKay (1916), Shim (1976) **Syn:** *Navicula hennedyi* W.Smith (as '*Hennedyi*' by Bailey and MacKay, 1916)

(M) *Lyrella lyra* (Ehrenberg) Karayeva
Records: 5 **Literature:** Bailey and MacKay (1916), Clemens (1933), Rao and Lewin (1976), Shim (1976), Tempère and Peragallo (1908) **Syn:** *Navicula lyra* Ehrenberg

(M) *Lyrella spectabilis* (W.Gregory) D.G.Mann
Records: 1 **Literature:** Tempère and Peragallo (1908) **Syn:** *Navicula spectabilis* W.Gregory

(M) *Petroneis glacialis* (Cleve) Witkowski, Lange-Bertalot & Metzeltin
Records: 1 **Literature:** Shim (1976) **Syn:** *Navicula glacialis* (Cleve) Cleve

(M) *Petroneis granulata* D.G.Mann, nom. illeg.
Records: 4 **Literature:** Rao and Lewin (1976), Tempère and Peragallo (1908), Tynni (1986) **Morphology:** WL: iNat (189466966) **Syn:** *Navicula granulata* Bailey, nom. illeg.

(M) *Petroneis humerosa* (Brébisson ex W.Smith) Stickle & D.G.Mann
Records: 5 **Literature:** Bailey and MacKay (1916), Clemens (1933), Tempère and Peragallo (1908) **Molecular (amplicon):** IMERSS (ENA:ERS21395352) **Syn:** *Navicula humerosa* Brébisson ex W.Smith

(M) *Petroneis latissima* (W.Gregory) A.J.Stickle & D.G.Mann
Records: 2 **Literature:** Shim (1976), Tynni (1986) **Syn:** *Navicula latissima* W.Gregory

Phylum: Bacillariophyta
Class: Bacillariophyceae
Subclass: Bacillariophycidae
Order: Mastogloiales
Family: Achnanthaceae

(FM) *Achnanthes adnata* Bory
Records: 7 **Literature:** Bailey and MacKay (1916), Clemens (1933), Lord (1866), Roelofs (1983), Tynni (1986) **Morphology:** WL: iNat (192066595) Voucher (WL-SEM-33) **Syn:** *Achnanthes brevipes* C.Agardh, nom. illeg.

(M) *Achnanthes armillaris* (O.F.Müller) Guiry
Records: 10 **Literature:** Bailey and MacKay (1916), Buchanan (1966), Clemens (1933), Rao and Lewin (1976), Roelofs (1983), Shim (1976), Stockner and Cliff (1976), Tempère and Peragallo (1908), Waters et al. (1992) **Morphology:** WL: iNat (191996286) Voucher (WL-SEM-30, WL-LM-33) **Syn:** *Achnanthes longipes* Agardh

(B) *Achnanthes brevipes* var. *intermedia* (Kützing) Cleve
Records: 2 **Literature:** Clemens (1933) **Morphology:** WL: iNat (192250763) Voucher (WL-SEM-7, WL-LM-32) **Syn:** *Achnanthes subsessilis* Kützing; *Achnanthes brevipes* H.Peragallo & M.Peragallo conserved against *A. adnata* Bory

(M) *Achnanthes brevipes* var. *minor* H.Peragallo
Records: 1 **Literature:** Bailey and MacKay (1916) **Notes:** *Achnanthes brevipes* H.Peragallo & M.Peragallo conserved against *A. adnata* Bory

(M) *Achnanthes* cf. *subconstricta* (F.Meister) K.Toyoda
Records: 1 **Morphology:** WL: iNat (50191595)

(FM) *Achnanthes groenlandica* (Cleve) Grunow
Records: 2 **Literature:** Rao and Lewin (1976), Tynni (1986)

(M) *Achnanthes groenlandica* var. *meridiana* Giffen ✦
Records: 2 **Morphology:** WL: iNat (323108312) Voucher (WL-SEM-108)

(F) *Achnanthes lemmermannii* Hustedt
Records: 1 **Literature:** Roelofs (1983)

(FM) *Achnanthes parvula* Kützing
Records: 2 **Literature:** Bailey and MacKay (1916), Clemens (1933)

(F) ***Platessa conspicua*** **(Ant.Mayer) Lange-Bertalot**
Records: 1 **Literature:** Roelofs (1983) **Syn:** *Achnanthes conspicua* Ant.Mayer

Phylum: Bacillariophyta
Class: Bacillariophyceae
Subclass: Bacillariophycidae
Order: Mastogloiales
Family: Mastogloiaceae
(M) ***Mastogloia exigua*** **F.W.Lewis**
Records: 3 **Literature:** Bailey and MacKay (1916), Roelofs (1983)
Morphology: WL: iNat (324981051)
(M) ***Mastogloia ovata*** **Grunow**
Records: 1 **Literature:** Shim (1976)
(M) ***Mastogloia splendida*** **(Gregory) H.Pergallo**
Records: 1 **Literature:** Lord (1866) **Syn:** *Cocconeis splendida* W.Gregory

Phylum: Bacillariophyta
Class: Bacillariophyceae
Subclass: Bacillariophycidae
Order: Mastogloiales
Family: Stauroneidaceae
(M) ***Astartiella stroemii*** **(Hustedt) Bukhtiyarova**
Records: 1 **Literature:** Rao and Lewin (1976) **Syn:** *Achnanthes stroemii* Hustedt (as '*strömi*')
(F) ***Craticula ambigua*** **(Ehrenberg) D.G.Mann**
Records: 1 **Literature:** Clemens (1933) **Syn:** *Navicula ambigua* Ehrenberg
(F) ***Prestauroneis*** **cf.** ***integra*** **(W.Smith) Bruder**
Records: 18 **Molecular (amplicon):** IMERSS (ENA:ERS21395352)
(M) ***Prestauroneis crucicula*** **(W.Smith) Genkal & Yarushina**
Records: 1 **Literature:** Rao and Lewin (1976) **Syn:** *Navicula crucicula* (W.Smith) Donkin (as unrecognized '*Navicula crucicula* f. *rostrata* Tarnavschi')
(F) ***Stauroneis acuta*** **W.Smith**
Records: 1 **Literature:** Tempère and Peragallo (1908)
(F) ***Stauroneis gracilis*** **Ehrenberg**
Records: 1 **Literature:** Tempère and Peragallo (1908)
(F) ***Stauroneis phoenicenteron*** **(Nitzsch) Ehrenberg**
Records: 2 **Literature:** Tempère and Peragallo (1908), Tynni (1986)
(F) ***Stauroneis pteroidea*** **Ehrenberg**
Records: 1 **Literature:** Tempère and Peragallo (1908)

Phylum: Bacillariophyta
Class: Bacillariophyceae
Subclass: Bacillariophycidae
Order: Naviculales
Family: incertae sedis
(M) ***Fogedia finmarchica*** **(Cleve & Grunow) Witkowski, Metzeltin & Lange-Bertalot**
Records: 1 **Literature:** Rao and Lewin (1976) **Syn:** *Navicula finmarchica* (Cleve & Grunow) Cleve (as '*finnmarchica*')
(M) ***Fogedia krammeri*** **Witkowski, Lange-Bertalot, Kociolek & M.Kulikovskiy ✦**
Records: 2 **Morphology:** WL: iNat (312508075) Voucher (WL-SEM-94)
(F) ***Kobayasiella subtilissima*** **(Cleve) Lange-Bertalot**
Records: 1 **Literature:** Roelofs (1983) **Syn:** *Navicula subtilissima* Cleve

Phylum: Bacillariophyta

Class: Bacillariophyceae
Subclass: Bacillariophycidae
Order: Naviculales
Family: Amphipleuraceae
(FM) *Frustulia amphipleuroides* (Grunow) A.Cleve
Records: 1 **Literature:** Tempère and Peragallo (1908)
(F) *Frustulia rhomboides* (Ehrenberg) De Toni
Records: 4 **Literature:** Bailey and MacKay (1916), Buchanan (1966), Shim (1976), Tempère and Peragallo (1908) **Syn:** *Navicula rhomboides* Ehrenberg

Phylum: Bacillariophyta
Class: Bacillariophyceae
Subclass: Bacillariophycidae
Order: Naviculales
Family: Berkeleyaceae
(M) *Berkeleya rutilans* (Trentepohl ex Roth) Grunow
Records: 46 **Literature:** Lobban (1985), Rao and Lewin (1976) **Morphology:** WL: iNat (255733193) Voucher (WL-SEM-10, WL-SEM-32, WL-SEM-80) NHA (NHA-655680), UBC (A034671), UC (UC1057045) **Molecular (amplicon):** IMERSS (ENA:ERS27639099) **Syn:** *Amphipleura rutilans* (Trentepohl ex Roth) Cleve
(M) *Parlibellus berkeleyi* (Kützing) E.J.Cox
Records: 20 **Literature:** Lobban (1985) **Morphology:** UBC (A032617) **Syn:** *Navicula pseudocomoides* Hendey
(M) *Parlibellus delognei* (Van Heurck) E.J.Cox
Records: 12 **Literature:** Lobban (1985) **Morphology:** WL: iNat (256407711) Voucher (WL-SEM-15, WL-SEM-16), UBC (A033418) **Molecular (amplicon):** IMERSS (ENA:ERS27630061) **Syn:** *Navicula delognei* Van Heurck
(M) *Parlibellus delognei* f. *ellipticus* (Lobban) E.J.Cox ✦
Records: 73 **Morphology:** WL: iNat (256411671) Voucher (WL-SEM-16, WL-LM-29) **Molecular (amplicon):** EBI (ENA:JX905787), IMERSS (ENA:ERS21395346) **Syn:** *Parlibellus delognei* f. *elliptica* (Lobban) E.J.Cox
(M) *Parlibellus hamulifer* (Grunow) E.J.Cox
Records: 2 **Literature:** Rao and Lewin (1976) **Morphology:** WL: iNat (257182702) **Syn:** *Navicula hamulifera* Grunow
(M) *Parlibellus rhombicus* (W.Gregory) E.J.Cox
Records: 6 **Literature:** Bailey and MacKay (1916), Clemens (1933), Lord (1866), Rao and Lewin (1976), Tempère and Peragallo (1908), Tynni (1986) **Syn:** *Navicula rhombica* W.Gregory

Phylum: Bacillariophyta
Class: Bacillariophyceae
Subclass: Bacillariophycidae
Order: Naviculales
Family: Brachysiraceae
(F) *Brachysira microcephala* (Grunow) Compère
Records: 1 **Literature:** Roelofs (1983) **Syn:** *Navicula microcephala* Grunow

Phylum: Bacillariophyta
Class: Bacillariophyceae
Subclass: Bacillariophycidae
Order: Naviculales
Family: Diadesmidaceae
(M) *Luticola cohnii* (Hilse) D.G.Mann
Records: 1 **Literature:** Tynni (1986) **Syn:** *Navicula mutica* var. *cohnii* (Hilse) Grunow

Phylum: Bacillariophyta
Class: Bacillariophyceae
Subclass: Bacillariophycidae
Order: Naviculales
Family: Diploneidaceae
(M) *Diploneis advena* (A.W.F.Schmidt) Cleve
Records: 2 **Literature:** Bailey and MacKay (1916), Clemens (1933) **Syn:** *Navicula advena* A.W.F.Schmidt
(M) *Diploneis bombus* (Ehrenberg) Ehrenberg
Records: 4 **Literature:** Bailey and MacKay (1916), Clemens (1933), Lord (1866), Tempère and Peragallo (1908) **Syn:** *Navicula bombus* (Ehrenberg) Kützing, *Navicula didyma* A.W.F.Schmidt
(F) *Diploneis* cf. *petersenii* Hustedt
Records: 1 **Literature:** Roelofs (1983)
(M) *Diploneis constricta* Cleve
Records: 1 **Literature:** Bailey and MacKay (1916) **Syn:** *Navicula constricta* Grunow, nom. illeg.
(M) *Diploneis crabro* (Ehrenberg) Ehrenberg
Records: 4 **Literature:** Clemens (1933), Lord (1866), Roelofs (1983) **Morphology:** WL: iNat (262752077) **Syn:** *Navicula crabro* (Ehrenberg) Kützing
(M) *Diploneis crabro* var. *minuta* Cleve
Records: 1 **Literature:** Bailey and MacKay (1916) **Syn:** *Navicula multicostata* var. *minuta* (Cleve) H.Perag. & Perag.
(FM) *Diploneis didymus* (Ehrenberg) Ehrenberg
Records: 1 **Literature:** Tempère and Peragallo (1908) **Notes:** orth. corr. '*Diploneis didyma*'
(FM) *Diploneis elliptica* (Kützing) Cleve
Records: 2 **Literature:** Bailey and MacKay (1916), Tempère and Peragallo (1908) **Syn:** *Navicula elliptica* Kützing
(F) *Diploneis elliptica* var. *magnapunctata* Fontell
Records: 1 **Literature:** Tynni (1986)
(F) *Diploneis exemta* (A.W.F.Schmidt) Cleve ✦✦
Records: 1 **Morphology:** WL: iNat (266785298) Voucher (WL-SEM-92)
(F) *Diploneis finnica* (Ehrenberg) Cleve
Records: 1 **Literature:** Tempère and Peragallo (1908) **Syn:** *Navicula antinitescens* M.Peragallo
(M) *Diploneis interrupta* Cleve
Records: 3 **Literature:** Rao and Lewin (1976), Tempère and Peragallo (1908), Tynni (1986)
(M) *Diploneis lineata* (Donkin) Cleve
Records: 1 **Literature:** Shim (1976)
(F) *Diploneis mereschkowskyi* Lange-Bertalot, Fuhrmann & Werum
Records: 1 **Literature:** Tynni (1986) **Syn:** *Diploneis smithii* var. *rhombica* Mereschkowsky
(FM) *Diploneis smithii* (Brébisson) Cleve

Records: 6 **Literature:** Bailey and MacKay (1916), Clemens (1933), Lord (1866), Roelofs (1983), Tempère and Peragallo (1908), Tynni (1986) **Molecular (amplicon):** IMERSS (ENA:ERS21395345) **Syn:** *Navicula smithii* Brébisson

(M) ***Diploneis smithii* var. *scutellum* (O'Meara) F.W.Mills**

Records: 1 **Literature:** Bailey and MacKay (1916) **Syn:** *Navicula smithii* var. *scutellum* (O'Meara) Van Heurck

(M) ***Diploneis splendida* Cleve**

Records: 2 **Literature:** Bailey and MacKay (1916), Clemens (1933) **Syn:** *Navicula splendida* W.Gregory, nom. illeg.

(M) ***Diploneis suborbicularis* (W.Gregory) Cleve**

Records: 2 **Literature:** Bailey and MacKay (1916), Clemens (1933) **Syn:** *Navicula suborbicularis* (W.Gregory) Donkin

(FM) ***Diploneis subovalis* Cleve**

Records: 2 **Literature:** Rao and Lewin (1976), Tynni (1986)

(M) ***Diploneis vacillans* (A.W.F.Schmidt) Cleve**

Records: 2 **Literature:** Bailey and MacKay (1916), Clemens (1933) **Syn:** *Navicula vacillans* A.W.F.Schmidt

(M) ***Diploneis vacillans* var. *delicatula* Cleve**

Records: 1 **Literature:** Bailey and MacKay (1916) **Notes:** reported as unrecognized variety '*N.* v. *delicatula* Cleve'

(M) ***Diploneis vacillans* var. *minuta* Grunow**

Records: 1 **Literature:** Bailey and MacKay (1916) **Notes:** reported as unrecognized variety '*N.* v. *minuta* Grun.'

(M) ***Diploneis vetula* (A.W.F.Schmidt) Cleve**

Records: 1 **Literature:** Shim (1976)

Phylum: Bacillariophyta
Class: Bacillariophyceae
Subclass: Bacillariophycidae
Order: Naviculales
Family: Metascolioneidaceae

(B) ***Metascolioneis tumida* (Brébisson ex Kützing) Blanco & Wetzel**

Records: 2 **Literature:** Bailey and MacKay (1916), Tempère and Peragallo (1908) **Syn:** *Scoliopleura tumida* (Brébisson ex Kützing) Rabenhorst

Phylum: Bacillariophyta
Class: Bacillariophyceae
Subclass: Bacillariophycidae
Order: Naviculales
Family: Naviculaceae

(M) ***Austariella jamalinensis* (Cleve) Witkowski, Lange-Bertalot & Metzeltin**

Records: 1 **Literature:** Rao and Lewin (1976) **Syn:** *Navicula jamalinensis* Cleve

(F) ***Caloneis alpestris* (Grunow) Cleve**

Records: 1 **Literature:** Roelofs (1983)

(M) ***Caloneis brevis* (W.Gregory) Cleve**

Records: 4 **Literature:** Bailey and MacKay (1916), Clemens (1933), Rao and Lewin (1976), Tempère and Peragallo (1908) **Syn:** *Navicula brevis* W.Gregory

(M) ***Caloneis brevis* var. *elliptica* (Van Heurck) F.W.Mills**

Records: 2 **Literature:** Bailey and MacKay (1916), Tempère and Peragallo (1908) **Syn:** *Navicula brevis* var. *elliptica* Van Heurck

(M) ***Caloneis brevis* var. *vexans* (Grunow) Cleve**

Records: 1 **Literature:** Shim (1976)

(F) ***Caloneis latiuscula* (Kützing) Cleve**

Records: 1 **Literature:** Shim (1976)

(M) ***Caloneis liber* (W.Smith) Cleve**

Records: 4 **Literature:** Bailey and MacKay (1916), Clemens (1933), Tempère and Peragallo (1908) **Morphology:** WL: iNat (254372216) Voucher (WL-LM-14) **Syn:** *Navicula liber* W.Smith

(F) ***Caloneis limosa*** **(Kützing) R.M.Patrick**
Records: 1 **Literature:** Tempère and Peragallo (1908) **Syn:** *Navicula limosa* Kützing

(M) ***Caloneis westii*** **(W.Smith) Hendey**
Records: 4 **Literature:** Bailey and MacKay (1916), Clemens (1933), Tempère and Peragallo (1908), Tynni (1986) **Syn:** *Navicula formosa* W.Gregory

(FB) ***Gyrosigma acuminatum*** **(Kützing) Rabenhorst**
Records: 15 **Literature:** Buchanan (1966), Roelofs (1983) **Molecular (amplicon):** IMERSS (ENA:ERS27214058) **Syn:** *Gyrosigma spenceri* (Bailey ex Quekett) Griffith & Henfrey

(F) ***Gyrosigma arcuatum*** **(Donkin) Sterrenburg ✦**
Records: 3 **Morphology:** WL: iNat (254242421) Voucher (WL-SEM-3)

(F) ***Gyrosigma attenuatum*** **(Kützing) Rabenhorst**
Records: 1 **Literature:** Clemens (1933) **Syn:** *Gyrosigma hippocampus* Hassall, nom. illeg. (as '*hippocampa*')

(M) ***Gyrosigma balticum*** **(Ehrenberg) Rabenhorst**
Records: 3 **Literature:** Clemens (1933), Shim (1976), Tempère and Peragallo (1908)

(M) ***Gyrosigma californicum*** **(Grunow) G.Reid**
Records: 1 **Literature:** Bailey and MacKay (1916) **Syn:** *Pleurosigma balticum* var. *californicum* Grunow

(M) ***Gyrosigma fasciola*** **(Ehrenberg) J.W.Griffith & Henfrey**
Records: 9 **Literature:** Bailey and MacKay (1916), Buchanan (1966), Gran and Angst (1931), Lord (1866), Phifer (1932), Rao and Lewin (1976), Roelofs (1983), Waters et al. (1992) **Syn:** *Ceratoneis fasciola* Ehrenberg, *Pleurosigma fasciola* (Ehrenberg) W.Smith

(M) ***Gyrosigma fasciola*** **var.** ***sulcatum*** **(Grunow) Cleve**
Records: 2 **Literature:** Clemens (1933), Shim (1976)

(M) ***Gyrosigma fasciola*** **var.** ***tenuirostris*** **(Grunow) Cleve**
Records: 2 **Literature:** Roelofs (1983), Shim (1976) **Syn:** *Pleurosigma tenuirostris* Grunow (as '*Pleurosigma* [*fasciola* var.?] *tenuirostris*')

(M) ***Gyrosigma limosum*** **Sterrenburg & Underwood**
Records: 1 **Molecular (amplicon):** EBI (ENA:SRS2340122)

(F) ***Gyrosigma scalproides*** **(Rabenhorst) Cleve**
Records: 1 **Literature:** Shim (1976)

(M) ***Gyrosigma strigilis*** **(W.Smith) J.W.Griffin & Henfrey**
Records: 3 **Literature:** Roelofs (1983), Shim (1976), Waters et al. (1992)

(F) ***Haslea*** **cf.** ***avium*** **(M.E.Tiffany, Herwig & Sterrenburg) Yuhang Li & Kuidong Xi**
Records: 32 **Molecular (amplicon):** IMERSS (ENA:ERS21395352)

(M) ***Haslea*** **cf.** ***howeana*** **(Hagelstein) Giffen**
Records: 56 **Molecular (amplicon):** IMERSS (ENA:ERS21395352)

(M) ***Haslea*** **cf.** ***stundlii*** **(Hustedt) Blanco, Borrego-Ramos & Olenici**
Records: 46 **Molecular (amplicon):** IMERSS (ENA:ERS21395352)

(M) ***Haslea crucigera*** **(W.Smith) Simonsen**
Records: 38 **Literature:** Lobban (1985) **Morphology:** WL: iNat (257302461) Voucher (WL-SEM-50, WL-LM-27), M (184908 / 463130 / E 6388) **Molecular (amplicon):** EBI (ENA:ERS2393374), IMERSS (ENA:ERS21395346) **Syn:** *Navicula crucigera* W.Sm.

(M) ***Haslea gigantea*** **(Hustedt) Simonsen**
Records: 1 **Literature:** Shim (1976)

(M) ***Haslea pseudostrearia*** **Massé, Rincé & E.J.Cox**
Records: 13 **Molecular (amplicon):** IMERSS (ENA:ERS27214058)

(M) *Haslea spicula* (Hickie) Bukhtiyarova
Records: 1 **Literature:** Buchanan (1966) **Syn:** *Stauroneis spicula* Hickie
(M) *Haslea wawrikae* (Husedt) Simonsen
Records: 1 **Literature:** Shim (1976)
(F) *Hippodonta hungarica* (Grunow) Lange-Bertalot, Metzeltin & Witkowski
Records: 1 **Literature:** Tempère and Peragallo (1908)
(M) *Membraneis challengeri* (Grunow) Paddock
Records: 16 **Literature:** Chester et al. (1979), Clemens (1933), Johnson (1931), Shim (1976) **Morphology:** WL: iNat (4856515), Hakai (Hakai_phyto_QU39_2017-10-11_1m_17) **Notes:** reports of *Tropidoneis antarctica* var. *polyplasta* Gran & Angst (variety not confirmed) by Shim (1976) and Chester et al. (1979) most likely refer to *Membraneis challengeri*, a diatom we observe frequently **Syn:** *Navicula challengeri* Grunow
(M) *Navicula abunda* Hustedt
Records: 1 **Literature:** Rao and Lewin (1976)
(M) *Navicula agnita* Hustedt
Records: 1 **Literature:** Rao and Lewin (1976)
(FM) *Navicula ammophila* Grunow
Records: 1 **Literature:** Bailey and MacKay (1916)
(FM) *Navicula apiculata* Brébisson
Records: 2 **Literature:** Bailey and MacKay (1916), Clemens (1933)
(B) *Navicula arenaria* Donkin
Records: 23 **Literature:** Waters et al. (1992) **Molecular (amplicon):** IMERSS (ENA:ERS27217975)
(M) *Navicula arenicola* Grunow
Records: 1 **Literature:** Bailey and MacKay (1916)
(F) *Navicula aurora* Sovereign
Records: 1 **Literature:** Tempère and Peragallo (1908) **Syn:** *Navicula peregrina* var. *truncata* M.Peragallo
(F) *Navicula avenacea* (Rabenhorst) Brébisson ex Grunow
Records: 1 **Literature:** Bailey and MacKay (1916) **Syn:** *Navicula viridula* var. *avenacea* (Brébisson) Van Heurck
(B) *Navicula bottnica* Grunow
Records: 9 **Literature:** Bailey and MacKay (1916) **Morphology:** BRU (ABRU00000299), MSC (MSC0183585), MU (000179642), NY (02114395) **Syn:** *Schizonema smithii* C.Agardh
(FM) *Navicula brunelii* Poulin, Hudon & A.Cardinal
Records: 13 **Molecular (amplicon):** IMERSS (ENA:ERS27214058)
(M) *Navicula cancellata* Donkin
Records: 4 **Literature:** Bailey and MacKay (1916), Clemens (1933), Rao and Lewin (1976), Tempère and Peragallo (1908)
(M) *Navicula* cf. *agatkae* Witkowski, Lange-Bertalot & Metzeltin
Records: 14 **Molecular (amplicon):** IMERSS (ENA:ERS21395359)
(F) *Navicula* cf. *annexa* Hustedt
Records: 1 **Literature:** Roelofs (1983)
(M) *Navicula* cf. *criophiliforma* Witkowski, Riaux-Gobin & Daniszewska-Kowalczyk
Records: 8 **Molecular (amplicon):** IMERSS (ENA:ERS21395345)
(M) *Navicula* cf. *flagellifera* Hustedt
Records: 40 **Molecular (amplicon):** IMERSS (ENA:ERS21395356)
(M) *Navicula* cf. *septentrionalis* Cleve
Records: 1 **Morphology:** WL: iNat (4874617)
(FM) *Navicula cincta* (Ehrenberg) Ralfs
Records: 1 **Literature:** Bailey and MacKay (1916)
(M) *Navicula cluthensis* f. *rostrata* (Simonsen) Hustedt
Records: 1 **Literature:** Rao and Lewin (1976)

(M) ***Navicula concordia*** **Riaux-Gobin & Witkowski**
Records: 28 **Molecular (amplicon):** IMERSS (ENA:ERS27630061)
(M) ***Navicula crucifera*** **Grunow**
Records: 1 **Literature:** Rao and Lewin (1976)
(M) ***Navicula cryptocephala*** **Kützing**
Records: 4 **Literature:** Bailey and MacKay (1916), Buchanan (1966), Rao and Lewin (1976), Tempère and Peragallo (1908)
(F) ***Navicula delicatissima*** **Hustedt ex Simonsen**
Records: 1 **Literature:** Chester et al. (1979)
(M) ***Navicula digitoradiata*** **(W.Gregory) Ralfs**
Records: 5 **Literature:** Buchanan (1966), Rao and Lewin (1976), Roelofs (1983), Tempère and Peragallo (1908), Tynni (1986)
(F) ***Navicula digitoradiata*** **var.** ***cyprinus*** **(Ehrenberg) Van Heurck**
Records: 3 **Literature:** Bailey and MacKay (1916), Clemens (1933), Tempère and Peragallo (1908)
(M) ***Navicula directa*** **(W.Smith) Brébisson**
Records: 7 **Literature:** Bailey and MacKay (1916), Chester et al. (1979), Clemens (1933), Rao and Lewin (1976), Shim (1976), Tempère and Peragallo (1908), Tynni (1986)
(M) ***Navicula distans*** **(W.Smith) Brébisson**
Records: 5 **Literature:** Chester et al. (1979), Clemens (1933), Gran and Angst (1931), McQuoid and Hobson (1997), McQuoid and Hobson (2001)
(M) ***Navicula diversistriata*** **Hustedt**
Records: 2 **Literature:** Rao and Lewin (1976), Roelofs (1983)
(B) ***Navicula flanatica*** **Grunow**
Records: 2 **Literature:** Bailey and MacKay (1916), Rao and Lewin (1976)
(FMB) ***Navicula gregaria*** **Donkin**
Records: 2 **Molecular (amplicon):** IMERSS (ENA:ERS27630061)
(B) ***Navicula heufleri*** **Grunow**
Records: 1 **Literature:** Bailey and MacKay (1916)
(M) ***Navicula incus*** **Grunow**
Records: 1 **Literature:** Bailey and MacKay (1916) **Syn:** *Navicula directa* var. *incus* (Grunow) Cleve
(F) ***Navicula kuetzingiana*** **H.L.Smith**
Records: 1 **Literature:** Clemens (1933)
(M) ***Navicula longa*** **(W.Gregory) Ralfs**
Records: 2 **Literature:** Rao and Lewin (1976), Tempère and Peragallo (1908)
(F) ***Navicula ludloviana*** **A.W.F.Schmidt**
Records: 1 **Literature:** Tempère and Peragallo (1908)
(FM) ***Navicula menisculus*** **Schumann**
Records: 1 **Literature:** Rao and Lewin (1976) **Notes:** reported as unrecognized '*Navicula menisculus* var. *schumanni* Cleve-Euler'
(M) ***Navicula meniscus*** **Schumann**
Records: 1 **Literature:** Bailey and MacKay (1916) **Syn:** *Navicula peregrina* var. *meniscus* (Schumann) Grunow
(M) ***Navicula mollis*** **(W.Smith) Cleve**
Records: 2 **Literature:** Bailey and MacKay (1916), Clemens (1933) **Notes:** orth. corr. '*Navicula molle* Smith'
(M) ***Navicula northumbrica*** **Donkin**
Records: 2 **Literature:** Bailey and MacKay (1916), Clemens (1933)
(M) ***Navicula palpebralis*** **Brébisson ex W.Smith**
Records: 4 **Literature:** Clemens (1933), Roelofs (1983), Shim (1976), Tynni (1986)
(M) ***Navicula palpebralis*** **var.** ***semiplena*** **(Greville) Cleve**
Records: 1 **Literature:** Bailey and MacKay (1916)
(M) ***Navicula pennata*** **A.W.F.Schmidt**

Records: 2 **Literature:** Bailey and MacKay (1916), Tempère and Peragallo (1908)

(B) ***Navicula peregrina*** **(Ehrenberg) Kützing**

Records: 1 **Literature:** Tynni (1986)

(FM) ***Navicula perminuta*** **Grunow**

Records: 133 **Literature:** Tynni (1986) **Molecular (amplicon):** IMERSS (ENA:ERS21395352) **Syn:** *Navicula diserta* Hustedt

(FM) ***Navicula phyllepta*** **Kützing**

Records: 1 **Literature:** Bailey and MacKay (1916) **Syn:** *Navicula lanceolata* var. *phyllepta* (Kützing) van Heurck

(F) ***Navicula radiosa*** **Kützing**

Records: 1 **Literature:** Tempère and Peragallo (1908)

(F) ***Navicula ramosissima*** **f.** ***amplior*** **(Kützing) Cleve**

Records: 1 **Literature:** Bailey and MacKay (1916)

(F) ***Navicula reinhardtii*** **(Grunow) Grunow**

Records: 1 **Literature:** Tempère and Peragallo (1908)

(M) ***Navicula rhynchocephala*** **Kützing**

Records: 3 **Literature:** Buchanan (1966), Roelofs (1983), Shim (1976)

(F) ***Navicula rhynchocephala*** **var.** ***elongata*** **Grunow**

Records: 1 **Literature:** Rao and Lewin (1976)

(FM) ***Navicula rostellata*** **Kützing**

Records: 4 **Literature:** Bailey and MacKay (1916), Rao and Lewin (1976), Roelofs (1983), Tempère and Peragallo (1908) **Notes:** reported as unknown variety '*N. rostellata* var. *major*' by Roelofs (1983) **Syn:** *Navicula viridula* var. *rostellata* (Kützing) Cleve;

(M) ***Navicula rusticensis*** **Lobban**

Records: 2 **Morphology:** UBC (A032507)

(B) ***Navicula salinarum*** **Grunow**

Records: 3 **Literature:** Rao and Lewin (1976), Shim (1976), Tynni (1986) **Molecular (amplicon):** IMERSS (ENA:ERS21395345)

(FMB) ***Navicula salinicola*** **Hustedt**

Records: 14 **Literature:** Tynni (1986) **Molecular (amplicon):** IMERSS (ENA:ERS27214058)

(M) ***Navicula scabriuscula*** **(Cleve & Grove) Mereschkowsky**

Records: 1 **Literature:** Bailey and MacKay (1916) **Syn:** *Amphora scabriuscula* Cleve & Grove

(M) ***Navicula spuria*** **Cleve**

Records: 1 **Literature:** Bailey and MacKay (1916)

(M) ***Navicula subinflatoides*** **Hustedt**

Records: 1 **Literature:** Rao and Lewin (1976)

(M) ***Navicula triplex*** **VanLandingham**

Records: 1 **Literature:** Clemens (1933) **Syn:** *Navicula hendeyi* D.M.Williams, nom. illeg.

(F) ***Navicula tripunctata*** **(O.F.Müller) Bory**

Records: 3 **Literature:** Bailey and MacKay (1916), Rao and Lewin (1976), Tynni (1986) **Syn:** *Navicula gracilis* Ehrenberg, *Navicula neglecta* (Thwaites) Petit, nom. illeg.

(F) ***Navicula viridula*** **(Kützing) Ehrenberg**

Records: 1 **Literature:** Shim (1976)

(F) ***Placogeia kriegeri*** **(Krasske) Bukhtiyarova**

Records: 1 **Literature:** Rao and Lewin (1976) **Syn:** *Navicula kriegeri* Krasske

(F) ***Placogeia similis*** **(Krasske) Bukhtiyarova**

Records: 1 **Literature:** Rao and Lewin (1976) **Syn:** *Navicula similis* Krasske

(M) ***Pseudogomphonema kamtschaticum*** **(Grunow) Medlin**

Records: 4 **Literature:** Rao and Lewin (1976) **Morphology:** WL: iNat (189379380), M (186371 / 460200 / E 3726) **Molecular (amplicon):** IMERSS (ENA:ERS27217975) **Syn:** *Gomphonema kamtschaticum* Grunow

(FM) ***Seminavis macilenta*** **(W.Gregory) D.B.Danielidis & D.G.Mann**
Records: 3 **Literature:** Bailey and MacKay (1916), Clemens (1933), Rao and Lewin (1976) **Syn:** *Amphora macilenta* W.Gregory

(M) ***Seminavis robusta*** **D.B.Danielidis & D.G.Mann ✦**
Records: 99 **Morphology:** WL: iNat (263684273) Voucher (WL-LM-44) **Molecular (amplicon):** IMERSS (ENA:ERS21395352)

(M) ***Seminavis ventricosa*** **(W.Gregory) M.Garcia-Baptista**
Records: 2 **Literature:** Lord (1866), Tempère and Peragallo (1908) **Syn:** *Amphora angusta* var. *ventricosa* (W.Gregory) Cleve, *Amphora ventricosa* W.Gregory

(M) ***Trachyneis aspera*** **(Ehrenberg) Cleve**
Records: 9 **Literature:** Bailey and MacKay (1916), Clemens (1933), Lord (1866), Rao and Lewin (1976), Roelofs (1983), Shim (1976), Tempère and Peragallo (1908), Tynni (1986) **Morphology:** WL: iNat (190443004) Voucher (WL-SEM-35, WL-LM-39) **Syn:** *Stauroneis pulchella* W.Smith, *Trachyneis aspera* var. *pulchella* (W.Smith) Cleve

(M) ***Trachyneis*** **cf.** ***clepsydra*** **(Donkin) Cleve**
Records: 1 **Literature:** Bailey and MacKay (1916)

(M) ***Trachyneis intermedium*** **(Grunow ex A.W.F.Schmidt) L.W.Bailey**
Records: 1 **Literature:** Tempère and Peragallo (1908) **Syn:** *Trachyneis aspera* var. *intermedia* (Grunow ex A.W.F.Schmidt) Cleve

(M) ***Trachyneis vulgaris*** **(Cleve) Bailey**
Records: 1 **Literature:** Bailey and MacKay (1916) **Syn:** *Trachyneis aspera* var. *vulgaris* Cleve

Phylum: Bacillariophyta
Class: Bacillariophyceae
Subclass: Bacillariophycidae
Order: Naviculales
Family: Neidiaceae

(F) ***Neidium affine*** **var.** ***amphirhynchus*** **(Grunow) H.Peragallo & M.Peragallo**
Records: 1 **Literature:** Tempère and Peragallo (1908) **Syn:** *Navicula amphirhynchus* Ehrenberg

(F) ***Neidium ampliatum*** **(Ehrenberg) Krammer**
Records: 1 **Literature:** Tempère and Peragallo (1908) **Syn:** *Navicula iridis* var. *ampliata* (Ehrenberg) Dippel

(F) ***Neidium bisulcatum*** **var.** ***subundulatum*** **(Grunow) Reimer**
Records: 1 **Literature:** Roelofs (1983)

(F) ***Neidium dilatatum*** **(Ehrenberg) Cleve**
Records: 1 **Literature:** Tempère and Peragallo (1908) **Syn:** *Navicula dilatata* Ehrenberg

(F) ***Neidium hitchcockii*** **(Ehrenberg) Cleve**
Records: 1 **Literature:** Tempère and Peragallo (1908) **Syn:** *Navicula hitchcockii* Ehrenberg

Phylum: Bacillariophyta
Class: Bacillariophyceae
Subclass: Bacillariophycidae
Order: Naviculales
Family: Pinnulariaceae

(F) ***Pinnularia alpina*** **W.Smith**
Records: 1 **Literature:** Tempère and Peragallo (1908) **Syn:** *Navicula alpina* (W.Smith) Grunow

(F) ***Pinnularia apiculata*** **Gregory**
Records: 1 **Literature:** Bailey and MacKay (1916) **Syn:** *Navicula cancellata* var. *apiculata* (Gregory) Peragallo & Peragallo
(F) ***Pinnularia biceps*** **W.Gregory**
Records: 1 **Literature:** Roelofs (1983)
(F) ***Pinnularia cruciformis*** **(Donkin) Cleve**
Records: 3 **Literature:** Rao and Lewin (1976), Roelofs (1983), Tempère and Peragallo (1908)
(F) ***Pinnularia gentilis*** **(Donkin) Cleve**
Records: 1 **Literature:** Tempère and Peragallo (1908) **Syn:** *Navicula gentilis* Donkin
(F) ***Pinnularia gigas*** **Ehrenberg**
Records: 1 **Literature:** Tempère and Peragallo (1908) **Syn:** *Navicula gigas* (Ehrenberg) Kützing
(F) ***Pinnularia legumen*** **Ehrenberg**
Records: 1 **Literature:** Shim (1976)
(F) ***Pinnularia major*** **(Kützing) Rabenhorst**
Records: 1 **Literature:** Tempère and Peragallo (1908)
(F) ***Pinnularia microstauron*** **(Ehrenberg) Cleve**
Records: 2 **Literature:** Roelofs (1983), Tynni (1986)
(F) ***Pinnularia nobilis*** **(Ehrenberg) Ehrenberg**
Records: 1 **Literature:** Tempère and Peragallo (1908)
(F) ***Pinnularia peragalloi*** **Kulikovskiy, Lange-Bertalot & Metzeltin**
Records: 1 **Literature:** Tempère and Peragallo (1908) **Syn:** *Navicula divergens* var. *bacillaris* M.Peragallo
(F) ***Pinnularia quadratarea*** **var.** ***stuxbergii*** **(Grunow) H.Peragallo & M.Peragallo (AlgaeBase: (Cleve) Cleve)**
Records: 1 **Literature:** Tempère and Peragallo (1908) **Syn:** *Navicula stuxbergii* Cleve (as '*Stuxbergii*')
(F) ***Pinnularia transversa*** **(Cleve) Ant.Mayer**
Records: 2 **Literature:** Shim (1976), Tempère and Peragallo (1908)
(F) ***Pinnularia viridis*** **(Nitzsch) Ehrenberg**
Records: 1 **Literature:** Tempère and Peragallo (1908) **Syn:** *Navicula viridis* (Nitzsch) Ehrenberg

Phylum: Bacillariophyta
Class: Bacillariophyceae
Subclass: Bacillariophycidae
Order: Naviculales
Family: Plagiotropidaceae
(M) ***Meuniera membranacea*** **(Cleve) P.C.Silva**
Records: 1 **Literature:** Buchanan (1966) **Syn:** *Stauroneis membranacea* (Cleve) Hustedt
(M) ***Plagiotropis lepidoptera*** **(W.Gregory) Kuntze**
Records: 5 **Literature:** Bailey and MacKay (1916), Clemens (1933), Shim (1976), Tempère and Peragallo (1908) **Morphology:** WL: iNat (50272686) **Syn:** *Amphiprora lepidoptera* W.Gregory, *Tropidoneis lepidoptera* (W.Gregory) Cleve
(M) ***Plagiotropis lepidoptera*** **var.** ***proboscidea*** **(Grunow) H.Peragallo & M.Peragallo**
Records: 1 **Literature:** Tempère and Peragallo (1908) **Syn:** *Tropidoneis lepidoptera* var. *proboscidea* Cleve
(M) ***Plagiotropis vitrea*** **(W.Smith) Grunow**
Records: 2 **Literature:** Rao and Lewin (1976) **Morphology:** M (186195 / 458883 / E 2521) **Syn:** *Tropidoneis vitrea* (W.Smith) Cleve

Phylum: Bacillariophyta
Class: Bacillariophyceae
Subclass: Bacillariophycidae
Order: Naviculales
Family: Pleurosigmataceae

(M) *Pleurosigma acutum* Norman ex Ralfs
Records: 2 **Literature:** Forbes and Waters (1993), Shim (1976)

(M) *Pleurosigma aestuarii* (Brébisson ex Kützing) W.Smith
Records: 1 **Literature:** Tempère and Peragallo (1908)

(M) *Pleurosigma affine* Grunow
Records: 1 **Literature:** Tempère and Peragallo (1908)

(M) *Pleurosigma angulatum* (J.T.Quekett) W.Smith
Records: 2 **Literature:** Clemens (1933), Shim (1976)

(M) *Pleurosigma decorum* W.Smith
Records: 4 **Literature:** Clemens (1933), Tempère and Peragallo (1908)
Morphology: WL: iNat (198948244) Voucher (WL-SEM-8, WL-LM-17)

(M) *Pleurosigma delicatulum* W.Smith
Records: 2 **Literature:** Bailey and MacKay (1916), Clemens (1933) **Syn:** *Pleurosigma angulatum* var. *delicatulum* (W.Smith) van Heurck

(M) *Pleurosigma elongatum* W.Smith
Records: 4 **Literature:** Buchanan (1966), Shim (1976), Stockner and Cliff (1975), Waters et al. (1992)

(M) *Pleurosigma formosum* W.Smith
Records: 5 **Literature:** Clemens (1933), Gran and Angst (1931), Johnson (1931), Lord (1866), Shim (1976)

(M) *Pleurosigma formosum* var. *longissimum* Grunow
Records: 2 **Literature:** Shim (1976), Tempère and Peragallo (1908)

(M) *Pleurosigma inflatum* Shadbolt
Records: 1 **Literature:** Lord (1866) **Syn:** *Pleurosigma naviculaceum* Brébisson

(M) *Pleurosigma intermedium* W.Smith
Records: 16 **Literature:** Bailey and MacKay (1916), Clemens (1933), Lord (1866), Shim (1976), Tempère and Peragallo (1908) **Morphology:** WL: iNat (198983502) Voucher (WL-LM-37) **Molecular (amplicon):** EBI (ENA:ERS2393374), IMERSS (ENA:ERS21395345) **Syn:** *Pleurosigma nubecula* W.Smith

(M) *Pleurosigma longum* Cleve
Records: 3 **Literature:** Roelofs (1983), Shim (1976), Tempère and Peragallo (1908)

(M) *Pleurosigma longum* var. *lanceolatum* H. Peragallo & M. Peragallo
Records: 2 **Literature:** Bailey and MacKay (1916), Clemens (1933)

(M) *Pleurosigma nicobaricum* Grunow
Records: 1 **Literature:** Buchanan (1966)

(M) *Pleurosigma normanii* Ralfs
Records: 4 **Literature:** Buchanan (1966), Shim (1976) **Morphology:** WL: iNat (4873106)

(M) *Pleurosigma rigidum* W.Smith
Records: 2 **Literature:** Bailey and MacKay (1916), Clemens (1933)

(M) *Pleurosigma speciosum* W.Smith
Records: 1 **Literature:** Lord (1866)

(M) *Pleurosigma strigosum* W.Smith
Records: 4 **Literature:** Bailey and MacKay (1916), Clemens (1933), Rao and Lewin (1976), Tempère and Peragallo (1908)

(M) *Rhoicosigma arcticum* Cleve
Records: 1 **Literature:** Rao and Lewin (1976) **Syn:** *Gyrosigma arcticum* (Cleve) Cleve

(M) *Rhoicosigma compactum* (Greville) Grunow

Records: 1 **Literature:** Buchanan (1966)

Phylum: Bacillariophyta
Class: Bacillariophyceae
Subclass: Bacillariophycidae
Order: Naviculales
Family: Proschkiniaceae
(M) ***Proschkinia complanatoides*** **(Hustedt ex Simonsen) D.G.Mann**
Records: 1 **Literature:** Shim (1976) **Syn:** *Navicula complanatoides* Hustedt ex Simonsen
(M) ***Proschkinia complanatula*** **(Hustedt ex Simonsen) D.G.Mann ✦**
Records: 1 **Morphology:** WL: iNat (188679253) Voucher (WL-SEM-105) **Molecular (amplicon):** IMERSS (ENA:ERS21395345)

Phylum: Bacillariophyta
Class: Bacillariophyceae
Subclass: Bacillariophycidae
Order: Naviculales
Family: Scoliotropidaceae
(FMB) ***Biremis ambigua*** **(Cleve) D.G.Mann**
Records: 1 **Literature:** Rao and Lewin (1976) **Syn:** *Pinnularia ambigua* Cleve
(M) ***Scoliotropis latestriata*** **(Brébisson ex Kützing) Cleve**
Records: 1 **Literature:** Tempère and Peragallo (1908)

Phylum: Bacillariophyta
Class: Bacillariophyceae
Subclass: Bacillariophycidae
Order: Naviculales
Family: Sellaphoraceae
(M) ***Fallacia aequorea*** **(Hustedt) D.G.Mann**
Records: 1 **Literature:** Rao and Lewin (1976) **Syn:** *Navicula aequorea* Hustedt
(M) ***Fallacia*** **cf.** ***hodgeana*** **(R.M.Patrick & Freese) Y.H.Li & H.Suzuki**
Records: 2 **Molecular (amplicon):** IMERSS (ENA:ERS27630061)
(M) ***Fallacia floriniae*** **(M.Møller) Witkowski**
Records: 4 **Literature:** Roelofs (1983), Tynni (1986) **Morphology:** WL: iNat (297936805) **Syn:** *Navicula pseudony* Hustedt
(M) ***Fallacia forcipata*** **(Greville) Stickle & D.G.Mann**
Records: 2 **Literature:** Clemens (1933), Roelofs (1983) **Syn:** *Navicula forcipata* Greville
(M) ***Fallacia forcipata*** **var.** ***densistriata*** **(A.W.F.Schmidt) Gogorev**
Records: 1 **Literature:** Rao and Lewin (1976)
(F) ***Fallacia insociabilis*** **(Krasske) D.G.Mann**
Records: 1 **Literature:** Rao and Lewin (1976) **Syn:** *Navicula insociabilis* Krasske
(M) ***Fallacia litoricola*** **(Hustedt) D.G.Mann**
Records: 3 **Literature:** Rao and Lewin (1976) **Molecular (amplicon):** IMERSS (ENA:ERS21395352) **Syn:** *Navicula litoricola* Hustedt
(M) ***Fallacia nummularia*** **(Greville) D.G.Mann**
Records: 1 **Literature:** Bailey and MacKay (1916) **Syn:** *Navicula forcipata* var. *nummularia* (Greville) Cleve
(F) ***Sellaphora pseudobacillum*** **(Grunow) Lange-Bertalot & Metzeltin**
Records: 1 **Literature:** Tempère and Peragallo (1908)
(FM) ***Sellaphora pupula*** **(Kützing) Mereschkovsky**
Records: 2 **Literature:** Roelofs (1983), Tempère and Peragallo (1908) **Syn:** *Navicula pupula* Kützing
(F) ***Sellaphora pupula*** **f.** ***capitata*** **(Skvortzov & K.I.Meyer) Poulin**

Records: 1 **Literature:** Roelofs (1983) **Syn:** *Navicula pupula* var. *capitata* Skvortzov & K.I.Meyer

Phylum: Bacillariophyta
Class: Bacillariophyceae
Subclass: Bacillariophycidae
Order: Rhopalodiales
Family: Rhopalodiaceae

(F) *Epithemia adnata* (Kützing) Brébisson
Records: 2 **Literature:** Bailey and MacKay (1916), Tempère and Peragallo (1908) **Syn:** *Epithemia zebra* (Ehrenberg) Kützing (as '*zaebra*')

(F) *Epithemia argus* (Ehrenberg) Kützing
Records: 1 **Literature:** Buchanan (1966)

(F) *Epithemia hyndmanii* W.Smith
Records: 1 **Literature:** Tempère and Peragallo (1908)

(F) *Epithemia sorex* Kützing
Records: 1 **Literature:** Tempère and Peragallo (1908)

(FB) *Epithemia turgida* (Ehrenberg) Kützing
Records: 4 **Literature:** Roelofs (1983), Shim (1976), Tempère and Peragallo (1908), Tynni (1986)

(F) *Epithemia turgida* var. *granulata* (Ehrenberg) Brun
Records: 1 **Literature:** Tempère and Peragallo (1908)

(F) *Epithemia turgida* var. *westermannii* (Ehrenberg) Grunow
Records: 1 **Literature:** Tempère and Peragallo (1908) **Notes:** orth. corr. '*Epithemia Westermannii*'

(F) *Rhopalodia gibba* (Ehrenberg) O.Müller
Records: 2 **Literature:** Shim (1976), Tempère and Peragallo (1908) **Syn:** *Epithemia gibba* (Ehrenberg) Kützing

(FM) *Rhopalodia gibba* var. *ventricosa* (Kützing) H.Peragallo & M.Peragallo
Records: 1 **Literature:** Tynni (1986) **Syn:** *Epithemia ventricosa* Kützing

(FM) *Rhopalodia gibberula* (Ehrenberg) O.Müller
Records: 1 **Literature:** Tynni (1986) **Syn:** *Epithemia gibberula* (Ehrenberg) Kützing

(B) *Rhopalodia musculus* (Kützing) O.Müller
Records: 2 **Literature:** Bailey and MacKay (1916), Roelofs (1983) **Syn:** *Epithemia musculus* Kützing

Phylum: Bacillariophyta
Class: Bacillariophyceae
Subclass: Bacillariophycidae
Order: Surirellales
Family: Entomoneidaceae

(M) *Entomoneis alata* (Ehrenberg) Ehrenberg
Records: 4 **Literature:** Bailey and MacKay (1916), Clemens (1933), Tempère and Peragallo (1908), Tynni (1986) **Notes:** reported as *Amphiprora alata* var. *intermedia* Cleve (unverified) by Guiry and Guiry 2025 **Syn:** *Amphiprora alata* (Ehrenberg) Kützing

(M) *Entomoneis alata* f. *minor* Ehrenberg
Records: 1 **Literature:** Tempère and Peragallo (1908) **Syn:** *Amphiprora alata* f. *minor* Tempère & Peragallo, nom. inval., nomen nudum

(M) *Entomoneis gigantea* (Grunow) Nizamuddin
Records: 1 **Literature:** Buchanan (1966) **Syn:** *Amphiprora gigantea* Grunow

(M) *Entomoneis ornata* (Bailey) Reimer
Records: 1 **Literature:** Tempère and Peragallo (1908) **Syn:** *Amphiprora ornata* Bailey

(FM) *Entomoneis paludosa* (W.Smith) Reimer

Records: 2 **Literature:** Tynni (1986), Waters et al. (1992) **Syn:** *Amphiprora paludosa* W.Smith

Phylum: Bacillariophyta

Class: Bacillariophyceae

Subclass: Bacillariophycidae

Order: Surirellales

Family: Surirellaceae

(M) *Campylodiscus bicostatus* W.Smith ex Roper ✦

Records: 1 **Morphology:** WL: iNat (189680990) Voucher (WL-LM-42)

(F) *Campylodiscus costatus* W.Smith

Records: 1 **Literature:** Tempère and Peragallo (1908)

(M) *Campylodiscus ecclesianus* Greville

Records: 1 **Literature:** Tempère and Peragallo (1908)

(M) *Campylodiscus fastuosus* Ehrenberg

Records: 2 **Literature:** Bailey and MacKay (1916), Lord (1866) **Syn:** *Campylodiscus parvulus* W.Smith (as '*C. parrulus*'), *Surirella fastuosa* (Ehrenberg) Ehrenberg

(M) *Campylodiscus latus* Shadboldt

Records: 1 **Literature:** Shim (1976)

(M) *Campylodiscus neofastuosus* Ruck & Nakov

Records: 3 **Literature:** Buchanan (1966), Clemens (1933) **Morphology:** WL: iNat (255455760) Voucher (WL-LM-19) **Syn:** *Surirella fastuosa* (Ehrenberg) Ehrenberg

(M) *Campylodiscus thuretii* Brébisson

Records: 3 **Literature:** Bailey and MacKay (1916), Clemens (1933), Shim (1976)

(F) *Coronia limbata* (Brébisson) Ruck & Guiry

Records: 1 **Literature:** Shim (1976) **Syn:** *Campylodiscus limbatus* Brébisson

(F) *Iconella bifrons* (Ehrenberg) Ruck & Nakov

Records: 1 **Literature:** Tempère and Peragallo (1908) **Syn:** *Surirella bifrons* (Ehrenberg) Ehrenberg (as '*Surirella* (*Suriraya*) *bifrons*')

(F) *Iconella biseriata* (Ehrenberg) Ruck & Yakov

Records: 1 **Literature:** Tempère and Peragallo (1908) **Syn:** *Surirella biseriata* Brébisson (as '*Surirella* (*Suriraya*) *biseriata*')

(F) *Iconella elegans* (Ehrenberg) Bukhtiyarova

Records: 1 **Literature:** Tynni (1986) **Syn:** *Surirella elegans* Ehrenberg

(F) *Iconella linearis* (W.Smith) Ruck & Nakov

Records: 1 **Literature:** Tempère and Peragallo (1908) **Syn:** *Surirella linearis* W.Smith

(F) *Iconella nervosa* (A.W.F.Schmidt) C.Cocquyt & R.Jahn

Records: 1 **Literature:** Tempère and Peragallo (1908) **Syn:** *Surirella tenera* var. *nervosa* A.W.F.Schmidt

(F) *Iconella rectangulata* Ruck & Nakov

Records: 1 **Literature:** Lord (1866) **Syn:** *Campylodiscus striatus* Ehrenberg ex Kützing

(F) *Iconella robusta* (Ehrenberg) Ruck & Nakov

Records: 2 **Literature:** Shim (1976), Tempère and Peragallo (1908) **Syn:** *Navicula robusta* Ehrenberg (as '*Navicula* (*Surirella*) *robusta*')

(F) *Iconella splendida* (Ehrenberg) Ruck & Nakov

Records: 2 **Literature:** Buchanan (1966), Tempère and Peragallo (1908)

(FM) *Iconella tenera* (W.Gregory) Ruck & Nakov

Records: 1 **Literature:** Tempère and Peragallo (1908) **Syn:** *Surirella tenera* W.Gregory (as '*Surirella tenera?*')

(M) *Petrodictyon gemma* (Ehrenberg) D.G.Mann

Records: 2 **Literature:** Lord (1866), Tempère and Peragallo (1908) **Syn:** *Surirella gemma* (Ehrenberg) Kützing

(F) *Stenopterobia intermedia* (F.W.Lewis) Van Heurck ex Hanna
Records: 2 **Literature:** Roelofs (1983), Shim (1976) **Syn:** *Surirella intermedia* F.W.Lewis

(FM) *Surirella arabica* Grunow
Records: 1 **Morphology:** M (186983 / 461903 / E 5276)

(M) *Surirella crumena* Brébisson ex Kützing
Records: 1 **Literature:** Tynni (1986) **Syn:** *Surirella ovata* var. *crumena* (Brébisson ex Kützing) Van Heurck

(M) *Surirella frickei* Hustedt
Records: 1 **Morphology:** M (186631 / 461556 / E 4960)

(M) *Surirella hybrida* Grunow
Records: 1 **Literature:** Tempère and Peragallo (1908)

(M) *Surirella japonica* A.W.F.Schmidt
Records: 2 **Literature:** Bailey and MacKay (1916), Clemens (1933)

(F) *Surirella kittonii* A.W.F.Schmidt
Records: 1 **Literature:** Tempère and Peragallo (1908)

(F) *Surirella kittonii* var. *asperula* (Grunow) H.Peragallo & M.Peragallo
Records: 1 **Literature:** Tempère and Peragallo (1908)

(M) *Surirella lata* W.Smith
Records: 2 **Literature:** Bailey and MacKay (1916), Clemens (1933)

(F) *Surirella librile* (Ehrenberg) Ehrenberg
Records: 1 **Literature:** Tempère and Peragallo (1908) **Syn:** *Cymatopleura solea* (Brébisson) W.Smith

(F) *Surirella microlibrile* Van de Vijver, Pottiez & Jüttner
Records: 1 **Literature:** Tempère and Peragallo (1908) **Syn:** *Cymatopleura apiculata* W.Smith

(M) *Surirella recedens* A.W.F.Schmidt
Records: 1 **Literature:** Roelofs (1983) **Syn:** *Surirella fastuosa* var. *recedens* (A.W.F.Schmidt) Cleve

(M) *Surirella regina* Janisch
Records: 1 **Literature:** Tempère and Peragallo (1908)

(M) *Surirella striatula* Turpin
Records: 1 **Literature:** Tynni (1986)

Phylum: Bacillariophyta
Class: Bacillariophyceae
Subclass: Bacillariophycidae
Order: Thalassiophysales
Family: Catenulaceae

(M) *Amphora abludens* Simonsen
Records: 1 **Literature:** Rao and Lewin (1976)

(M) *Amphora acuta* W.Gregory
Records: 1 **Literature:** Tempère and Peragallo (1908)

(M) *Amphora arcuata* A.W.F.Schmidt
Records: 1 **Literature:** Tempère and Peragallo (1908)

(M) *Amphora arcus* W.Gregory
Records: 1 **Literature:** Clemens (1933)

(M) *Amphora arenaria* Donkin
Records: 1 **Literature:** Tempère and Peragallo (1908)

(M) *Amphora arenicola* Grunow ex Cleve
Records: 2 **Literature:** Bailey and MacKay (1916), Clemens (1933)

(M) *Amphora binodis* W.Gregory
Records: 2 **Literature:** Bailey and MacKay (1916), Clemens (1933) **Syn:** *Amphora binodis* var. *bigibba* (Grunow) H.Peragallo & M.Peragallo

(M) ***Amphora*** **cf.** ***vixvisibilis*** **Chunlian Li & Witkowski**
Records: 13 **Molecular (amplicon):** IMERSS (ENA:ERS27214058)
(M) ***Amphora cingulata*** **Cleve**
Records: 1 **Literature:** Tempère and Peragallo (1908)
(FM) ***Amphora costata*** **var.** ***inflata*** **(Grunow) H.Peragallo & M.Peragallo**
Records: 1 **Literature:** Tempère and Peragallo (1908)
(M) ***Amphora dubia*** **W.Gregory**
Records: 2 **Literature:** Bailey and MacKay (1916), Clemens (1933)
(M) ***Amphora graeffeana*** **Hendey**
Records: 1 **Literature:** Shim (1976) **Syn:** *Amphora graeffei* Cleve, nom. illeg. (as '*graeffii*')
(M) ***Amphora helenensis*** **Giffen**
Records: 26 **Molecular (amplicon):** IMERSS (ENA:ERS27214058)
(M) ***Amphora laevis*** **W.Gregory**
Records: 1 **Literature:** Rao and Lewin (1976)
(M) ***Amphora laevissima*** **W.Gregory**
Records: 1 **Literature:** Rao and Lewin (1976) **Syn:** *Amphora laevis* var. *laevissima* (W.Gregory) Cleve
(M) ***Amphora lanceolata*** **Ehrenberg**
Records: 1 **Literature:** Shim (1976)
(M) ***Amphora latecingulata*** **(Grunow) H.*****Peragallo in*** **Tempère & Peragallo**
Records: 1 **Literature:** Tempère and Peragallo (1908)
(M) ***Amphora libyca*** **var.** ***baltica*** **(Brander) A.Cleve**
Records: 1 **Literature:** Rao and Lewin (1976)
(M) ***Amphora lineolata*** **(Ehrenberg) Ehrenberg**
Records: 1 **Literature:** Rao and Lewin (1976)
(M) ***Amphora marina*** **W.Smith**
Records: 1 **Literature:** Bailey and MacKay (1916)
(F) ***Amphora mexicana*** **A.W.F.Schmidt**
Records: 2 **Literature:** Rao and Lewin (1976)
(M) ***Amphora obtusa*** **W.Gregory**
Records: 1 **Literature:** Tempère and Peragallo (1908)
(M) ***Amphora ocellata*** **var.** ***bistriata*** **(Grunow) H.Peragallo & M.Peragallo**
Records: 1 **Literature:** Tempère and Peragallo (1908) **Syn:** *Amphora bistriata* H.Peragallo & M.Peragallo, nom. illeg.
(FM) ***Amphora ovalis*** **(Kützing) Kützing**
Records: 7 **Literature:** Bailey and MacKay (1916), Clemens (1933), Lord (1866), McQuoid and Hobson (1997), McQuoid and Hobson (2001), Roelofs (1983), Tempère and Peragallo (1908) **Syn:** *Amphora ovalis* (Kützing) Kützing
(M) ***Amphora proteus*** **W.Gregory**
Records: 5 **Literature:** Bailey and MacKay (1916), Roelofs (1983), Tynni (1986) **Morphology:** M (186994 / 461908 / E 5280), NHMUK (Diatom BM Adams TS.630) **Notes:** orth. corr. '*Amphora prateus*'
(M) ***Amphora proteus*** **var.** ***oculata*** **H.Peragallo & M.Peragallo**
Records: 2 **Literature:** Rao and Lewin (1976), Tempère and Peragallo (1908)
(M) ***Amphora pusio*** **Cleve**
Records: 2 **Literature:** Clemens (1933), Shim (1976)
(M) ***Amphora pusio*** **var.** ***parvula*** **(Flögel) H.Peragallo & M.Peragallo**
Records: 1 **Literature:** Bailey and MacKay (1916)
(M) ***Amphora truncata*** **W.Gregory**
Records: 1 **Literature:** Tynni (1986)
(M) ***Halamphora acutiuscula*** **(Kützing) Levkov**
Records: 2 **Literature:** Clemens (1933), Rao and Lewin (1976) **Syn:** *Amphora acutiuscula* Kützing
(M) ***Halamphora clara*** **(A.W.F.Schmidt) Levkov**

Records: 1 **Literature:** Bailey and MacKay (1916) **Syn:** *Amphora clara* A.W.F.Schmidt

(B) ***Halamphora coffeiformis*** **(C.Agardh) Mereschkowsky**
Records: 3 **Literature:** Bailey and MacKay (1916), Rao and Lewin (1976) **Morphology:** WL: iNat (4874695) **Syn:** *Amphora coffeiformis* (C.Agardh) Kützing, *Amphora salina* W.Smith

(M) ***Halamphora costata*** **(W.Smith) Levkov**
Records: 1 **Literature:** Bailey and MacKay (1916)

(M) ***Halamphora cymbifera*** **(W.Gregory) Levkov**
Records: 2 **Literature:** Clemens (1933), Rao and Lewin (1976) **Syn:** *Amphora cymbifera* W.Gregory

(B) ***Halamphora eunotia*** **(Cleve) Levkov**
Records: 3 **Literature:** Bailey and MacKay (1916), Clemens (1933), Roelofs (1983) **Syn:** *Amphora eunotia* Cleve

(M) ***Halamphora exigua*** **(W.Gregory) Levkov**
Records: 2 **Literature:** Bailey and MacKay (1916), Shim (1976) **Syn:** *Amphora exigua* W.Gregory

(M) ***Halamphora granulata*** **(Gregory) Levkov**
Records: 1 **Literature:** Roelofs (1983) **Syn:** *Amphora granulata* W.Gregory

(B) ***Halamphora holsatica*** **(Hustedt) Levkov**
Records: 1 **Literature:** Rao and Lewin (1976) **Syn:** *Amphora holsatica* Hustedt

(M) ***Halamphora hyalina*** **(Kützing) Rimet & R.Jahn**
Records: 1 **Literature:** Bailey and MacKay (1916) **Syn:** *Amphora hyalina* Kützing

(B) ***Halamphora nagumoi*** **J.G.Stepanek, Mayama & Kociolek**
Records: 3 **Molecular (amplicon):** IMERSS (ENA:ERS21395345)

(B) ***Halamphora turgida*** **(W.Gregory) Levkov**
Records: 2 **Literature:** Bailey and MacKay (1916), Clemens (1933) **Syn:** *Amphora turgida* W.Gregory

(M) ***Tetramphora sulcata*** **(Brébisson) Stepanek & Kociolek**
Records: 13 **Molecular (amplicon):** IMERSS (ENA:ERS27214058)

Phylum: Bacillariophyta
Class: Bacillariophyceae
Subclass: Eunotiophycidae
Order: Eunotiales
Family: Eunotiaceae

(F) ***Actinella punctata*** **F.W.Lewis**
Records: 1 **Literature:** Buchanan (1966)

(F) ***Eunophora berggrenii*** **(Cleve) D.G.Mann, M.A.Harper & Levkov**
Records: 1 **Literature:** Tynni (1986) **Syn:** *Amphora berggrenii* Cleve

(F) ***Eunotia arcus*** **Ehrenberg**
Records: 1 **Literature:** Roelofs (1983)

(F) ***Eunotia bidens*** **Ehrenberg**
Records: 1 **Literature:** Tempère and Peragallo (1908) **Syn:** *Eunotia praerupta* var. *bidens* (Ehrenberg) Grunow

(F) ***Eunotia exigua*** **(Brébisson ex Kützing) Rabenhorst**
Records: 1 **Literature:** Roelofs (1983)

(F) ***Eunotia major*** **(W.Smith) Rabenhorst**
Records: 1 **Literature:** Rao and Lewin (1976) **Syn:** *Eunotia monodon* var. *major* (W.Smith) Hustedt (as '*maior*')

(F) ***Eunotia minor*** **(Kützing) Grunow**
Records: 1 **Literature:** Roelofs (1983)

(F) ***Eunotia monodon*** **Ehrenberg**
Records: 1 **Literature:** Tempère and Peragallo (1908)

(F) ***Eunotia sudetica*** **O.Müller**

Records: 2 **Literature:** Roelofs (1983), Tempère and Peragallo (1908)

Incertae sedis (placement uncertain)

Phylum: Bacillariophyta
Class: incertae sedis
Subclass: incertae sedis
Order: incertae sedis
Family: incertae sedis
(M) ***Andrzeja fenestrata*** **Mayama & Kryk ✦**
Records: 2 **Morphology:** WL: iNat (331455851) Voucher (WL-SEM-60-3)

Table S1. Salish Sea diatom records by source: historical literature, molecular data, and morphological data. Material that is uniquely contributed through this research is marked 'WL' (Webber Lab) and 'IMERSS'. Accession numbers are provided for all molecular data submitted to the European Nucleotide Archive (ENA).

Type	Source	Records	Unique taxa
Literature	Various works cited in annotated checklist	2259	815
Molecular	IMERSS – 2023 Galiano BioBlitz (amplicon) (ENA accession: PRJEB102646)	1287	55
Molecular	Ecological genomics of a seasonally anoxic fjord; Saanich Inlet – data derived from the European Bioinformatics Institute (amplicon) (MGnify 2021)	474	43
Molecular	IMERSS – Plankton samples (amplicon) (ENA accession: PRJEB102646)	432	40
Molecular	*Zostera* epiphyte samples (amplicon) (Jackman et al. 2025) (ENA accession: PRJEB72893)	428	69
Molecular	IMERSS – *Scagelia* epiphyte sample (amplicon) (ENA accession: PRJEB102646)	63	41
Molecular	IMERSS – Diatom clones (Sanger) (ENA accession: PRJEB102646)	6	3
Morphology	Phytoplankton community composition links to environmental drivers across a fjord to shelf gradient on the central coast of British Columbia (Del Bel Belluz et al. 2024)	2354	45
Morphology	Harmful algae in the Strait of Georgia, 2015–2023 (Esenkulova and Salinas-Ruiz 2024)	1338	1
Morphology	WL – Morphological data (LM and SEM micrographs, iNaturalist observations) generated by Webber Lab (iNaturalist 2025)	317	174
Morphology	Continuous plankton recorder survey (CPR Survey) – Marine Biological Association (Lear 2022)	201	20
Morphology	University of British Columbia Herbarium (UBC) – Algae Collection (University of British Columbia Herbarium 2024)	58	5
Morphology	The Diatom Collection of Franz Josef Weinzierl at the Botanische Staatssammlung München (M) (Staatliche Naturwissenschaftliche Sammlungen Bayerns 2025)	17	16
Morphology	Harmful Algal Event Database (HAEDAT) (Provoost and Enevoldsen 2025)	12	2
Morphology	Brown University Herbarium (BRU) (Brown University Herbarium 2025)	6	4
Morphology	Michigan State University Herbarium (MSC) (Michigan State University Herbarium 2025)	6	5
Morphology	W.S. Turrell Herbarium, Miami University (MU) (W.S. Turrell Herbarium 2025)	6	5
Morphology	The New York Botanical Garden (NY) (Ramirez et al. 2025)	4	3
Morphology	University and Jepson Herbaria, University of California, Berkeley (UC) (Alexander and Gross 2024)	4	1
Morphology	Natural History Museum (London) (NHMUK) (Natural History Museum 2025)	2	2
Morphology	Florida Museum of Natural History (FLAS) (Florida Museum of Natural History 2025)	1	1
Morphology	University of New Hampshire Nature History Collections (NHA) (University of New Hampshire Nature History Collections 2025)	1	1

References

- Alexander J, Gross J (2024) The University and Jepson herbaria -- algae. Occurrence dataset. Version 1.12. Berkeley Natural History Museums, Berkeley, California, USA. URL: https://doi.org/10.15468/2axacp
- Bailey LW, MacKay AH (1916) Diatoms from the eastern coast of Vancouver Island. Transactions of the Royal Society of Canada, series 3, 9 (4): 141-174.
- Bérard-Therriault L, Cardinal A, Poulin M (1987) Les diatomées (Bacillariophyceae) benthiques de substrats durs des eaux marines et saumâtres du Québec. 8. Centrales. Le Naturaliste canadien 114: 81-103.
- Bérard-Therriault L, Poulin M, Bossé L (1999) Guide d'identification du phytoplancton marin de l'estuaire et du Golfe du Saint-Laurent incluant également certains protozoaires. Publication spéciale canadienne des sciences halieutiques et aquatiques 128. National Research Council Canada, Ottawa, Canada, 387 pp.
- Brown University Herbarium (2025) Brown University - algae. Occurrence dataset. Brown University, Providence, Rhode Island, USA. URL: https://doi.org/10.15468/kpsj8r
- Buchanan RJ (1966) A study of the species composition and ecology of the protoplankton of a British Columbia inlet. PhD thesis. University of British Columbia, Vancouver, Canada, 301 pp. https://doi.org/10.14288/1.0104566
- Chester AJ, Damkaer DM, Dey DB, Heron GA, Larrance JD (1979) Plankton of the Strait of Juan De Fuca 1976–1977. Pacific Marine Environmental Laboratory, Seattle, USA; National Oceanic and Atmospheric Administration, United States Environmental Protection Agency, Washington, DC, USA.
- Clemens WA (1933) A check list of the marine fauna and flora of the Canadian Pacific coast. National Research Council of Canada, Ottawa, Canada, 89 pp. https://doi.org/10.4224/21273380
- Del Bel Belluz J, Jackson JM, Kellogg CTE, Peña MA, Giesbrecht IJW, Hobson LA (2024) Phytoplankton community composition links to environmental drivers across a fjord to shelf gradient on the central coast of British Columbia. Frontiers in Marine Science 11: 1458677. https://doi.org/10.3389/fmars.2024.1458677
- Esenkulova S, Salinas-Ruiz P (2024) Harmful algae in the Strait of Georgia, 2015–2023. Sampling event dataset. Version 1.9. Pacific Salmon Foundation. https://doi.org/10.48689/736c4721-db8e-4116-a5f8-d9084126b135
- Florida Museum of Natural History (2025) University of Florida Herbarium - macroalgae. Occurrence dataset. Florida Museum of Natural History, Gainesville, Florida, USA. URL: https://doi.org/10.15468/q69whp
- Forbes JR, Waters RE (1993) Phytoplankton species composition and abundance along the Pacific coast of Canada, 1979–1989. Volume 1: 1979–1984. Canadian Data Report of Hydrography and Ocean Sciences 117. Department of Fisheries and Oceans, Sidney, Canada, 212 pp.
- Godhe A, McQuoid MR, Karunasagar I, Karunasagar I, Rehnstam-Holm AS (2006) Comparison of three common molecular tools for distinguishing among geographically separated clones of the diatom *Skeletonema marinoi* Sarno et Zingone (Bacillariophyceae). Journal of Phycology 42 (2): 280-291. https://doi.org/10.1111/j.1529-8817.2006.00197.x
- Gran HH, Angst EC (1931) Plankton diatoms of Puget Sound. Publications–Puget Sound Biological Station 7: 417-514.
- Gucluer SM, Gross MG (1964) Recent marine sediments in Saanich Inlet, a stagnant marine basin. Limnology and Oceanography 9 (3): 359-376. https://doi.org/10.4319/lo.1964.9.3.0359
- Guiry MD, Guiry GM (2025) AlgalBase. https://www.algaebase.org. Accessed on: 2025-10-20.
- Haigh R, Taylor FJR, Sutherland TF (1992) Phytoplankton ecology of Sechelt Inlet, a fjord system on the British Columbia coast. I. General features of the nano- and microplankton. Marine Ecology Progress Series 89: 117-134. https://doi.org/10.3354/meps089117

- Harrison PJ, Clifford PJ, Cochlan WP, Yin Y, St John MA, Thompson PA, Sibbald MJ, Albright LJ (1991) Nutrient and phytoplankton dynamics in the Fraser River plume, Strait of Georgia, British Columbia. Marine Ecology Progress Series 70: 291-304. https://doi.org/10.3354/meps070291
- Hasle GR, Syvertsen EE (1997) Marine diatoms. In: Thomas CR (Ed.) Identifying marine phytoplankton. Academic Press, San Diego, USA, 874 pp. https://doi.org/10.17031/1629
- Hernández-Becerril DU, Tapia Peña MI (1995) Planktonic diatoms from the Gulf of California and coasts off Baja California: species of the genus *Thalassiosira*. Botanica Marina 38 (1–6): 543-555. https://doi.org/10.1515/botm.1995.38.1-6.543
- Hobson LA (1983) Phytoplankton crops, bacterial metabolism and oxygen in Saanich Inlet, a fjord in Vancouver Island, British Columbia. Sedimentary Geology 36 (2–4): 117-130. https://doi.org/10.1016/0037-0738(83)90005-2
- Hobson LA, McQuoid MR (2001) Pelagic diatom assemblages are good indicators of mixed water intrusions into Saanich Inlet, a stratified fjord in Vancouver Island. Marine Geology 174 (1–4): 125-138. https://doi.org/10.1016/S0025-3227(00)00146-8
- Hollibaugh JT, Seibert DLR, Thomas WH (1980) A comparison of the acute toxicities of ten heavy metals to phytoplankton from Saanich Inlet. Estuarine and Coastal Marine Science 10 (1): 93-105. https://doi.org/10.1016/S0302-3524(80)80052-1
- Honeywill C (1998) A study of British *Licmophora* species and a discussion of its morphological features. Diatom Research 13 (2): 221-271. https://doi.org/10.1080/0269249X.1998.9705450
- Hoppenrath M, Beszteri B, Drebes G, Halliger H, Van Beusekom JEE, Janisch S, Wiltshire KH (2007) *Thalassiosira* species (Bacillariophyceae, Thalassiosirales) in the North Sea at Helgoland (German Bight) and Sylt (North Frisian Wadden Sea)—a first approach to assessing diversity. European Journal of Phycology 42 (3): 271-288. https://doi.org/10.1080/09670260701352288
- Horner RA, Postel JR, Halsband-Lenk C, Pierson JJ, Pohnert G, Wichard T (2005) Winter-spring phytoplankton blooms in Dabob Bay, Washington. Progress in Oceanography 67 (3–4): 286-313. https://doi.org/10.1016/j.pocean.2005.09.005
- iNaturalist (2025) iNaturalist research-grade observations. Occurrence dataset. iNaturalist.org. URL: https://doi.org/10.15468/ab3s5x
- Jackman AS, Schenk S, Adamczyk EM, Herrin MJ, van Asselt A, Humphrey E, Witkowski A, Morien E, Parfrey LW (2025) Integrating DNA sequencing and morphological identification enhances understanding of epiphytic diatom diversity and ecology. Botany 103: 1–14. https://doi.org/10.1139/cjb-2024-0100
- Johnson WL (1931) Seasonal distribution of plankton at Friday Harbour, Washington. Thesis. University of Washington, Seattle, USA, 77 pp.
- Kennedy OD, LeBrasseur R (1977) Microplankton in the Strait of Georgia: I. Pelagic diatoms in Departure Bay and adjacent waters. Manuscript Report, Fisheries Research Board of Canada 1418. Department of Fisheries and Oceans, Canada, 141 pp.
- Lear D (2022) Continuous plankton recorder survey (CPR survey). Sampling event dataset. Version 1.3. The Marine Biological Association of the UK, Devon, UK. URL: https://doi.org/10.17031/1629
- Légaré JE (1957) The qualitative and quantitative distribution of plankton in the Strait of Georgia in relation to certain oceanographic factors. Journal of the Fisheries Research Board of Canada 14 (4): 521-552. https://doi.org/10.1139/f57-015
- Li Y, Zhao QL, Lü SH (2014) Taxonomy and species diversity of the diatom genus *Thalassiosira* (Bacillariophyceae) in Zhejiang coastal waters, the East China Sea. Nova Hedwigia 99 (3-4): 373-402. https://doi.org/10.1127/0029-5035/2014/0170

- Lobban CS (1985) Marine tube-dwelling diatoms of the Pacific coast of North America. I. *Berkeleya*, *Haslea*, *Nitzschia*, and *Navicula* sect. *Microstigmaticae*. Canadian Journal of Botany 63 (10): 1779-1784. https://doi.org/10.1139/b85-249
- Lord JK (1866) The naturalist in Vancouver Island and British Columbia. In two volumes. Vol. II. Richard Bentley, London, England, 375 pp. https://doi.org/10.5962/bhl.title.48504
- McQuoid MR, Hobson LA (1997) A 91-year record of seasonal and interannual variability of diatoms from laminated sediments in Saanich Inlet, British Columbia. Journal of Plankton Research 19 (2): 173-194. https://doi.org/10.1093/plankt/19.2.173
- McQuoid MR, Hobson LA (2001) A Holocene record of diatom and silicoflagellate microfossils in sediments of Saanich Inlet, ODP Leg 169S. Marine Geology 174 (1-4): 111-123. https://doi.org/10.1016/S0025-3227(00)00145-6
- MGnify (2021) Ecological genomics of a seasonally anoxic fjord; Saanich Inlet. Sampling event dataset. European Molecular Biology Laboratory, European Bioinformatics Institute (EMBL-EBI), Cambridgeshire, UK. URL: https://doi.org/10.15468/yw9wpv
- Michigan State University Herbarium (2025) Michigan State University algae. Occurrence dataset. Michigan State University, East Lansing, Michigan, USA. URL: https://doi.org/10.15468/dey5s9
- Natural History Museum (2025) Natural History Museum, data portal: collection specimens. Occurrence dataset. Natural History Museum, London, UK. URL: https://doi.org/10.5519/qd.f4i40jj0
- Parsons TR, LeBrasseur RJ, Fulton JD (1967) Some observations on the dependence of zooplankton grazing on the cell size and concentration of phytoplankton blooms. Journal of the Oceanographical Society of Japan 23 (1): 10-17. https://doi.org/10.5928/kaiyou1942.23.10
- Peck JI, Harrington NR (1897) Observations on the plankton of Puget Sound. Transactions of the New York Academy of Sciences 16: 378-387.
- Phifer LD (1932) Seasonal distribution and occurrence of planktonic diatoms at Friday Harbor, Washington. PhD thesis. University of Washington, Seattle, USA, 120 pp.
- Powys RI (1987) The geochemistry and diatom assemblages of varved sediments from Saanich Inlet, B.C. MSc thesis. University of British Columbia, Vancouver, Canada, 200 pp. https://doi.org/10.14288/1.0053228
- Provoost P, Enevoldsen H (2025) Harmful algal event database (HAEDAT). Sampling event dataset. Version 2.2. Intergovernmental Oceanographic Commission–UNESCO Harmful Algal Bloom Programme. URL: https://doi.org/10.25607/0wdrmq
- Ramirez J, Watson K, Feder L, Gjieli E, Sessa E (2025) The New York Botanical Garden Herbarium (NY). Occurrence dataset. Version 1.79. The New York Botanical Garden, New York City, USA. URL: https://doi.org/10.15468/6e8nje
- Rao VN, Lewin J (1976) Benthic marine diatom flora of False Bay, San Juan Island, Washington. Syesis 9: 173-213.
- Roelofs AK (1983) The distribution of diatoms in the surface sediments of British Columbia Inlets. PhD thesis. University of British Columbia, Vancouver, Canada, 267 pp. https://doi.org/10.14288/1.0095908
- Round FE, Crawford RM, Mann DG (1990) The diatoms: biology and morphology of the genera. Cambridge University Press, Cambridge, UK, 747 pp.
- Sancetta C, Calvert SE (1988) The annual cycle of sedimentation in Saanich Inlet, British Columbia: implications for the interpretation of diatom fossil assemblages. Deep-Sea Research 35 (1): 71-90. https://doi.org/10.1016/0198-0149(88)90058-1
- Sancetta C (1989) Processes controlling the accumulation of diatoms in sediments: a model derived from British Columbian fjords. Paleoceanography 4 (3): 235-251. https://doi.org/10.1029/PA004i003p00235

- Sancetta C (1990) Occurrence of Thalassiosiraceae (Bacillariophyceae) in two fjords of British Columbia. In: Geissler U, Håkansson H, Miller U, Schmid AM (Eds) Contributions to the knowledge of microalgae, particularly diatoms. Nova Hedwigia, Beihefte 100. J. Cramer, Berlin, Germany, 300 pp.
- Shim JH (1976) Distribution and taxonomy of planktonic marine diatoms in the Strait of Georgia, B.C. PhD thesis. University of British Columbia, Vancouver, Canada, 248 pp. https://doi.org/10.14288/1.0093789
- Staatliche Naturwissenschaftliche Sammlungen Bayerns (2025) The diatom collection of Franz Josef Weinzierl at the Botanische Staatssammlung München. Occurrence dataset. The Bavarian State Collections of Natural History, Munich, Germany. URL: https://doi.org/10.15468/dixlft
- Stockner JG, Cliff DD (1975) Marine phytoplankton production, distribution and species composition in Pendrell and Hotham Sounds, British Columbia. Fisheries and Marine Service, Technical Report 516. Pacific Environment Institute, West Vancouver; Department of the Environment, Ottawa, Canada, 80 pp.
- Stockner JG, Cliff DD (1976) Phytoplankton succession and abundance in Howe Sound, British Columbia: a coastal marine embayment-fiord under stress. Fisheries and Marine Service, Technical Report 658. Pacific Environment Institute, West Vancouver; Department of the Environment, Ottawa, Canada, 24 pp.
- Stockner JG, Cliff DD (1979) Phytoplankton ecology of Vancouver Harbor. Journal of the Fisheries Research Board of Canada 36 (1): 1-10. https://doi.org/10.1139/f79-001
- Sutherland TF, Taylor FJ, Pond S (2023) Resting stage occurrence and auxospore formation of diatoms collected from fjordic sediments in Sechelt Inlet, British Columbia, Canada. Technical Report of Fisheries and Aquatic Sciences 3521. Department of Fisheries and Oceans, Ottawa, Canada, 21 pp. URL: https://publications.gc.ca/collections/collection_2023/mpo-dfo/Fs97-6-3521-eng.pdf
- Takano H (1980) New and rare diatoms from Japanese marine waters. V. *Thalassiosira tealata* sp. nov. Bulletin Tokai Regional Fisheries Research Laboratory 103: 55-63.
- Tempère J, Peragallo H (1908) Diatomées du monde entier. 2nd edition, fascicles 2-7. Arcachon, Gironde, France, 480 pp.
- Tynni R (1986) Observations of diatoms on the coast of the state of Washington. Geological Survey of Finland, Report of Investigation 75. Geological Survey of Finland, Espoo, Finland, 25 pp.
- University of British Columbia Herbarium (2024) University of British Columbia Herbarium (UBC) - algae collection. Occurrence dataset. Version 14.19. University of British Columbia, Vancouver, Canada. URL: https://doi.org/10.5886/ujwtvvs2
- University of New Hampshire Natural History Collections (2025) University of New Hampshire - macroalgal collection. Occurrence dataset. University of New Hampshire, Durham, New Hampshire, USA. URL: https://doi.org/10.15468/kembya
- Waters RE, Brown LN, Robinson MG (1992) Phytoplankton of Esquimalt Lagoon, British Columbia: comparison with west Vancouver Island coastal and offshore waters. Canadian. Technical Report of Hydrography Ocean Sciences 137. Department of Fisheries and Oceans, Victoria, Canada, 59 pp.
- W.S. Turrell Herbarium (2025) Miami University - algae. Occurrence dataset. Miami University, Oxford, Ohio, USA. URL: https://doi.org/10.15468/9xp52b
